# Carbosulfonylation of Alkynes: A Direct Conversion of sp-C to sp^3^-C through Visible Light-Mediated 3-Component Reaction

**DOI:** 10.1021/acs.orglett.4c02700

**Published:** 2024-09-11

**Authors:** Mandapati Bhargava Reddy, Vanessa E. Becker, Eoghan M. McGarrigle

**Affiliations:** † Centre for Synthesis & Chemical Biology, UCD School of Chemistry, Belfield, Dublin 4, Ireland; ‡ A2P CDT in Sustainable Chemistry and BiOrbic Bioeconomy SFI Research Centre, University College Dublin, Belfield, Dublin 4, Ireland

## Abstract

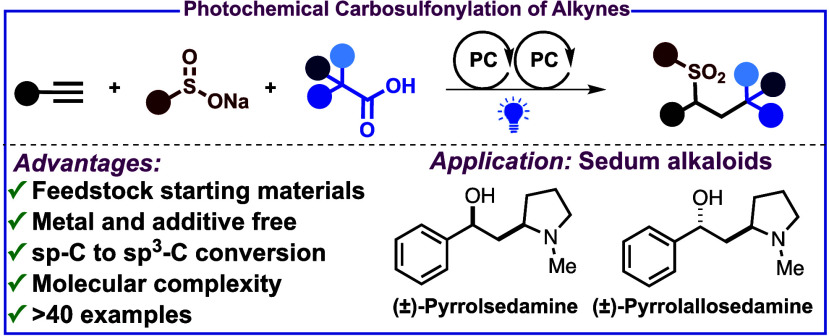

A 3-component metal-free carbosulfonylation of alkynes is reported using readily available alkyl carboxylic acids and arylsulfinates under visible light irradiation. This photochemical approach gives direct conversion of sp-C to sp^3^-C yielding highly functionalized alkyl sulfones. It employs feedstock chemicals as starting materials and shows a broad substrate scope and moderate diastereoselectivity. The method’s utility is highlighted in the synthesis of sedum alkaloids. A single photocatalyst is proposed to be active in two distinct photocatalytic cycles operating in tandem.

Alkyne difunctionalizations are some of the most straightforward approaches to construct highly functionalized and complex molecules in a single step.^1^ In particular, multicomponent difunctionalizations involving feedstock chemicals can convert alkynes into valuable organic compounds in a single step.^2^ In this context, decarboxylative carbofunctionalizations involving abundant feedstock chemicals and carboxylic acids have gained attention.^3^ MacMillan and Rueping have developed metallaphotoredox-catalyzed Markovnikov and anti-Markovnikov type hydroalkylations of alkynes with alkyl carboxylic acids to give alkene products.^4^ Despite recent advances, direct carbofunctionalizations of alkynes involving carboxylic acids are limited to 2-component couplings and they generally require metal catalysts and additives. Herein, we report a direct 3-component carbofunctionalization of alkynes using carboxylic acids and sulfinates to make alkyl sulfones.

Alkyl sulfones are versatile compounds due to their importance in material science, synthetic chemistry, and pharmaceuticals.^5^ Furthermore, alkyl sulfones have recently emerged as radical acceptors in photochemical reactions.^6^ Carbosulfonylation of alkynes is one of the simplest approaches to construct complex sulfone-containing compounds.^7^ In 2017, Nevado reported the nickel-catalyzed carbosulfonylation of alkynes with arylboronic acids and sulfonyl chlorides via sulfonyl radicals.^8^ Recently, Rueping developed a stereocontrolled carbosulfonylation of alkynes using aryl halides and aryl sulfinates under metallaphotoredox conditions (Scheme 1a).^9^ These two examples are sp-C to sp^2^-C carbosulfonylations yielding a new sp^2^-sp^2^ C–C bond. Very recently, Qiu reported sp^2^/sp-C to sp^3^-C alkylative carbosulfonylation of alkenes and alkynes to give products with sp^3^-sp^3^ C–C bonds, but using redox-active esters (Scheme 1b).^10^ These carbosulfonylation protocols are underexplored and generally require transition metal catalysts. Thus, an efficient metal-free carbosulfonylation of alkynes from readily available starting materials and abundant feedstock chemicals is highly desirable. We report here the development of such a method.

**Scheme 1:**
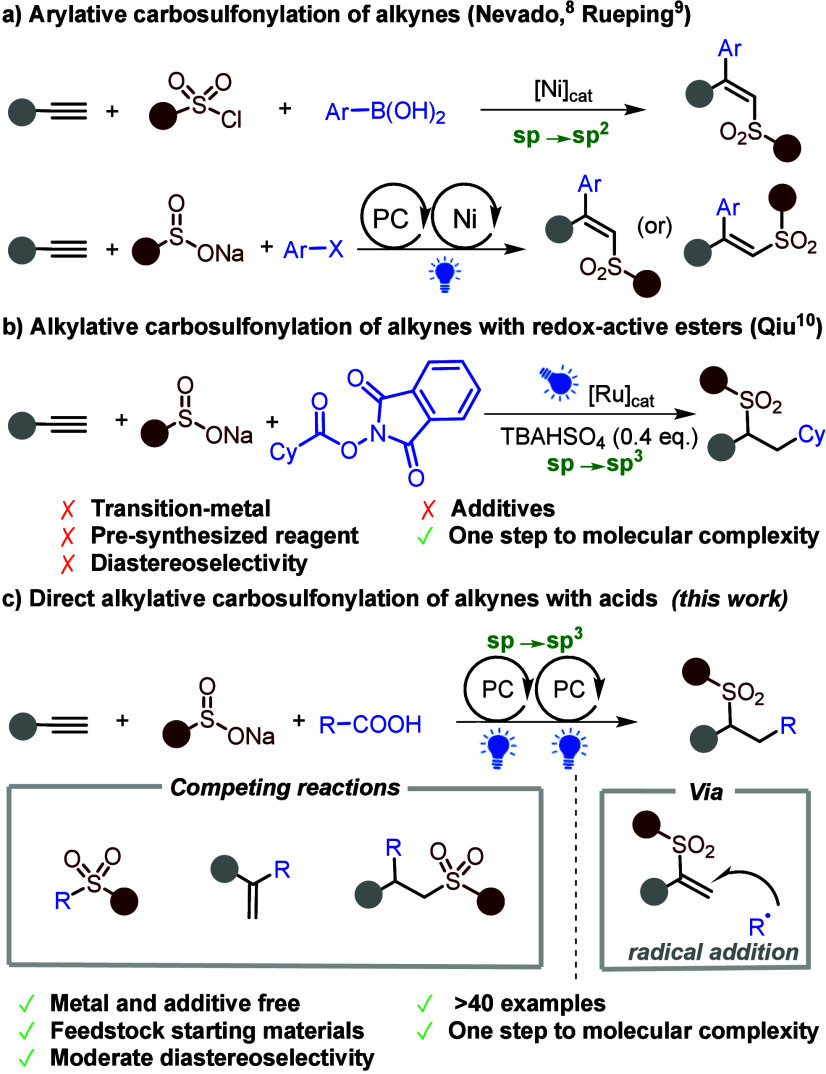
Approaches to Carbosulfonylation of Alkynes

Recently, organic organophotoredox catalysis has gained an important role in organic synthesis due to the broad range of substrates, scope of reactions, and as an alternative to transition metal catalysis.^11^ In particular, 4CzIPN and other similar organic photocatalysts have emerged as alternatives to more traditional Ir-based photocatalysts.^12^ These organophotocatalysts can generate alkyl radicals from carboxylic acids via single-electron transfer (SET).^13^ Inspired by previous results and to further explore our interest in metal-free sulfonylations,^14a,14b^ we have developed a highly regioselective carbosulfonylation of alkynes with readily available carboxylic acids and arylsulfinates to access sp^3^-rich valuable alkyl sulfones in a single step.^14c^

We began with 4-chlorophenylacetylene **1h**, *p*-toluenesulfinate **2a**, and Boc-l-proline **3a** as model substrates to develop a photochemical carbosulfonylation procedure (Table 1). After screening, it was found that visible light irradiation of a 1:2:2 mixture of **1h**:**2a**:**3a** with 4CzIPN as catalyst (1 mol %) in DMSO under nitrogen for 14 h gave the desired carbosulfonylated product **4h** in 82% spectroscopic yield in a 4:1 d.r. The major diastereomer was isolated in 63% yield (entry 1). A solvent screen identified DMSO as the best solvent (entries 1–4). The introduction of Cs_2_CO_3_ led to a lowered yield of product **4h** (entry 5). 4CzIPN was found to be the best photocatalyst, with 3DPAFIPN failing to produce the desired product **4h** and iridium-based photocatalysts being less efficient (entries 6–8). No further improvement was observed when catalyst loading was doubled (entry 9). Using air instead of a nitrogen atmosphere gave only 56% of desired product **4h** (entry 10). Both photocatalyst and blue light irradiation were confirmed to be required (entries 11, 12). For further optimization, see the Supporting Information (SI).

**Table 1:**
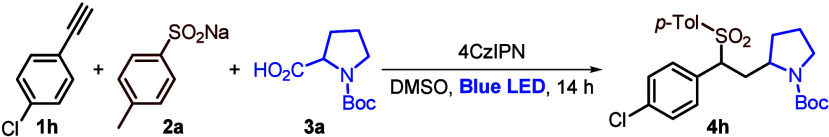

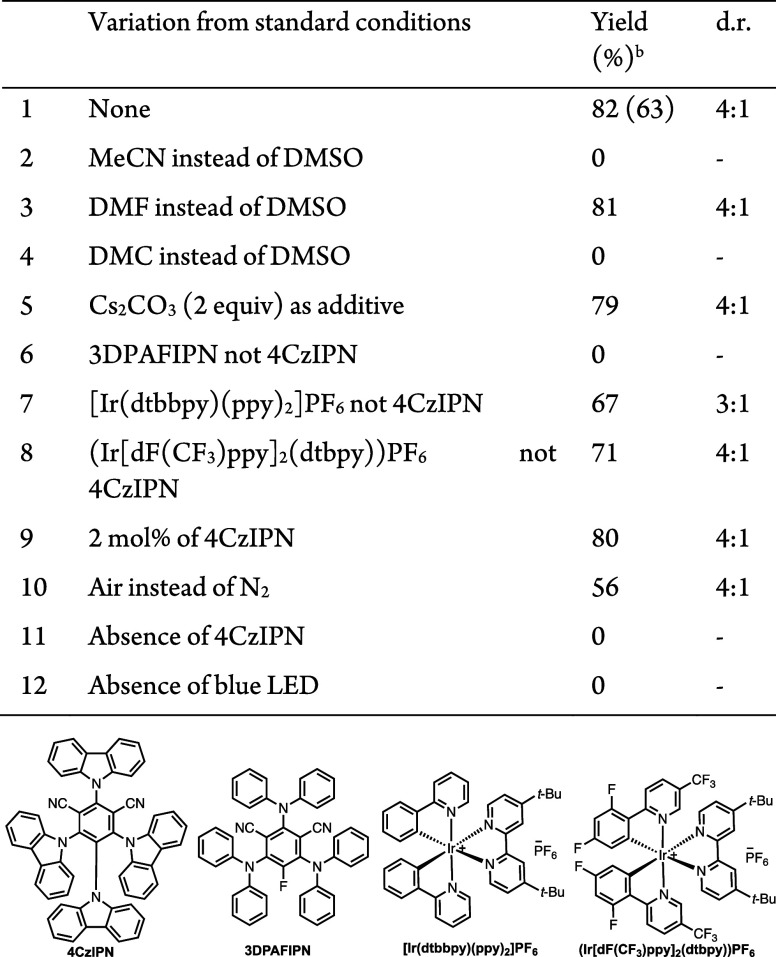
Optimization of Reaction Conditions^a^

a Reaction conditions: **1h** (0.1 mmol), **2a** (0.2 mmol), **3a** (0.2 mmol), and catalyst (1 mol %) in 2 mL solvent were irradiated with blue LED (456 nm, 40W) in the presence of N_2_ atmosphere.

b Yields and selectivity were determined by NMR with 1,3,5-trimethoxybenzene as an internal standard. Isolated yield of major diastereomer in parentheses. DMC = Dimethyl carbonate.

Having found suitable reaction conditions, we examined the generality of this method, starting by varying the alkyne (Scheme 2). The photochemical carbosulfonylation was successful with phenylacetylenes bearing electron-donating groups such as 4-Me and 4-OMe reacting with sodium *p*-toluenesulfinate **2a** and Boc-l-proline **3a** to give **4b** and **4c** with a 64% and 58% yield of the major diastereomer, respectively. 2-OMe and 3-NH_2_ substituted phenylacetylenes afforded **4d** and **4e** in 62% and 54% yield, respectively, as a mixture of diastereomers. Halogen-substituted phenylacetylenes (3-F, 2-Cl, and 4-Cl) were also suitable substrates for the carbosulfonylation reaction and gave the corresponding products **4f**–**4h** in good yields. Phenylacetylenes with electron-withdrawing groups such as 4-CN, 4-COOMe, −CF_3_, and 4-Ph effectively reacted to give **4i**–**4m** in moderate yields. In contrast, 4-NO_2_ and 4-CHO substituted phenylacetylenes failed to afford the desired products **4n** and **4o**. Gratifyingly, heteroaromatic-substituted alkynes bearing pyridine and thiophene rings and aliphatic alkynes were also well tolerated under this protocol, giving sulfones **4p**–**4s** in 45–65% yield.

**Scheme 2:**
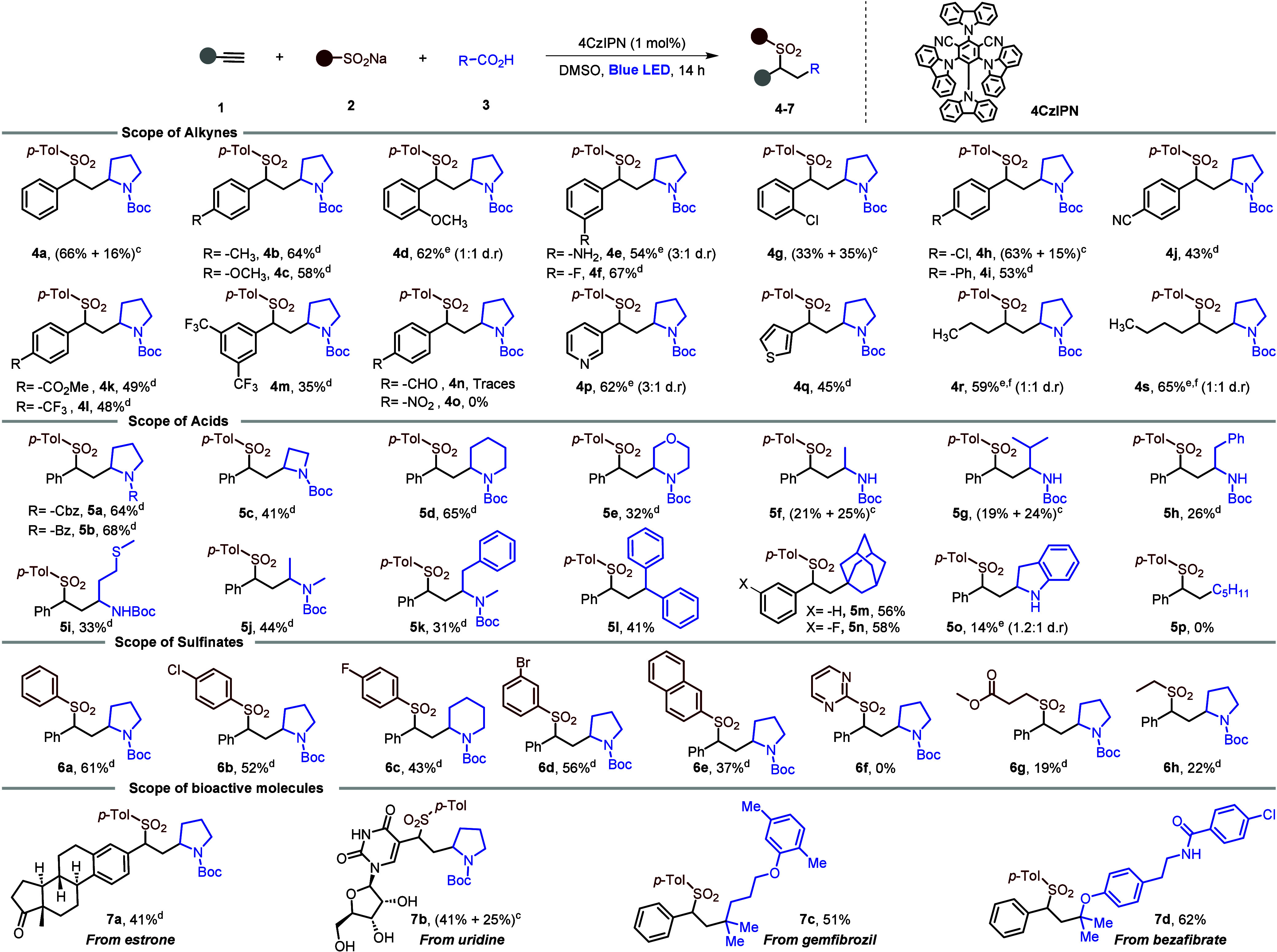
Reaction Scope Reaction conditions: **1** (0.1 mmol), **2** (0.2 mmol), **3** (0.2 mmol) and catalyst (1 mol %) in DMSO (2 mL) irradiation with blue LED (456 nm, 40W) under N_2_. Isolated yields. Isolated yields of both diastereomers. Isolated yield of major diastereomer (4:1 d.r.). Isolated yield of mixture of diastereomers. Isolated yield after Boc deprotection.

Next, we explored the scope with a range of alkyl carboxylic acids under the optimized reaction conditions (Scheme 2). Cyclic secondary carboxylic acids with a variety of protecting groups and ring sizes were suitable for this photochemical carbosulfonylation and gave the corresponding products **5a**–**5e** in good yields. To further expand the substrate scope of carboxylic acids, various acyclic amino acids were screened using our protocol. The desired products **5f**–**5k** were formed in moderate yields. To our delight, carboxylic acids without an α-heteroatom such as diphenylacetic acid and 1-adamantanecarboxylic acid also gave the desired products **5l**–**5n** in 41%, 56%, and 58% yield, respectively. Indoline-2-carboxylic acid gave the corresponding product **5o** in lower yield, but primary carboxylic acids such as hexanoic acid failed to produce the desired product **5p**. For primary carboxylic acids use of the redox-active ester in Qiu’s carbosulfonylation of *alkenes* provides the desired product.^10^ Finally, we explored the substrate scope with respect to sulfinates and late-stage modification of bioactive molecules. Aryl sulfinates bearing halogens in the *p*- or *m*-positions gave the corresponding products **6b**–**6d** in good yields. Sodium naphthalene-2-sulfinate gave the corresponding sulfone **6e** in moderate yield but pyrimidine sufinate failed to afford corresponding product **6f** under this protocol. Notably, sodium alkylsulfinates also reacted to give the desired products **6g** and **6h**, albeit in lower yield. Alkynes derived from bioactive molecules such as estrone and uridine gave the corresponding products in good yields. Further, bioactive molecules containing carboxylic acids such as gemfibrozil and bezafibrate also smoothly underwent the reaction and afforded the corresponding products in 51% and 62% yields, respectively. To demonstrate its utility, we applied our methodology to the synthesis of the sedum alkaloids pyrrolallosedamine and pyrrolsedamine (Scheme 3), natural products that are effective in the treatment of cognitive disorders.^15^

**Scheme 3:**
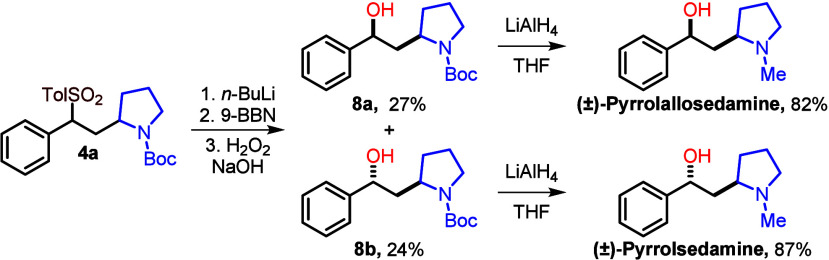
Application to the Synthesis of Sedum Alkaloids

To gain insights into the mechanism of the reaction, we performed a series of quenching and control experiments (Scheme 4 and see the SI). Addition of the radical scavenger TEMPO to standard reaction conditions stopped the production of **4a**, and the TEMPO adduct of both the tosyl radical and the alkyl radical were detected by HRMS (see SI). Treatment of alkyne **1a** with sulfinate **2a** under optimized conditions failed to give vinyl sulfinate **10**,^16^ but **10** was produced in the presence of sulfinic acid (Scheme 4a,b). The reaction of proposed vinyl sulfonate intermediate **10** with Boc-l-proline **3a** under optimized conditions failed to produce **4a** (Scheme 4c). In contrast, the reaction of **10** with presynthesized Boc-Pro-ONa (**3a-Na**) or sulfinate **2a** with Boc-l-proline **3a** gave the desired product **4a** in 58% and 62% yields, respectively (Scheme 4d,e). This reveals that sodium sulfinate **2a** plays a role in the generation of the alkyl radical in reaction mixture and supports a proposal that vinyl sulfinate **10** may be one of the intermediates of the reaction. This was further supported by reaction monitoring by NMR spectroscopy (SI Figure S3). When the disulfone compound **11** was synthesized and treated with acid **3a** in the presence of NaHCO_3_, the desired product **4a** was obtained in 20% yield (Scheme 4f), supporting our proposal of a bis-sulfonyl intermediate. In Stern–Volmer quenching experiments, the 4CzIPN was quenched by sulfinate **2a** and a mixture of both **2a** and acid **3a**; it failed to quench by acid **3a** alone, indicating the importance of sodium sulfinate to the generation of alkyl radical from **3a** (see SI).

**Scheme 4:**
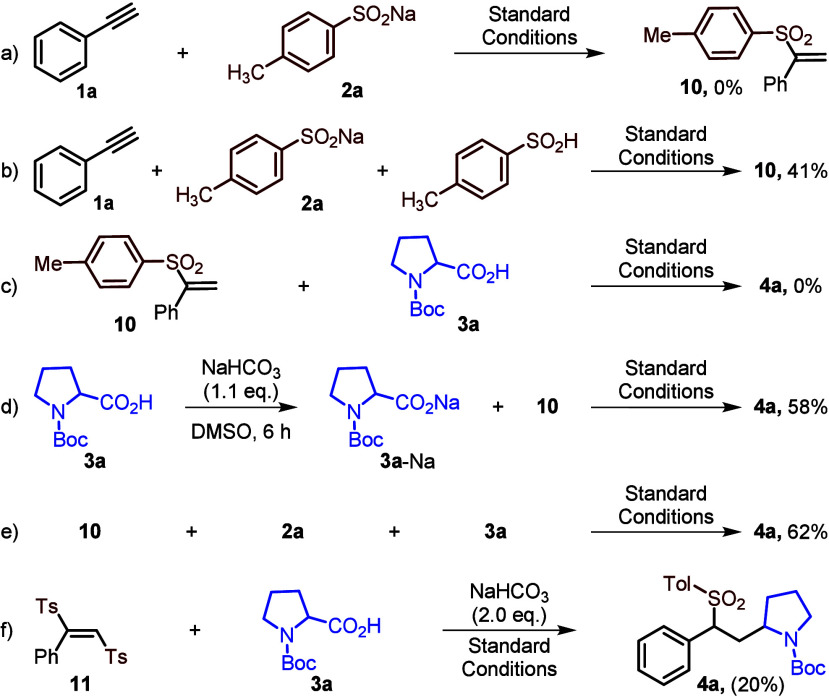
Mechanistic Investigations

Based on the above experiments and previous literature,^3c,6b,13,16,17^ we propose the reaction pathway shown in Scheme 5. Absorption of blue light generates the excited photocatalyst PC* which then oxidizes sodium sulfinate **2** to form sulfinyl radical **A** (*E*_1/2_ (PhO_2_S^•^/PhSO_2_Na) = −0.37 vs SCE) along with PC^•–^(*E*_1/2_ (PC*/PC^•–^) = +1.43 vs SCE).^18^ Then sulfinyl radical **A** reacts with the alkyne to form vinyl radical **B**.^8^ Sodium sulfinate **2** then reacts with vinyl radical **B** to form radical anion **C**.^17^ The radical anion **C** is protonated by acid **3a** to give radical **D** and the proline salt **3a-Na**. SET quenches the PC^•–^ and then the resulting anion undergoes elimination of sulfinate **2** to give vinyl sulfone **E**. This proposal may resolve the apparent contradictory literature proposals (see the SI for further discussion).^19^ Thus, sulfinate is proposed to act as a sulfinyl radical source and a catalyst.^7d^ The proline salt **3a-Na** reacts with the excited photocatalyst PC* to form alkyl radical **F**. Vinyl sulfone **E** reacts with the alkyl radical **F** to give **G**. SET with PC^•–^ gives anion **H** and regenerates PC. Protonation of **H** gives the final product **4**.

**Scheme 5:**
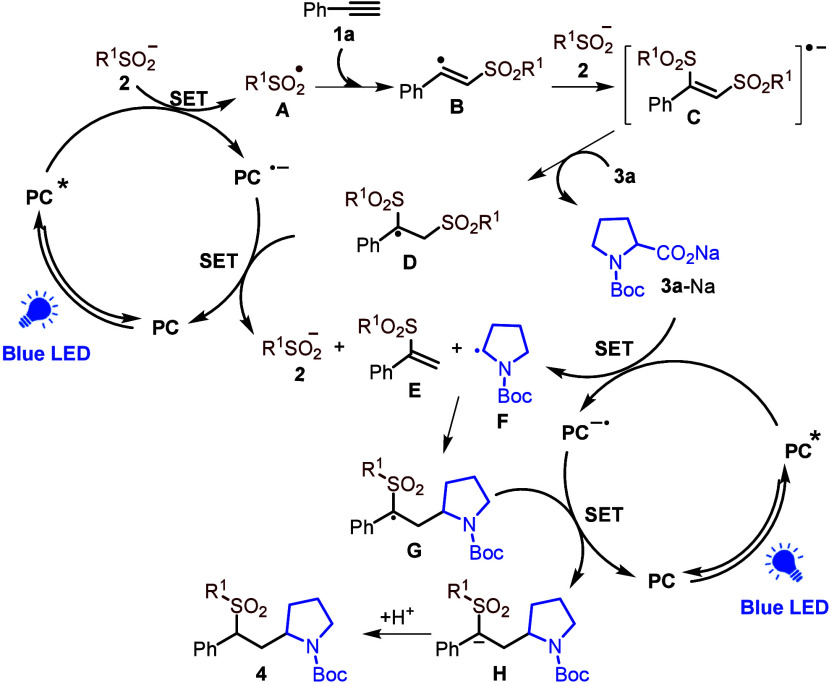
Plausible Mechanistic Proposal (PC = 4CzIPN)

The success of the 3-component reaction relies on the radicals **A** and **F** behaving differently. We speculate that slow generation of **3a-Na** (and thus **F**), as the byproduct of formation of **D**, may help to regulate the 3-component reaction; the electrophilic sulfinyl radical **A** would be expected to react preferentially with electron-rich **1a**, while nucleophilic alkyl radical **F** reacts preferentially with electron-poor **E**. In contrast, the reactions of **A** with **E**, and **F** with **1a** would be expected to be slow. Such considerations may enable the development of other 3-component reactions involving two different radicals reacting sequentially. Dual photoredox^20^ processes involving a single photocatalyst in two distinct cycles have great potential for rapid assembly of complex molecules.

In conclusion, we have demonstrated a robust carbosulfonylation of alkynes to give alkyl sulfones using readily available alkyl carboxylic acids and sodium sulfinates under photochemical conditions. This mild, metal-free sp-C to sp^3^-C reaction protocol has a broad substrate scope with high functional group tolerance. It enables the generation of novel functionalized alkyl sulfones in a single step from common feedstock chemicals. Furthermore, we synthesized two sedum alkaloids as an application of our methodology. We have proposed a plausible mechanism which is supported by quenching and control experiments. We anticipate the synthesis of these novel alkyl sulfones will enable further applications in synthetic chemistry.

## Data Availability

The data underlying this study are available in the published article and its online Supporting Information.

## Supporting Information Available

Experimental procedures and ^1^H and ^13^C NMR spectra for all new compounds (PDF)
FAIR data, including the primary NMR FID files, for compound(s) **4a**–**m**,**p**–**s**, **5a**–**o**,**6a**–**e**,**g**,**h**, **7a**–**d**, **8a**,**b**, **9**, **10**, **10f**, pyrrolsedamine, and pyrolallosedamine (ZIP)
