# Supplementary material for: Carbosulfonylation of Alkynes: A Direct Conversion of sp-C to sp3-C through Visible Light-Mediated 3-Component Reaction
Source: Org Lett. 2024 Sep 11;26(37):7858–63. doi: 10.1021/acs.orglett.4c02700 (PMC11421081; doi:10.1021/acs.orglett.4c02700)
Supplement: Supplementary file 1 — ol4c02700_si_001.pdf [file ol4c02700_si_001.pdf]

Electronic Supporting Information

Carbosulfonylation of alkynes: a direct conversion of sp-C to sp^3-C through visible light-mediated 3-component reaction

Mandapati Bhargava Reddy[a,b], Vanessa E. Becker[a] and Eoghan M. McGarrigle*[a,b]

[a]Centre for Synthesis & Chemical Biology, *UCD School of Chemistry, University College Dublin, Belfield, Dublin 4, Ireland*

[b]*A2P CDT in Sustainable Chemistry and BiOrbic Bioeconomy SFI Research Centre, University College Dublin, Belfield, Dublin 4, Ireland.*

email: eoghan.mcgarrigle@ucd.ie

S.No	Table of contents	Pages
1	General considerations	S2
2	General procedures	S3-S4
3	Intermediate trapping experiment	S5-S8
4	Stern-Volmer quenching studies	S9-S11
5	Mechanistic Considerations	S12-S13
6	Synthesis and characterisation data	S14-S49
7	References	S50
8	Copies of ^{1}H and ^{13}C NMR spectra	S51-S111

1. General considerations

Chemicals were purchased and used without further purification unless otherwise stated. The ^{1}H and ^{13}C NMR spectra were recorded in $CDCl_3$ on Varian VNMR, JEOL and Agilent DD2 spectrometers (400, 500 and 600 MHz for ^{1}H NMR; 101, 126 and 151 MHz for ^{13}C NMR) with TMS as an internal standard. Mass spectra were recorded on an Agilent-6546-QToF spectrometer. TLC was performed using Merck pre-coated TLC plates (Merck 60 F_{254}) and detected under UV light. Flash column chromatography (FCC) was performed using either silica gel [Davisil, 230-400 mesh (40-63 μm)] or using a Biotage Isolera® UV-VIS Flash Purification System Version 2.3.1 with Sfär Silica HC D (20 μm) prepacked silica cartridges. Structural assignments were made with additional information from gCOSY, gHSQC, and gHMBC experiments

FAIR Data is available as Supporting Information for Publication and includes the primary NMR FID files for compounds: **4a-m,p-s, 5a-o,6a-e,g,h,7a-d,8a,b,9,10,10f**, pyrrolsedamine and pyrolallosedamine.

Details of Light source:

Manufacturer: Kessil; Model: PR160L; Wavelength: 456 nm, Distance: 5 cm. Manufacturer gives spectral width as ~430-510 nm with radiant flux max at 456 nm of ~0.3 W/nm and average intensity of PR160 series' as 399 mW/cm^2 (measured from 1 cm distance), max power consumption 50W.

2. General procedures and spectroscopic data

2.1 General procedure A for carbosulfonylation

Alkyne (0.1 mmol, 1 equiv) was added to a crimptop vial followed by sodium arylsulfinate (0.2 mmol, 2 equiv), acid (0.2 mmol, 2 equiv) and 4CzIPN (1 mol%) and closed. The vial was evacuated and purged with N_2 three times. Anhydrous DMSO (2 mL) was added. The mixture was stirred under blue LED (456 nm) irradiation for 14 hours. Then, the reaction mixture was quenched with H_2O (20 mL) and the aqueous layer was extracted with ethyl acetate (3 x 10 mL). The combined organic layers were washed with brine solution (40 mL) and dried over Na_2SO_4. The solvent was removed under reduced pressure. The crude product was purified by flash column chromatography using EtOAc/pentane as an eluent to give the corresponding final product.

2.1 General procedure B for carbosulfonylation

Alkyne (0.2 mmol, 2 equiv) was added to a crimptop vial followed by sodium arylsulfinate (0.2 mmol, 2 equiv), acid (0.1 mmol, 1 equiv) and 4CzIPN (1 mol%) and closed. The vial was evacuated and purged with N_2 three times. Anhydrous DMSO (2 mL) was added. The mixture was stirred under blue LED (456 nm) irradiation for 14 hours. Then, the reaction mixture was quenched with H_2O (20 mL) and the aqueous layers were extracted with ethyl acetate (3 x 10 mL). The combined organic layer were washed with brine solution (40 mL) and dried over Na_2SO_4. The solvent was removed under reduced pressure. Trifluoroacetic acid (20 equiv.) and CH_2Cl_2 was added (0.1 M) to the crude product and the reaction was stirred overnight at rt. Following this the solvent was removed under reduced pressure. Et_2O was added (10 mL) and the organic layer was washed with H_2O (3 x 5 mL). The combined aqueous layers were basified with saturated NaOH solution to pH 10 and extracted with EtOAc (3 x 10 mL). The combined organic layers were dried over Na_2SO_4 and evaporated under reduced pressure to give the desired product.

For simple NMR spectra the Boc group was cleaved in cases with aliphatic alkynes as substrates and this procedure gave sufficiently pure compounds without the need for column chromatography.

2.2 Scale-up reaction

To a crimptop vial phenylacetylene (1 mmol, 1 equiv), Boc-L-proline (2 mmol, 2 equiv), sodium 4-methylbenzenesulfinate (2 mmol, 2 equiv) and 4CzIPN (1 mol%) were added. The

vial was sealed and evacuated and purged with N_2 three times. Anhydrous DMSO (6.7 mL) was added. The reaction was stirred under blue LED irradiation for 18 h. The reaction was quenched with H_2O (30 mL) and the aqueous layer was extracted with ethyl acetate (3 x 20 mL). The combined organic layers were washed with water (2 x 50 mL) and brine (1 x 50 mL) and dried over Na_2SO_4. The solvent was removed under reduced pressure. The product was purified by flash column chromatography to give the major diastereomer **4a** as a white solid (259 mg, 60%). The minor diastereomer **4a'** was isolated as a white solid (83.3 mg, 19%).

2.3 Unsuccessful substrates

We were unable to isolate the desired product from reactions using primary carboxylic acids: phenyl acetic acid, N-Boc glycine, and pentanoic acid. Either no desired product or complex mixtures were obtained.

2.4 Optimization table

Table S1: Optimization of carbosulfonylation reaction

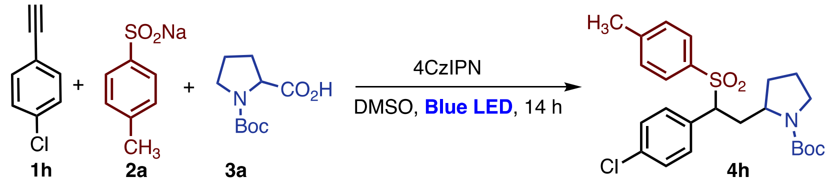

Entry	Stoichiometric Ratio (1h:2a:3a)	Solvent	Catalyst (mol %)	Yield (%)
1	1:2:2	DMSO	1	82
2	1:3:3	DMSO		83
3	1:1.5:1.5	DMSO	1	71
4	2:2:1	DMSO	1	46
5	2:1:2	DMSO	1	58
6	1:2:2	DMSO	0.5	73
7	1:2:2	EtOH	1	0
8	1:2:2	DCE	1	32
9	1:2:2	ACN:H_2O (9:1)	1	28

[a]**Reaction conditions: 1h** (0.1 mmol), **2a**, **3a** and catalyst (1 mol%) in 2 mL solvent were irradiated with blue LED (456 nm, 40W) in presence of N_2 atmosphere. [b]Yields and selectivity were determined by NMR with 1,3,5-trimethoxybenzene as an internal standard.

3. Intermediate trapping experiment

3.1 HRMS analysis of reaction mixture

General procedure A was followed except that TEMPO (0.5 mmol, 5 equiv) was added. **4a** was not observed. After completion of the reaction, the reaction mixture was directly analysed by HRMS and TEMPO adducts identified.

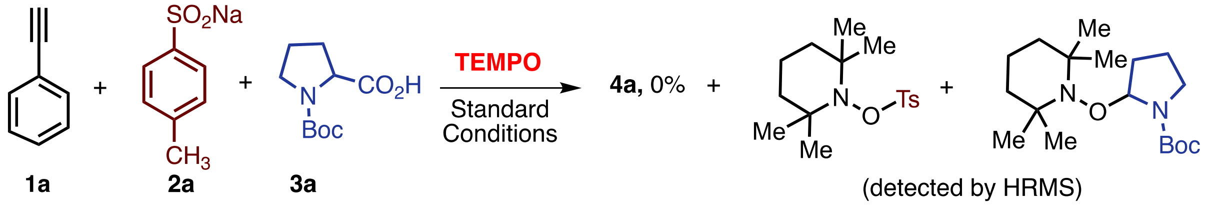

Custom Workflow Report

Agilent Trusted Answers

Sample Information

**Name**	20240116_EMB_VB_5-394	**Data File Path**	D:\MassHunter\Data\UCD_Data\Eoghan McGarrigle\Vanessa Becker\QTOF\20240116_EMB_VB_5-394_run2_isoMeOH.d
**Sample ID**		**Acq. Time (Local)**	16/01/2024 18:00:19 (UTC-08:00)
**Instrument**	Agilent_6546_QToF	**Method Path (Acq)**	D:\MassHunter\Methods\0_JM_POS_UV-MS_Poroshell50_MeOH-isocratic.m
**MS Type**	QTOF	**Version (Acq SW)**	6200 series TOF/6500 series Q-TOF 10.1 (48.0)
**Inj. Vol. (ul)**	2	**IRM Status**	All ions missed
**Position**	Vial 8	**Method Path (DA)**	D:\MassHunter\Methods\09_DA_METHODS\01_DA_z1-3.m
**Plate Pos.**		**Target Source Path**	C16H25NO3S,C18H34N2O3,C17H25NO
**Operator**		**Result Summary**	4 qualified (5 targets)

Compound Spectra

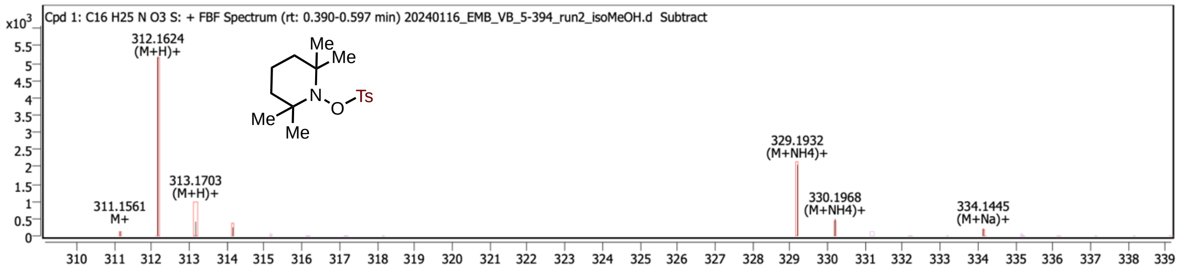

Compound Spectra

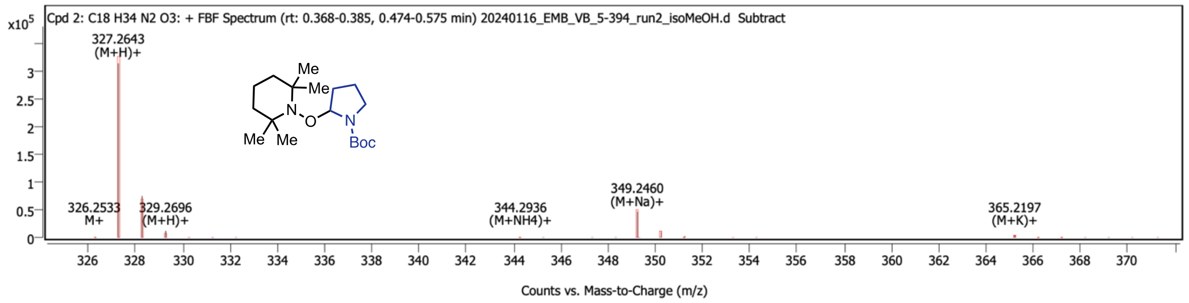

Fig. S1. HRMS spectrum.

The intermediate **10** (0.1 mmol, 1 equiv) was treated with **3a**-Na (0.2 mmol, 2 equiv) and TEMPO (0.4 mmol, 4 equiv) under standard conditions **4a** was not observed. After completion of the reaction, the reaction mixture was directly analysed by HRMS and TEMPO adduct of **10** identified.

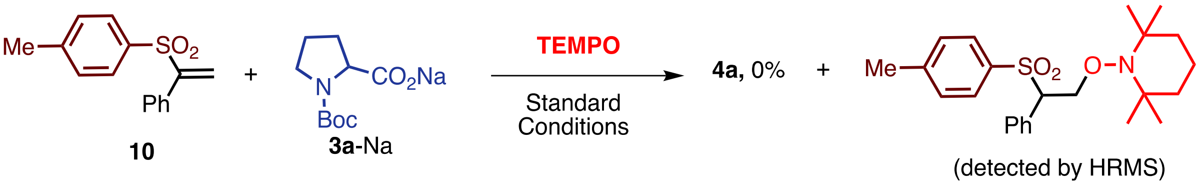

Custom Workflow Report

Agilent | Trusted Answers

Sample Information

**Name**	20240610_EMG_BR_4-85	**Data File Path**	D:\MassHunter\Data\UCD_Data\Eoghan McGarrigle\Bhargava Reddy\QTOF\20240610_EMG_BR_4-85.d
**Sample ID**		**Acq. Time (Local)**	11/06/2024 07:24:51 (UTC-07:00)
**Instrument**	Agilent_6546_QToF	**Method Path (Acq)**	D:\MassHunter\Methods\0_JM_POS_UV-MS_Poroshell50.m
**MS Type**	QTOF	**Version (Acq SW)**	6200 series TOF/6500 series Q-TOF 10.1 (48.0)
**Inj. Vol. (ul)**	0.5	**IRM Status**	Success
**Position**	Vial 93	**Method Path (DA)**	D:\MassHunter\Data\UCD_Data\Eoghan McGarrigle\Bhargava Reddy\QTOF\20240610_EMG_BR_4-85.d\AcqData\0_JM_POS_UV-MS_Poroshell50.m
**Plate Pos.**		**Target Source Path**	C24H33NO3S
**Operator**		**Result Summary**	1 qualified (1 targets)

Compound Details

Cpd. 1: C24 H33 N O3 S

Name	Formula	RT	RI	Mass	Score	Algorithm	Lib/DB
	C24 H33 N O3 S	5.462		415.2181	95.72	FBF	

Species	m/z	Score (Lib)	Num Spectra	Score (DB)	Score (MFG)	Score (RT)
M+ (M+H)+ (M+NH4)+ (M+Na)+ (M+K)+	415.2160 416.2254 433.2511 438.2071 454.1810					

Compound Spectra (overlaid)

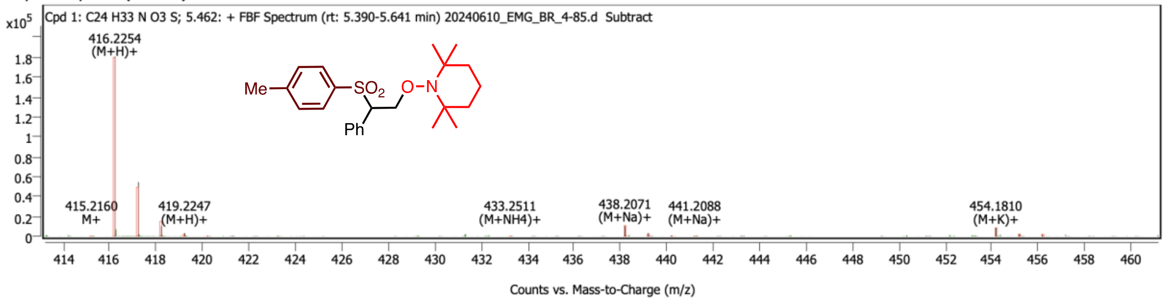

Fig. S2. HRMS spectrum of TEMPO adduct of **10**.

3.2 ^{1}H NMR analysis of reaction mixture *vs* time.

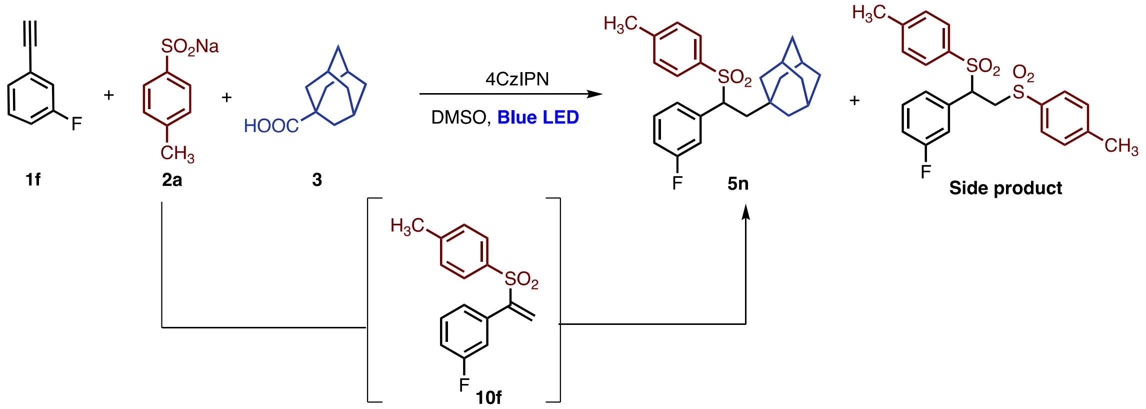

1-Ethynyl-3-fluorobenzene **1f** (0.025 mmol, 1 equiv) was added to NMR tube followed by *p*-toluenesulfinate **2a** (0.050 mmol, 2 equiv), adamantane-1-carboxylic acid **3** (0.050 mmol, 2 equiv), 4CzIPN (1 mol%) and 3,4,5-trimethoxybenzene as an internal standard. Anhydrous DMSO-d6 (0.6 mL) was added. The mixture was irradiated under blue LED (Distance: 5 cm). After 10 min irradiation, irradiation was stopped and the tube was wrapped in tin foil and taken to a spectrometer where a ^{1}H NMR spectrum was recorded. The tube was then resubjected to irradiation and this process was repeated after every 10 min of irradiation.

We observed the formation of **10f** (terminal -CH_2, two singlets at δ 6.54 and δ 6.32), conversion of **10f** into product **5n** (-SO_2CH, dd, δ 4.56). We also observed trace ammounts of a species tentatively assigned as a bissulfone side product (-SO_2CH, dd, δ 4.80).

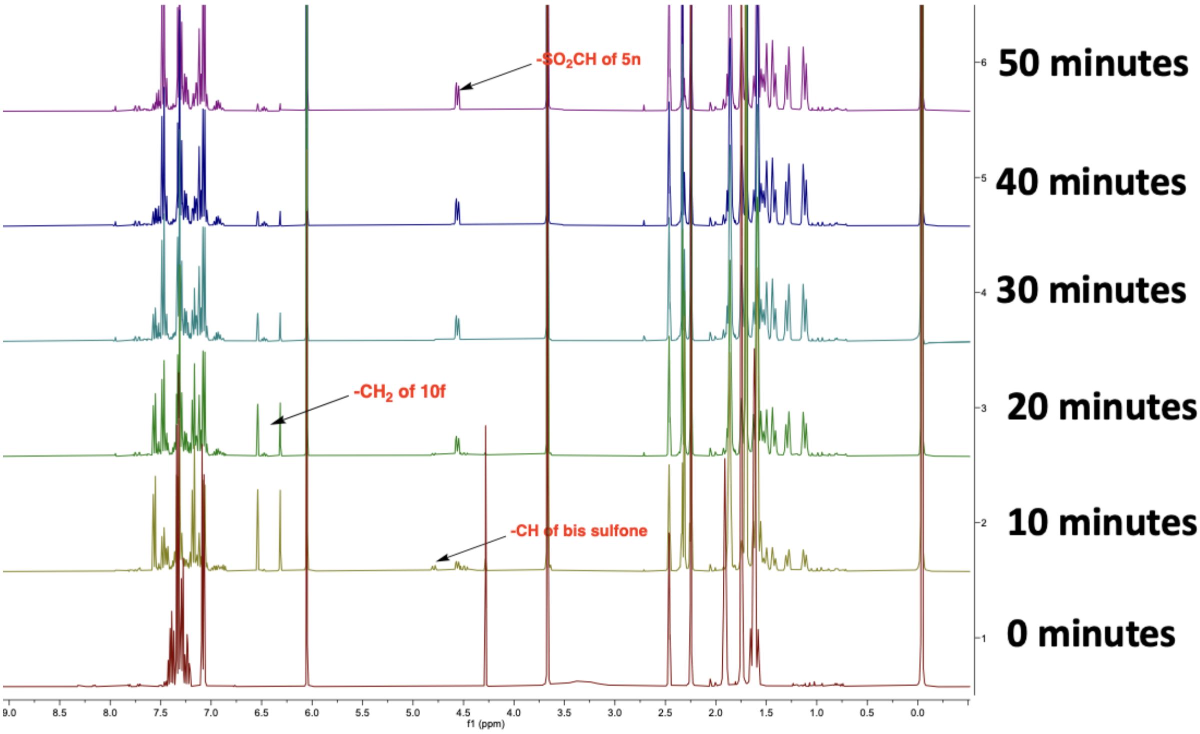

Fig. S3. ^{1}H NMR (400 MHz) spectrum of reaction mixture in DMSO-d6.

Table S2: Time course study of Starting material, Intermediate and Product

Time (Min)	Intermediate **10f** (%)	Product **5n** (%)	Alkyne **1a (%)**
0	0	0	100
10	49	25	4
20	28	50	2
30	13	61	0
40	7	66	0
50	3	68	0

[b]Yields and was determined by NMR with 1,3,5-trimethoxybenzene as an internal standard.

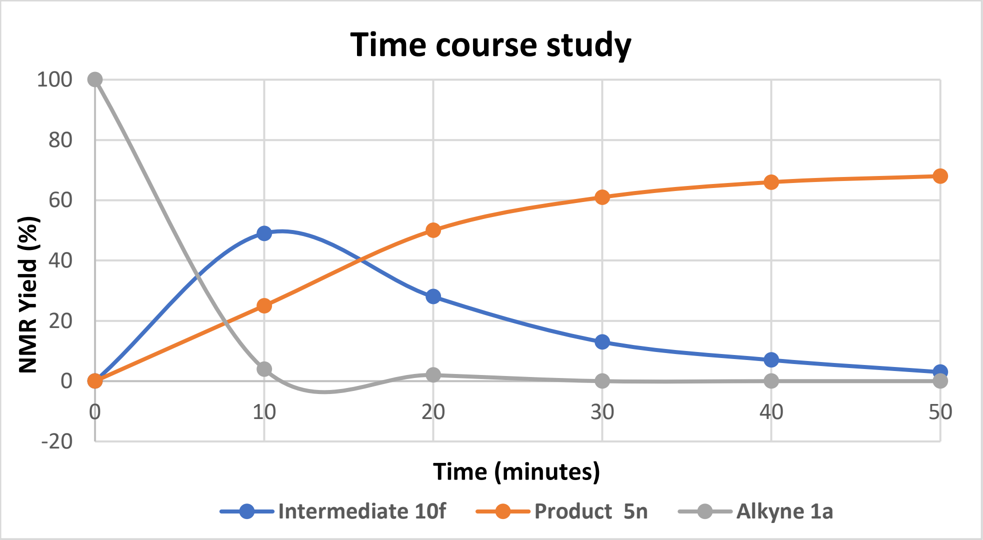

Fig. S4. Time course study graph (lines connecting data points are to guide the eye only).

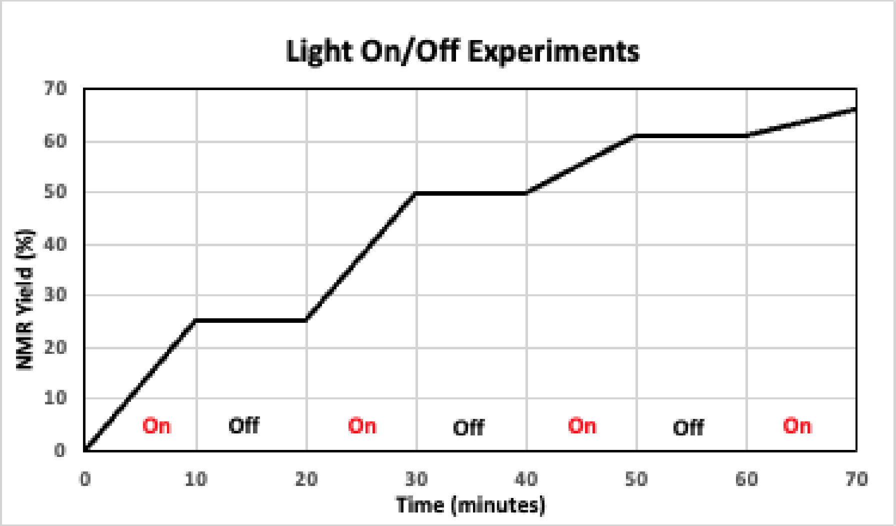

Fig. S5. Light on/off experiments over time.

4. Stern-Volmer quenching studies:

Fluorescence quenching experiments were carried out on a Agilent Technologies, Cary Eclipse Fluorescence Spectrofluorophotometer with a 4 mL quartz cuvette with a cap. The samples were prepared by mixing of 2 μM 4CzIPN with variable concentrations (0.0, 0.2, 0.4, 0.6 and 0.8 mM) of aryl sulfinate (**2a**), Boc-L-proline (**3a**) and mixtures of both in DMSO. The excitation wavelength was fixed at 365 nm, and the emission wavelength was measured at 537 nm.

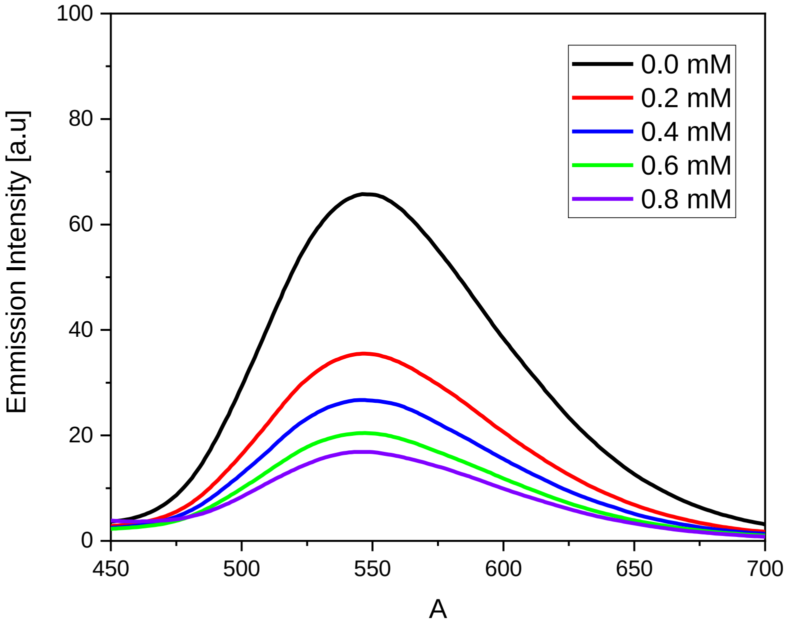

Fig. S6. Emission spectra of 4CzIPN (2 μM) at different concentrations of aryl sulfinate (**2a**).

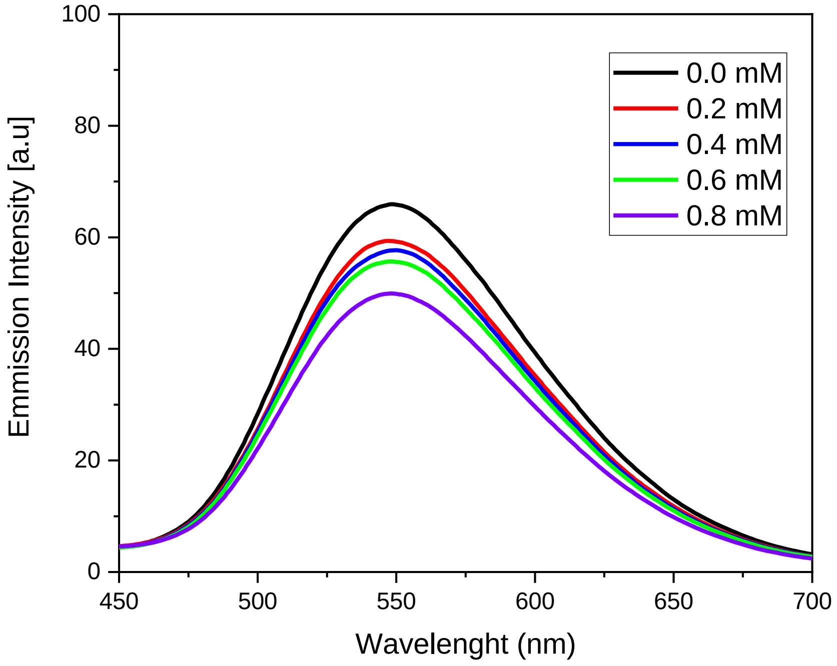

Fig. S7. Emission spectra of 4CzIPN (2 μM) at different concentrations of Boc-L-proline (**3a**).

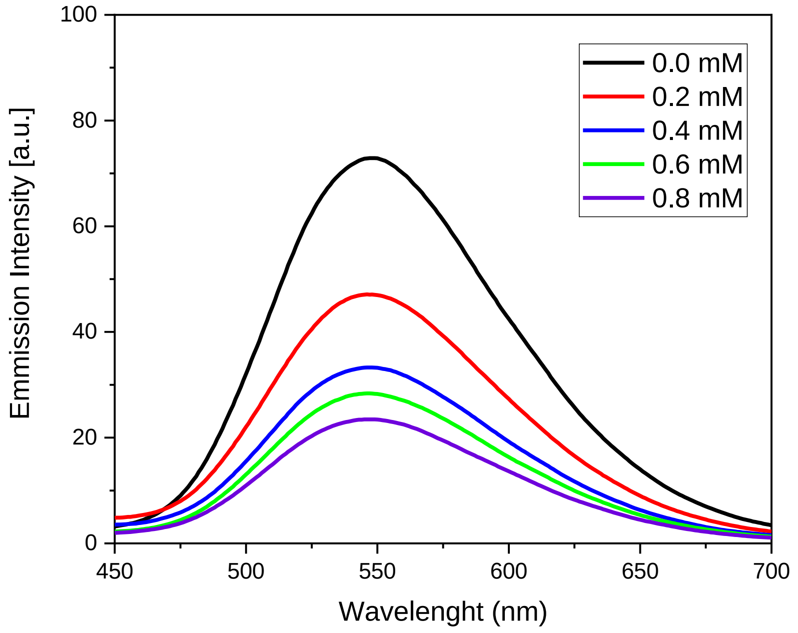

Fig. S8. Emission spectra of 4CzIPN (2 μM) at different concentrations of both aryl sulfinate (**2a**) + Boc-L-proline (**3a**).

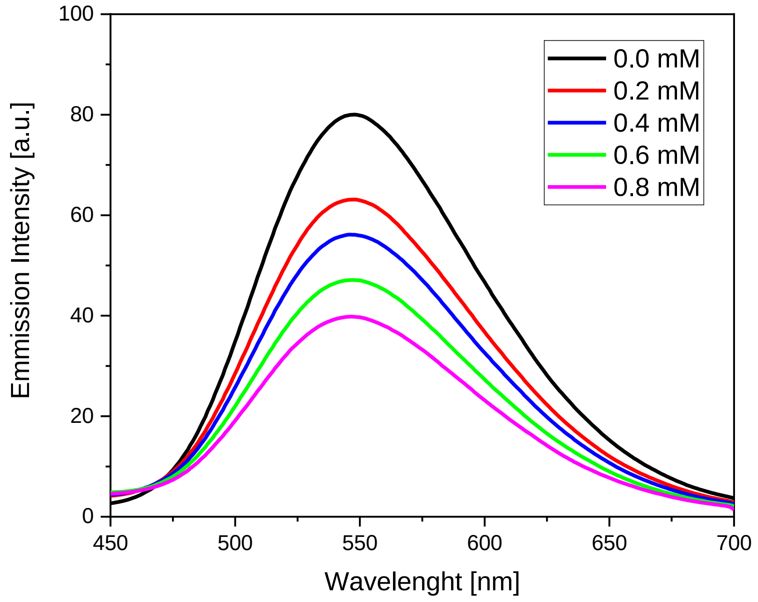

Fig. S9. Emission spectra of 4CzIPN (2 μM) at different concentrations of **3a-Na**.

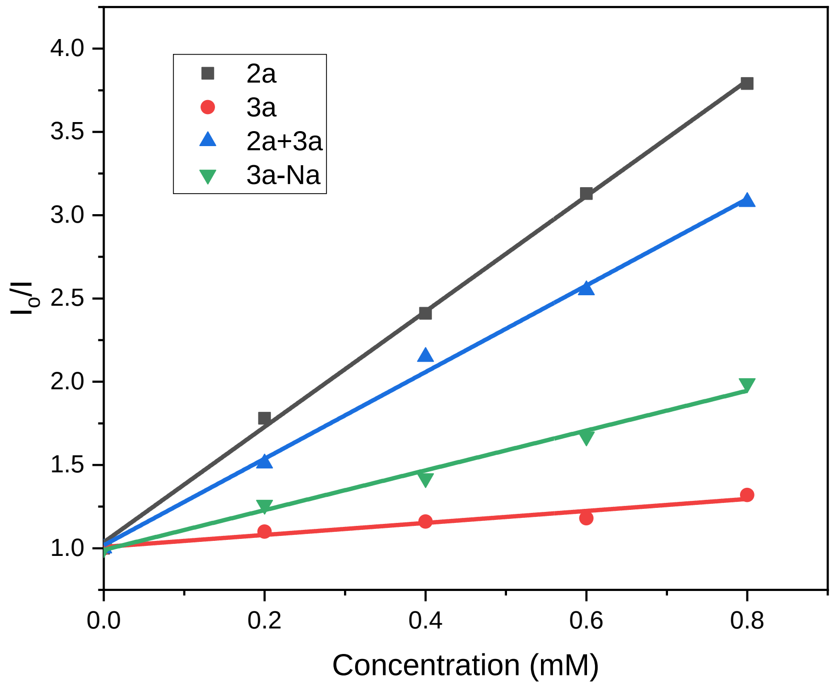

Fig. S10. Steady-state Stern-Volmer plot of 4CzIPN (2 μM) at different concentrations of aryl sulfinate (**2a**), Boc-L-proline (**3a**) and both.

5. Mechanistic Considerations

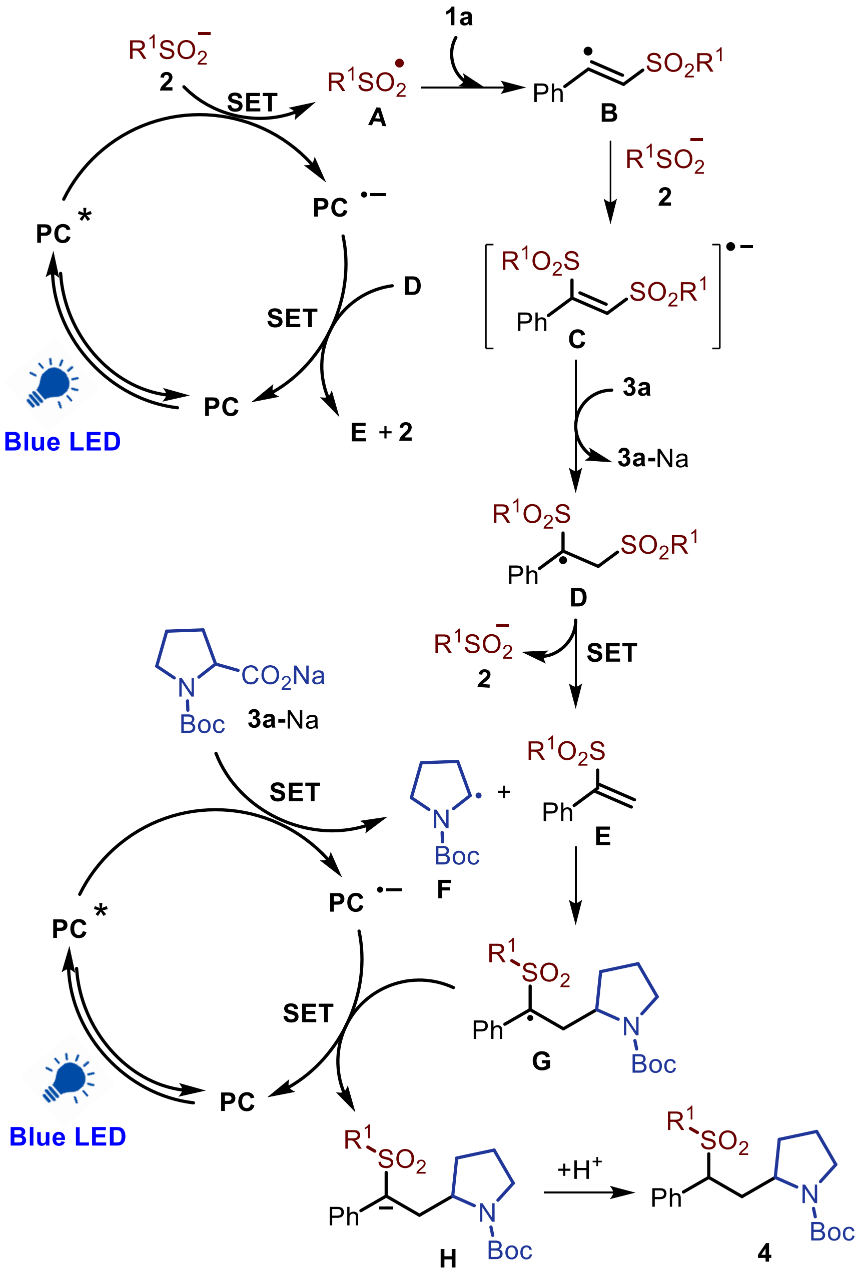

Lei and co-workers proposed previously[1] that when using Eosin Y as photocatalyst that Eosin Y reacts with sulfinate salt to give sulfinyl radical **A**. Then they proposed that SET from the $PC^{\bullet-}$ would generate a phenylacetylene-derived radical anion from **1a** which would react with sulfinyl radical **A** to give the anti-Markovnikov product after proton transfer.

In our case we used 4CzIPN and we noted that generation of a phenylacetylene radical anion doesn't seem consistent with the redox potentials of 4CzIPN or Eosin Y and phenylacetylene, therefore we felt an alternative explanation should be proposed: (a) Vega-Peñaloza, A.; Mateos, J.; Companyó, X.; Escudero-Casao, M.; Dell'Amico, L. A Rational Approach to Organo-Photocatalysis: Novel Designs and Structure-Property Relationships. *Angew. Chem., Int. Ed.*

2021, *60*, 1082-1097; (b) Luo, J.; Zhang, J. Donor-Acceptor Fluorophores for Visible-Light-Promoted Organic Synthesis: Photoredox/Ni Dual Catalytic C(sp3)-C(sp2) Cross-Coupling. *ACS Catal.* **2016**, *6*, 873-877; (c) Speckmeier, E.; Fischer, T. G.; Zeitler, K. A Toolbox Approach to Construct Broadly Applicable Metal-Free Catalysts for Photoredox Chemistry: Deliberate Tuning of Redox Potentials and Importance of Halogens in Donor-Acceptor Cyanoarenes. *J. Am. Chem. Soc.* **2018**, *140*, 15353-15365; (d) Seavill, P. W.; Holt, K. B.; Wilden, J. D.; Electrochemical Synthesis of Copper (I) Acetylides via Simultaneous Copper ion and Catalytic Base Electrogeneration for use in Click Chemistry. *RSC Adv.* **2019**, *9*, 29300-29304; e) Herbrik, F.; Camarero González, P.; Krstic, M.; Puglisi, A.; Benaglia, M.; Sanz, M. A.; Rossi, S. Eosin Y: Homogeneous Photocatalytic In-Flow Reactions and Solid-Supported Catalysts for In-Batch Synthetic Transformations. *Appl. Sci.* **2020**, *10*, 5596.

Furthermore, Lei's proposal is contrary to the regioselectivity observed by Sun, Nevado, and Rueping[2] in reactions involving sulfinyl radical additions to alkynes. In the case of Sun, an intramolecular reaction takes place wherein the first step is proposed to be addition of sulfinyl radical to an internal alkyne generating a vinyl radical stabilised by a phenyl group rather than an alkyl group. In the cases of Nevado and Rueping the sulfinyl radical is proposed to react directly with terminal alkynes to give vinyl radical **B** which is subsequently intercepted by a metal catalyst, and metal-catalysed cross-coupling then gives alkene products with the sulfonyl group at the terminal end. We note that two different methods were used in these papers for generating the sulfinyl radical.

Our proposal is consistent with those of Nevado, Rueping and Sun, and can also explain the experimental outcomes observed by Lei. Finally, we note, when the disulfone compound **11** was synthesized and treated with acid **3a** in the presence of $NaHCO_3$, the desired product **4a** was obtained in 20% yield (Scheme 4f), supporting our proposal of a bissulfonyl intermediate.

6. Synthesis and characterisation data

***tert*-Butyl 2-(2-phenyl-2-tosylethyl)pyrrolidine-1-carboxylate (4a):**

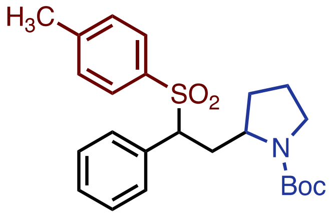

4a was synthesized following general procedure A (0.3 mmol); the d.r. was determined from the ^{1}H NMR spectrum of the crude mixture 4:1 (based on tolyl $-CH_3$ peaks integration).

Major diastereomer: Isolated yield of major diastereomer 66% (83 mg) (eluent: EtOAc/Pentane = 1:4), white solid.

^{1}H NMR (400 MHz, $CDCl_3$): δ 7.33 (d, *J* = 8.6 Hz, 2H), 7.28-7.24 (m, 1H), 7.20 (app t, *J* = 7.2 Hz, 2H), 7.17-7.09 (m, 4H), 4.19-3.89 (m, 1H), 3.72-3.09 (br. m, 3H), 2.82-2.59 (m, 1H), 2.56 (s, 3H), 2.23 (m, 1H), 1.84-1.60 (m, 4H), 1.40 (s, 9H).

^{13}C NMR (101 MHz, $CDCl_3$): δ 154.2, 144.5, 133.8, 131.4, 130.0, 129.2, 128.9, 128.9, 128.4, 79.7+79.0 (rotameric peaks), 69.2, 54.7+54.3 (rotameric peaks), 46.2, 31.1+30.8 (rotameric peaks), 29.8+29.7 (rotameric peaks), 28.5, 23.6+22.9 (rotameric peaks), 21.6.

HRMS: $[M+H]^+$ calculated for $C_{24}H_{32}NO_4S$: 430.2052; found: 430.2042.

Minor diastereomer: Isolated yield of minor diastereomer 16% (20 mg) (eluent: EtOAc/Pentane = 1:4), white solid.

^{1}H NMR (500 MHz, $CDCl_3$): δ 7.38 (d, *J* = 7.9 Hz, 2H), 7.27 (app d, *J* = 2.1 Hz, 1H), 7.25-7.19 (m, 3H), 7.15 (app d, *J* = 8.0 Hz, 3H), 4.46-4.05 (br. m, 1H), 3.84 (br. s, 1H), 3.49-3.12 (m, 2H), 2.93-2.58 (br. m, 1H), 2.38 (s, 3H), 2.25-2.20 (m, 1H), 1.73 (br. s, 4H), 1.44 (s, 9H)
^{13}C NMR (126 MHz, $CDCl_3$): δ 154.4 (br. s), 144.2, 134.5, 133.1, 130.3, 129.9, 129.2, 129.0, 128.3, 79.8+79.1 (rotameric peaks), 69.8+69.1 (rotameric peaks), 56.3+54.9 (rotameric peaks), 46.8+46.1 (rotameric peaks), 34.2+33.3 (rotameric peaks), 31.4, 28.5, 23.7+23.0 (rotameric peaks), 21.6.

HRMS: $[M+Na]^+$ calculated for $C_{24}H_{31}NO_4SNa$: 452.1871; found: 452.1866.

***tert*-Butyl 2-(2-(*p*-tolyl)-2-tosylethyl)pyrrolidine-1-carboxylate (4b):**

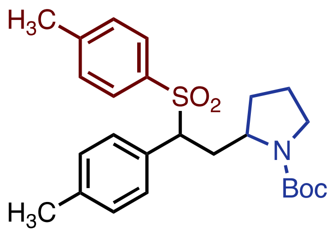

4b was synthesized following general procedure A; the d.r. was determined from the ^{1}H NMR spectrum of the crude mixture 4:1. Isolated yield of major diastereomer 64% (28 mg) (eluent: EtOAc/Pentane = 1:4), white solid.

^{1}H NMR (400 MHz, CDCl$_3$): δ 7.36 (d, *J* = 7.8 Hz, 2H), 7.17 (d, *J* = 7.8 Hz, 2H), 7.03 (app s, 4H), 4.16-3.89 (m, 1H), 3.70-3.10 (br. m, 3H), 2.81-2.57 (m, 1H), 2.39 (s, 3H), 2.31 (s, 3H), 2.31-2.13 (m, 1H), 1.81-1.57 (m, 4H), 1.42 (s, 9H).

^{13}C NMR (101 MHz, CDCl$_3$): δ 154.2, 144.5, 138.8, 133.9, 129.9, 129.3, 129.2, 129.1, 128.1, 79.7, 68.9, 54.3 (br. s), 46.2, 31.1 (br. s), 29.8 (br. s), 28.5, 22.9, 21.6, 21.2.

HRMS: $[M+H]^+$ calculated for $C_{25}H_{34}NO_4S$: 444.2208; found: 444.2202.

***tert*-Butyl 2-(2-(4-methoxyphenyl)-2-tosylethyl)pyrrolidine-1-carboxylate (4c):**

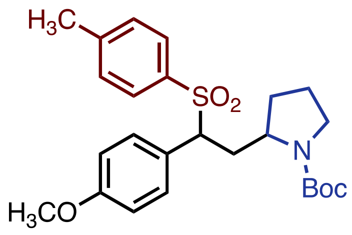

4c was synthesized following general procedure A; the d.r. was determined from the ^{1}H NMR spectrum of the crude mixture 4:1. Isolated yield of major diastereomer 58% (26 mg) (eluent: EtOAc/Pentane = 1:4), white solid.

^{1}H NMR (400 MHz, CDCl$_3$): δ 7.36 (d, *J* = 7.9 Hz, 2H), 7.17 (d, *J* = 8.2 Hz, 2H), 7.04 (d, *J* = 8.3 Hz, 2H), 6.75 (d, *J* = 8.5 Hz, 2H), 4.13-3.89 (m, 1H), 3.78 (s, 3H), 3.70-3.17 (br. m, 3H), 2.77-2.59 (m, 1H), 2.38 (s, 3H), 2.21-2.15 (m, 1H), 1.82-1.62 (m, 4H), 1.42 (s, 9H).

^{13}C NMR (101 MHz, CDCl$_3$): δ 160.0, 154.2, 144.4, 133.9, 131.2, 129.2, 129.2, 123.1, 113.8, 79.7, 68.5 (br. s), 55.2, 54.3 (br. s), 46.2, 31.1 (br. s), 29.8+29.7 (rotameric peaks), 28.5, 23.6+22.9 (rotameric peaks), 21.6.

HRMS: $[M+Na]^+$ calculated for $C_{25}H_{33}NO_5SNa$: 482.1977; found: 482.1970.

***tert*-Butyl 2-(2-(2-methoxyphenyl)-2-tosylethyl)pyrrolidine-1-carboxylate (4d):**

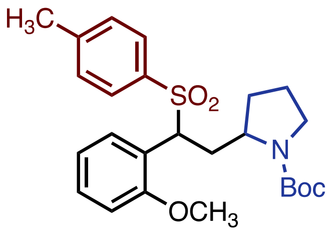

4d was synthesized following general procedure A; the d.r. was determined from the ^{1}H NMR spectrum of the crude mixture 1:1. Isolated yield of mixture of diastereomers 62% (28 mg) (eluent: EtOAc/Pentane = 1:4), white solid.

^{1}H NMR (500 MHz, CDCl$_3$): δ 7.67-7.44 (m, 2H), 7.35 (app d, J = 7.7 Hz, 4H), 7.26-7.18 (m, 2H), 7.09 (dd, J = 11.9, 7.6 Hz, 4H), 7.01-6.91 (m, 2H), 6.56, 6.54 (2xd, J = 8.3 Hz, 2H), 4.94-4.71 (m, 2H), 3.91 (dd, J = 7.7, 3.9 Hz, 1H), 3.55-3.41 (m, 2H), 3.35 (s, 3H), 3.32 (s, 3H), 3.26-3.16 (br. m, 3H), 2.93-2.86 (m, 1H), 2.73-2.71 (m, 1H), 2.35 (s, 3H), 2.33 (s, 3H), 2.28-2.08 (m, 2H), 1.90-1.62 (m, 8H), 1.47-1.34 (m, 18H).

^{13}C NMR (101 MHz, CDCl$_3$): δ 157.5, 154.3, 143.9, 143.8, 135.2, 134.9, 129.9, 129.8, 129.6, 129.4, 129.1, 128.7, 128.6, 120.6, 119.9, 110.2, 110.1, 79.6, 59.7, 59.2, 56.5, 55.2, 55.0, 54.5, 46.1 (br. s), 32.5, 31.0, 30.3, 29.9, 28.6, 28.4, 23.0, 22.9, 21.49, 21.47.

HRMS: $[M+Na]^+$ calculated for $C_{25}H_{33}NO_5SNa$: 482.1977; found: 482.1971.

***tert*-Butyl 2-(2-(3-aminophenyl)-2-tosylethyl)pyrrolidine-1-carboxylate (4e):**

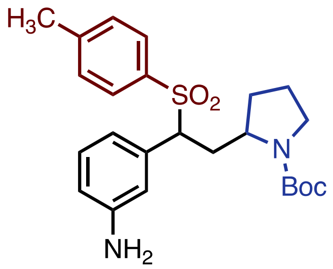

4e was synthesized following general procedure A; the d.r. was determined from the ^{1}H NMR spectrum of the crude mixture 3:1. Isolated yield of mixture of diastereomers 54% (25 mg), (eluent: EtOAc/Pentane = 2:3), yellow oil.

^{1}H NMR (400 MHz, CDCl$_3$): δ 7.41 (d, J = 8.9 Hz, 2H), 7.21-7.12 (m, 2H), 6.94 (t, J = 7.7 Hz, 1H), 6.64-6.51 (m, 2H), 6.39 (d, J = 7.6 Hz, 1H), 4.06-3.19 (br. m, 6H), 2.79-2.55 (m, 1H), 2.37 (s, 3H), 2.19-2.13 (m, 1H), 1.85-1.54 (m, 4H), 1.42 (app d, J = 7.1 Hz, 9H).

^{13}C NMR (101 MHz, CDCl$_3$): δ 154.3, 146.5, 144.4, 134.0, 132.4, 129.3, 129.1, 120.1, 116.3, 115.6, 79.6, 69.3, 54.4 (br. s), 46.2 (br. s), 31.3, 30.7, 29.9 (br. s), 28.5, 28.4, 23.6, 22.9, 21.58, 21.56.

HRMS: $[M+Na]^+$ calculated for $C_{24}H_{32}N_2O_4SNa$: 467.1980; found: 467.1974.

***tert*-Butyl 2-(2-(3-fluorophenyl)-2-tosylethyl)pyrrolidine-1-carboxylate (4f):**

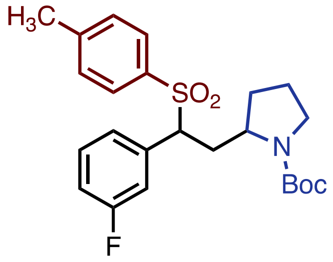

4f was synthesized following general procedure A; the d.r. was determined from the ^{1}H NMR spectrum of the crude mixture 4:1. Isolated yield of major diastereomer 67% (30 mg) (eluent: EtOAc/Pentane = 1:4), colourless viscous oil.

^{1}H NMR (400 MHz, CDCl$_3$): δ 7.38 (d, *J* = 8.0 Hz, 2H), 7.18 (app td, *J* = 7.9, 5.6 Hz, 3H), 7.06-6.84 (m, 3H), 4.26-3.92 (br. m, 1H), 3.79-3.07 (br. m, 3H), 2.69-2.61 (m, 1H), 2.38 (s, 3H), 2.29-2.22 (m, 1H), 1.81-1.59 (m, 4H), 1.41 (s, 9H).

^{13}C NMR (101 MHz, CDCl$_3$): δ 162.4 (d, *J* = 249.4 Hz), 154.2, 144.9, 134.0, 133.6, 129.9, 129.3, 129.1, 126.0, 116.7 (d, *J* = 22.9 Hz), 115.6 (d, *J* = 21.1 Hz), 79.9, 68.8, 54.9+54.2 (rotameric peaks), 46.2, 31.3, 29.8, 28.5, 23.6+22.9 (rotameric peaks), 21.6.

^{19}F NMR (376 MHz, CDCl$_3$): δ -112.3, -112.7 (rotameric peaks).

HRMS: $[M+Na]^+$ calculated for $C_{24}H_{30}FNO_4SNa$: 470.1777; found: 470.1773.

***tert*-Butyl 2-(2-(2-chlorophenyl)-2-tosylethyl)pyrrolidine-1-carboxylate (4g):**

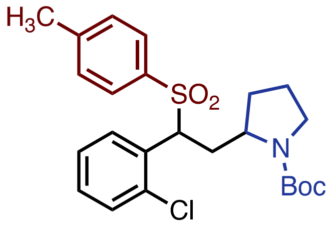

4g was synthesized following general procedure A; the d.r. was determined from the ^{1}H NMR spectrum of the crude mixture 1:1.

Diastereomer 1: Isolated yield of diastereomer 1, 33% (16 mg) (eluent: EtOAc/Pentane = 1:4), colourless viscous oil.

^{1}H NMR (500 MHz, CDCl$_3$): δ 7.84-7.66 (m, 1H), 7.42-7.36 (m, 2H), 7.33-7.25 (m, 1H), 7.20 (d, *J* = 7.0 Hz, 1H), 7.19-7.10 (m, 3H), 4.79 (br. s, 1H), 3.52-3.20 (br. m, 3H), 2.90-2.73 (m, 1H), 2.36 (s, 3H), 2.33-2.16 (m, 1H), 1.81-1.73 (m, 4H), 1.41 (s, 9H).

^{13}C NMR (126 MHz, CDCl$_3$): δ 154.2, 144.7, 135.7, 134.4, 130.4, 130.0, 129.7, 129.2, 129.0, 127.1, 79.7, 63.3 (br. s), 54.3, 46.2 (br. s), 31.3, 30.0, 28.5, 23.0, 21.6.

HRMS: $[M+Na]^+$ calculated for $C_{24}H_{30}ClNO_4SNa$: 486.1481 and 488.1455; found: 486.1476 and 488.1448.

Diastereomer 2: Isolated yield of diastereomer 2, 35% (17 mg) (eluent: EtOAc/Pentane = 1:4), colourless viscous oil.

^{1}H NMR (400 MHz, CDCl$_3$): δ 7.67 (s, 1H), 7.40 (d, J = 7.9 Hz, 2H), 7.30 (s, 1H), 7.23-7.10 (m, 4H), 4.86 (br. s, 1H), 3.90 (br. s, 1H), 3.34-3.17 (br. m, 2H), 2.99-2.81 (br. m, 1H), 2.37 (s, 3H), 2.28-2.00 (m, 1H), 1.90-1.57 (m, 4H), 1.50-1.32 (m, 9H).

^{13}C NMR (101 MHz, CDCl$_3$): δ 154.3, 144.5, 134.6, 131.5, 130.3, 129.7, 129.3, 129.3, 129.2, 129.0, 127.0, 79.7, 63.7 (br. s), 56.3 (br. s), 46.1 (br. s), 33.8, 31.1, 28.4, 23.1 (br. s), 21.6.

HRMS: [M+Na]$^+$ calculated for $C_{24}H_{30}ClNO_4SNa$: 486.1481 and 488.1455; found: 486.1475 and 488.1449.

***tert*-Butyl 2-(2-(4-chlorophenyl)-2-tosylethyl)pyrrolidine-1-carboxylate (4h):**

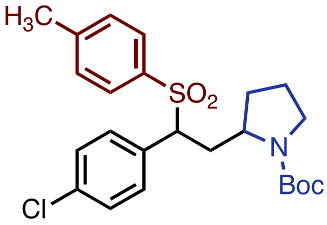

4h was synthesized following general procedure A (0.3 mmol); the d.r. was determined from the ^{1}H NMR spectrum of the crude mixture 4:1.

Major diastereomer: Isolated yield of major diastereomer 63% (86 mg) (eluent: EtOAc/Pentane = 1:4), white solid.

^{1}H NMR (400 MHz, CDCl$_3$): δ 7.46-7.31 (m, 2H), 7.29-7.14 (m, 4H), 7.14-6.98 (m, 2H), 4.24-3.90 (m, 1H), 3.80-3.09 (br. m, 3H), 2.74-2.53 (m, 1H), 2.38 (s, 3H), 2.31-2.13 (m, 1H), 1.90-1.54 (m, 4H), 1.40 (s, 9H).

^{13}C NMR (101 MHz, CDCl$_3$): δ 154.1 (br. s), 144.8 (br. s), 135.0+134.7 (rotameric peaks), 133.6, 131.3, 130.3+130.0 (rotameric peaks), 129.4, 129.2, 128.6, 79.8+79.1 (rotameric peaks), 68.6+68.4 (rotameric peaks), 54.7+54.2 (rotameric peaks), 46.2, 31.2+31.0 (rotameric peaks), 29.8, 28.5, 23.6+22.9 (rotameric peaks), 21.6.

HRMS: $[M+H]^+$ calculated for $C_{24}H_{31}ClNO_4S$: 464.1662 and 466.1636; found: 464.1654 and 466.1623.

Minor diastereomer: Isolated yield of minor diastereomer 15% (20 mg) (eluent: EtOAc/Pentane = 1:4), white solid.

^{1}H NMR (600 MHz, $CDCl_3$): δ 7.45-7.35 (m, 2H), 7.21-7.10 (m, 5H), 7.09-6.96 (m, 1H), 4.48-3.70 (br. m, 2H), 3.40-3.10 (br. m, 2H), 2.86-2.54 (m, 1H), 2.37 (s, 3H), 2.15-2.02 (m, 1H), 1.88-1.71 (m, 4H), 1.41 (s, 9H).

^{13}C NMR (151 MHz, $CDCl_3$): δ 155.1, 144.4, 134.6, 134.2, 131.6, 129.3, 128.9, 128.7, 128.4, 79.2, 68.2, 55.4+54.5 (rotameric peaks), 46.8+46.2 (rotameric peaks), 34.4, 31.5, 28.4, 23.7+23.0 (rotameric peaks), 21.6.

HRMS: $[M+Na]^+$ calculated for $C_{24}H_{30}ClNO_4SNa$: 486.1481 and 488.1455; found: 486.1476 and 488.1448.

***tert*-Butyl 2-(2-([1,1'-biphenyl]-4-yl)-2-tosylethyl)pyrrolidine-1-carboxylate (4i)**

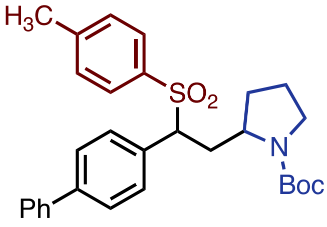

4i was synthesized following general procedure A; the d.r. was determined from the ^{1}H NMR spectrum of the crude mixture 4:1. Isolated yield of major diastereomer 53% (27 mg) (eluent: EtOAc/Pentane = 1:4), white solid.

^{1}H NMR (400 MHz, $CDCl_3$): δ 7.56 (d, J = 7.5 Hz, 2H), 7.48-7.37 (m, 7H), 7.26-7.17 (m, 4H), 4.29-3.93 (m, 1H), 3.83-3.12 (br. m, 3H), 2.89-2.61 (m, 1H), 2.39 (s, 3H), 2.33-2.13 (m, 1H), 1.90-1.59 (m, 4H), 1.43 (s, 9H).

^{13}C NMR (101 MHz, $CDCl_3$): δ 154.3, 144.6, 141.7, 140.2, 133.8, 130.5, 129.3, 129.3, 128.8, 127.7, 127.0, 127.0, 79.8+79.0 (rotameric peaks), 69.0, 54.8+54.3 (rotameric peaks), 46.2, 31.3+30.9 (rotameric peaks), 29.8, 28.6, 23.6+22.9 (rotameric peaks), 21.6.

HRMS: $[M+H]^+$ calculated for $C_{30}H_{36}NO_4S$: 506.2365; found: 506.2358.

***tert*-Butyl 2-(2-(4-cyanophenyl)-2-tosylethyl)pyrrolidine-1-carboxylate (4j):**

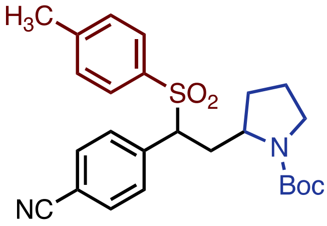

4j was synthesized following general procedure A; the d.r. was determined from the ^{1}H NMR spectrum of the crude mixture 4:1. Isolated yield of major diastereomer 43% (20 mg) (eluent: EtOAc/Pentane = 3:7), white solid.

^{1}H NMR (400 MHz, $CDCl_3$): δ 7.52 (d, J = 7.8 Hz, 2H), 7.34 (d, J = 8.1 Hz, 2H), 7.28 (d, J = 8.0 Hz, 2H), 7.19 (d, J = 8.5 Hz, 2H), 4.27, 4.02 (2xd, 11.9 Hz, 1H), 3.73-3.09 (br. m, 3H), 2.73-2.60 (m, 1H), 2.40 (s, 3H), 2.35-2.20 (m, 1H), 1.77-1.61 (m, 4H), 1.41, 1.37 (2xs, 9H).

^{13}C NMR (101 MHz, $CDCl_3$): δ 154.5, 145.1, 137.5+137.1 (rotameric peaks), 133.4, 132.0, 130.8, 129.5, 129.1, 118.4, 112.9+112.4 (rotameric peaks), 80.0, 69.1+68.9 (rotameric peaks), 54.9+54.2 (rotameric peaks), 46.3, 31.3, 29.9, 28.5, 23.6+22.8 (rotameric peaks), 21.6.

HRMS: $[M+Na]^+$ calculated for $C_{25}H_{30}N_2O_4SNa$: 477.1824; found: 477.1819.

***tert*-Butyl 2-(2-(4-(methoxycarbonyl)phenyl)-2-tosylethyl)pyrrolidine-1-carboxylate (4k):**

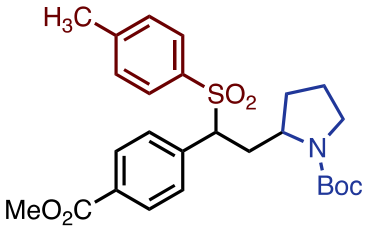

4k was synthesized following general procedure A; the d.r. was determined from the ^{1}H NMR spectrum of the crude mixture 4:1. Isolated yield of major diastereomer 49% (24 mg) (eluent: EtOAc/Pentane = 3:7), white solid.

^{1}H NMR (400 MHz, CDCl$_3$): δ 7.89 (d, *J* = 7.9 Hz, 2H), 7.34 (d, *J* = 7.8 Hz, 2H), 7.26-7.16 (m, 4H), 4.31-3.96 (m, 1H), 3.90 (s, 3H), 3.76-3.06 (br. m, 3H), 2.70 (td, *J* = 12.8, 4.4 Hz, 1H), 2.38 (s, 3H), 2.29-2.26 (m, 1H), 1.74-1.61 (m, 4H), 1.42 (s, 9H).

^{13}C NMR (101 MHz, CDCl$_3$): δ 166.5, 154.1, 144.9, 137.1+136.7 (rotameric peaks), 133.6, 130.6, 130.1, 129.5, 129.4, 129.2, 79.9+79.1 (rotameric peaks), 69.0, 54.9+54.2 (rotameric peaks), 52.2, 46.2, 31.3, 29.8, 28.5, 23.6+22.9 (rotameric peaks), 21.6.

HRMS: $[M+Na]^+$ calculated for $C_{26}H_{33}NO_6SNa$: 510.1926; found: 510.1922.

***tert*-Butyl 2-(2-tosyl-2-(4-(trifluoromethyl)phenyl)ethyl)pyrrolidine-1-carboxylate (4l):**

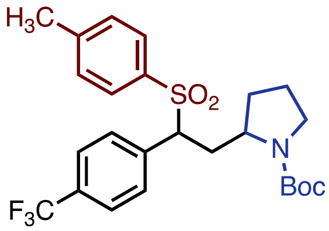

4l was synthesized following general procedure A; the d.r. was determined from the ^{1}H NMR spectrum of the crude mixture 4:1. Isolated yield of major diastereomer 48% (24 mg) (eluent: EtOAc/Pentane = 1:4), white solid.

^{1}H NMR (400 MHz, CDCl$_3$): δ 7.49 (d, *J* = 8.0 Hz, 2H), 7.35 (d, *J* = 9.4 Hz, 2H), 7.29 (d, *J* = 8.0 Hz, 2H), 7.21-7.15 (m, 2H), 4.26, 4.03 (2xd, 12.0 Hz, 1H), 3.76-3.03 (br. m, 3H), 2.71-2.65 (m, 1H), 2.40 (s, 3H), 2.28-2.24 (m, 1H), 1.61-1.80 (m, 4H), 1.41, 1.38 (2xs, 9H).
^{13}C NMR (101 MHz, CDCl$_3$): δ 154.1, 145.1, 135.8, 133.5, 130.4, 129.4, 129.1, 125.2, 122.5, 116.4, 79.9+79.1 (rotameric peaks), 68.9+68.8 (rotameric peaks), 54.8+54.2 (rotameric peaks), 46.3, 31.4+31.2 (rotameric peaks), 29.8, 28.5, 23.6+22.9 (rotameric peaks), 21.6.

^{19}F NMR (376 MHz, CDCl$_3$): δ -62.8.

HRMS: $[M+Na]^+$ calculated for $C_{25}H_{30}F_3NO_4SNa$: 520.1745; found: 520.1738.

***tert*-Butyl 2-(2-(3,5-bis(trifluoromethyl)phenyl)-2-tosylethyl)pyrrolidine-1-carboxylate (4m):**

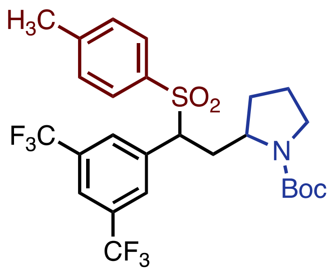

4m was synthesized following general procedure A; the d.r. was determined from the ^{1}H NMR spectrum of the crude mixture 4:1. Isolated yield of major diastereomer 35% (20 mg) (eluent: EtOAc/Pentane = 1:4), white solid.

^{1}H NMR (400 MHz, $CDCl_3$): δ 7.80-7.74 (m, 1H), 7.51 (s, 2H), 7.32 (d, J = 7.7 Hz, 2H), 7.26-7.14 (m, 2H), 4.47-4.10 (m, 1H), 3.91-3.04 (br. m, 3H), 2.74-2.30 (m, 5H) (2 aliphatic protons overlap with tolyl protons), 1.97-1.57 (m, 4H), 1.41-1.39 (m, 9H).

^{13}C NMR (101 MHz, $CDCl_3$): δ 154.6, 145.5 (br. s), 135.3, 132.9, 131.4 (q, J = 32.1 Hz), 130.3, 129.9, 129.6, 129.0, 122.5 (app q), 80.0+79.3 (rotameric peaks), 68.8, 55.5+54.4 (rotameric peaks), 46.3, 31.7 (br. s), 30.6+30.3 (rotameric peaks), 28.3, 23.6+22.9 (rotameric peaks), 21.5.

^{19}F NMR (376 MHz, $CDCl_3$): δ -62.9.

HRMS: $[M+Na]^+$ calculated for $C_{26}H_{29}F_6NO_4SNa$: 588.1619; found: 588.1615.

***tert*-Butyl 2-(2-(pyridin-3-yl)-2-tosylethyl)pyrrolidine-1-carboxylate (4p):**

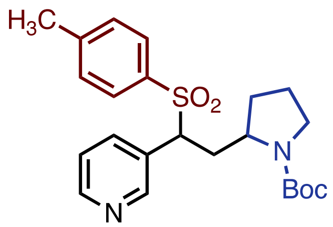

4p was synthesized following general procedure A; the d.r. was determined from the ^{1}H NMR spectrum of the crude mixture 3:1. Isolated yield of mixture of diastereomers 62% (27 mg) (eluent: EtOAc/Pentane = 2:1), colourless viscous oil.

^{1}H NMR (500 MHz, CDCl$_3$): δ 8.52 (d, *J* = 25.4 Hz, 1H), 8.08 (d, *J* = 25.8 Hz, 1H), 7.72 (dd, *J* = 26.2, 18.5 Hz, 1H), 7.27-7.24 (m, 1H), 7.44-7.31 (m, 2H), 7.18 (app t, *J* = 10.5 Hz, 2H), 4.28-3.95 (m, 1H), 3.75-3.14 (br. m, 3H), 2.67 (td, *J* = 13.0, 4.6 Hz, 1H), 2.38 (app d, *J* = 6.0 Hz, 3H), 2.30-2.25 (m, 1H), 1.81-1.60 (m, 4H), 1.44-1.37 (m, 9H).

^{13}C NMR (101 MHz, CDCl$_3$): δ 154.4, 151.3, 151.0, 150.2, 149.8, 145.0, 136.9, 133.4, 129.5, 129.0, 128.8, 128.3, 127.8, 123.4, 79.9, 79.2, 66.6, 54.9, 54.2, 46.2, 31.5, 31.0, 29.8, 28.5, 28.4, 23.6, 22.8, 21.6.

HRMS: $[M+H]^+$ calculated for $C_{23}H_{31}N_2O_4S$: 431.2004; found: 431.2003.

***tert*-Butyl 2-(2-(thiophen-3-yl)-2-tosylethyl)pyrrolidine-1-carboxylate (4q):**

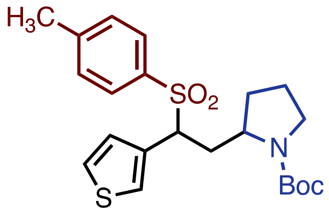

4q was synthesized following general procedure A; the d.r. was determined from the ^{1}H NMR spectrum of the crude mixture 4:1. Isolated yield of major diastereomer 45% (20 mg) (eluent: EtOAc/Pentane = 1:4), white solid.

^{1}H NMR (400 MHz, CDCl$_3$): δ 7.36 (d, *J* = 10.3 Hz, 2H), 7.26-7.11 (m, 3H), 7.11-6.91 (m, 2H), 4.20, 3.98 (2xd, 12.2 Hz, 1H), 3.83-3.14 (br. m, 3H), 2.67-2.53 (m, 1H), 2.38 (s, 3H), 2.23 (ddd, *J* = 13.4, 10.3, 3.2 Hz, 1H), 1.93-1.60 (m, 4H), 1.41 (s, 9H).

^{13}C NMR (101 MHz, CDCl$_3$): δ 154.3, 144.5, 133.6, 132.2, 129.1, 128.6+128.2 (rotameric peaks), 126.3+126.2 (rotameric peaks), 125.8+125.5 (rotameric peaks), 79.8+79.0 (rotameric peaks), 64.9+64.7 (rotameric peaks), 54.7+54.4 (rotameric peaks), 46.3, 31.8+31.3 (rotameric peaks), 29.9+29.6 (rotameric peaks), 28.5, 23.6+22.8 (rotameric peaks), 21.6.

HRMS: $[M+Na]^+$ calculated for $C_{22}H_{29}NO_4S_2Na$: 458.1435; found: 458.1428.

2-(2-Tosylpentyl)pyrrolidine (4r)

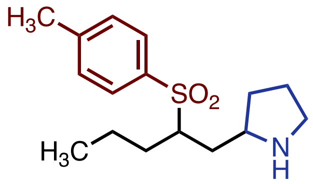

4r was synthesized following general procedure B; the d.r. was determined from the ^{1}H NMR spectrum of the crude mixture 1:1 (based on tolyl -CH_3 peaks integration, before -Boc deprotection). Isolated yield of mixture of diastereomers 59% (17 mg), yellow oil.

^{1}H NMR (400 MHz, $CDCl_3$): δ 7.80-7.66 (m, 2H), 7.37-7.28 (m, 2H), 3.37-3.06 (m, 2H), 3.01-2.79 (m, 2H), 2.43 (s, 3H), 2.37-2.23 (m, 1H), 2.00-1.81 (m, 2H), 1.80-1.60 (m, 4H), 1.52-1.38 (m, 2H), 1.38 -1.27 (m, 1H), 1.27- 1.14 (m, 1H), 0.85-0.81 (m, 3H).

^{13}C NMR (101 MHz, $CDCl_3$): δ 144.4, 134.93, 134.86, 129.7, 128.87, 128.86, 62.6, 56.7, 46.4, 35.1, 34.2, 31.8, 31.6, 31.0, 25.3, 21.6, 19.93, 19.90, 14.0, 13.9.

HRMS: $[M+H]^+$ calculated for $C_{16}H_{26}NO_2S$: 296.1684; found: 296.1681.

2-(2-Tosylhexyl)pyrrolidine (4s)

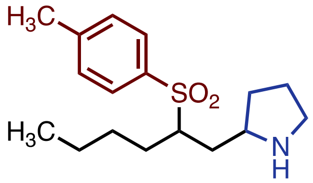

4r was synthesized following general procedure B; the d.r. was determined from the ^{1}H NMR spectrum of the crude mixture 1:1 (based on tolyl -CH_3 peaks integration, before -Boc deprotection). Isolated yield of mixture of diastereomers 65% (20 mg), yellow oil.

^{1}H NMR (400 MHz, $CDCl_3$): δ 7.83-7.69 (m, 2H), 7.34 (d, J = 8.0 Hz, 2H), 5.53 (br. s, 1H), 3.58-3.01 (m, 1H), 3.25-2.97 (m, 3H), 2.44 (s, 3H), 2.17-1.98 (m, 2H), 1.93-1.62 (m, 4H), 1.49-1.33 (m, 3H), 1.31-1.12 (m, 3H), 0.80 (app dt, J = 9.4, 7.1 Hz, 3H).

^{13}C NMR (101 MHz, CDCl$_3$): δ 144.8, 144.7, 134.6, 134.1, 129.8, 129.7, 129.0, 128.9, 62.5, 62.1, 57.7, 57.4, 45.7, 45.6, 33.1, 32.3, 31.5, 31.3, 29.2, 29.0, 28.4, 28.3, 24.5, 24.4, 22.4, 21.6, 13.6, 13.5.

HRMS: $[M+H]^+$ calculated for $C_{17}H_{28}NO_2S$: 310.1840; found: 310.1839.

Benzyl 2-(2-phenyl-2-tosylethyl)pyrrolidine-1-carboxylate (5a):

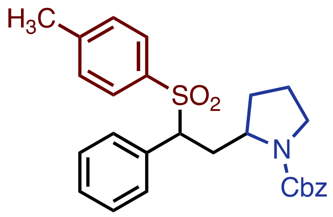

5a was synthesized following general procedure A; the d.r. was determined from the ^{1}H NMR spectrum of the crude mixture 4:1. Isolated yield of major diastereomer 64% (29 mg) (eluent: EtOAc/Pentane = 1:4), white solid.

^{1}H NMR (400 MHz, CDCl$_3$): δ 7.45-7.07 (m, 12H), 6.95 (t, J = 7.6 Hz, 1H), 6.87 (d, J = 7.7 Hz, 1H), 5.08-5.00 (m, 2H), 4.19-3.89 (m, 1H), 3.78-3.22 (br. m, 3H), 2.81-2.62 (m, 1H), 2.39 (app d, J = 7.2 Hz, 3H), 2.36-2.19 (m, 1H), 1.97-1.60 (m, 4H).

^{13}C NMR (101 MHz, CDCl$_3$): δ 154.7, 144.5, 136.8+136.4 (rotameric peaks), 133.7, 131.4+131.0 (rotameric peaks), 130.1+129.9 (rotameric peaks), 129.2, 128.8, 128.7, 128.6+128.4 (rotameric peaks), 128.3, 127.9+127.6 (rotameric peaks), 69.35+69.32 (rotameric peaks), 67.3+66.5 (rotameric peaks), 55.4+54.5 (rotameric peaks), 46.6+46.1 (rotameric peaks), 31.3+30.9 (rotameric peaks), 29.9+29.6 (rotameric peaks), 23.6+23.0 (rotameric peaks), 21.6.

HRMS: $[M+H]^+$ calculated for $C_{27}H_{30}NO_4S$: 464.1895; found: 464.1892.

1-Benzoyl-2-(2-phenyl-2-tosylethyl)pyrrolidine (5b):

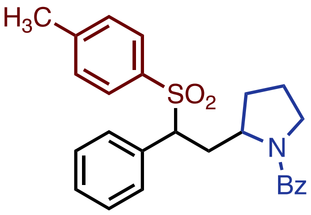

5b was synthesized following general procedure A; the d.r. was determined from the ^{1}H NMR spectrum of the crude mixture 4:1. Isolated yield of major diastereomer 68% (28 mg) (eluent: EtOAc/Pentane = 1:4), white solid.

^{1}H NMR (400 MHz, $CDCl_3$): δ 7.33-7.20 (m, 12H), 7.12 (d, *J* = 8.1 Hz, 2H), 4.29-4.06 (m, 2H), 3.42-3.24 (m, 2H), 2.94 (td, *J* = 12.7, 5.2 Hz, 1H), 2.47-2.36 (m, 1H), 2.35 (s, 3H), 2.00-1.86 (m, 2H), 1.75-1.60 (m, 2H).

^{13}C NMR (101 MHz, $CDCl_3$): δ 170.2, 144.5, 136.7, 133.4, 131.5, 130.1, 130.0, 129.3, 129.1, 128.8, 128.4, 128.1, 127.3, 69.4, 55.1, 49.8, 31.2, 30.2, 25.0, 21.6.

HRMS: $[M+H]^+$ calculated for $C_{26}H_{28}NO_3S$: 434.1789; found: 434.1787.

***tert*-Butyl 2-(2-phenyl-2-tosylethyl)azetidine-1-carboxylate (5c):**

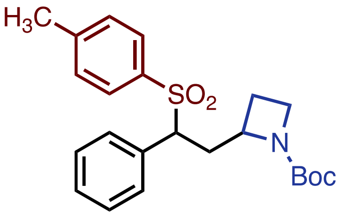

5c was synthesized following general procedure A; the d.r. was unable to be determined from the NMR spectrum of the crude mixture. Isolated yield of major diastereomer 41% (17 mg) (eluent: EtOAc/Pentane = 1:4), white solid.

^{1}H NMR (400 MHz, $CDCl_3$): δ 7.37 (d, *J* = 8.3 Hz, 2H), 7.28 (app dd, *J* = 7.6, 5.2 Hz, 1H), 7.25-7.20 (m, 2H), 7.16 (d, *J* = 8.0 Hz, 2H), 7.10 (d, *J* = 6.9 Hz, 2H), 4.00 (br. s, 2H), 3.74 (t, *J* = 7.6 Hz, 2H), 2.78 (ddd, *J* = 13.5, 11.4, 4.1 Hz, 1H), 2.66 (td, *J* = 9.7, 4.8 Hz, 1H), 2.38 (s, 3H), 2.10 (br. s, 1H), 1.79 (br. s, 1H), 1.42 (s, 9H).

^{13}C NMR (101 MHz, CDCl$_3$): δ 156.3, 144.5, 133.9, 132.1, 129.8, 129.2+129.1 (rotameric peaks), 128.9+128.8 (rotameric peaks), 128.6, 128.5, 79.6, 67.6, 59.2, 45.9 (br. s), 33.1 (br. s), 28.4, 25.6, 21.6.

HRMS: $[M+Na]^+$ calculated for $C_{23}H_{29}NO_4SNa$: 438.1715; found: 438.1709.

***tert*-Butyl 2-(2-phenyl-2-tosylethyl)piperidine-1-carboxylate (5d):**

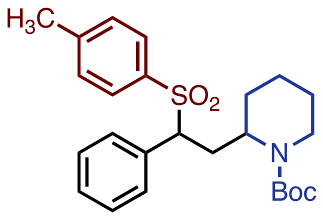

5d was synthesized following general procedure A; the d.r. was determined from the ^{1}H NMR spectrum of the crude mixture 4:1. Isolated yield of major diastereomer 65% (28 mg) (eluent: EtOAc/Pentane = 1:4), white solid.

^{1}H NMR (400 MHz, CDCl$_3$): δ 7.32-7.27 (m, 2H), 7.23-7.17 (m, 1H), 7.14 (t, J = 7.2 Hz, 2H), 7.11-7.02 (m, 4H), 4.14 (br. s, 1H), 4.08-3.32 (m, 3H), 2.67 (br. s, 1H), 2.46 (br. s, 1H), 2.30 (s, 3H), 1.66-1.36 (m, 6H), 1.30 (s, 9H).
^{13}C NMR (101 MHz, CDCl$_3$): δ 154.5, 144.4, 134.0, 132.1, 130.1, 129.8, 129.2, 129.0, 128.6, 128.3, 79.3, 69.2, 48.9, 38.8 (br. s), 29.6, 28.4, 26.9, 25.2, 21.5, 19.0+18.7 (rotameric peaks).

HRMS: $[M+H]^+$ calculated for $C_{25}H_{33}NO_4SNa$: 466.2028; found: 466.2023.

***tert*-Butyl 3-(2-phenyl-2-tosylethyl)morpholine-4-carboxylate (5e):**

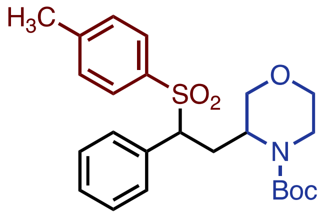

5e was synthesized following general procedure A; the d.r. was unable to be determined from the NMR spectrum of the crude mixture. Isolated yield of major diastereomer 32% (14 mg) (eluent: EtOAc/Pentane = 1:2), white solid.

^{1}H NMR (400 MHz, CDCl$_3$): δ 7.33 (d, *J* = 8.0 Hz, 2H), 7.26-7.24 (m, 1H), 7.20 (t, *J* = 7.1 Hz, 2H), 7.13 (d, *J* = 8.0 Hz, 2H), 7.07 (d, *J* = 7.0 Hz, 2H), 4.11 (d, *J* = 7.0 Hz, 1H), 3.86-3.85 (m, 3H), 3.39-3.31 (m, 2H), 2.86 (br. s, 2H), 2.51-2.22 (m, 5H), 1.40 (s, 9H).

^{13}C NMR (101 MHz, CDCl$_3$): δ 154.2, 144.5, 134.0, 132.0, 129.8, 129.2, 129.1, 128.8, 128.4, 80.2, 68.7, 66.9, 49.6, 39.2, 28.4, 25.9, 21.6.

HRMS: $[M+Na]^+$ calculated for $C_{24}H_{31}NO_5SNa$: 468.1820; found: 468.1812.

***tert*-Butyl (4-phenyl-4-tosylbutan-2-yl)carbamate (5f):**

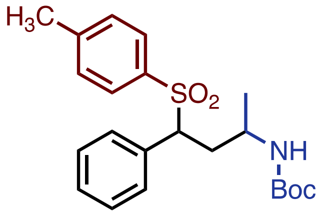

5f was synthesized following general procedure A; the d.r. was determined after purification 1.2:1.

Diastereomer 1: Isolated yield of diastereomer 1, 21% (8.5 mg) (eluent: EtOAc/Pentane = 1:4), colourless viscous oil.

^{1}H NMR (400 MHz, CDCl$_3$): δ 7.36 (d, *J* = 8.0 Hz, 2H), 7.28-7.19 (m, 3H), 7.14 (d, *J* = 8.0 Hz, 2H), 7.12-7.07 (m, 2H), 4.42-4.06 (m, 2H), 3.47 (br. s, 1H), 2.50-2.25 (m, 5H), 1.49-1.23 (m, 9H), 1.12 (d, *J* = 6.6 Hz, 3H).

^{13}C NMR (101 MHz, CDCl$_3$): δ 155.1, 144.4, 134.3, 132.3, 130.0, 129.2, 128.9, 128.8, 128.4, 79.2, 68.9, 44.0, 35.6, 28.3, 22.3, 21.6.

HRMS: $[M+Na]^+$ calculated for $C_{22}H_{29}NO_4SNa$: 426.1715; found: 426.1709.

Diastereomer 2: Isolated yield of diastereomer 2, 25% (10 mg) (eluent: EtOAc/Pentane = 1:4), colourless viscous oil.

^{1}H NMR (400 MHz, CDCl$_3$): δ 7.39-7.31 (m, 2H), 7.28-7.18 (m, 3H), 7.14 (d, *J* = 8.0 Hz, 2H), 7.09 (app dt, *J* = 6.8, 1.5 Hz, 2H), 4.29-4.04 (m, 2H), 3.62 (s, 1H), 2.42 (t, *J* = 7.3 Hz, 2H), 2.37 (s, 3H), 1.36 (s, 9H), 1.06 (d, *J* = 6.6 Hz, 3H).

^{13}C NMR (101 MHz, CDCl$_3$): δ 154.8, 144.5, 134.0, 132.2, 129.9, 129.2, 129.1, 128.7, 128.5, 79.2, 69.3, 45.1, 34.6, 28.3, 21.6, 20.4.

HRMS: $[M+Na]^+$ calculated for $C_{22}H_{29}NO_4SNa$: 426.1715; found: 426.1711.

***tert*-Butyl (4-methyl-1-phenyl-1-tosylpentan-3-yl)carbamate (5g):**

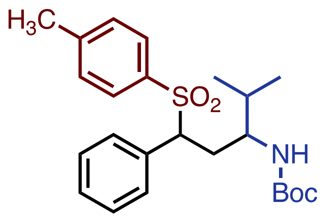

5g was synthesized following general procedure A; the d.r. was determined after purification 1.2:1.

Diastereomer 1: Isolated yield of major diastereomer 19% (8 mg) (eluent: EtOAc/Pentane = 1:4), white solid.

^{1}H NMR (500 MHz, CDCl$_3$): δ 7.36 (d, *J* = 8.0 Hz, 2H), 7.26-7.23 (m, 1H), 7.20 (t, *J* = 7.2 Hz, 2H), 7.14 (d, *J* = 7.9 Hz, 2H), 7.08 (d, *J* = 6.6 Hz, 2H), 4.13 (dd, *J* = 9.4, 3.4 Hz, 1H), 3.97 (d, *J* = 10.0 Hz, 1H), 3.60 (dt, *J* = 9.7, 4.9 Hz, 1H), 2.66 (dt, *J* = 14.6, 4.3 Hz, 1H), 2.37 (s, 3H), 2.21-2.11 (m, 1H), 1.74-1.60 (m, 1H), 1.41, 1.30 (2xs, 9H), 0.85 (app dd, *J* = 6.8, 2.5 Hz, 6H).

^{13}C NMR (126 MHz, CDCl$_3$): δ 155.4, 144.4, 134.2, 133.4, 130.0, 129.7, 129.2, 129.0, 128.4, 78.9, 69.7, 54.8, 31.8, 31.2, 28.3, 21.6, 19.2, 16.8.

HRMS: $[M+Na]^+$ calculated for $C_{24}H_{33}NO_4SNa$: 454.2028; found: 454.2021.

Diastereomer 2: Isolated yield of major diastereomer 24% (10 mg) (eluent: EtOAc/Pentane = 1:4), colourless viscous oil.

^{1}H NMR (500 MHz, CDCl$_3$): δ 7.37-7.33 (m, 2H), 7.25 (d, *J* = 7.9 Hz, 1H), 7.20 (t, *J* = 7.4 Hz, 2H), 7.14 (app dd, *J* = 8.0, 5.3 Hz, 2H), 7.08 (d, *J* = 7.5 Hz, 2H), 4.38 (d, *J* = 10.2 Hz, 1H), 4.15-4.08 (m, 1H), 3.22-2.98 (m, 1H), 2.37 (s, 3H), 2.34-2.31 (m, 2H), 1.77-1.63 (m, 1H), 1.41, 1.90 (2xs, 9H), 0.86-0.82 (m, 6H).

^{13}C NMR (126 MHz, CDCl$_3$): δ 155.5, 144.3, 134.41, 130.0, 129.7, 129.2, 129.0, 128.9, 128.4, 79.1, 69.2, 52.7, 33.0, 30.9, 28.3, 28.28, 21.6, 18.7, 17.8.

HRMS: $[M+Na]^+$ calculated for $C_{24}H_{33}NO_4SNa$: 454.2028; found: 454.2023.

***tert*-Butyl (1,4-diphenyl-4-tosylbutan-2-yl)carbamate (5h):**

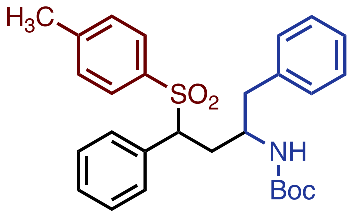

5h was synthesized following general procedure A; the d.r. was unable to be determined from the NMR spectrum of the crude mixture. Isolated yield of major diastereomer 26% (12 mg), (eluent: EtOAc/Pentane = 1:4), yellow oil.

^{1}H NMR (400 MHz, CDCl$_3$): δ 7.32 (d, *J* = 8.0 Hz, 2H), 7.29-7.17 (m, 6H), 7.13 (d, *J* = 8.1 Hz, 2H), 7.08-7.02 (m, 4H), 4.14 (app dd, *J* = 9.3, 4.3 Hz, 2H), 3.88 (app q, *J* = 7.6 Hz, 1H), 2.74 (d, *J* = 6.3 Hz, 2H), 2.60 (ddd, *J* = 14.3, 6.3, 4.4 Hz, 1H), 2.38 (s, 3H), 2.26 (dt, *J* = 15.2, 9.1 Hz, 1H), 1.29 (s, 9H).

^{13}C NMR (101 MHz, CDCl$_3$): δ 154.9, 144.4, 137.2, 134.0, 132.8, 129.8, 129.4, 129.23, 129.18, 129.1, 128.7, 128.5, 126.5, 79.2, 69.2, 50.6, 40.8, 33.0, 28.2, 21.6.

HRMS: $[M+Na]^+$ calculated for $C_{28}H_{33}NO_4SNa$: 502.2028; found: 502.2021.

***tert*-Butyl (5-(methylthio)-1-phenyl-1-tosylpentan-3-yl)carbamate (5i):**

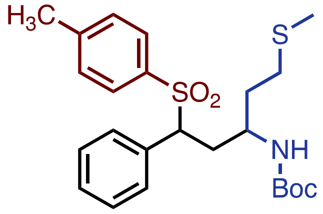

5i was synthesized following general procedure A; the d.r. was determined from the NMR spectrum of the crude mixture 5:1. Isolated yield of major diastereomer 33% (15 mg) (eluent: EtOAc/Pentane = 1:4), white solid.

^{1}H NMR (400 MHz, $CDCl_3$): δ 7.35 (dd, *J* = 8.3, 2.8 Hz, 2H), 7.27-7.19 (m, 3H), 7.14 (d, *J* = 8.1 Hz, 2H), 7.09-7.06 (m, 2H), 4.39-4.20 (m, 1H), 4.13 (d, *J* = 10.7 Hz, 1H), 3.72-3.42 (br. m, 1H), 2.64-2.37 (m, 4H), 2.37 (s, 3H), 2.02 (s, 3H), 1.76-1.72 (m, 2H), 1.36 (app d, *J* = 28.7 Hz, 9H).

^{13}C NMR (101 MHz, $CDCl_3$): δ 155.1, 144.5, 134.2+134.0 (rotameric peaks), 129.9, 129.7, 129.3+129.2 (rotameric peaks), 129.0, 128.9, 128.5, 79.3, 69.2+68.8 (rotameric peaks), 49.3+47.6 (rotameric peaks), 36.0+34.4 (rotameric peaks), 33.7+33.3 (rotameric peaks), 30.4, 28.3, 21.6, 15.5+15.5 (rotameric peaks).

HRMS: $[M+Na]^+$ calculated for $C_{24}H_{33}NO_4S_2Na$: 486.1748; found: 486.1744.

***tert*-Butyl methyl(4-phenyl-4-tosylbutan-2-yl)carbamate (5j):**

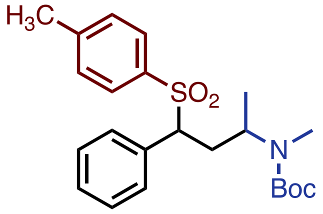

5j was synthesized following general procedure A; the d.r. was determined from the ^{1}H NMR spectrum of the crude mixture 4:1. Isolated yield of major diastereomer 44% (18 mg) (eluent: EtOAc/Pentane = 1:4), colourless viscous oil.

^{1}H NMR (400 MHz, CDCl$_3$): δ 7.42-6.98 (m, 9H), 4.43-3.93 (m, 2H), 2.71-2.44 (m, 3H), 2.35 (s, 3H), 2.30-2.12 (m, 2H), 1.34 (s, 9H), 1.07 (d, J = 6.8 Hz, 3H).

^{13}C NMR (101 MHz, CDCl$_3$): δ 155.3, 144.4, 134.3, 134.1, 132.5, 130.0+129.9 (rotameric peaks), 129.7, 129.2, 129.0, 128.4 (br. s), 79.7+79.2 (rotameric peaks), 69.9+69.1 (rotameric peaks), 49.4, 32.2+31.3 (rotameric peaks), 28.4, 28.3, 21.6, 18.5+18.2 (rotameric peaks).

HRMS: [M+Na]$^{+}$ calculated for $C_{23}H_{31}NO_4SNa$: 440.1871; found: 440.1867.

***tert*-Butyl (1,4-diphenyl-4-tosylbutan-2-yl)(methyl)carbamate (5k):**

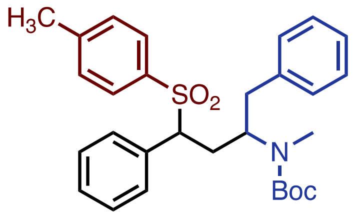

5k was synthesized following general procedure A; isolated yield of major diastereomer 31% (15 mg) (eluent: EtOAc/Pentane = 1:4), yellow oil.

^{1}H NMR (400 MHz, CDCl$_3$): δ 7.32, 7.28 (2xd, 8.1 Hz, 2H), 7.25-7.16 (m, 6H), 7.14-7.06 (m, 4H), 7.01 (d, J = 7.4 Hz, 2H), 4.52-4.38 (m, 1H), 4.07, 3.96 (2xd, 5.1, 3.5 Hz, 1H), 2.86-2.69 (m, 3H), 2.47 (s, 2H), 2.37-2.31 (m, 4H), 2.06 (s, 1H), 1.20, 1.20, 1.17 (2xs, 9H).

^{13}C NMR (101 MHz, CDCl$_3$): δ 155.5+155.2 (rotameric peaks), 144.5+144.2 (rotameric peaks), 138.1+137.8, 134.2+134.0 (rotameric peaks), 132.9, 129.7, 129.2, 129.0, 128.9+128.8 (rotameric peaks), 128.7, 128.4, 128.2, 126.4+126.3 (rotameric peaks), 79.5+79.0 (rotameric peaks), 69.9+69.1 (rotameric peaks), 55.6, 38.9+38.5 (rotameric peaks), 31.8, 30.4, 28.1, 21.6.

HRMS: [M+Na]$^{+}$ calculated for $C_{29}H_{35}NO_4SNa$: 516.2184; found: 516.2178.

(3-Tosylpropane-1,1,3-triyl)tribenzene (5l):

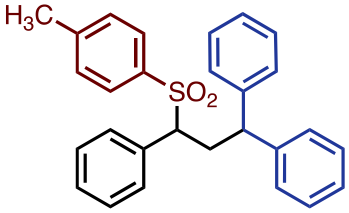

5l was synthesized following general procedure A; isolated yield 41% (17 mg) (eluent: EtOAc/Pentane = 1:9), white solid.

^{1}H NMR (400 MHz, $CDCl_3$): δ 7.36-7.30 (m, 3H), 7.29-7.20 (m, 7H), 7.18-7.11 (m, 5H), 7.05-7.02 (m, 4H), 3.85 (dd, J = 11.6, 3.3 Hz, 1H), 3.69 (dd, J = 12.0, 4.1 Hz, 1H), 3.19 (ddd, J = 13.7, 12.0, 3.3 Hz, 1H), 2.77 (ddd, J = 13.7, 11.6, 4.1 Hz, 1H), 2.37 (s, 3H).

^{13}C NMR (101 MHz, $CDCl_3$): δ 144.3, 144.1, 141.8, 134.3, 131.9, 130.1, 129.2, 129.0, 128.9, 128.8, 128.6, 128.5, 128.1, 127.4, 126.8, 126.5, 69.5, 47.6, 33.3, 21.6.

HRMS: $[M+NH_4]^+$ calculated for $C_{28}H_{30}NO_2S$: 444.1997; found: 444.1994.

1-(2-Phenyl-2-tosylethyl)adamantane (5m):

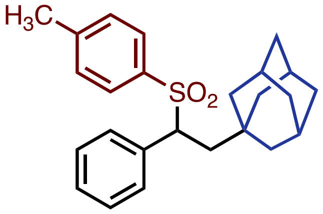

5m was synthesized following general procedure A; isolated yield 56% (22 mg) (eluent: EtOAc/Pentane = 1:9), white solid.

^{1}H NMR (400 MHz, $CDCl_3$): δ 7.37-7.32 (m, 2H), 7.25-7.16 (m, 3H), 7.16-7.07 (m, 4H), 4.13 (dd, J = 10.5, 1.8 Hz, 1H), 2.37 (s, 3H), 2.26 (dd, J = 14.2, 1.8 Hz, 1H), 2.00 (dd, J = 14.2, 10.5 Hz, 1H), 1.86-1.83 (m, 3H), 1.62-1.59 (m, 3H), 1.52-1.47 (m, 3H), 1.39-1.34 (m, 3H), 1.31-1.26 (m, 3H).

^{13}C NMR (101 MHz, $CDCl_3$): δ 144.1, 134.7, 134.4, 130.1, 129.1, 129.0, 128.3, 128.2, 67.6, 42.6, 41.1, 36.7, 33.1, 28.5, 21.6.

HRMS: $[M+NH_4]^+$ calculated for $C_{25}H_{34}NO_2S$: 412.2310; found: 412.2305.

1-(2-(3-fluorophenyl)-2-tosylethyl)adamantane (5n):

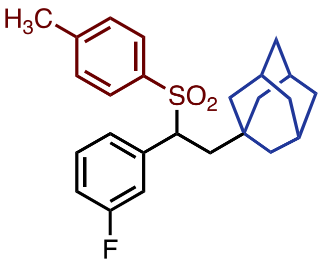

5n was synthesized following general procedure A; isolated yield 58% (24 mg) (eluent: EtOAc/Pentane = 1:9), white solid.

^{1}H NMR (400 MHz, $CDCl_3$): δ 7.38 (d, *J* = 8.3 Hz, 2H), 7.20-7.12 (m, 3H), 6.96 (ddd, *J* = 8.4, 2.5, 1.0 Hz, 1H), 6.91-6.84 (m, 2H), 4.12 (dd, *J* = 10.6, 1.7 Hz, 1H), 2.39 (s, 3H), 2.25 (dd, *J* = 14.3, 1.7 Hz, 1H), 1.94 (dd, *J* = 14.3, 10.5 Hz, 1H), 1.89-1.83 (m, 3H), 1.63-1.60 (d, *J* = 12.4 Hz, 3H), 1.54-1.48 (m, 3H), 1.39 -1.34 (m, 3H), 1.29-1.25 (m, 3H).

^{13}C NMR (101 MHz, $CDCl_3$): δ 161.8 (d, *J* = 235.2 Hz), 144.5, 137.3 (d, *J* = 10.7 Hz), 134.2, 129.6, 129.3, 126.0, 116.9 (d, *J* = 23.6 Hz), 115.4 (d, *J* = 21.4 Hz), 67.2, 67.2, 42.6, 41.2, 36.7, 33.1, 28.4, 21.6.

^{19}F NMR (376 MHz, $CDCl_3$): δ -112.6.

HRMS: $[M+NH_4]^+$ calculated for $C_{25}H_{33}FNO_2S$: 430.2216; found: 430.2212.

2-(2-Phenyl-2-tosylethyl)indoline (5o):

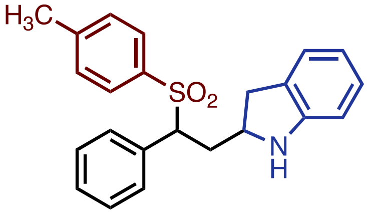

5o was synthesized following general procedure A; the d.r. was determined from the ^{1}H NMR spectrum of the crude mixture 1.2:1. Isolated yield of mixture of diastereomers 14% (5 mg) (eluent: EtOAc/Pentane = 1:3), white solid.

^{1}H NMR (400 MHz, $CDCl_3$): δ 7.43-7.37 (m, 2H), 7.30-7.22 (m, 3H), 7.19-7.10 (m, 4H), 7.02-6.94 (m, 2H), 6.66 (tdd, *J* = 7.4, 3.6, 1.1 Hz, 1H), 6.51 (app dd, *J* = 14.4, 7.7 Hz, 1H), 4.24 (ddd, *J* = 15.2, 10.5, 4.6 Hz, 1H), 4.00-3.89 (m, 1H), 3.73 (app qd, *J* = 8.8, 4.5 Hz, 1H), 3.07 (dd, *J* = 15.5, 8.8 Hz, 1H), 2.76-2.57 (m, 2H), 2.54-2.41 (m, 1H), 2.38 (app d, *J* = 3.4 Hz, 3H).

^{13}C NMR (101 MHz, $CDCl_3$): δ 150.5, 150.3, 144.5, 134.3, 134.2, 132.8, 132.2, 129.9, 129.7, 129.3, 129.2, 129.05, 129.00, 128.93, 128.9, 128.7, 128.6, 127.9, 127.40, 127.35, 124.6, 124.5, 119.1, 118.8, 109.7, 109.3, 69.2, 57.6, 56.7, 36.7, 36.0, 35.2, 34.9, 21.6.

HRMS: $[M+H]^+$ calculated for $C_{23}H_{24}NO_2S$: 378.1527; found: 378.1524.

***tert*-Butyl 2-(2-phenyl-2-(phenylsulfonyl)ethyl)pyrrolidine-1-carboxylate (6a):**

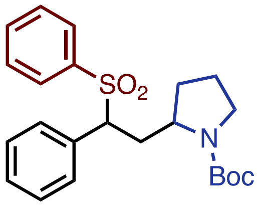

6a was synthesized following general procedure A; isolated yield of major diastereomer 61% (25 mg) (eluent: EtOAc/Pentane = 1:4), white solid.

^{1}H NMR (400 MHz, $CDCl_3$): δ 7.55 (t, *J* = 7.0 Hz, 1H), 7.46 (d, *J* = 7.7 Hz, 2H), 7.37 (d, *J* = 7.6 Hz, 2H), 7.26 (br. s, 1H), 7.20 (t, *J* = 7.3 Hz, 2H), 7.11 (d, *J* = 7.7 Hz, 2H), 4.29-3.90 (m, 1H), 3.83-3.06 (br. m, 3H), 2.89-2.59 (m, 1H), 2.25 (app t, *J* = 12.2 Hz, 1H), 1.92-1.57 (m, 4H), 1.41 (s, 9H).

^{13}C NMR (101 MHz, $CDCl_3$): δ 154.2, 136.8, 133.5, 131.3, 130.0, 129.2, 129.0, 128.6, 128.4, 79.8+79.0 (rotameric peaks), 69.3, 54.7+54.3 (rotameric peaks), 46.2, 31.0, 29.8, 28.6, 23.6+22.9 (rotameric peaks).

HRMS: $[M+Na]^+$ calculated for $C_{23}H_{29}NO_4SNa$: 438.1715; found: 438.1712.

***tert*-Butyl 2-(2-((4-chlorophenyl)sulfonyl)-2-phenylethyl)pyrrolidine-1-carboxylate (6b):**

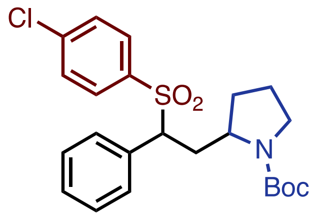

6b was synthesized following general procedure A; isolated yield of major diastereomer 52% (23 mg) (eluent: EtOAc/Pentane = 1:4), white solid.

^{1}H NMR (400 MHz, $CDCl_3$): δ 7.37 (d, J = 8.3 Hz, 2H), 7.32 (app d, J = 8.7 Hz, 3H), 7.23 (t, J = 7.0 Hz, 2H), 7.12 (d, J = 7.5 Hz, 2H), 4.31-3.88 (m, 1H), 3.78-3.07 (br. m, 3H), 2.74-2.67 (m, 1H), 2.29-2.23 (m, 1H), 1.93-1.61 (m, 4H), 1.42 (s, 9H).

^{13}C NMR (101 MHz, $CDCl_3$): δ 154.2, 140.4, 135.3, 131.1 (br. s), 130.6, 130.0, 129.2, 128.9, 128.6, 79.8+79.1 (rotameric peaks), 69.4, 54.8+54.3 (rotameric peaks), 46.2, 30.9, 29.8, 28.5, 23.6+22.9 (rotameric peaks).

HRMS: $[M+Na]^+$ calculated for $C_{23}H_{28}ClNO_4SNa$: 472.1325, 474.1298; found: 472.1320, 474.1292.

***tert*-Butyl 2-(2-((4-fluorophenyl)sulfonyl)-2-phenylethyl)piperidine-1-carboxylate (6c):**

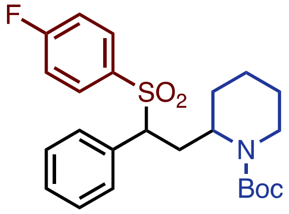

6c was synthesized following general procedure A; isolated yield of major diastereomer 43% (19 mg) (eluent: EtOAc/Pentane = 1:4), white solid.

^{1}H NMR (400 MHz, $CDCl_3$): δ 7.46-7.41 (m, 2H), 7.27-7.23 (m, 1H), 7.19 (t, J = 7.1 Hz, 2H), 7.10-7.04 (m, 2H), 7.00 (t, J = 8.6 Hz, 2H), 4.43-3.42 (m, 3H), 2.71 (br. s, 1H), 2.47 (br. s, 2H), 1.65-1.44 (m, 6H), 1.34 (s, 9H).

^{13}C NMR (101 MHz, CDCl$_3$): δ 166.7 (d, J = 242.6 Hz), 154.5, 133.0, 131.7 (d, J = 8.6 Hz), 129.7, 128.8, 128.5, 115.7 (d, J = 21.6 Hz), 79.4, 69.6, 49.0, 38.8, 29.6, 28.4, 26.8, 25.2, 18.8.
^{19}F NMR (376 MHz, CDCl$_3$): δ -103.6.

HRMS: $[M+Na]^+$ calculated for $C_{24}H_{30}FNO_4SNa$: 470.1777; found: 470.1773.

***tert*-Butyl 2-(2-((3-bromophenyl)sulfonyl)-2-phenylethyl)pyrrolidine-1-carboxylate (6d):**

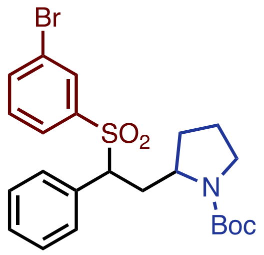

6d was synthesized following general procedure A; isolated yield of major diastereomer 56% (27 mg) (eluent: EtOAc/Pentane = 1:4), white solid.

^{1}H NMR (400 MHz, CDCl$_3$): δ 7.66-7.62 (m, 1H), 7.56 (br. s, 1H), 7.35 (app dt, J = 7.8, 1.3 Hz, 1H), 7.31 (d, J = 8.3 Hz, 1H), 7.27-7.19 (m, 3H), 7.12 (d, J = 7.5 Hz, 2H), 4.29-3.88 (m, 1H), 3.83-3.10 (br. m, 3H), 2.73 (br. s, 1H), 2.27 (br. s, 1H), 1.97-1.60 (m, 4H), 1.43 (s, 9H).

^{13}C NMR (101 MHz, CDCl$_3$): δ 154.2, 138.6, 136.5, 132.1, 130.9, 130.0, 129.2, 129.1, 128.5, 127.7, 122.7, 79.8, 69.5, 54.8+54.3 (rotameric peaks), 46.3, 30.8, 29.8, 28.6+28.5 (rotameric peaks), 23.6+22.9 (rotameric peaks).

HRMS: $[M+Na]^+$ calculated for $C_{23}H_{28}BrNO_4SNa$: 516.0820 and 518.0796; found: 516.0816 and 518.0797.

***tert*-Butyl 2-(2-(naphthalen-2-ylsulfonyl)-2-phenylethyl)pyrrolidine-1-carboxylate (6e):**

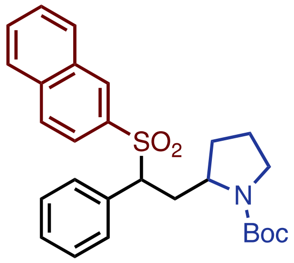

6e was synthesized following general procedure A; isolated yield of major diastereomer 37% (17 mg) (eluent: EtOAc/Pentane = 1:4), white solid.

^{1}H NMR (400 MHz, CDCl$_3$): δ 8.06 (br. s, 1H), 7.93-7.72 (m, 3H), 7.68-7.53 (m, 2H), 7.41 (dd, J = 8.6, 1.9 Hz, 1H), 7.30-7.09 (m, 5H), 4.40-3.98 (m, 1H), 3.80-3.04 (br. m, 3H), 2.79-2.76 (m, 1H), 2.40-2.31 (m, 1H), 1.94-1.60 (m, 4H), 1.38 (s, 9H).

^{13}C NMR (101 MHz, CDCl$_3$): δ 154.4, 135.3, 131.9, 131.3, 130.2, 129.5, 129.3, 128.7, 128.5, 127.9, 127.6, 123.9, 79.9, 69.5, 54.4, 46.3, 31.2, 29.9, 28.6, 23.0.

HRMS: $[M+Na]^+$ calculated for $C_{27}H_{31}NO_4SNa$: 488.1871; found: 488.1868.

***tert*-Butyl 2-(2-((3-methoxy-3-oxopropyl)sulfonyl)-2-phenylethyl)pyrrolidine-1-carboxylate (6g):**

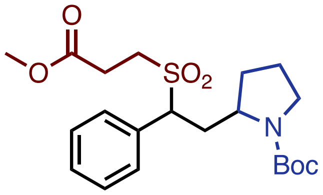

6f was synthesized following general procedure A; isolated yield of major diastereomer 19% (8 mg) (eluent: EtOAc/Pentane = 1:2), white solid.

^{1}H NMR (400 MHz, CDCl$_3$): δ 7.61-7.36 (m, 5H), 4.40-3.96 (m, 1H), 3.67 (s, 3H), 3.56-3.16 (br. m, 3H), 3.20-3.04 (m, 1H), 2.95 (br. s, 1H), 2.72-2.64 (m, 2H), 2.60 (ddd, J = 17.3, 9.3, 5.9 Hz, 1H), 2.26 (app d, J = 12.4 Hz, 1H), 1.94-1.65 (m, 4H), 1.42 (s, 9H).

^{13}C NMR (101 MHz, CDCl$_3$): δ 170.9, 131.8, 129.6, 129.2, 79.8, 67.2, 54.3, 52.3, 46.2, 45.8+45.2 (rotameric peaks), 30.7, 29.7, 28.5+28.3 (rotameric peaks), 26.3, 23.6, 22.9. (The carbonyl carbon signal of carbamate was not observed)

HRMS: $[M+Na]^+$ calculated for $C_{21}H_{31}NO_6SNa$: 448.1769; found: 448.1767.

***tert*-Butyl 2-(2-(ethylsulfonyl)-2-phenylethyl)pyrrolidine-1-carboxylate (6h):**

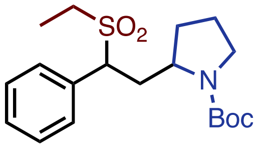

6g was synthesized following general procedure A; isolated yield of mixture of diastereomer 22% (8 mg) (eluent: EtOAc/Pentane = 1:2), white solid.

^{1}H NMR (400 MHz, CDCl$_3$): δ 7.55-7.44 (m, 2H), 7.42-7.32 (m, 3H), 4.28-3.92 (m, 1H), 3.82-3.08 (br. m, 3H), 2.69 (app dt, J = 15.0, 7.2 Hz, 3H), 2.28-2.25 (m, 1H), 1.97-1.68 (m, 4H), 1.42 (s, 9H), 1.24 (t, J = 7.5 Hz, 3H).

^{13}C NMR (101 MHz, CDCl$_3$): δ 154.2, 132.3, 129.6, 129.5, 129.1, 79.7+79.1 (rotameric peaks), 66.1+65.7 (rotameric peaks), 54.8+54.4 (rotameric peaks), 46.1, 44.8, 30.7, 29.8, 28.5, 23.6+22.9 (rotameric peaks), 6.1.

HRMS: $[M+Na]^+$ calculated for $C_{19}H_{29}NO_4SNa$: 390.1715; found: 390.1710.

***tert*-Butyl 2-(2-((8*R*,9*S*,13*S*,14*S*)-13-methyl-17-oxo-7,8,9,11,12,13,14,15,16,17-decahydro-6*H*-cyclopenta[*a*]phenanthren-3-yl)-2-tosylethyl)pyrrolidine-1-carboxylate (7a):**

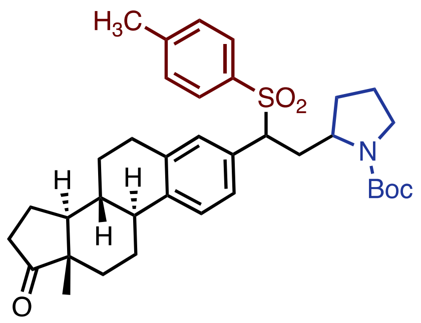

7a was synthesized following general procedure A; the d.r. was determined from the ^{1}H NMR spectrum of the crude mixture 4:1. Isolated yield of major of diastereomer 41% (25 mg) (eluent: EtOAc/Pentane = 1:4), colourless viscous oil.

^{1}H NMR (600 MHz, $CDCl_3$): δ 7.42 (app s, 2H), 7.24-7.10 (m, 3H), 6.92-6.81 (m, 2H), 4.06-3.91 (m, 1H), 3.62-3.22 (br. m, 3H), 2.84-2.59 (m, 3H), 2.50 (dd, *J* = 19.1, 8.7 Hz, 1H), 2.40 (s, 3H), 2.37 (d, *J* = 10.2 Hz, 1H), 2.26 (s, 1H), 2.14 (dt, *J* = 18.6, 8.9 Hz, 1H), 2.05 (dd, *J* = 13.0, 6.5 Hz, 2H), 1.97 (app t, *J* = 16.6 Hz, 2H), 1.78-1.70 (m, 2H), 1.58-1.47 (m, 8H), 1.40 (app d, *J* = 6.9 Hz, 9H), 0.91 (app d, *J* = 4.8 Hz, 3H).

^{13}C NMR (151 MHz, $CDCl_3$): δ 220.6, 154.2, 144.5, 140.6, 136.5, 133.9, 130.9+130.6, 129.4, 129.2, 128.3, 127.3+127.1, 125.3+125.3, 79.6, 69.0, 54.2 (br. s), 50.52, 50.51, 47.9, 46.2 (br. s), 44.3, 38.0, 35.8, 31.5, 29.9, 29.3+29.2 (rotameric peaks), 28.6, 26.4, 25.6, 22.9, 21.60, 21.58, 21.56, 13.85+13.84 (rotameric peaks).

HRMS: $[M+NH_4]^+$ calculated for $C_{36}H_{51}N_2O_5S$: 623.3518; found: 623.3513.

***tert*-Butyl 2-(2-(1-((2*R*,3*R*,4*S*,5*R*)-3,4-dihydroxy-5-(hydroxymethyl)tetrahydrofuran-2-yl)-2,4-dioxo-1,2,3,4-tetrahydropyrimidin-5-yl)-2-tosylethyl)pyrrolidine-1-carboxylate (7b):**

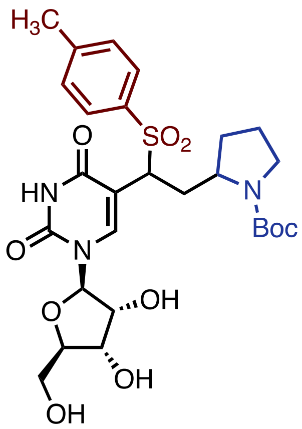

7b was synthesized following general procedure A; the d.r. was determined after purification 1.6:1.

Diastereomer 1: Isolated yield of major diastereomer 41% (25 mg) (eluent: MeOH/DCM = 5:95), white solid.

^{1}H NMR (500 MHz, CDCl$_3$): δ 9.34 (br. s, 1H), 8.26 (app d, *J* = 126.4 Hz, 1H), 7.65 (app dd, *J* = 8.0, 5.5 Hz, 2H), 7.29 (app dd, *J* = 13.4, 7.9 Hz, 2H), 6.08-5.74 (m, 1H), 4.55-4.22 (m, 4H), 4.07 (s, 1H), 3.99 (app dd, *J* = 25.7, 12.4 Hz, 1H), 3.77 (t, *J* = 11.1 Hz, 1H), 3.52 (s, 1H), 3.26 (br. s, 2H), 2.52 (app dt, *J* = 41.6, 13.2 Hz, 1H), 2.40 (s, 3H), 2.00-1.72 (m, 4H), 1.57 (app d, *J* = 38.8 Hz, 1H), 1.43 (app d, *J* = 8.1 Hz, 9H).

^{13}C NMR (101 MHz, CD$_3$OD): δ 163.0+162.8 (rotameric peaks), 155.0+154.8 (rotameric peaks), 150.2, 145.2, 141.4+141.0 (rotameric peaks), 134.0, 129.53+129.45 (rotameric peaks), 129.0, 104.8, 89.6+89.0 (rotameric peaks), 85.2+84.9 (rotameric peaks), 79.7+79.6 (rotameric peaks), 75.0+74.3 (rotameric peaks), 70.0+69.9 (rotameric peaks), 61.2+60.6 (rotameric peaks), 57.7, 54.6, 46.2, 30.3, 29.4+29.2 (rotameric peaks), 27.43+27.39 (rotameric peaks), 23.16+23.05 (rotameric peaks), 20.2.

HRMS: $[M+H]^+$ calculated for $C_{27}H_{38}N_3O_{10}S$: 596.2277; found: 596.2271.

Diastereomer 2: Isolated yield of minor diastereomer 25% (15 mg) (eluent: MeOH/DCM = 5:95), white solid.

^{1}H NMR (400 MHz, CDCl$_3$): δ 9.24 (app d, J = 40.3 Hz, 1H), 8.12 (app d, J = 53.8 Hz, 1H), 7.65 (dd, J = 11.1, 8.0 Hz, 2H), 7.33-7.24 (m, 2H), 5.89 (app d, J = 19.7 Hz, 1H), 4.73-4.26 (m, 4H), 4.14 (s, 1H), 4.02-3.72 (m, 4H), 3.31 (br. s, 1H), 3.13 (br. s, 1H), 2.40 (app d, J = 2.7 Hz, 3H), 2.29 (app d, J = 37.2 Hz, 1H), 2.10-1.70 (m, 4H), 1.47-1.41 (m, 1H), 1.35 (s, 9H).

^{13}C NMR (101 MHz, CDCl$_3$): δ 161.7, 155.2+154.9 (rotameric peaks), 150.4, 145.2+145.0 (rotameric peaks), 141.4, 134.3, 129.7, 129.1+129.0 (rotameric peaks), 106.2, 91.4+90.9 (rotameric peaks), 85.9+85.6 (rotameric peaks), 79.9, 75.1, 70.3, 61.0, 54.9, 47.1, 32.2+31.6 (rotameric peaks), 30.9+30.6 (rotameric peaks), 28.44+28.39 (rotameric peaks), 23.7, 21.6.

HRMS: [M+H]$^+$ calculated for $C_{27}H_{38}N_3O_{10}S$: 596.2277; found: 596.2273.

2-((4,4-Dimethyl-6-phenyl-6-tosylhexyl)oxy)-1,4-dimethylbenzene (7c):

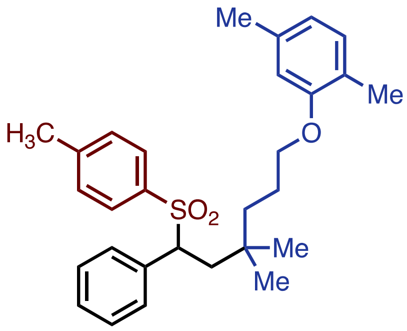

7c was synthesized following general procedure A; isolated yield 51% (23 mg) (eluent: EtOAc/Pentane = 1:9), white solid.

^{1}H NMR (400 MHz, CDCl$_3$): δ 7.36 (d, J = 8.3 Hz, 2H), 7.25-7.17 (m, 3H), 7.18-7.09 (m, 4H), 7.06-6.96 (m, 1H), 6.66 (dd, J = 7.2, 1.4 Hz, 1H), 6.57 (d, J = 1.6 Hz, 1H), 4.08 (dd, J = 10.5, 1.6 Hz, 1H), 3.86-3.63 (m, 2H), 2.47 (dd, J = 14.2, 1.7 Hz, 1H), 2.37 (s, 3H), 2.31 (s, 3H), 2.16 (app s, 4H), 1.80-1.65 (m, 1H), 1.64-1.57 (m, 1H), 1.35 (ddd, J = 13.5, 12.1, 4.6 Hz, 1H), 1.22 (ddd, J = 13.6, 12.4, 4.6 Hz, 1H), 0.78 (s, 3H), 0.77 (s, 3H).

^{13}C NMR (101 MHz, CDCl$_3$): δ 157.0, 144.2, 136.5, 134.4, 134.4, 130.2, 129.1, 129.0, 128.5, 128.3, 123.5, 120.6, 112.0, 68.9, 68.2, 38.3, 38.2, 33.5, 27.7, 27.5, 24.0, 21.6, 21.4, 15.8.

HRMS: [M+H]$^+$ calculated for $C_{29}H_{37}O_3S$: 465.2463; found: 465.2456.

4-Chloro-*N*-(4-((2-methyl-4-phenyl-4-tosylbutan-2-yl)oxy)phenethyl)benzamide (7d):

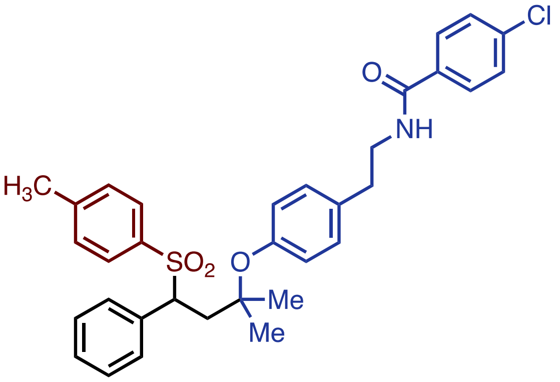

7d was synthesized following general procedure A; isolated yield 62% (35 mg) (eluent: EtOAc/Pentane = 2:3), white solid.

^{1}H NMR (400 MHz, $CDCl_3$): δ 7.65-7.57 (m, 2H), 7.34 (app dd, *J* = 8.3, 2.7 Hz, 4H), 7.28-7.10 (m, 7H), 7.10-7.00 (m, 2H), 6.81 (d, *J* = 8.3 Hz, 2H), 6.25 (br s, 1H), 4.52 (dd, *J* = 10.7, 1.8 Hz, 1H), 3.64 (app q, *J* = 6.6 Hz, 2H), 2.97 (dd, *J* = 14.4, 1.8 Hz, 1H), 2.85 (t, *J* = 7.0 Hz, 2H), 2.45 (dd, *J* = 14.3, 10.7 Hz, 1H), 2.36 (s, 3H), 1.20 (s, 3H), 0.94 (s, 3H).

^{13}C NMR (101 MHz, $CDCl_3$): δ 166.4, 153.5, 144.4, 137.6, 134.2, 134.0, 133.8, 133.0, 130.2, 129.3, 129.2, 129.0, 128.7, 128.5, 128.4, 128.3, 123.9, 79.3, 68.2, 41.3, 39.3, 34.9, 27.9, 27.4, 21.6.

HRMS: $[M+H]^+$ calculated for $C_{33}H_{35}ClNO_4S$: 576.1975 and 578.1955; found: 576.1967 and 578.1948.

***tert*-Butyl 2-(2-hydroxy-2-phenylethyl)pyrrolidine-1-carboxylate (8)[3]:**

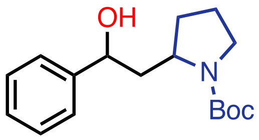

To an oven-dried crimp top vial sulfone **4a** (0.128 g, 0.3 mmol, 1 equiv) was added. The vial was sealed and evacuated and purged with N_2 three times. Anhydrous THF (3 mL) was added. The flask was cooled to -78 °C and *n*BuLi (0.24 mL, 0.6 mmol, 2 equiv, 2.5 M in THF) was added dropwise. The reaction was allowed to stir at -78 °C for 10 min before warming to 0 °C over 20 min. 9-Borabicyclo[3.3.1]nonane (1.8 mL, 0.9 mmol, 3 equiv, 0.5 M in THF) was

added. The reaction was allowed to warm to rt and stirred for 18 h. Following this the reaction was quenched by the sequential addition of H_2O (1 mL), 3 M NaOH (0.6 mL) and H_2O_2 (0.3 mL, 30% w/w) and allowed to stir at rt for 30 min. The product was extracted with EtOAc (3 x 5 mL). The combined organic layers were washed with brine (20 mL), dried over Na_2SO_4 and the solvent was removed under reduced pressure. The desired products was isolated by flash column chromatography (50% Et_2O/pentane) as two separable diastereomers (total yield: 45 mg, 51%).

Diastereomer 1 (8a): Isolated yield 27% (24 mg), yellow oil.

^{1}H NMR (400 MHz, $CDCl_3$): δ 7.40-7.38 (m, 2H), 7.32 (t, *J* = 7.5 Hz, 2H), 7.22 (t, *J* = 7.3 Hz, 1H), 5.43-5.41 (m, 1H), 4.71-4.59 (m, 1H), 4.30 (br. s, 1H), 3.40-3.36 (m, 2H), 2.06-1.96 (m, 1H), 1.92-1.85 (m, 2H), 1.75-1.68 (m, 2H), 1.61-1.57 (m, 1H), 1.49 (s, 9H).

^{13}C NMR (101 MHz, $CDCl_3$): δ 156.8, 144.5, 128.2, 126.8, 125.6, 80.1, 70.0, 54.0, 46.7, 46.4, 31.2, 28.5, 23.6.

Spectroscopic data were consistent with literature data.[4]

Diastereomer 2 (8b): Isolated yield 24% (21 mg), yellow oil.

^{1}H NMR (400 MHz, $CDCl_3$): δ 7.37-7.30 (m, 4H), 7.25-7.22 (m, 1H), 4.79-4.76 (m, 1H), 4.42 (br. s, 1H), 4.11 (br. s, 1H), 3.32-3.29 (m, 2H), 2.18-2.12 (m, 1H), 2.02-1.95 (m, 1H), 1.86-1.80 (m, 2H), 1.74-1.62 (m, 2H), 1.46 (s, 9H).

^{13}C NMR (101 MHz, $CDCl_3$): δ 155.6, 145.1, 128.3, 127.0, 125.6, 79.8, 72.7, 55.7, 46.4, 46.3, 32.5, 28.5, 23.8.

Spectroscopic data were consistent with literature data.[4]

(±)-Pyrrolallosedamine

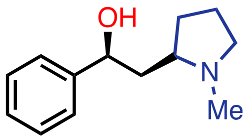

To an oven-dried crimp top vial evacuated and purged with N_2 three times, **8a** (24.0 mg, 0.082 mmol, 1equiv) was added. Dry THF (1.2 mL) was added. Following this, $LiAlH_4$ (0.41 mL, 0.41 mmol, 5 equiv, 1M solution in THF) was added dropwise. The reaction was heated at 60 °C on an aluminium heating block on a stirrer hot-plate for 14 h. Following this, the flask was cooled to 0 °C and 1 M HCl was added to pH 2. The mixture was diluted with water (5 mL) and extracted with CH_2Cl_2 (2 x 5 mL). The aqueous layer was then basified to pH 12 with 1 M NaOH and extracted with CH_2Cl_2 (5 x 5 mL). The combined organic layers were dried over Na_2SO_4, filtered and the solvent was removed *in vacuo* to give product (±)-pyrrolallosedamine as a colourless oil (13.8 mg, 82%).

^{1}H NMR (400 MHz, CDCl$_3$): δ 7.41-7.37 (m, 2H), 7.33 (ddd, *J* = 7.9, 6.8, 1.2 Hz, 2H), 7.26-7.21 (m, 1H), 5.08 (dd, *J* = 10.7, 2.5 Hz, 1H), 3.15 (ddd, *J* = 9.2, 6.8, 2.3 Hz, 1H), 2.67 (tt, *J* = 7.5, 3.5 Hz, 1H), 2.41 (s, 3H), 2.23 (td, *J* = 9.6, 7.2 Hz, 1H), 2.02-1.88 (m, 4H), 1.85-1.77 (m, 1H), 1.68 (dt, *J* = 14.8, 2.8 Hz, 1H).

^{13}C NMR (101 MHz, CDCl$_3$): δ 145.4, 128.2, 126.8, 125.6, 71.2, 65.0, 56.9, 40.6, 38.1, 28.3, 23.4.

HRMS: $[M+H]^+$ calculated for $C_{13}H_{20}NO$: 206.1544; found: 206.1543.

(±)-Pyrrolsedamine

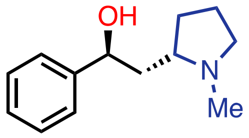

To an oven-dried crimp top vial evacuated and purged with N_2 three times, **8b** (20.7 mg, 0.071 mmol, 1 equiv) was added. Dry THF (1 mL) was added. Following this, $LiAlH_4$ (0.36 mL, 0.36 mmol, 5 equiv, 1M solution in THF) was added dropwise. The reaction was heated at 60 °C on an aluminium heating block on a stirrer hot-plate for 14 h. Following this, the flask was cooled to 0 °C and 1 M HCl was added to pH 2. The mixture was diluted with water (5 mL) and extracted with CH_2Cl_2 (2 x 5 mL). The aqueous layer was then basified to pH 12 with 1 M

NaOH and extracted with CH_2Cl_2 (5 x 5 mL). The combined organic layers were dried over Na_2SO_4, filtered and the solvent was removed *in vacuo* to give product (±)-pyrrolsedamine as a colourless oil (12,7 mg, 87%).

^{1}H NMR (400 MHz, $CDCl_3$): δ 7.39-7.30 (m, 4H), 7.26-7.20 (m, 1H), 4.86 (dd, *J* = 9.9, 2.6 Hz, 1H), 3.11 (ddd, *J* = 10.7, 7.3, 5.6 Hz, 1H), 2.91 (ddd, *J* = 8.3, 6.7, 4.2 Hz, 1H), 2.46-2.40 (m, 4H), 2.09-1.97 (m, 1H), 1.83-1.70 (m, 3H), 1.64 (ddd, *J* = 14.1, 5.7, 2.6 Hz, 1H), 1.41 (tdd, *J* = 7.7, 5.4, 3.9 Hz, 1H).

^{13}C NMR (101 MHz, $CDCl_3$): δ 145.5, 128.2, 127.0, 125.5, 73.9, 66.2, 55.3, 43.1, 30.4, 22.8.

Spectroscopic data were consistent with literature data.[5]

2-(2-Phenyl-2-tosylethyl)pyrrolidine (9):

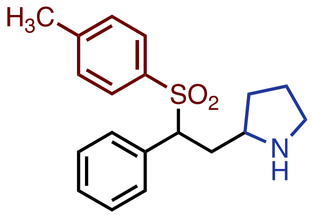

To a round bottom flask **4a** (0.15 mmol, 1 equiv) and trifluoroacetic acid (3 mmol, 20 equiv) were added. CH_2Cl_2 (3 mL) was added and the reaction was stirred overnight at rt. Following this, the solvent was removed under reduced pressure. Et_2O was added (10 mL) and the organic layer was washed with H_2O (3 x 5 mL). The combined aqueous layers were basified to pH 10 with $NaHCO_3$ and extracted with EtOAc (3 x 5 mL). The combined organic layers were dried over Na_2SO_4 and evaporated under reduced pressure gave the desired product **9** as a white solid (35 mg, 91%).

^{1}H NMR (500 MHz, $CDCl_3$): δ 7.40-7.35 (m, 2H), 7.27-7.24 (m, 1H), 7.24-7.19 (m, 2H), 7.16-7.09 (m, 4H), 4.16 (dd, *J* = 10.1, 4.9 Hz, 1H), 3.03-2.94 (m, 1H), 2.91 (ddd, *J* = 10.5, 7.5, 5.4 Hz, 1H), 2.75 (ddd, *J* = 10.4, 8.0, 6.5 Hz, 1H), 2.44 (ddd, *J* = 13.6, 7.9, 4.9 Hz, 1H), 2.37 (s, 3H), 2.30 (ddd, *J* = 13.6, 10.1, 6.2 Hz, 1H), 1.80-1.59 (m, 4H), 1.33-1.21 (m, 1H).
^{13}C NMR (126 MHz, $CDCl_3$): δ 144.3, 134.3, 132.9, 129.8, 129.2, 129.1, 128.7, 128.4, 69.9, 56.5, 46.5, 34.8, 31.2, 25.2, 21.6.

HRMS: $[M+H]^+$ calculated for $C_{19}H_{24}NO_2S$: 330.1527; found: 330.1523.

1-Methyl-4-((1-phenylvinyl)sulfonyl)benzene (10):

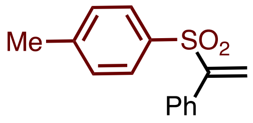

Alkyne (0.1 mmol, 1 equiv) was added to a crimptop vial followed by sodium *p*-toluenesulfinate (0.1 mmol, 1 equiv), *p*-toluenesulfinic acid (0.1 mmol, 1 equiv) and 4CzIPN (1 mol%) and closed. The vial was evacuated and purged with N_2 three times. Anhydrous DMSO (2 mL) was added. The mixture was stirred under blue LED irradiation for 14 hours. Then, the reaction mixture was quenched with H_2O (20 mL) and the aqueous layer was extracted with ethyl acetate (3 x 10 mL). The combined organic layers were washed with brine solution (40 mL) and dried over Na_2SO_4. The solvent was removed under reduced pressure. The desired products was isolated by flash column chromatography (10% EtOAc/pentane) as a colourless oil (10 mg, 41%).

^{1}H NMR (400 MHz, $CDCl_3$): δ 7.56 (d, *J* = 8.4 Hz, 2H), 7.37-7.30 (m, 3H), 7.28-7.27 (m, 2H), 7.19 (d, *J* = 7.6 Hz, 2H), 6.60 (s, 1H), 5.92 (s, 1H), 2.36 (s, 3H).

^{13}C NMR (101 MHz, $CDCl_3$): δ 151.2, 144.3, 135.8, 132.6, 129.5, 129.2, 129.1, 128.4, 128.2, 125.5, 21.6.

Spectroscopic data were consistent with literature data.[6]

1-Fluoro-3-(1-tosylvinyl)benzene (10f):

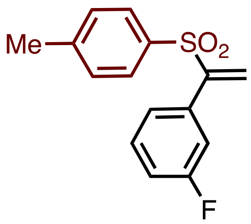

10f was synthesized following general procedure A, except reaction was quenched after 30 minutes; isolated yield 39% (11 mg) (eluent: EtOAc/Pentane = 1:9), colourless oil.

^{1}H NMR (400 MHz, $CDCl_3$: δ 7.58 (d, J = 8.0 Hz, 2H), 7.25-7.19 (m, 3H), 7.14-7.00 (m, 3H), 6.63 (s, 1H), 5.96 (s, 1H), 2.38 (s, 3H).

^{13}C NMR (101 MHz, $CDCl_3$): δ 162.9 (d, J = 252.0 Hz), 150.1, 144.6, 135.5, 134.5, 129.8, 129.6, 128.4, 126.1, 124.8 (d, J = 5.3 Hz), 116.2 (d, J = 12.3 Hz), 116.0 (d, J = 14.6 Hz), 21.6.

^{19}F NMR (376 MHz, $CDCl_3$): δ -112.38 (ddd, J = 9.6, 8.6, 5.8 Hz).

HRMS: $[M+H]^+$ calculated for $C_{15}H_{14}FO_2S$: 277.0698; found: 277.0692.

7. References:

1. Wang, H.; Lu, Q.; Chiang, C.-W.; Luo, Y.; Zhou, J.; Wang, G.; Lei, A. Markovnikov-Selective Radical Addition of S-Nucleophiles to Terminal Alkynes through a Photoredox Process. *Angew. Chem., Int. Ed.* **2017**, 56, 595-599.
2. a) Gao, Y.; Liu, S.; Su, W. CO2-Facilitated Radical Sequential (3 + 2) Annulationof 1,6-Enynes via Cooperation of Sulfinate Catalysis and Photocatalysis. *Green Chem.* **2023**, *25*, 7335-7343; b) García-Domínguez, A.; Müller, S.; Nevado, C. Nickel-Catalyzed Intermolecular Carbosulfonylation of Alkynes via Sulfonyl Radicals. *Angew. Chem., Int. Ed.* **2017**, 56, 9949-9952; c) Zhu, C.; Yue, H.; Maity, B.; Atodiresei, I.; Cavallo, L.; Rueping, M. A Multicomponent Synthesis of Stereodefined Olefins via Nickel Catalysis and Single Electron/Triplet Energy Transfer. *Nat. Catal.* **2019**, *2*, 678-687.
3. Liu, L.; Henderson, J. A.; Yamamoto, A.; Brémond, P.; Kishi, Y. *Org.* Synthesis of Alcohols from *m*-Fluorophenylsulfones and Dialkylboranes: Application to the C14–C35 Building Block of E7389. *Org. Lett.* **2012**, *14*, 2262-2265.
4. Mega, R. S.; Duong, V. K.; Noble, A.; Aggarwal, V. K. Decarboxylative Conjunctive Cross-coupling of Vinyl Boronic Esters using Metallaphotoredox Catalysis. *Angew. Chem., Int. Ed.* **2020**, *59*, 4375-4379.
5. Bhosale, V. A.; Markad, S. B.; Waghmode, S. B. A Concise Enantioselective Synthesis of Pyrrolidine Sedum Alkaloids (R)-(R)-(+), (S)-(S)-(−)-Pyrrolsedamine and (S)-(R)-(+)-Pyrrolallosedamine by Using Proline Catalysed α-Amination Reaction. *Tetrahedron* **2017**, *73*, 5344-5349.
6. Wang, H.; Lu, Q.; Chiang, C.-W.; Luo, Y.; Zhou, J.; Wang, G.; Lei, A. Markovnikov-Selective Radical Addition of S-Nucleophiles to Terminal Alkynes through a Photoredox Process. *Angew. Chem., Int. Ed.* **2017**, *56*, 595-599.

8. Copies of ^{1}H and ^{13}C NMR spectra

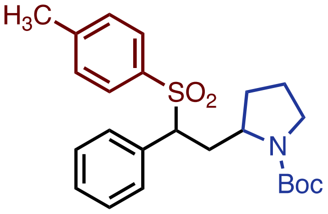

Major Diastereomer

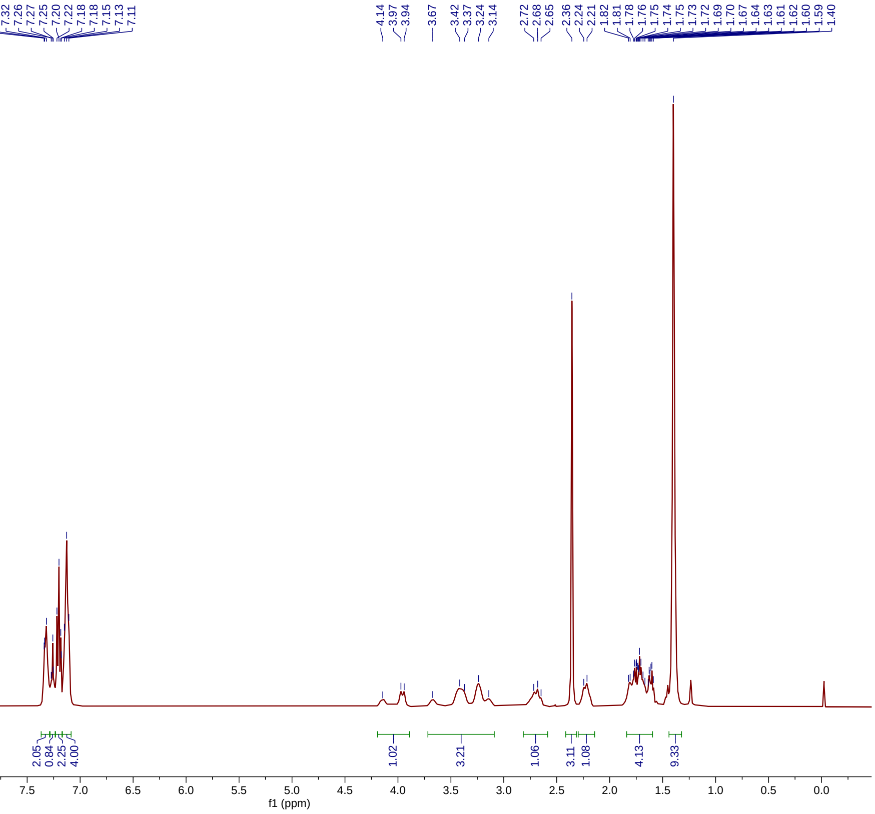

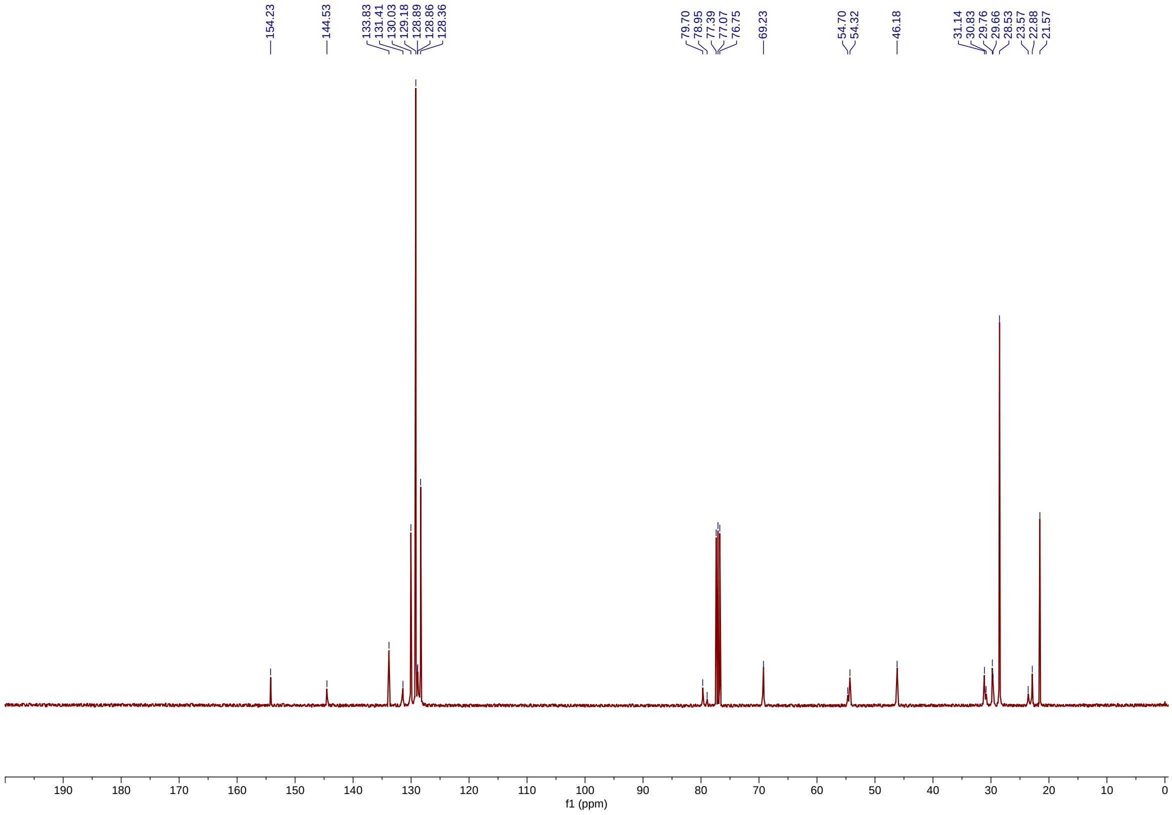

^{1}H (400 MHz) and ^{13}C (101 MHz) spectra of compound 4a in $CDCl_3$

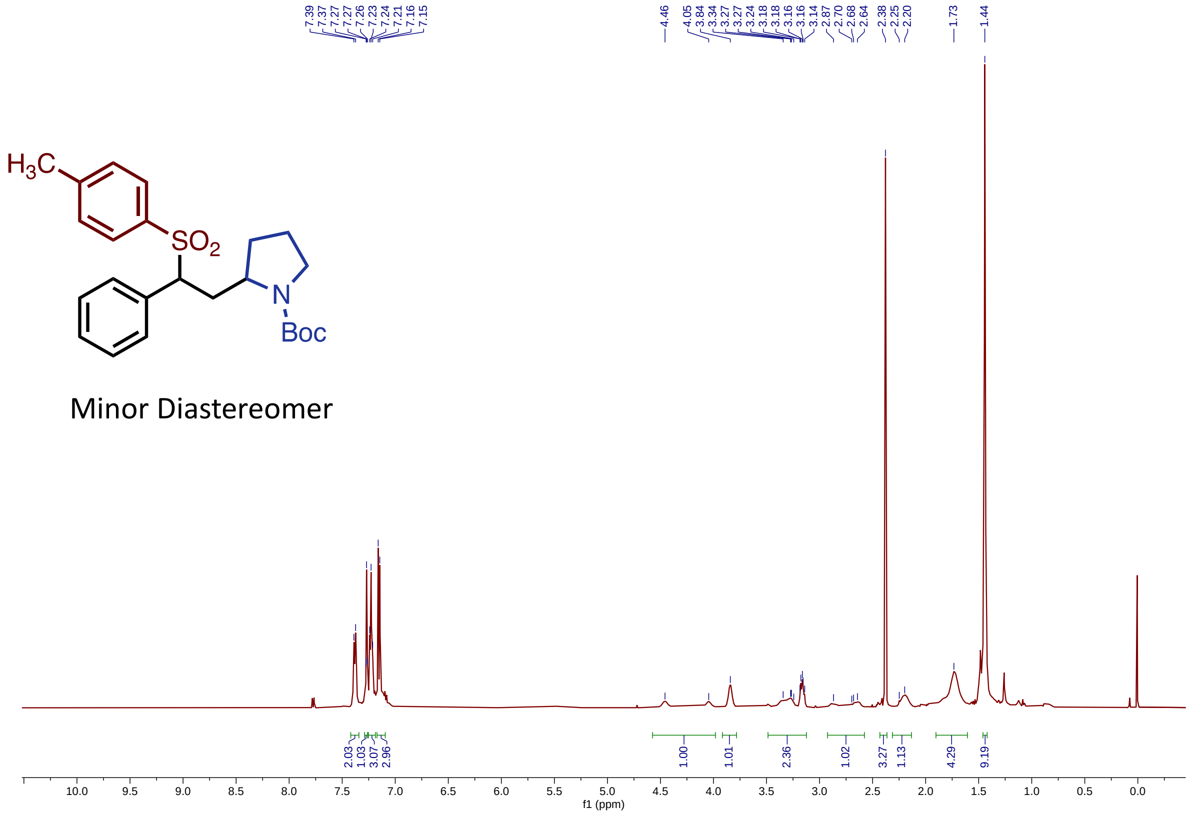

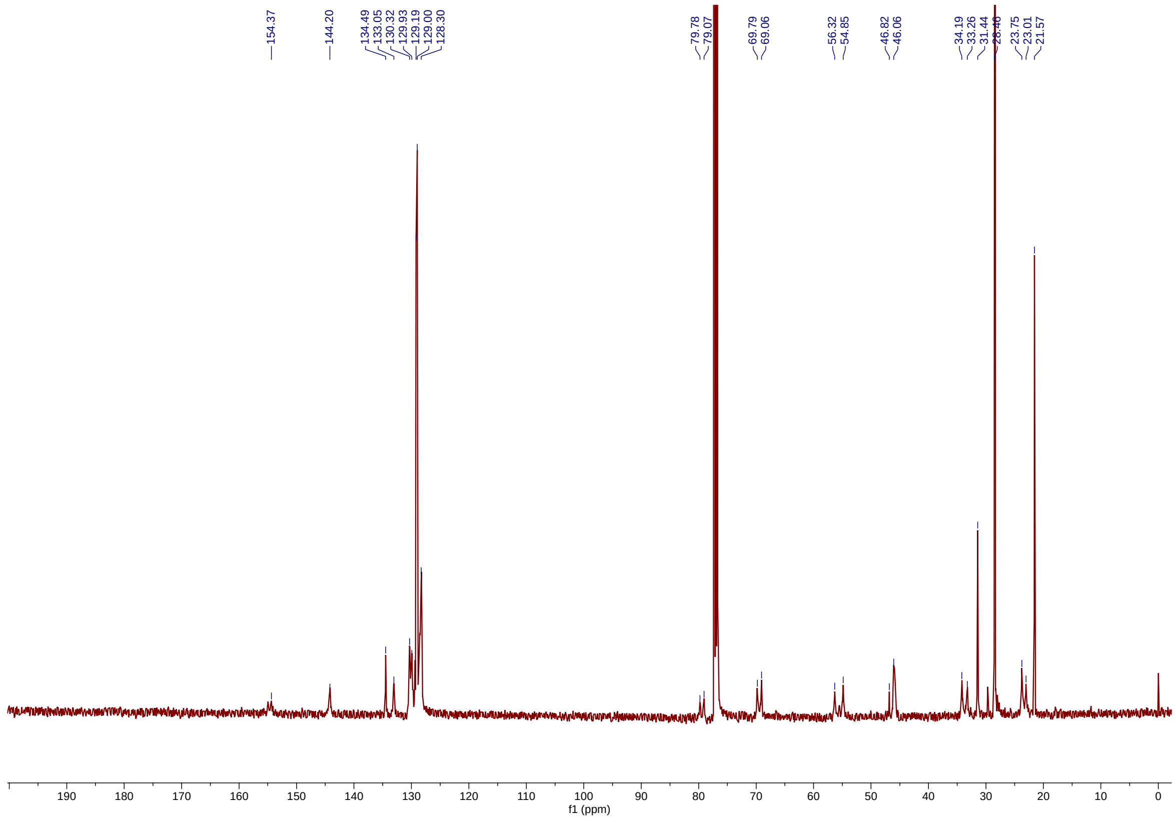

^{1}H (500 MHz) and ^{13}C (126 MHz) spectra of compound 4a' in $CDCl_3$

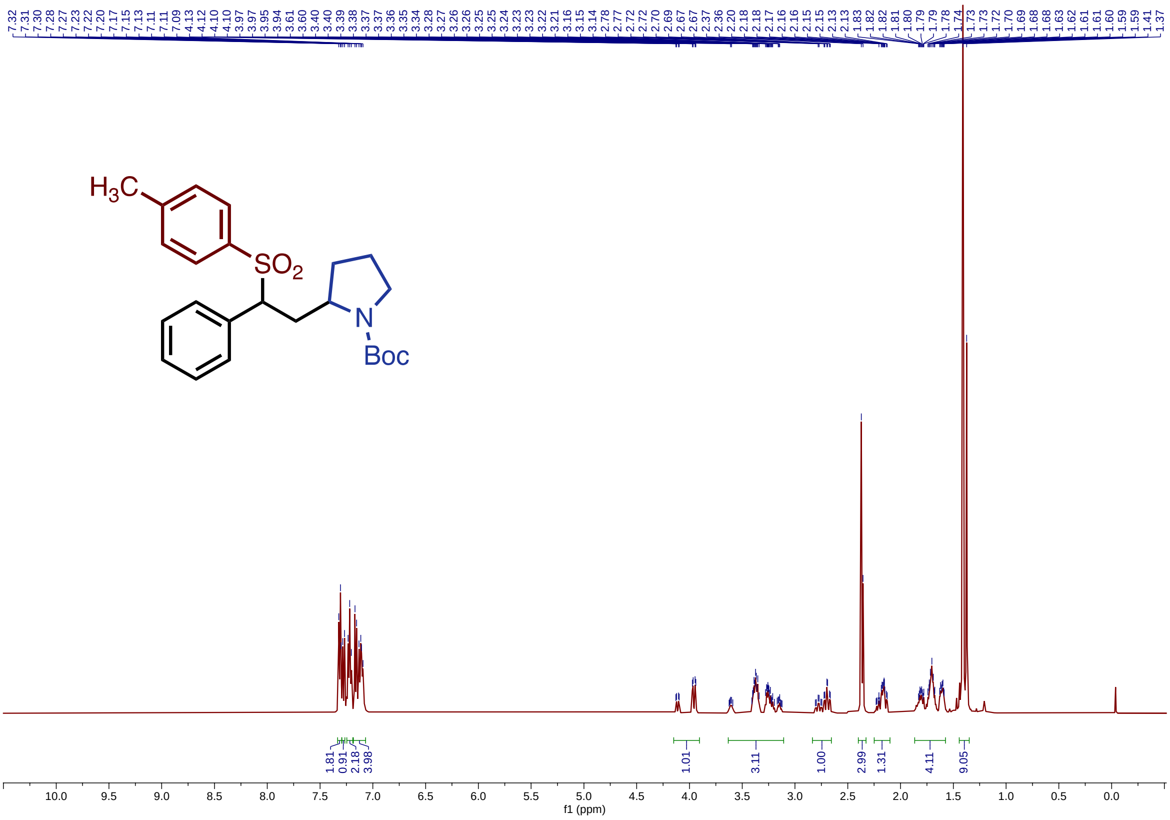

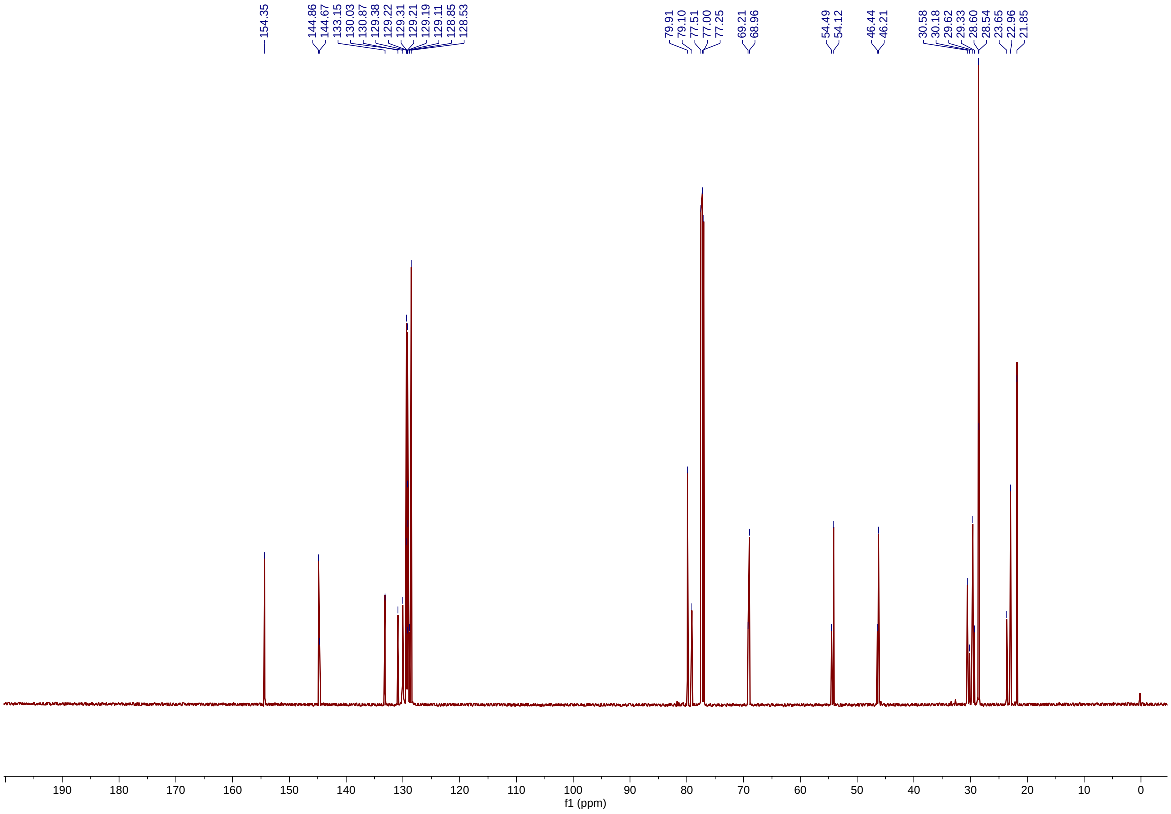

^{1}H (500 MHz) and ^{13}C (126 MHz) spectra of compound 4a in $CDCl_3$, -20 °C

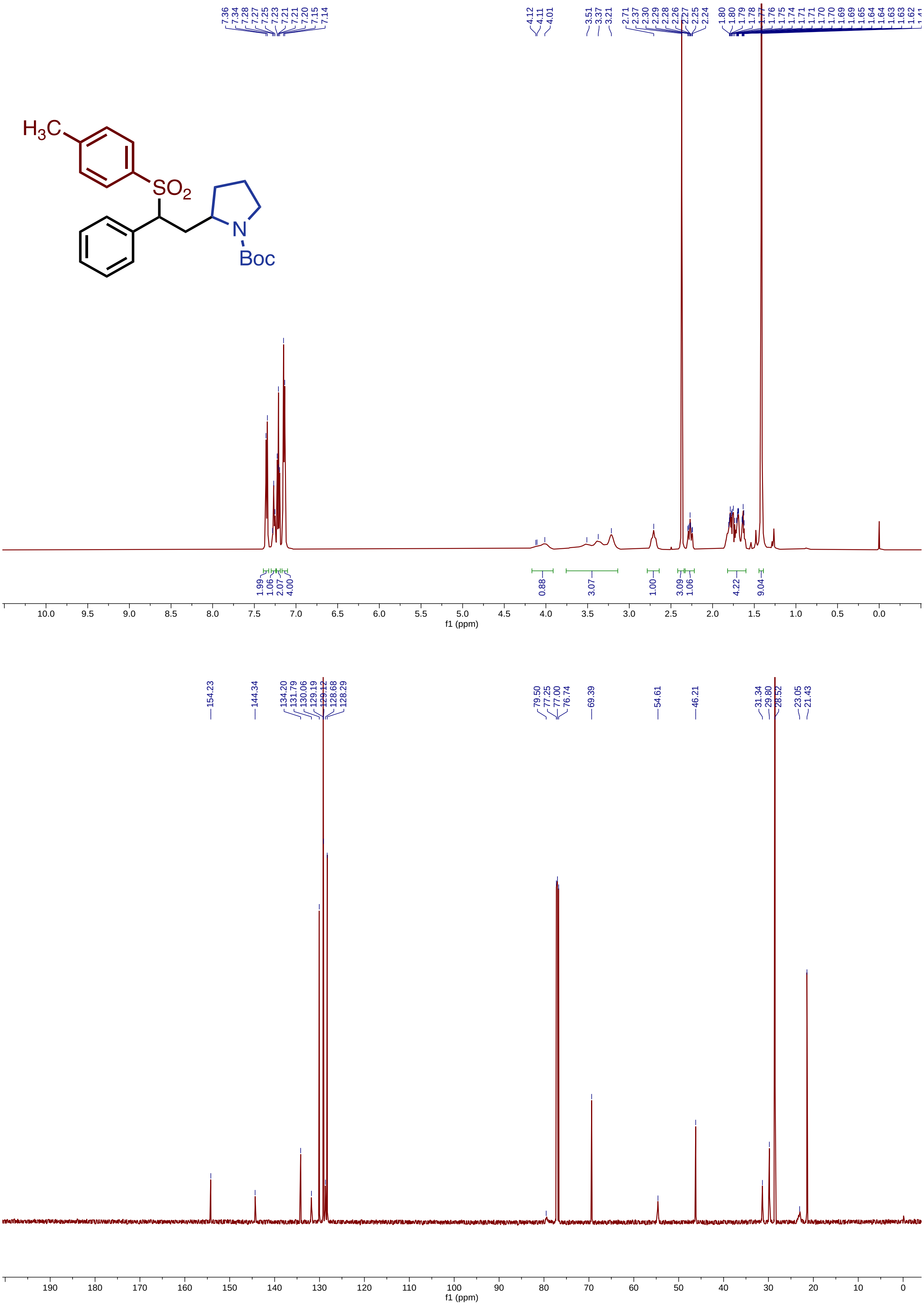

^{1}H (500 MHz) and ^{13}C (126 MHz) spectra of compound 4a in $CDCl_3$, 50 °C

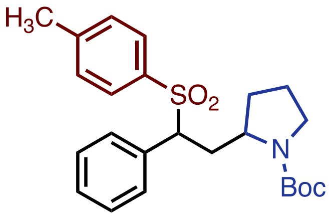

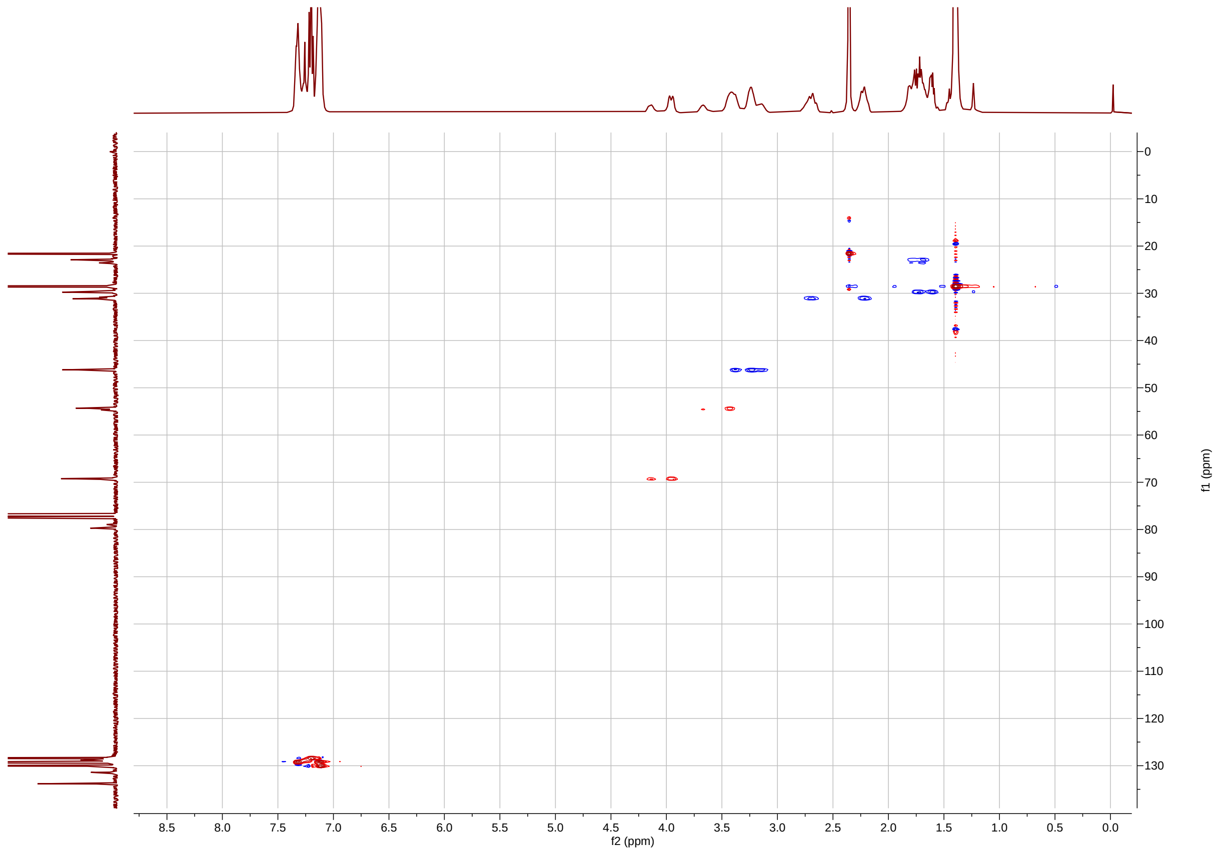

HSQC spectrum of compound 4a in $CDCl_3$

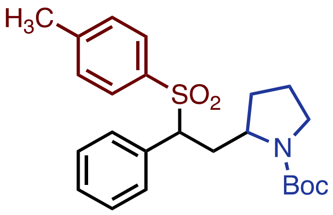

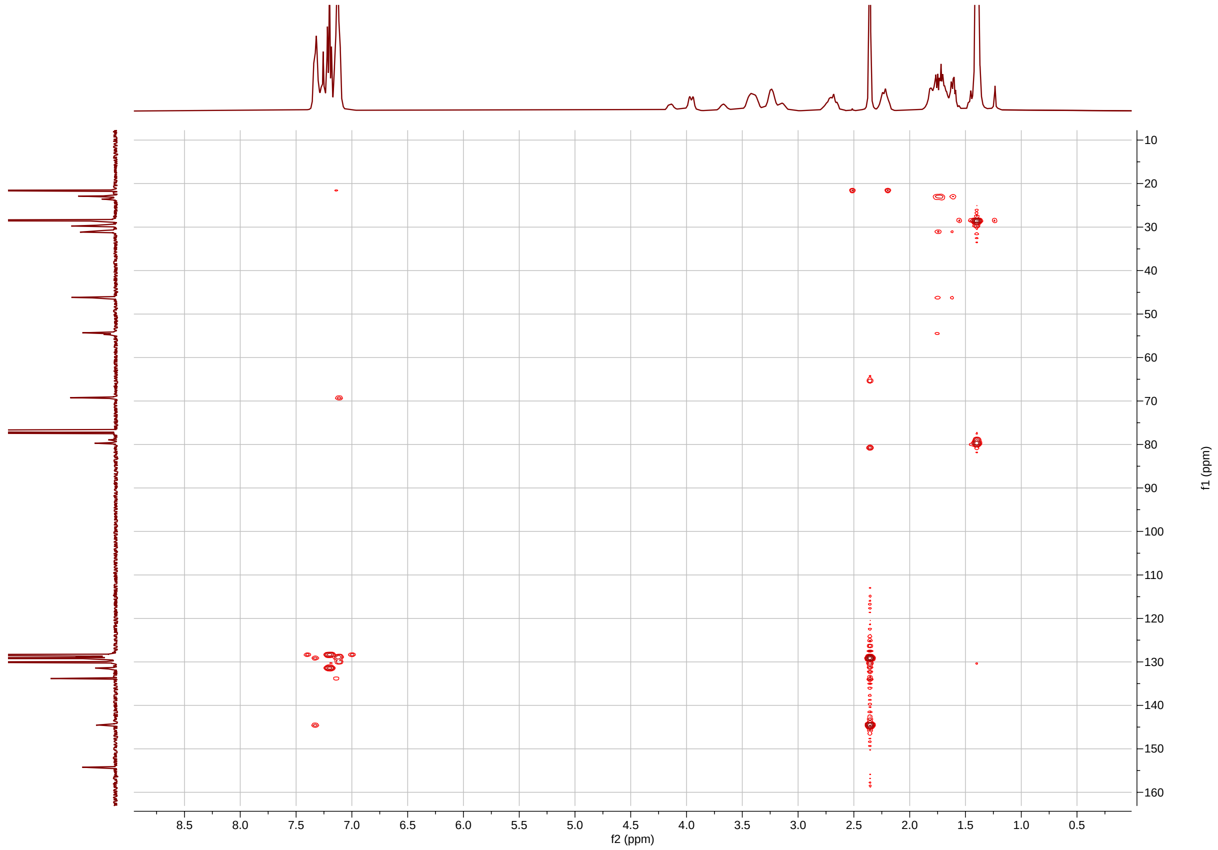

HMBC spectrum of compound 4a in $CDCl_3$

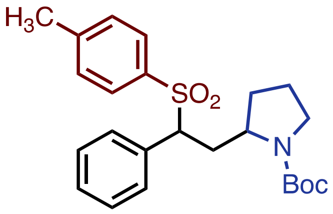

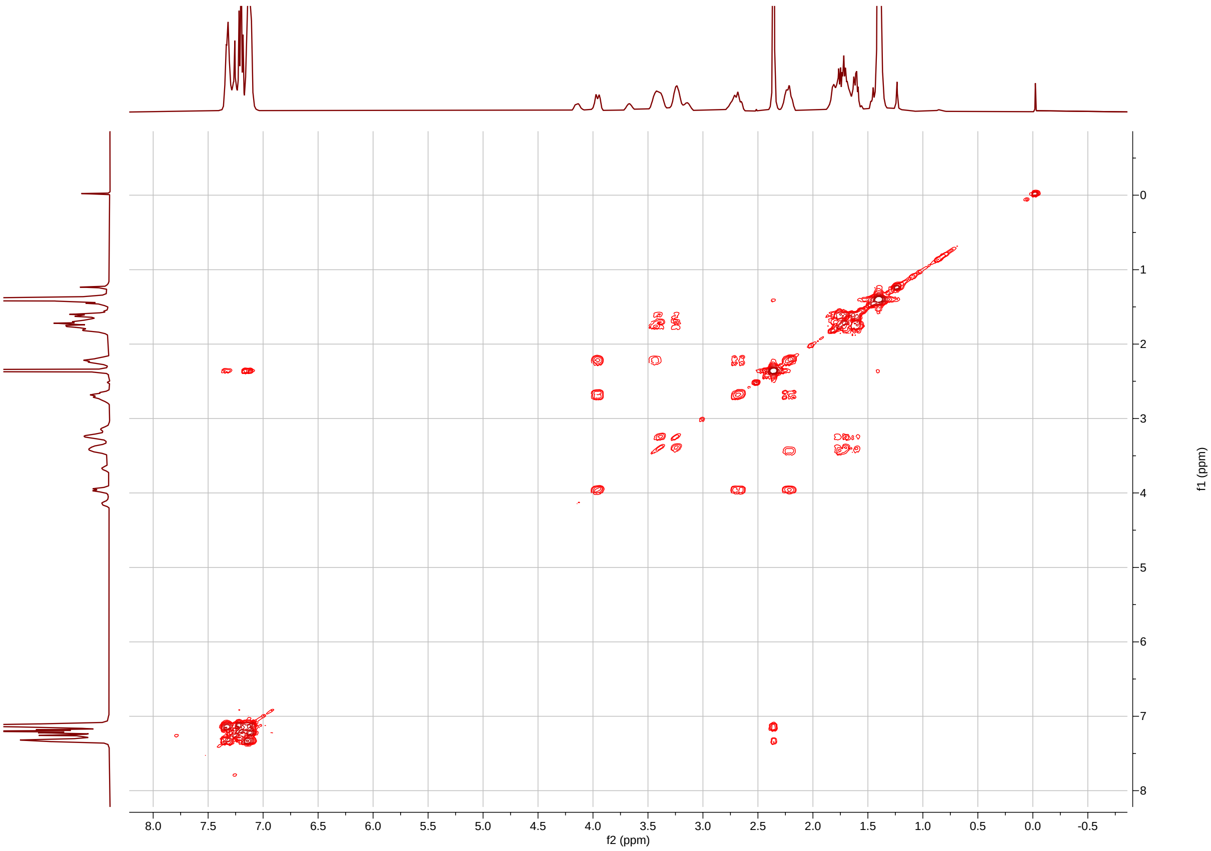

COSY spectrum of compound 4a in $CDCl_3$

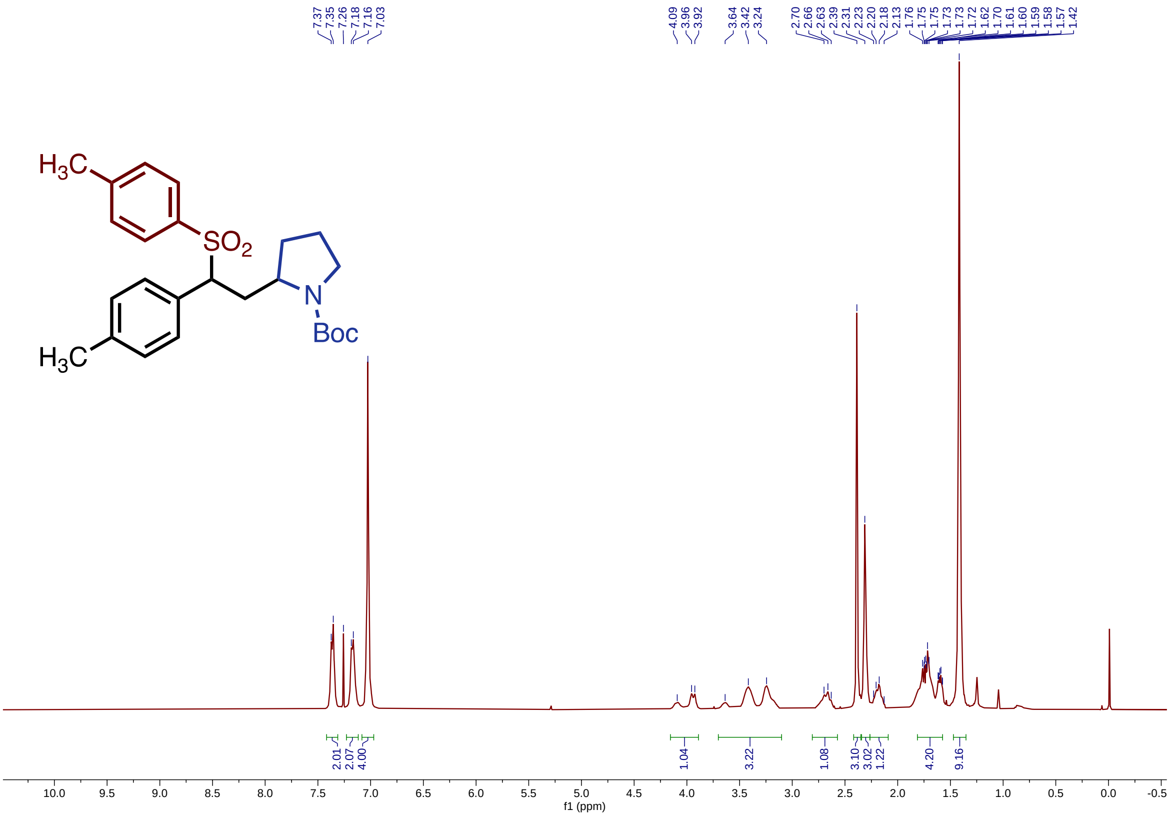

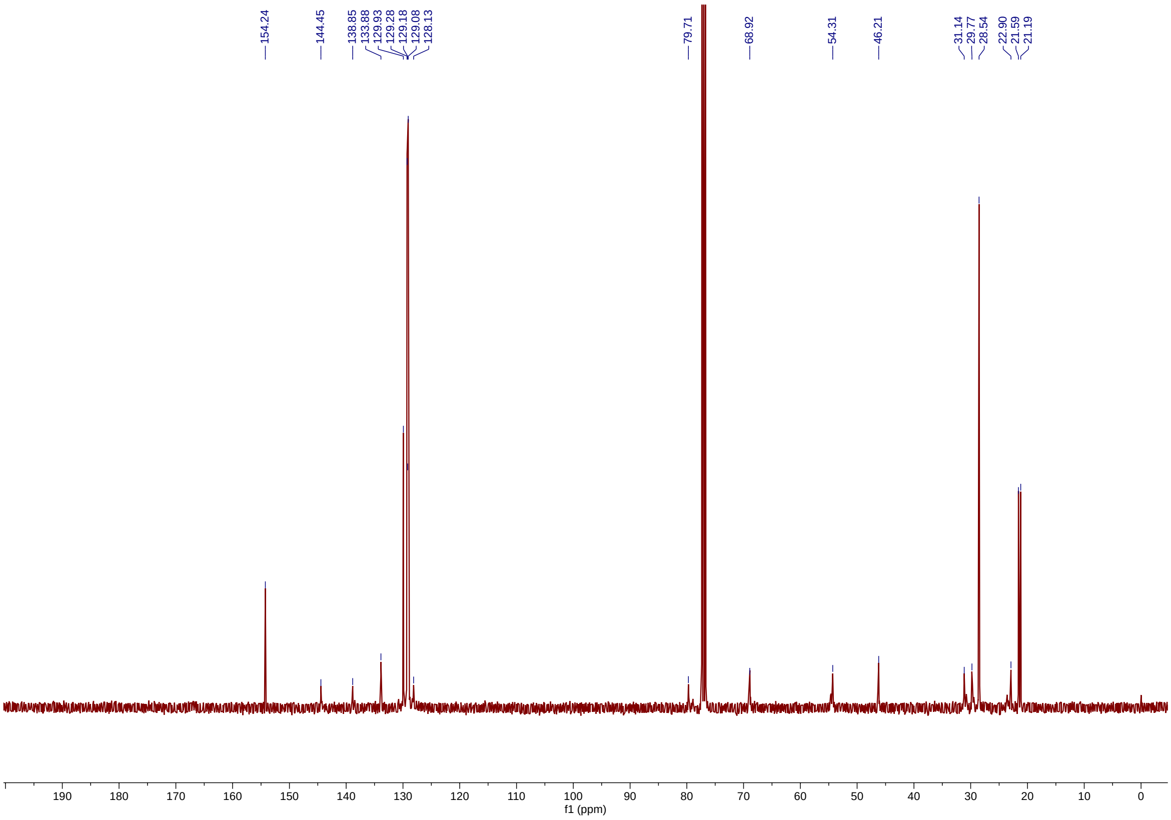

^{1}H (400 MHz) and ^{13}C (101 MHz) spectra of compound 4b in $CDCl_3$

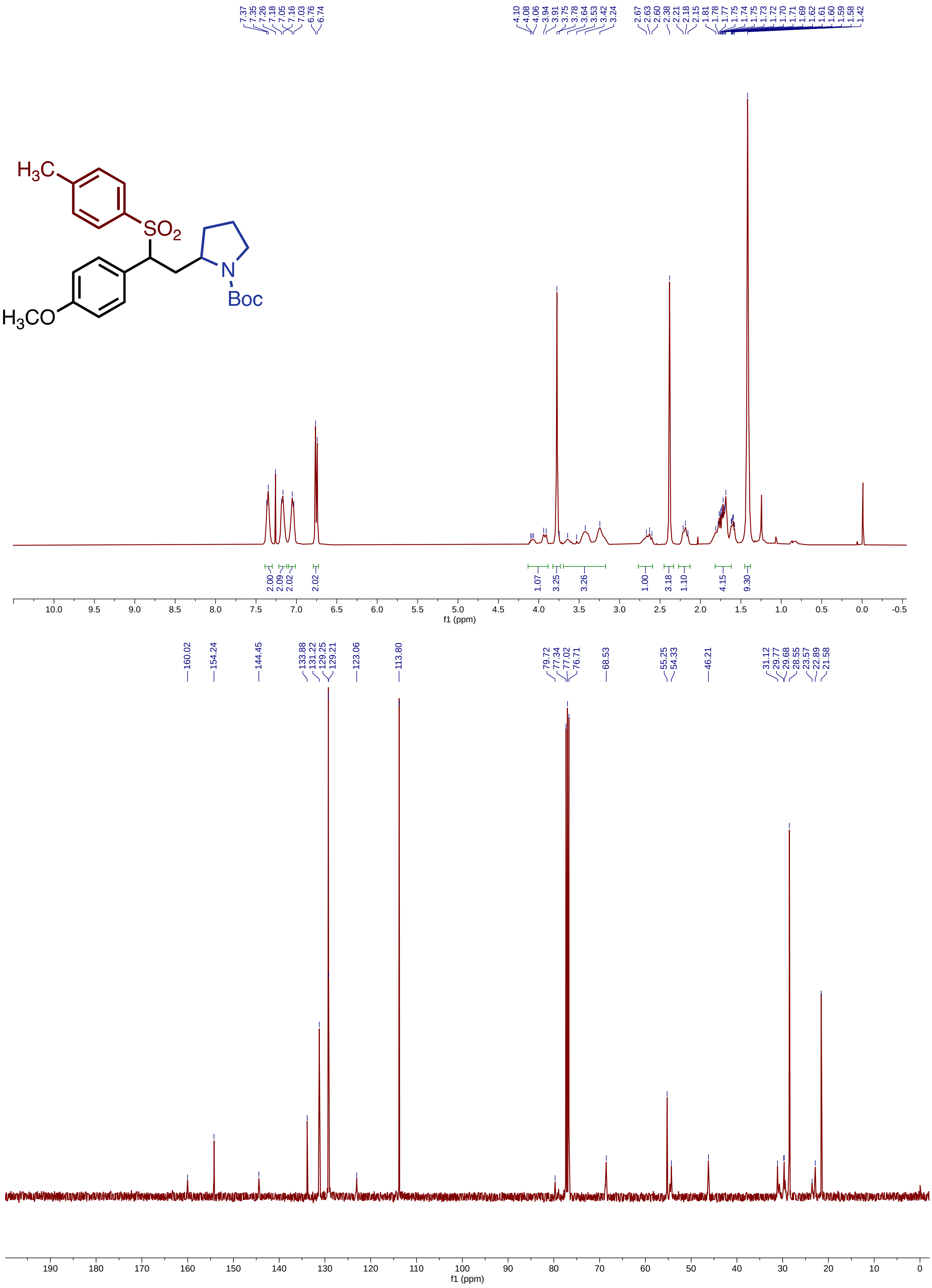

^{1}H (400 MHz) and ^{13}C (101 MHz) spectra of compound 4c in $CDCl_3$

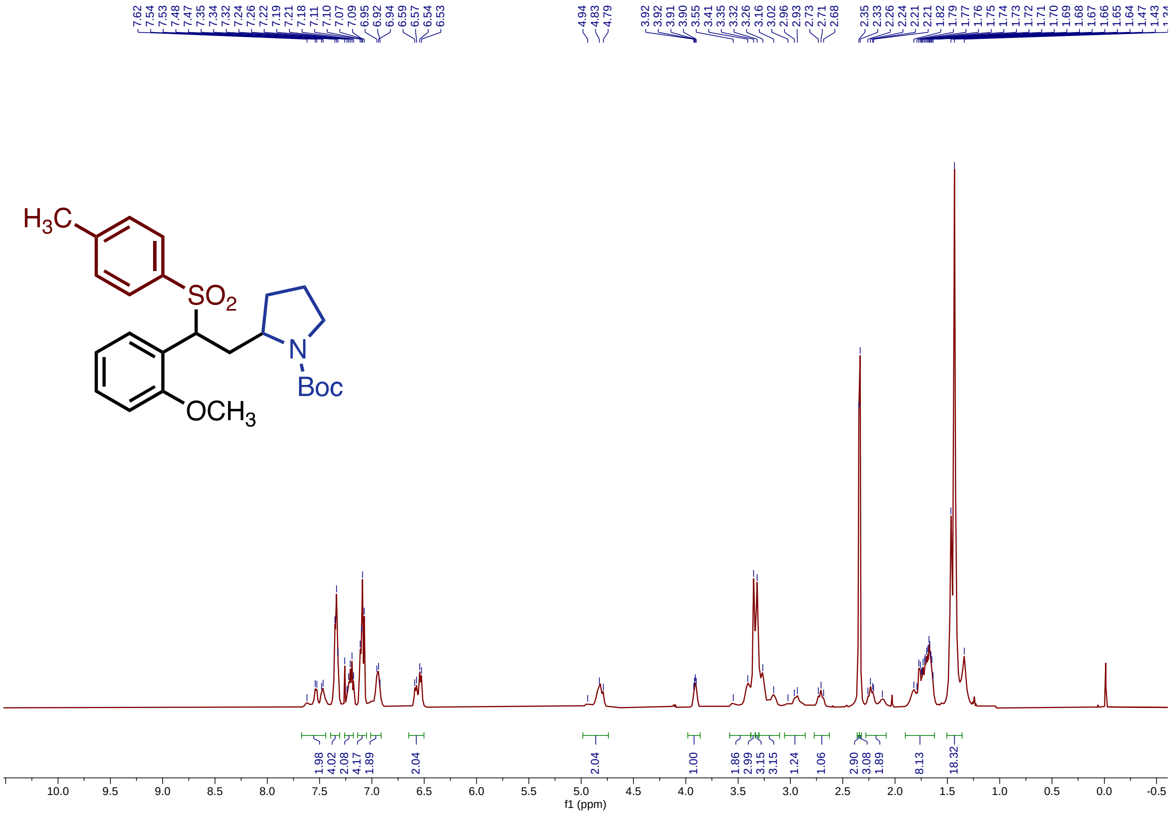

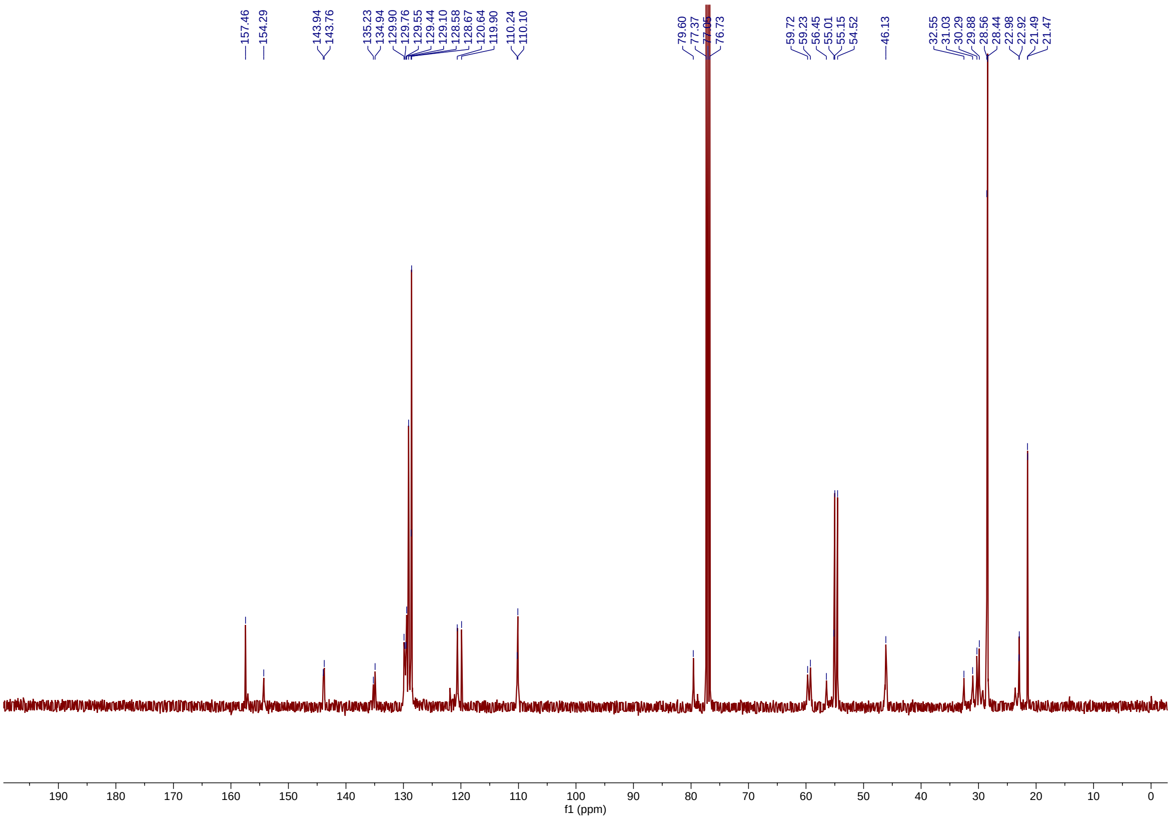

^{1}H (500 MHz) and ^{13}C (126 MHz) spectra of compound 4d in $CDCl_3$

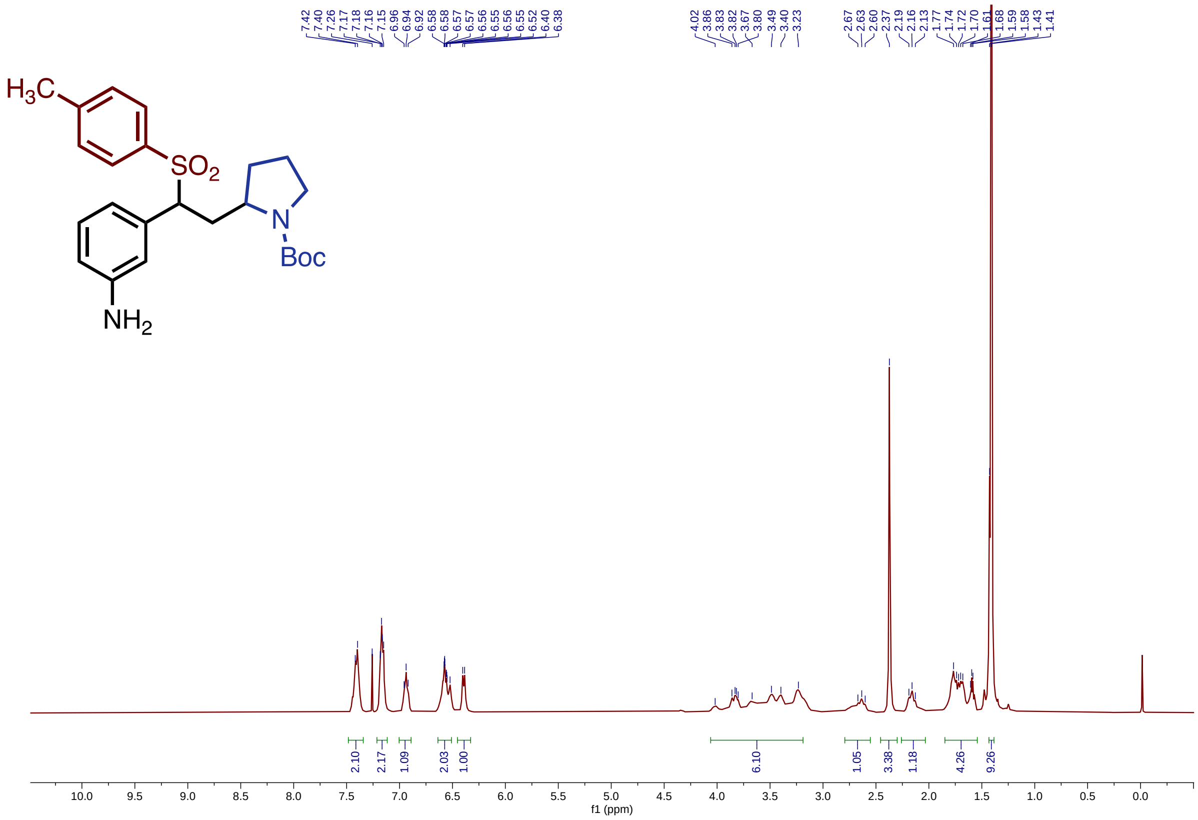

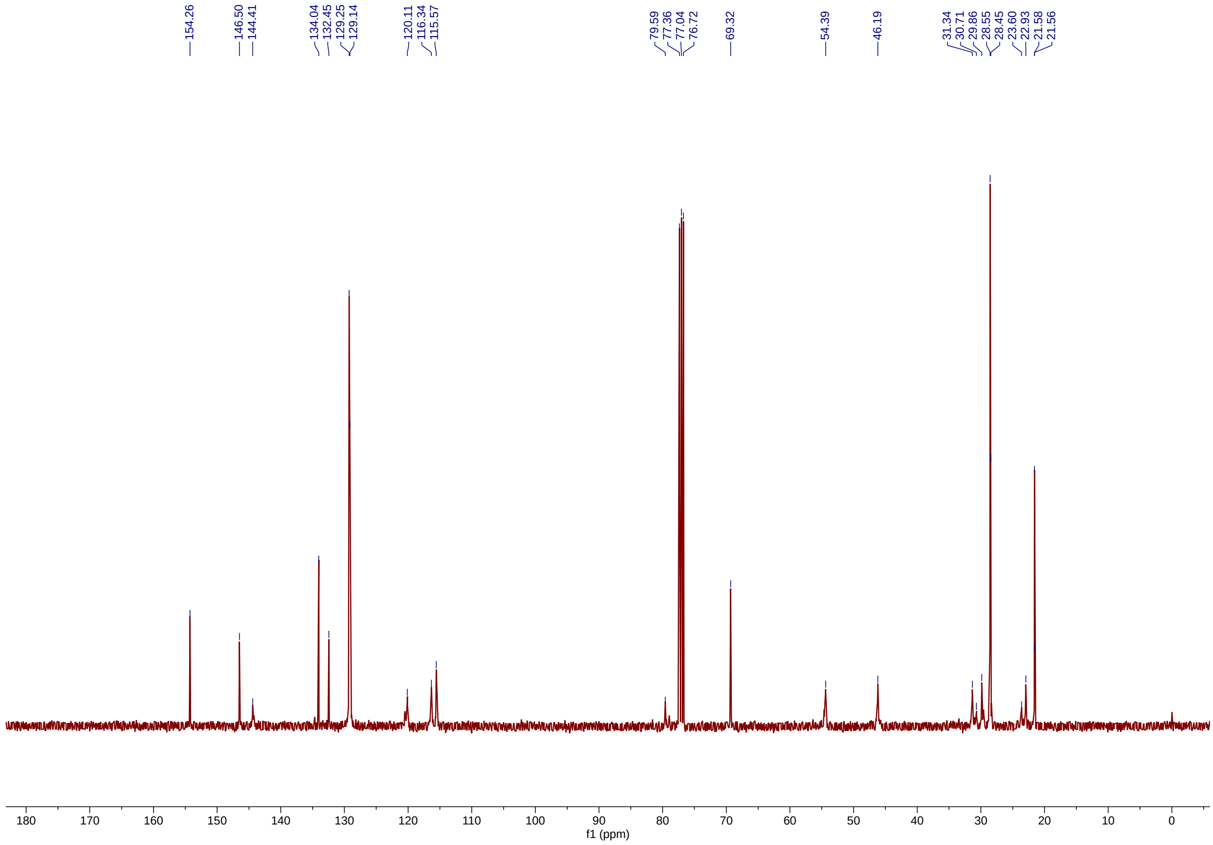

^{1}H (400 MHz) and ^{13}C (101 MHz) spectra of compound 4e in CDCl₃

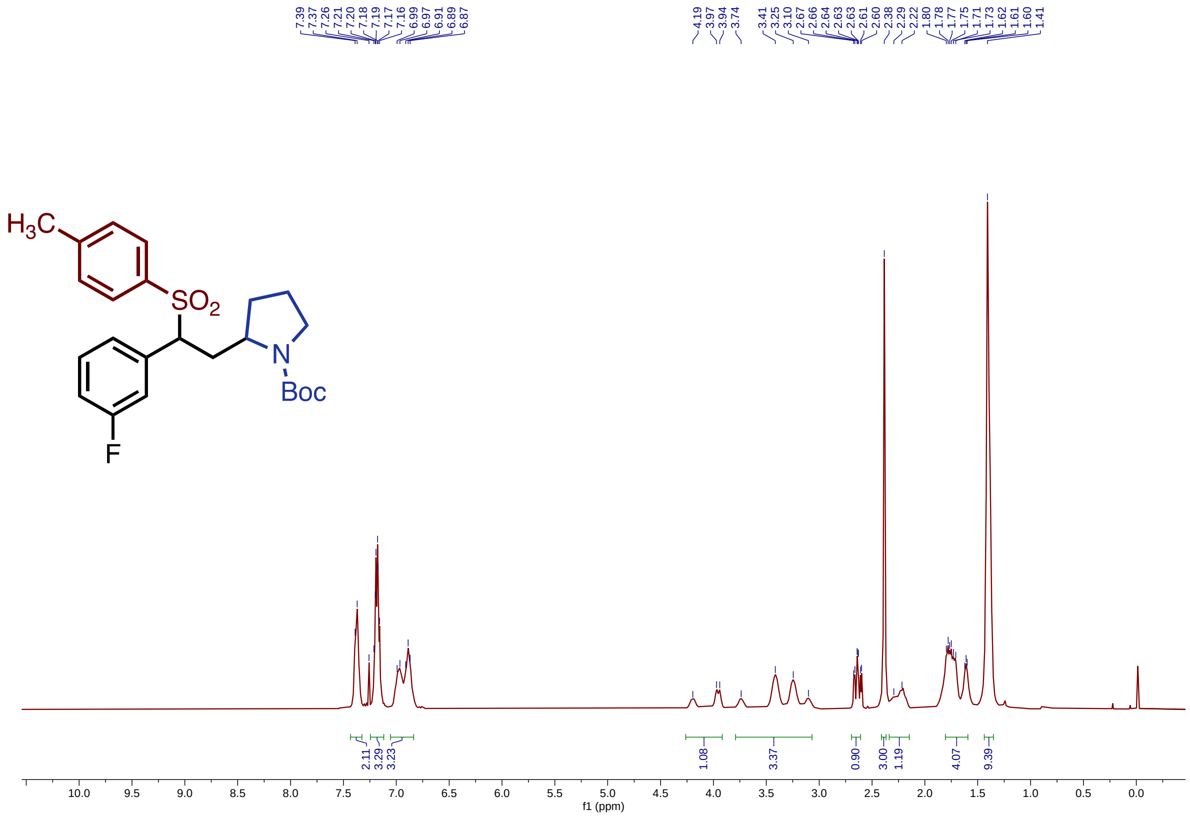

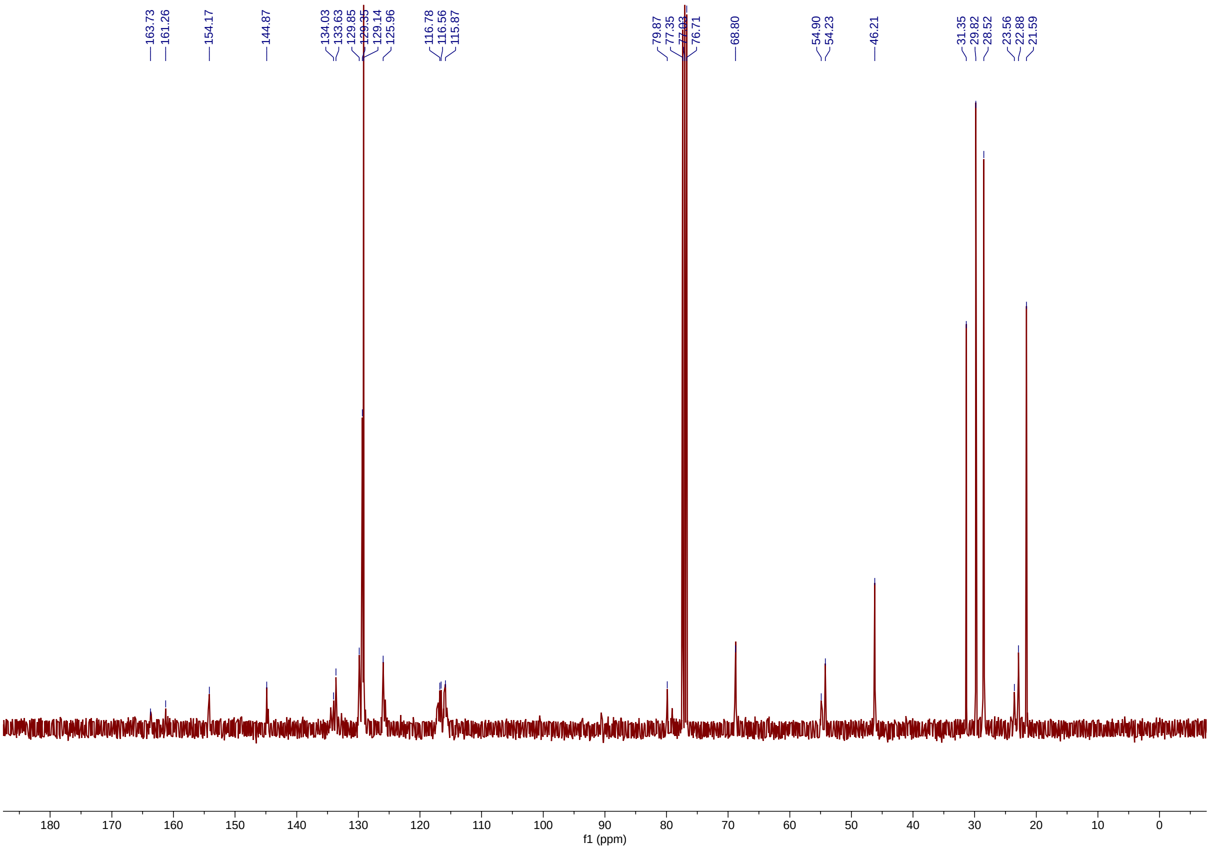

^{1}H (400 MHz) and ^{13}C (101 MHz) spectra of compound 4f in $CDCl_3$

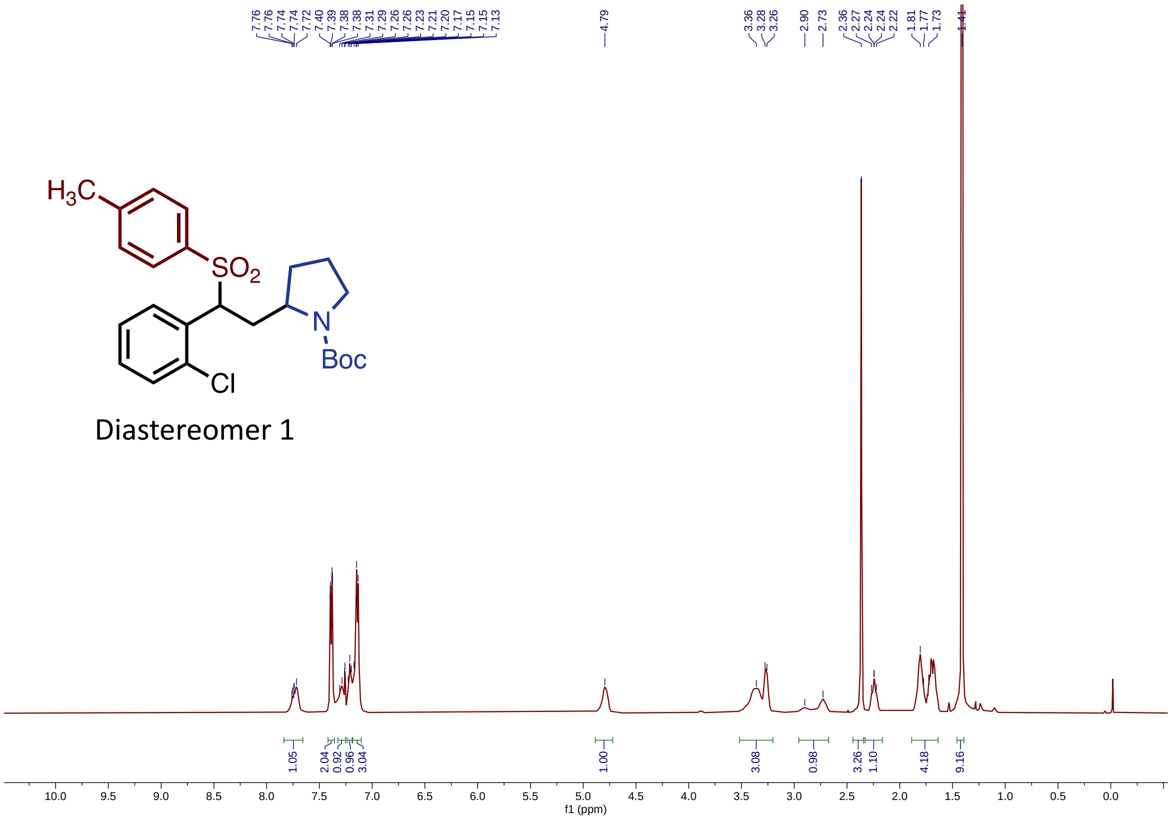

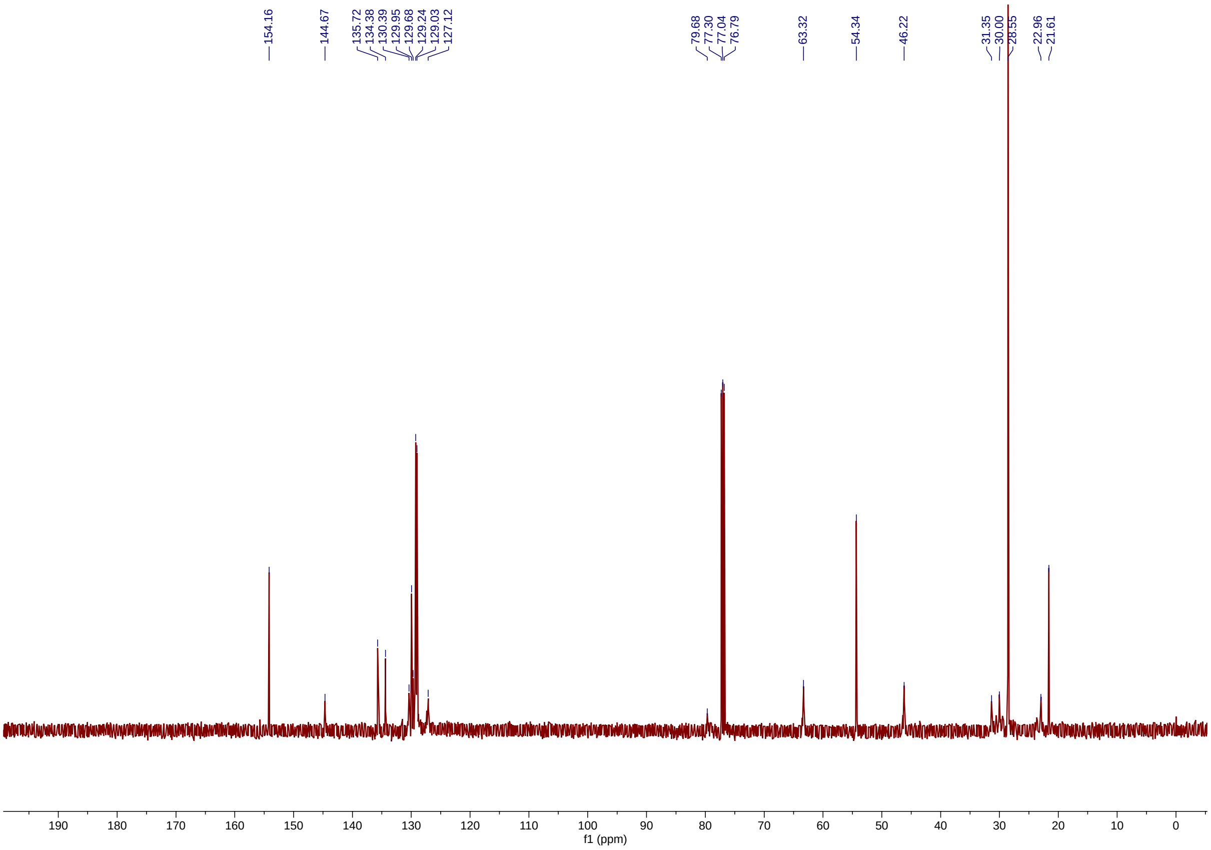

^{1}H (500 MHz) and ^{13}C (126 MHz) spectra of compound 4g in $CDCl_3$

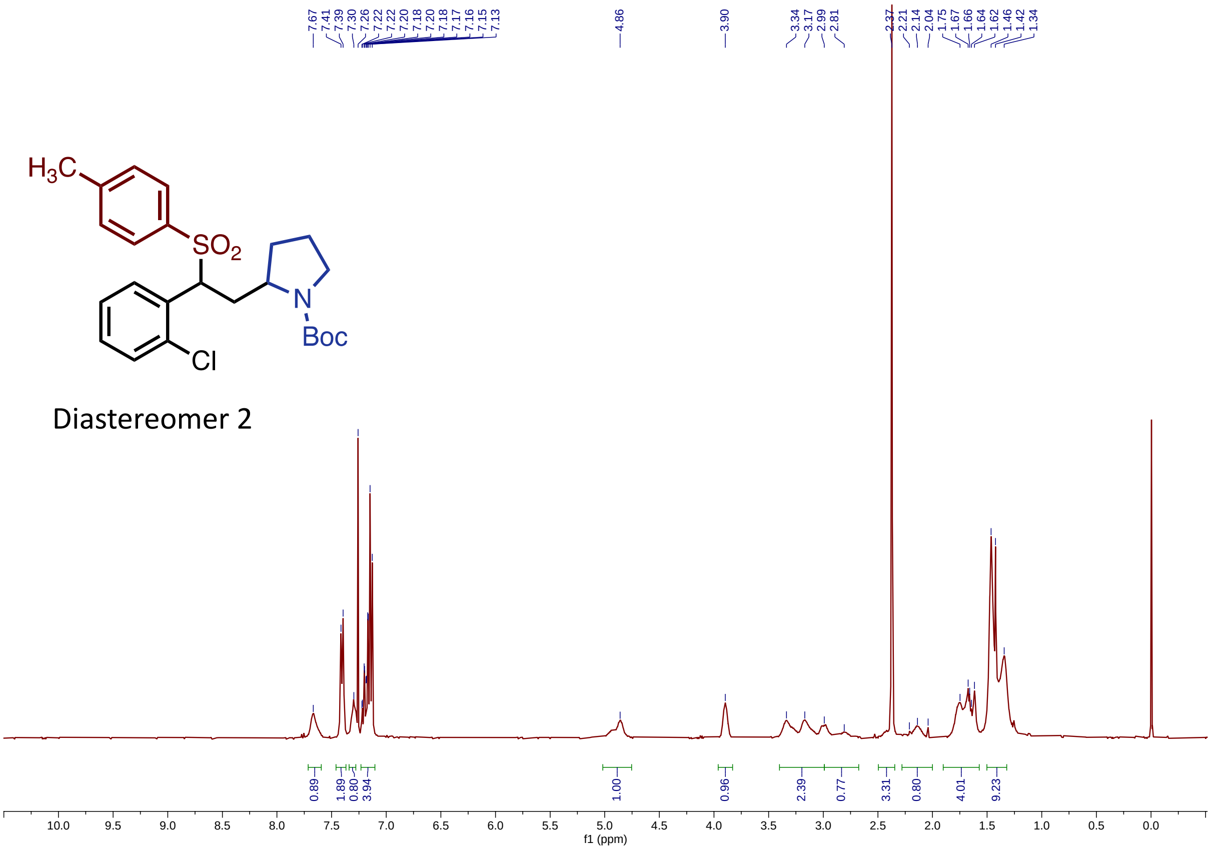

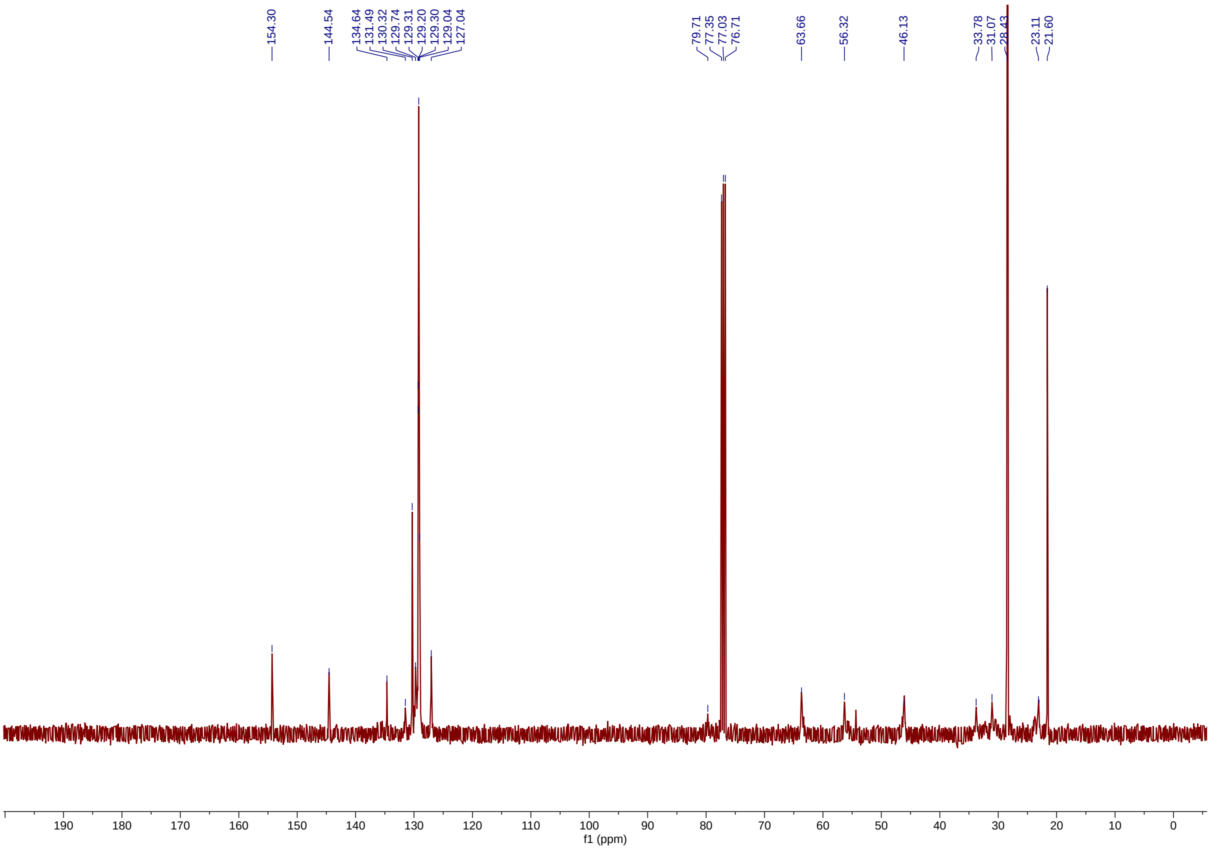

^{1}H (400 MHz) and ^{13}C (101 MHz) spectra of compound 4g in $CDCl_3$

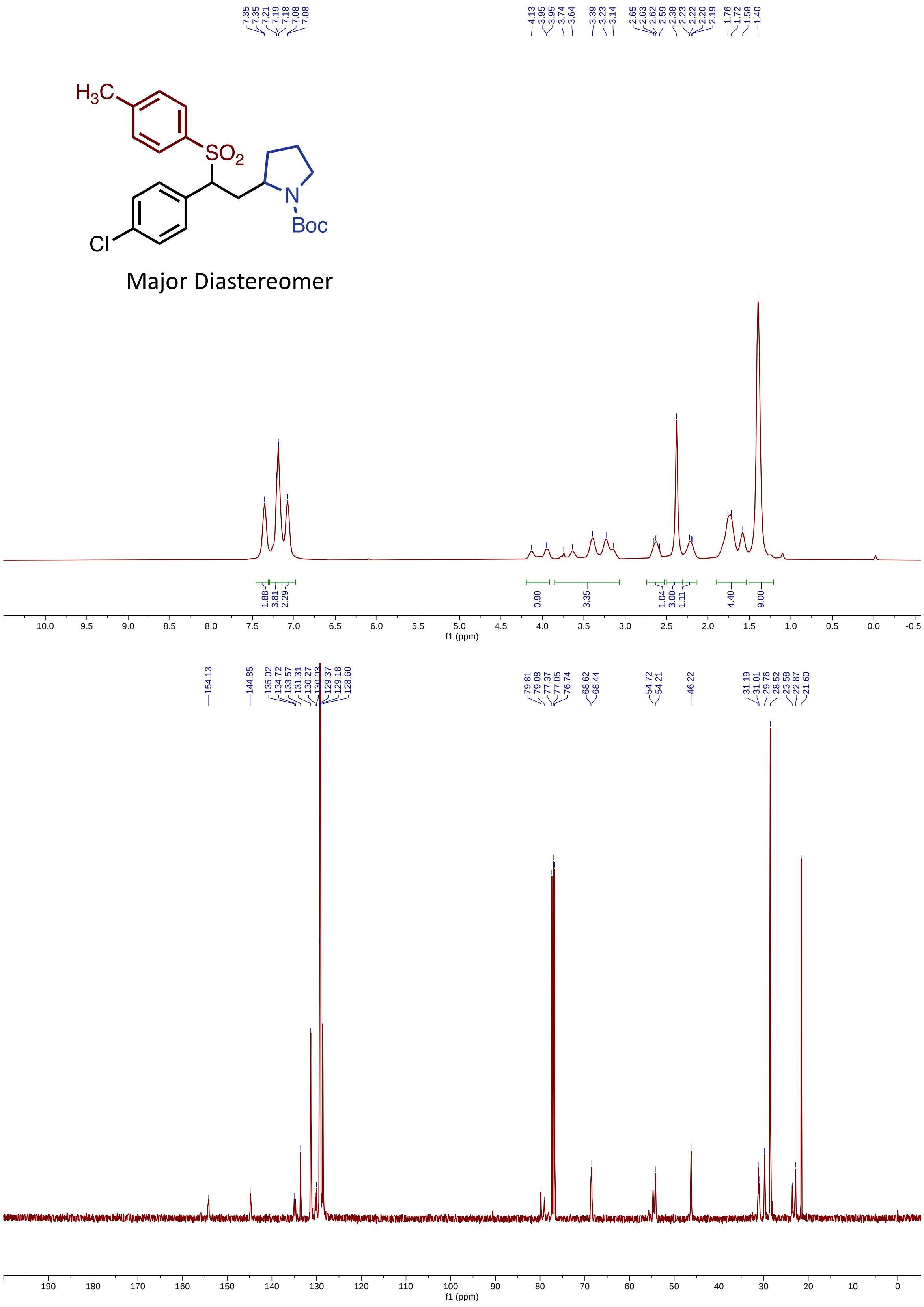

^{1}H (400 MHz) and ^{13}C (101 MHz) spectra of compound 4h in $CDCl_3$

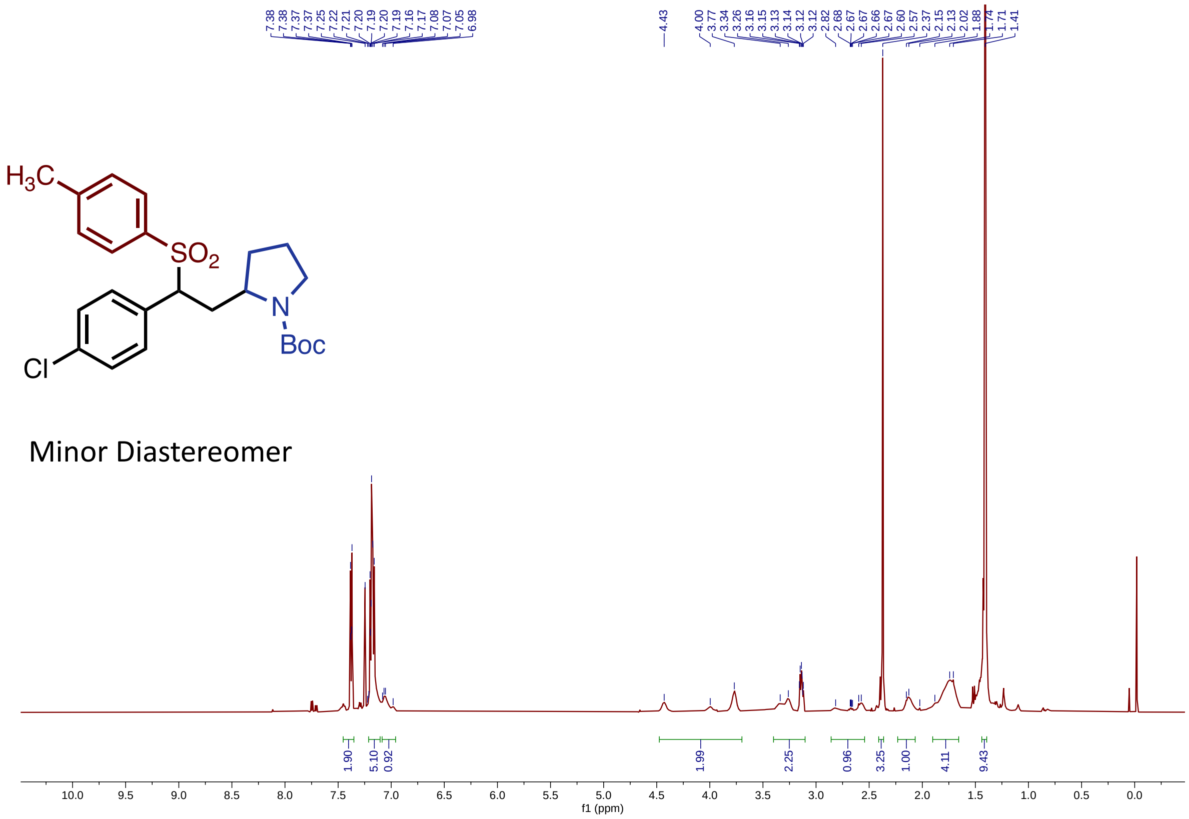

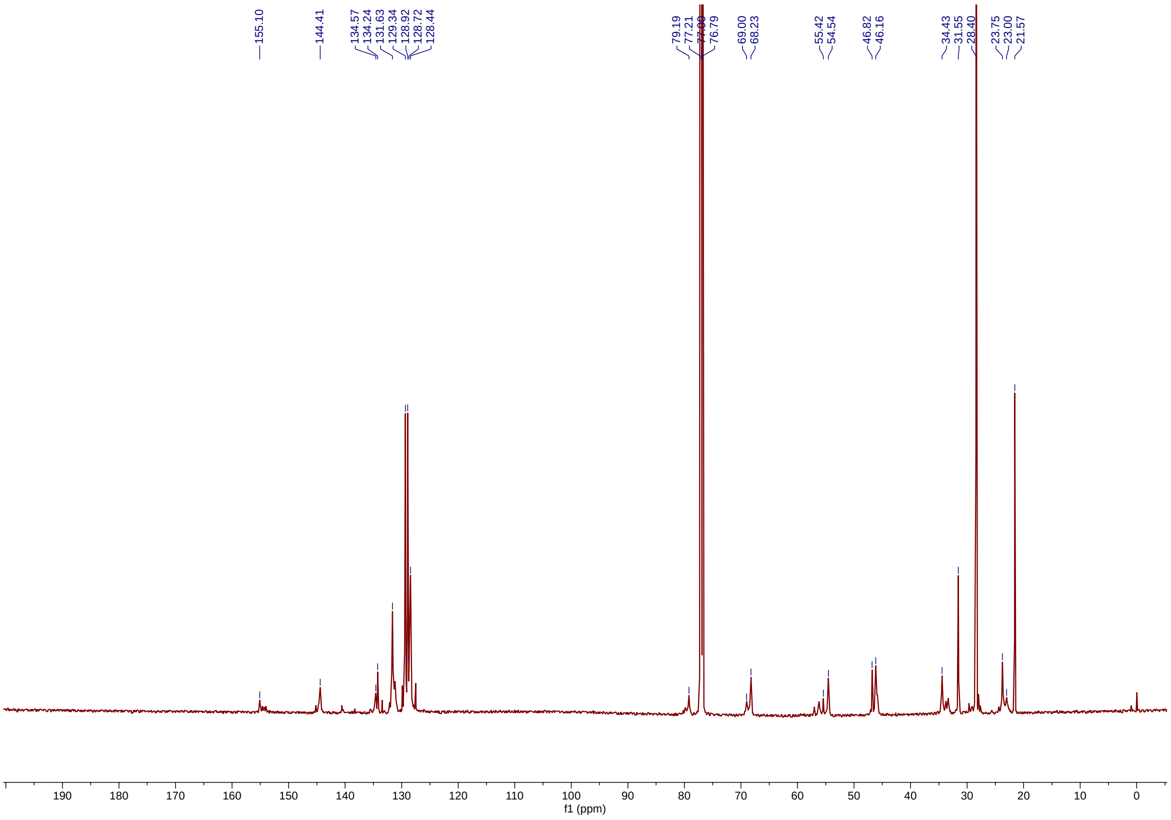

^{1}H (600 MHz) and ^{13}C (151 MHz) spectra of compound 4h in $CDCl_3$

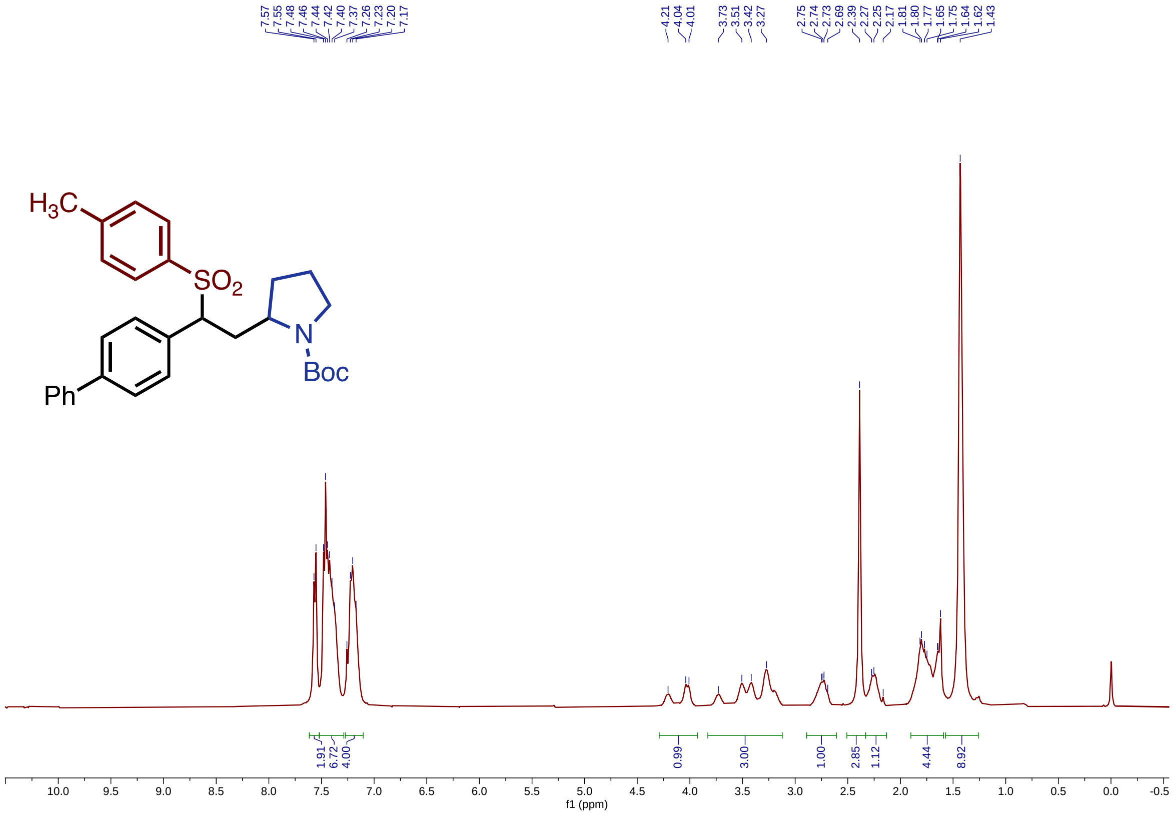

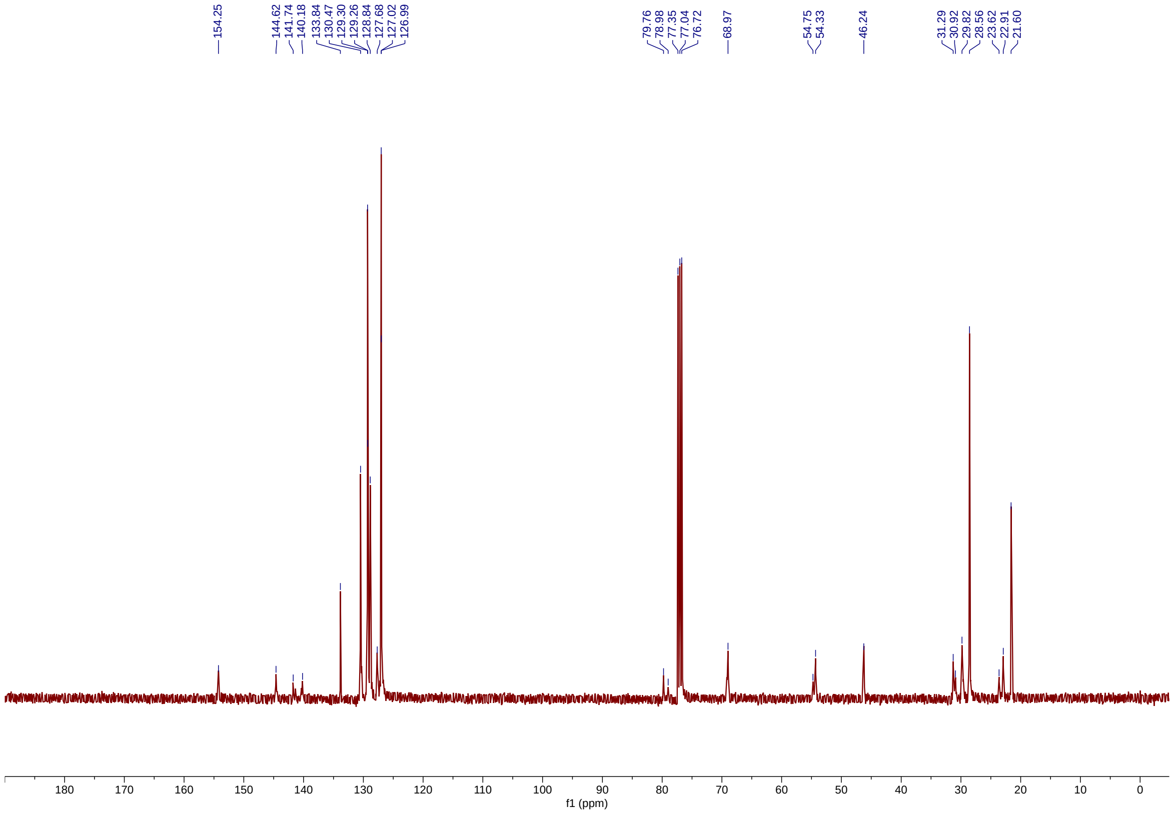

^{1}H (400 MHz) and ^{13}C (101 MHz) spectra of compound 4i in $CDCl_3$

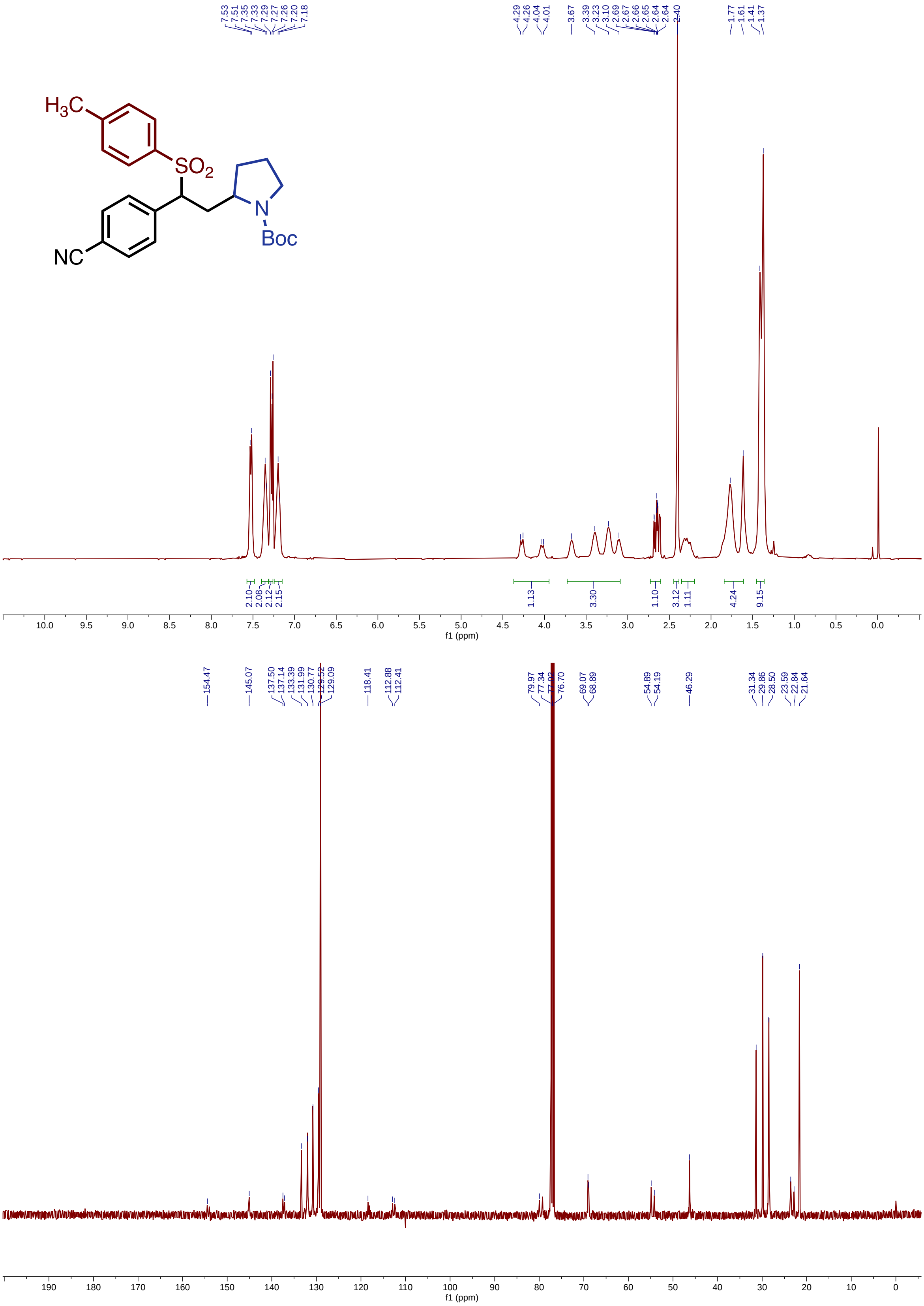

^{1}H (400 MHz) and ^{13}C (101 MHz) spectra of compound 4j in $CDCl_3$

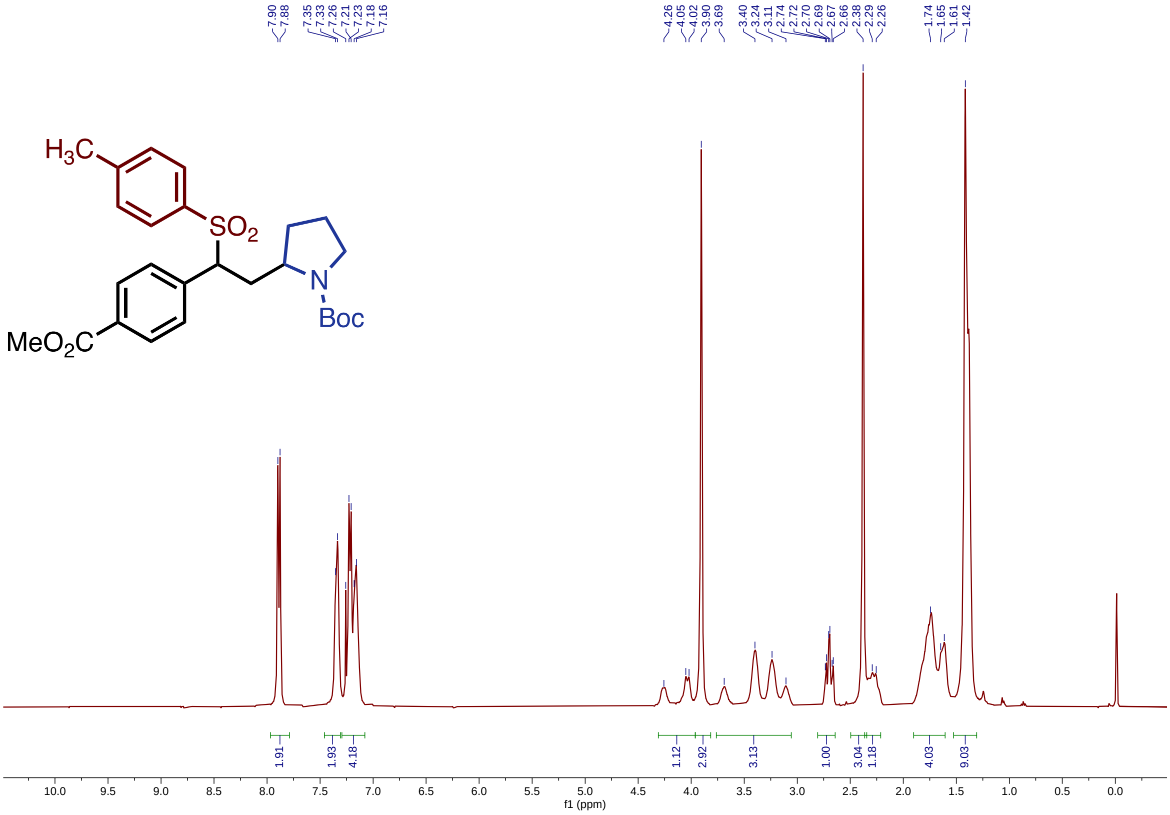

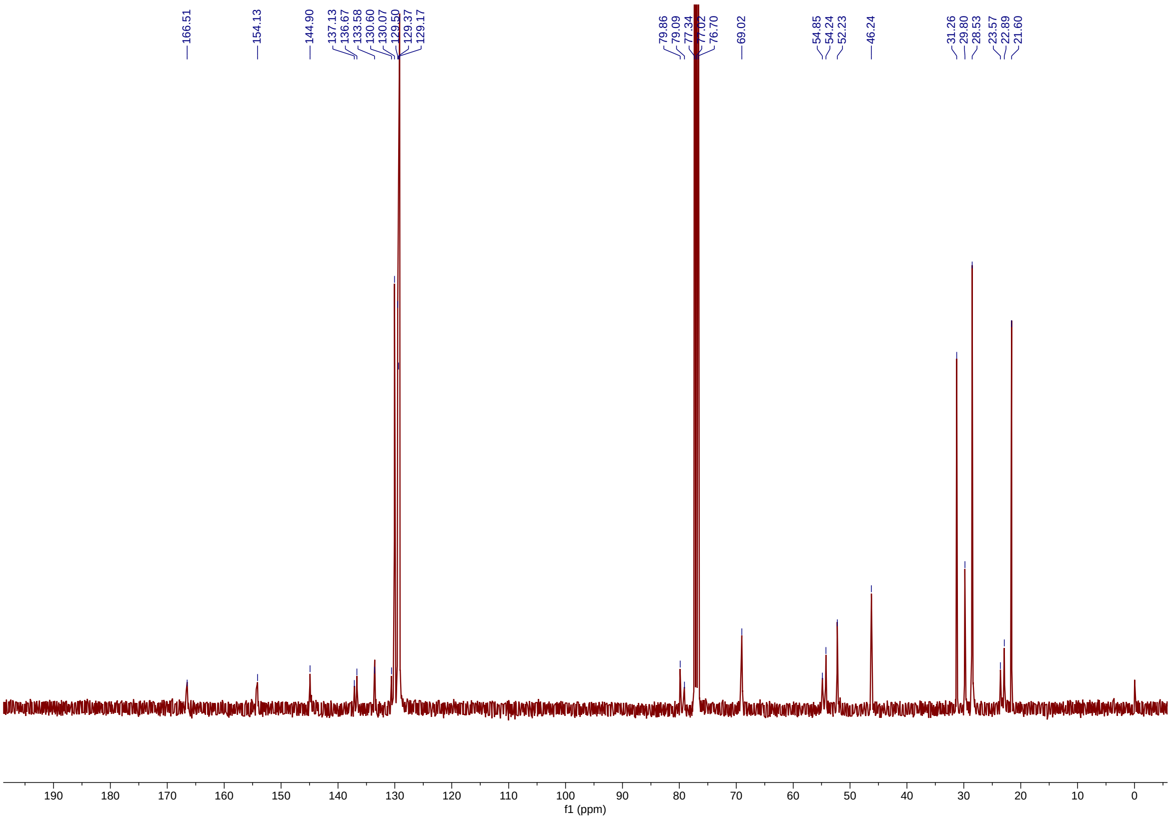

^{1}H (400 MHz) and ^{13}C (101 MHz) spectra of compound 4k in $CDCl_3$

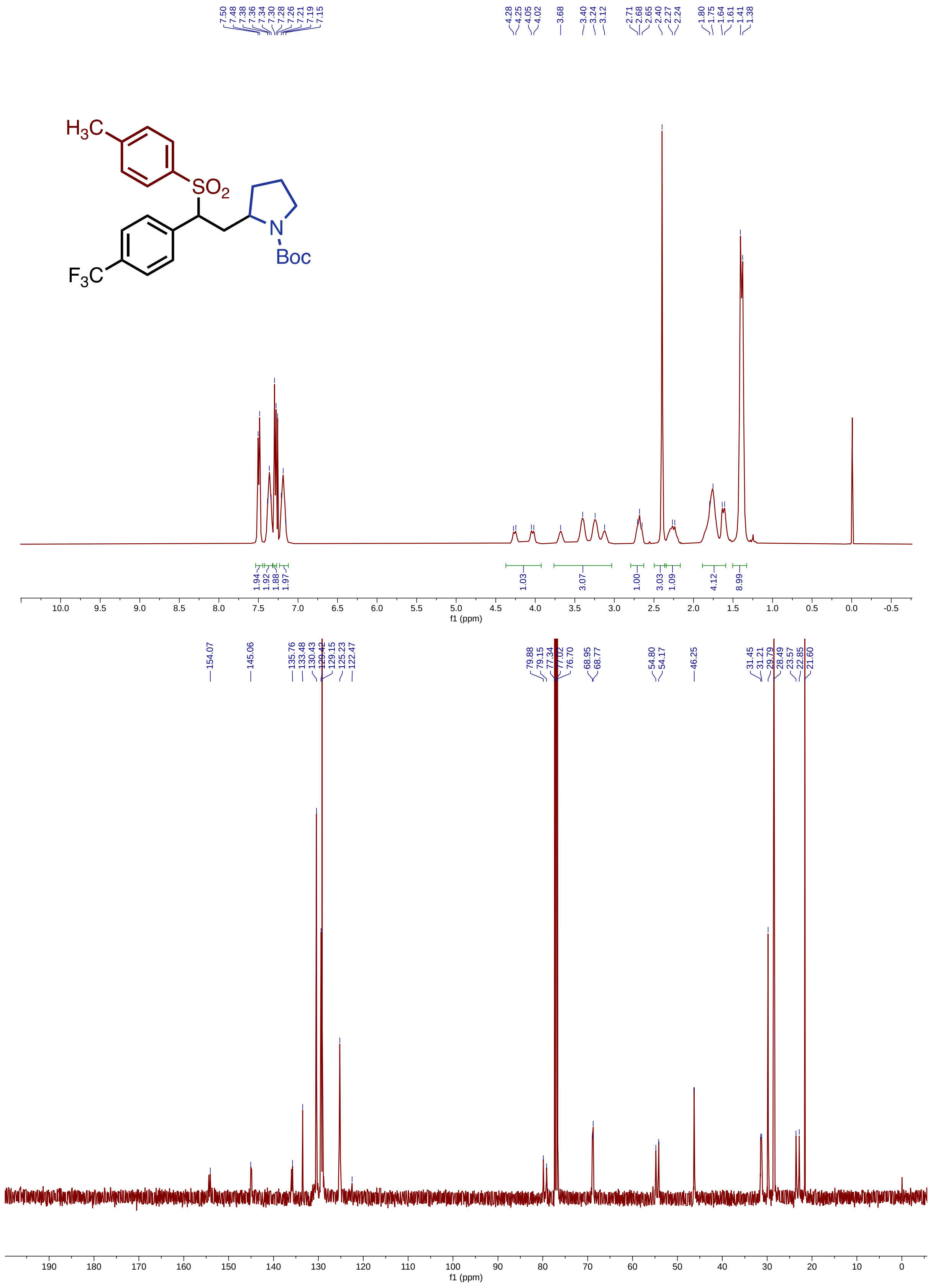

^{1}H (400 MHz) and ^{13}C (101 MHz) spectra of compound 4l in $CDCl_3$

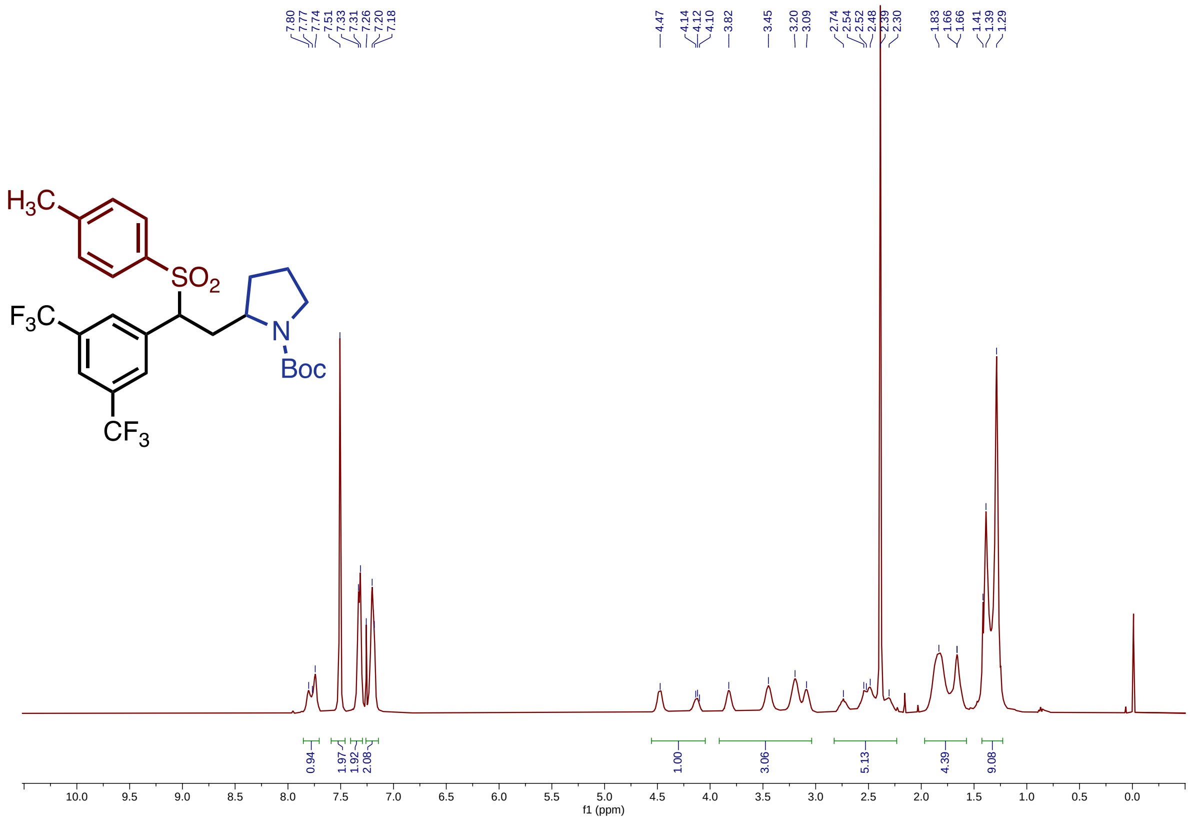

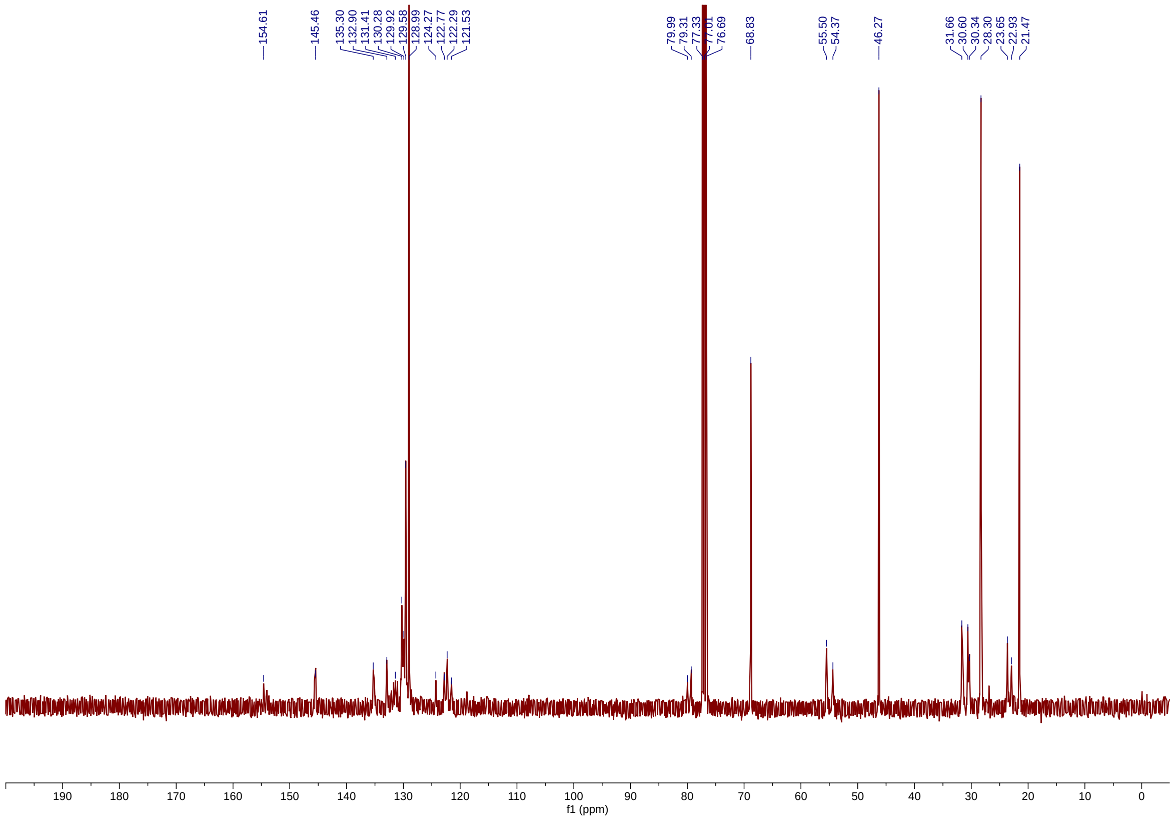

^{1}H (400 MHz) and ^{13}C (101 MHz) spectra of compound 4m in $CDCl_3$

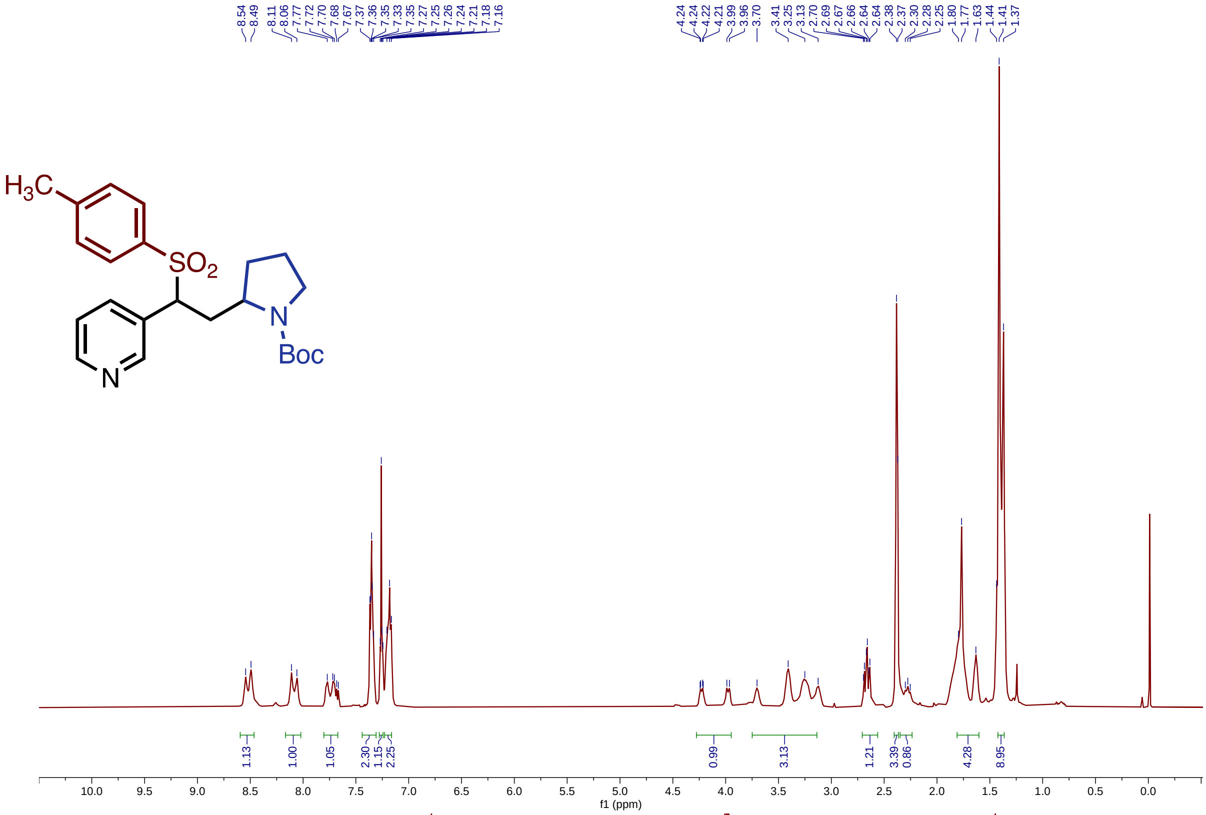

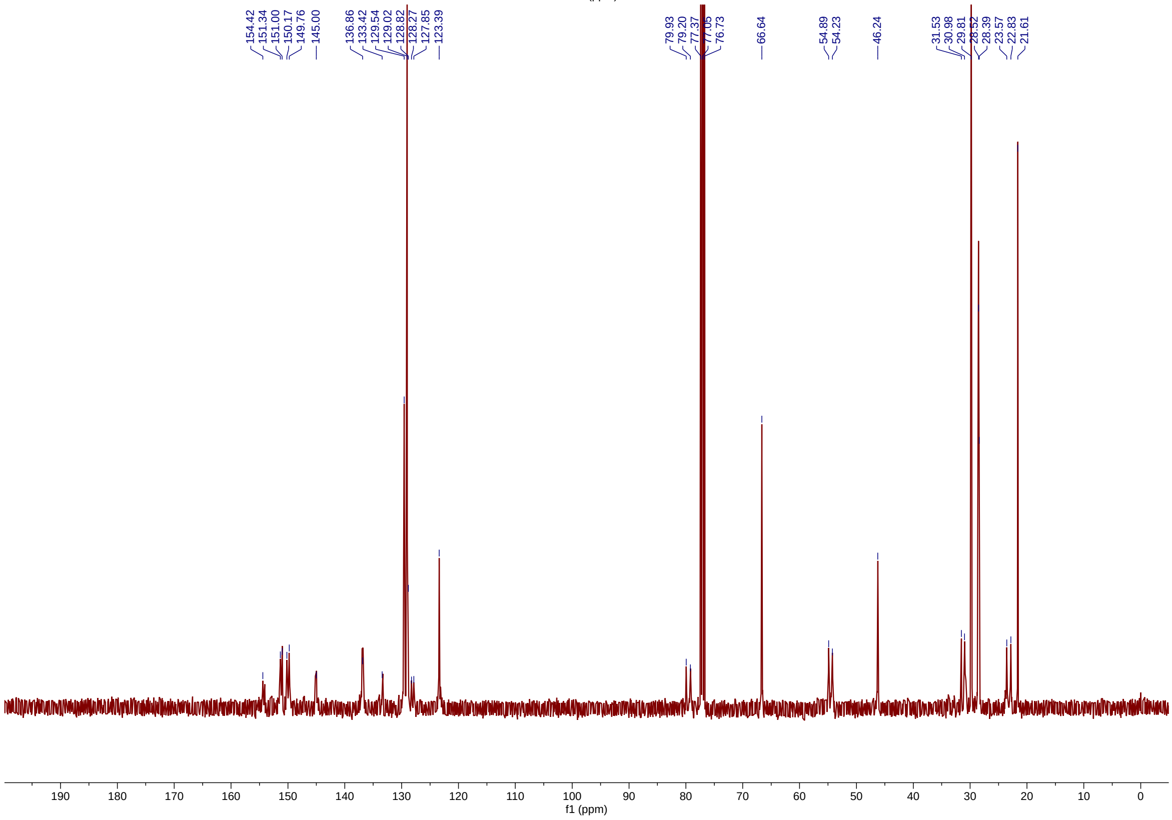

^{1}H (500 MHz) and ^{13}C (101 MHz) spectra of compound 4p in $CDCl_3$

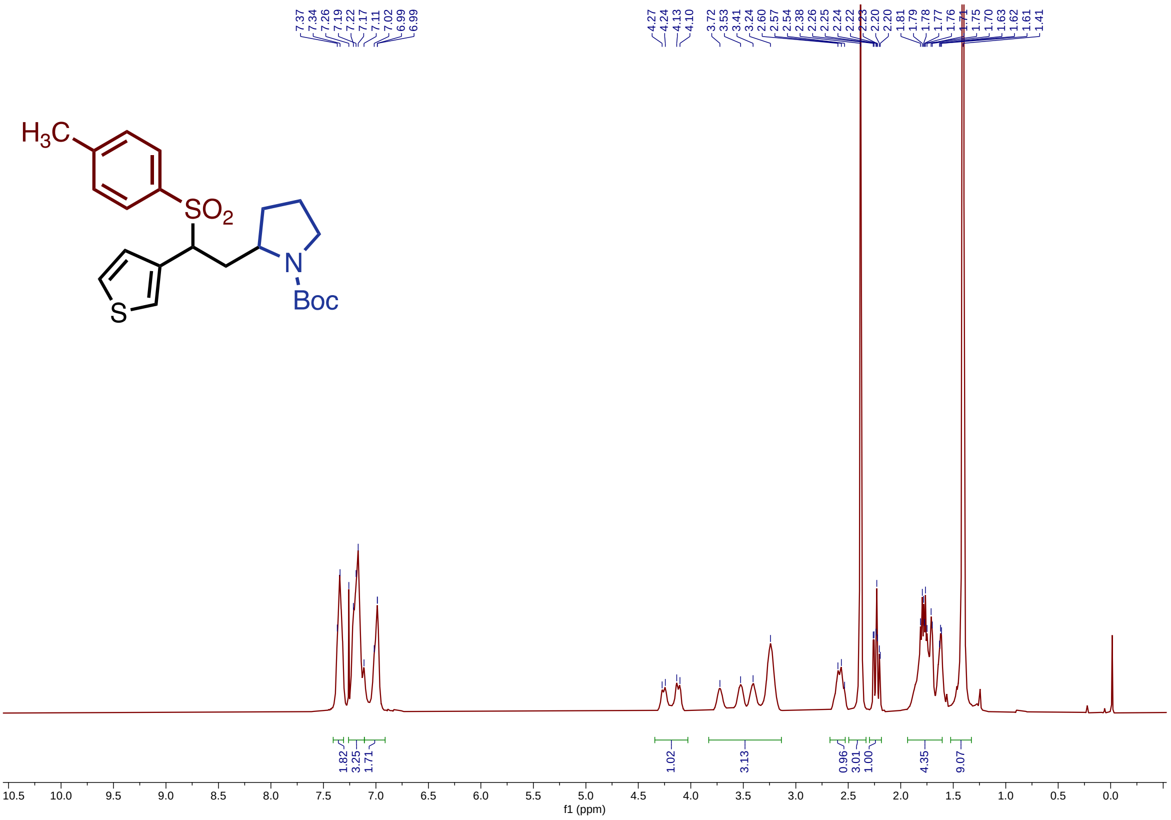

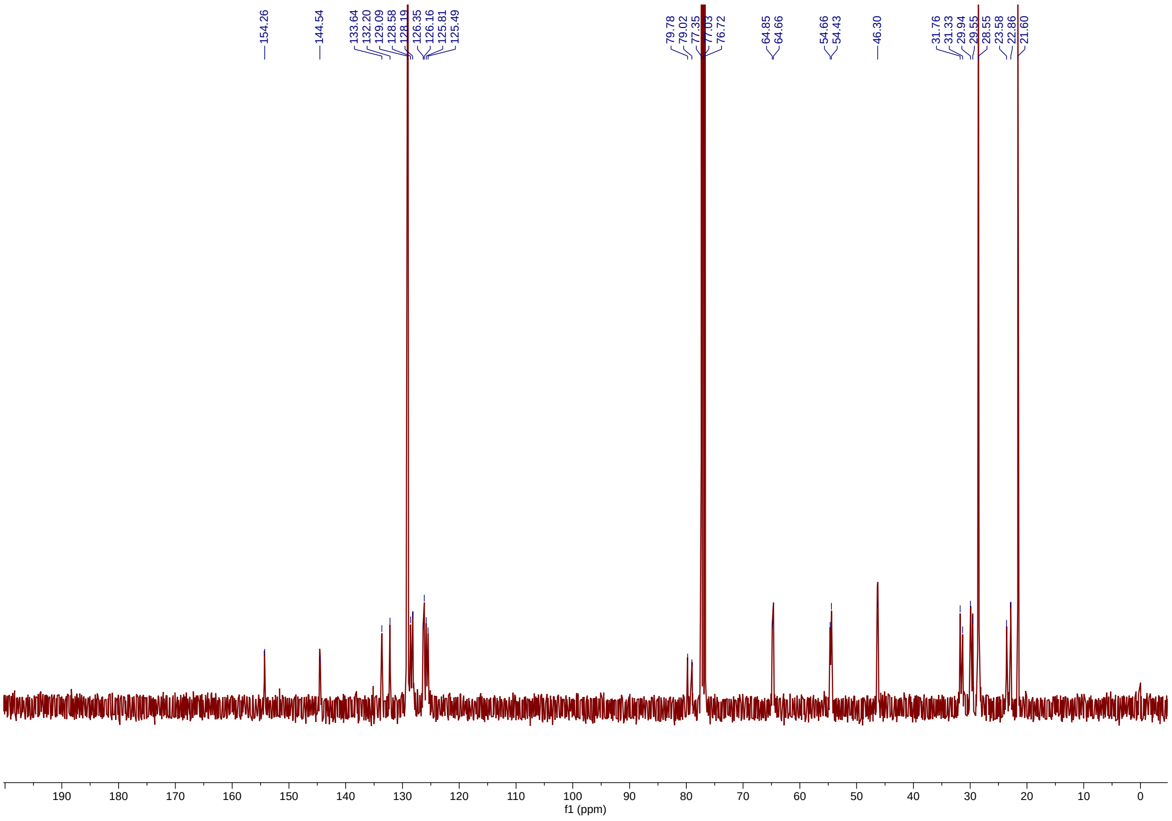

^{1}H (400 MHz) and ^{13}C (101 MHz) spectra of compound 4q in $CDCl_3$

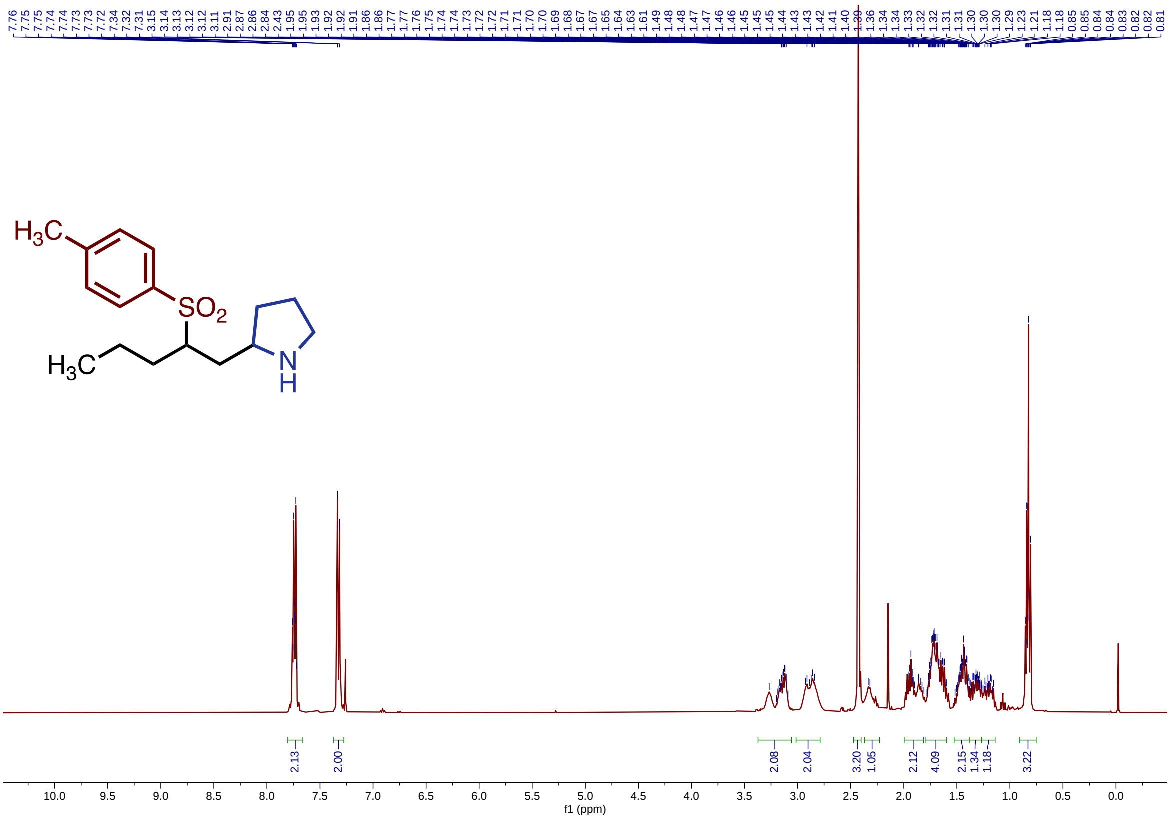

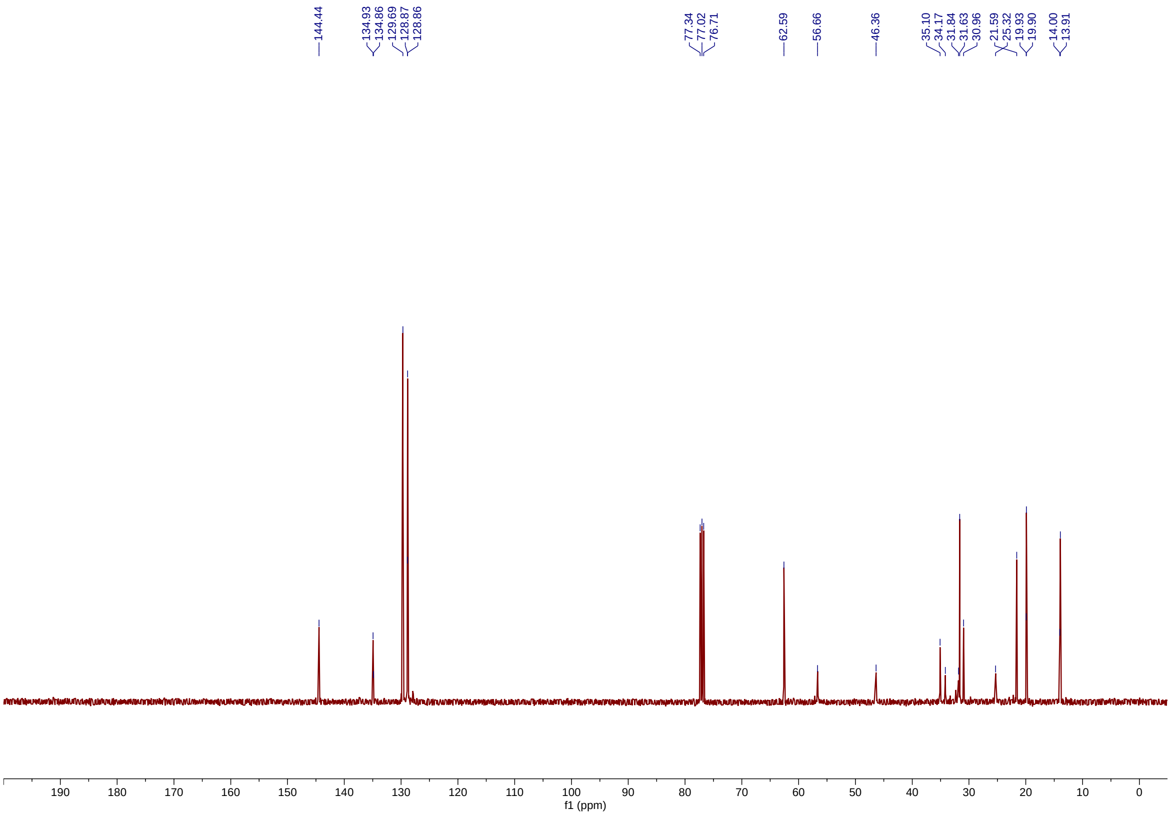

^{1}H (400 MHz) and ^{13}C (101 MHz) spectra of compound 4r in $CDCl_3$

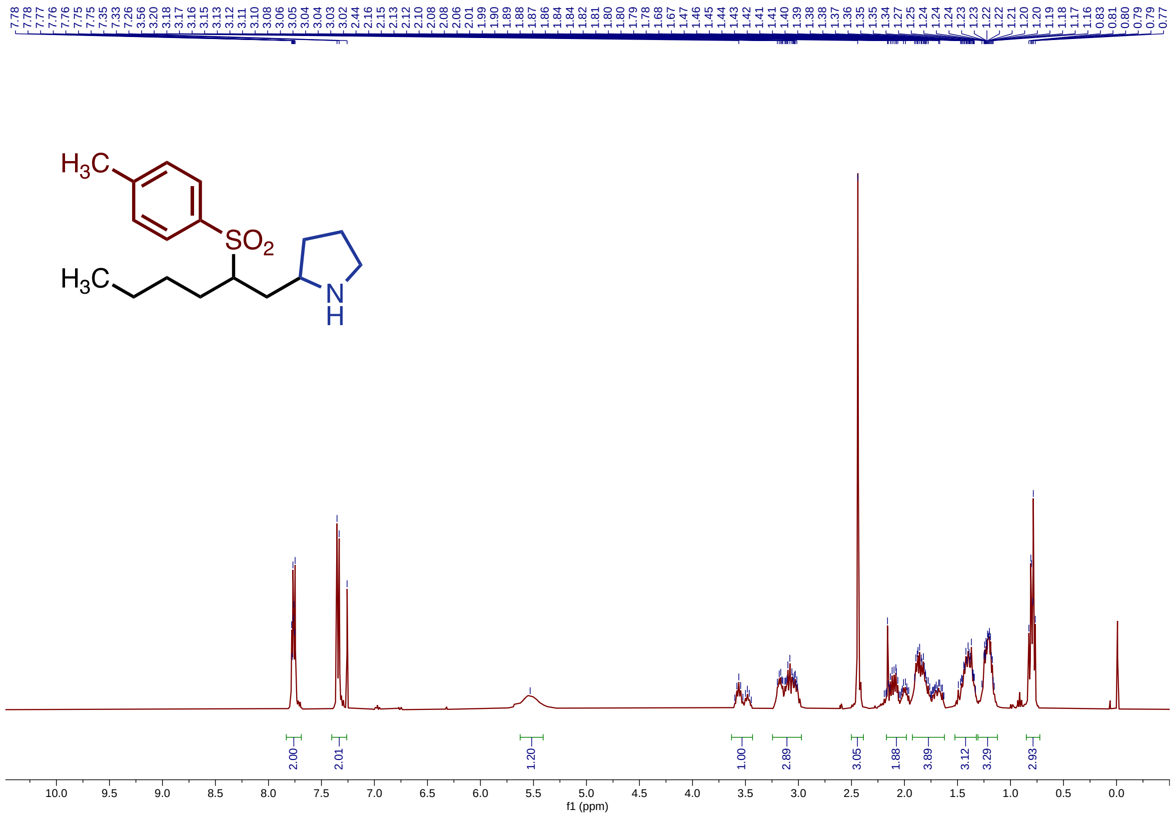

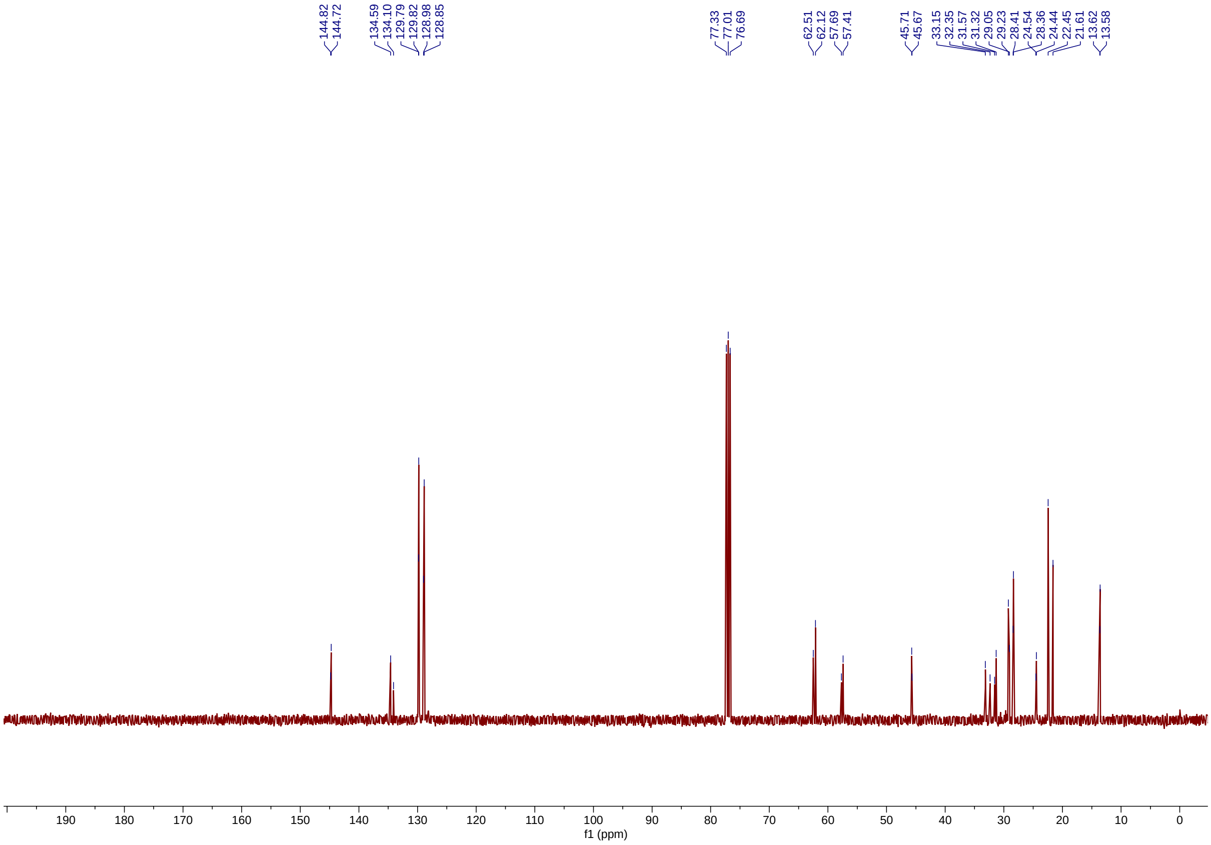

^{1}H (400 MHz) and ^{13}C (101 MHz) spectra of compound 4s in $CDCl_3$

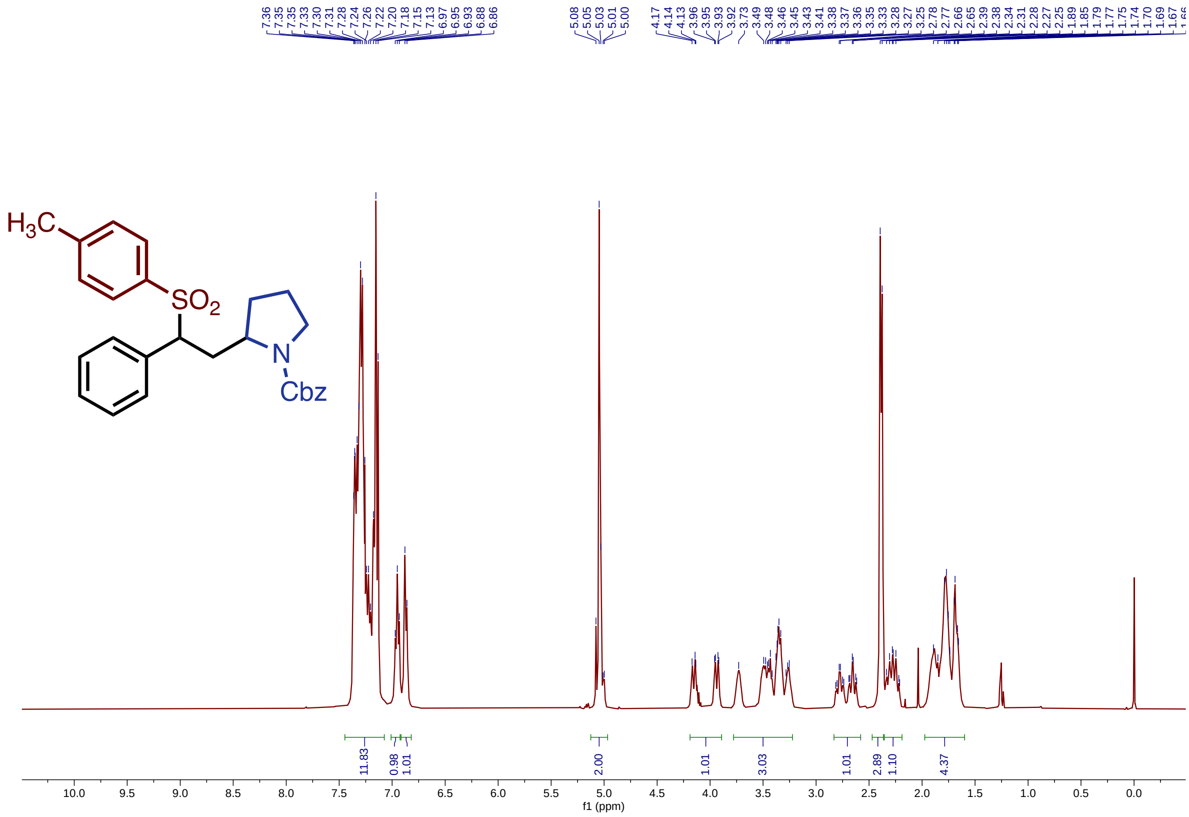

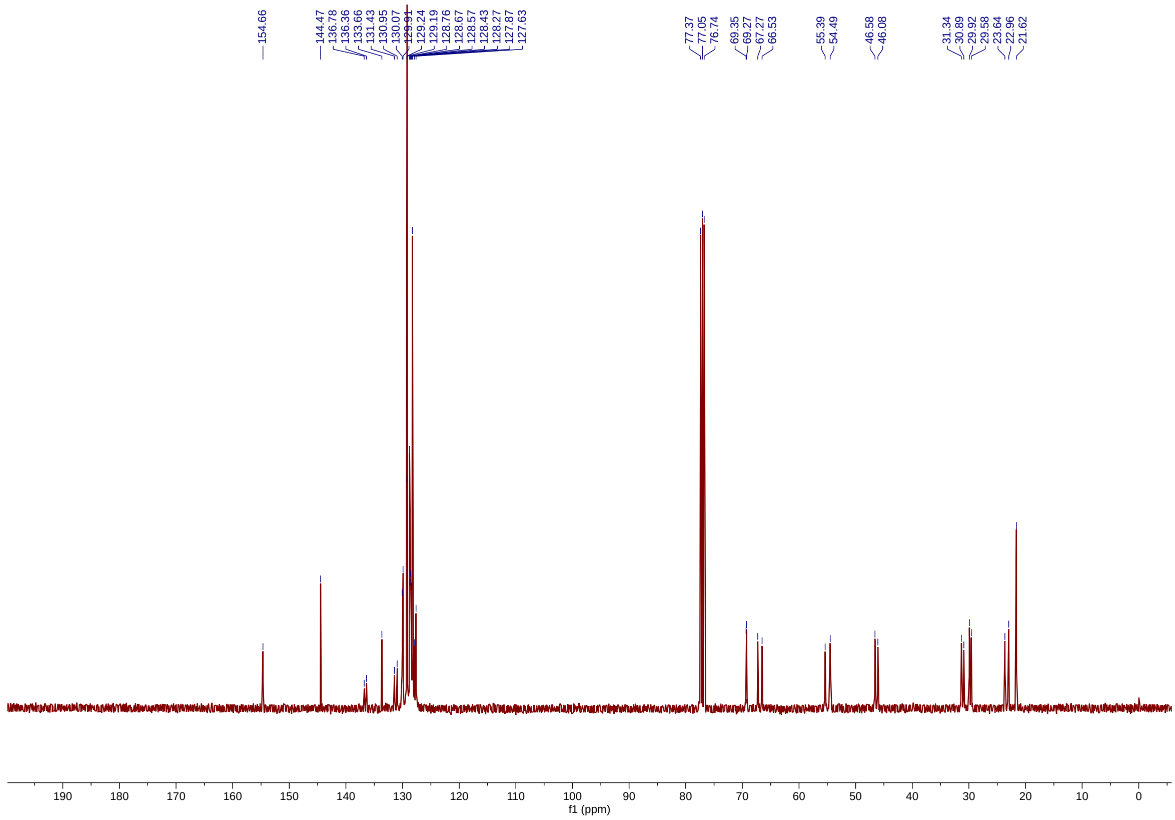

^{1}H (400 MHz) and ^{13}C (101 MHz) spectra of compound 5a in $CDCl_3$

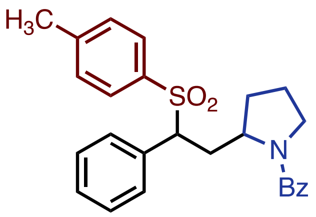

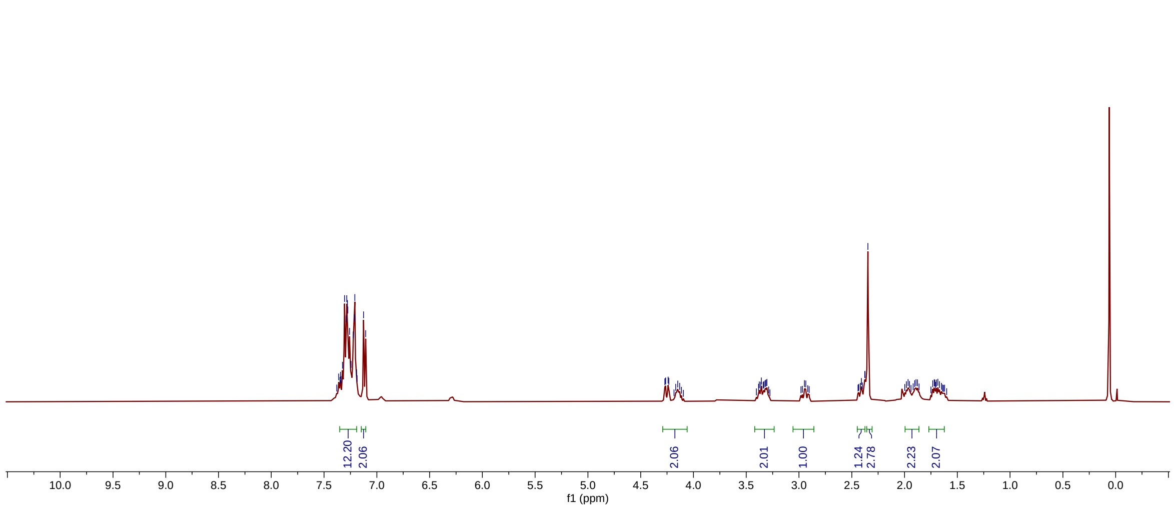

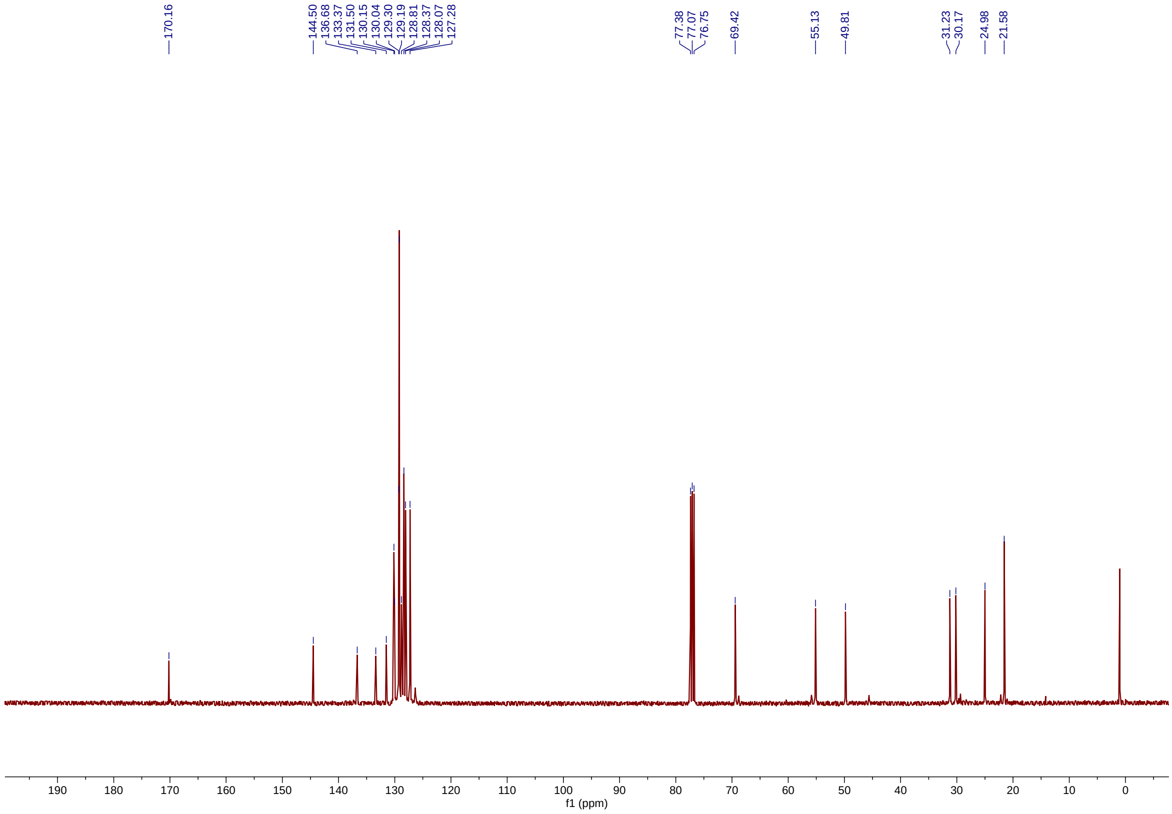

^{1}H (400 MHz) and ^{13}C (101 MHz) spectra of compound 5b in $CDCl_3$

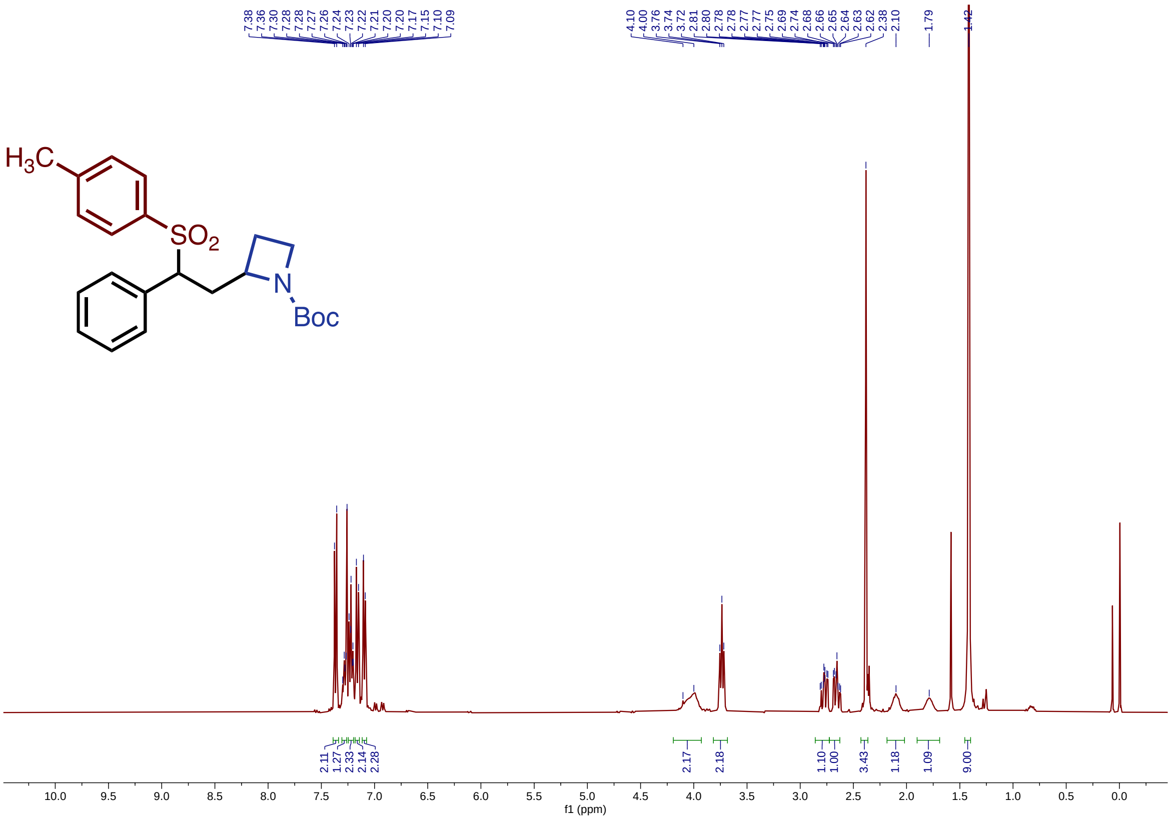

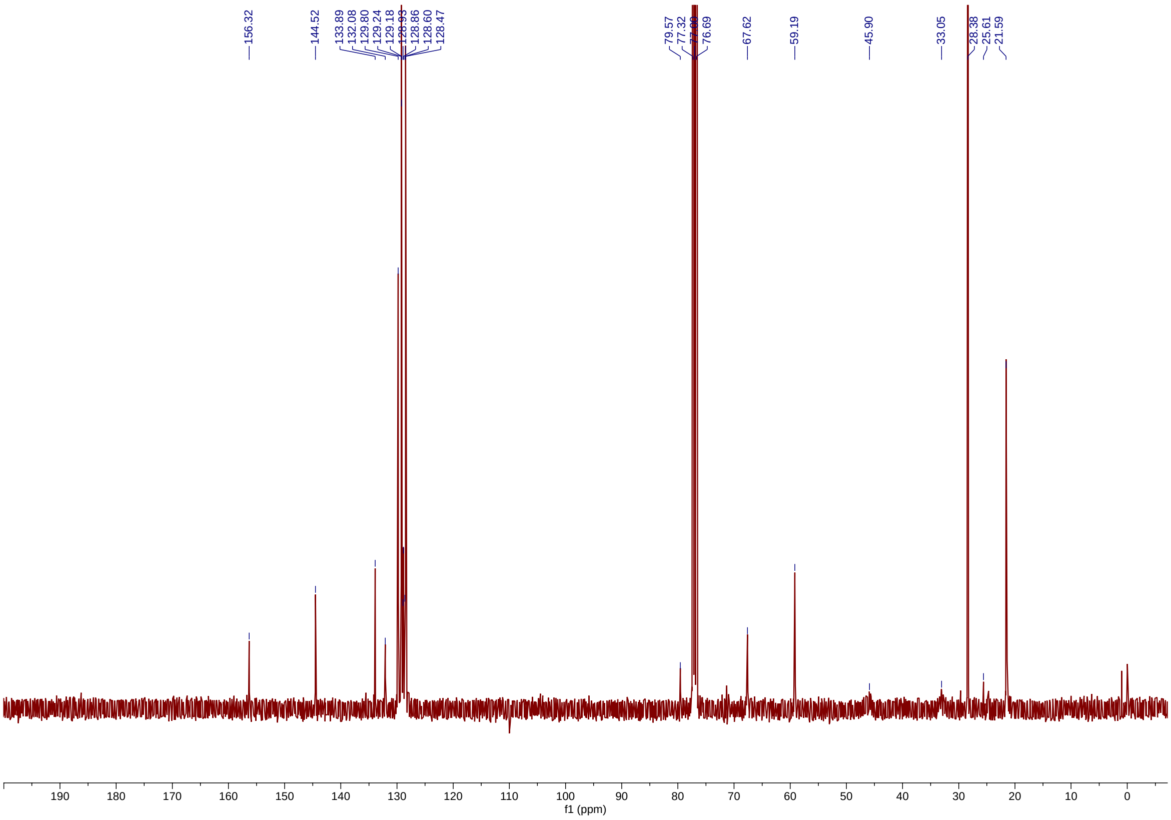

^{1}H (400 MHz) and ^{13}C (101 MHz) spectra of compound 5c in $CDCl_3$

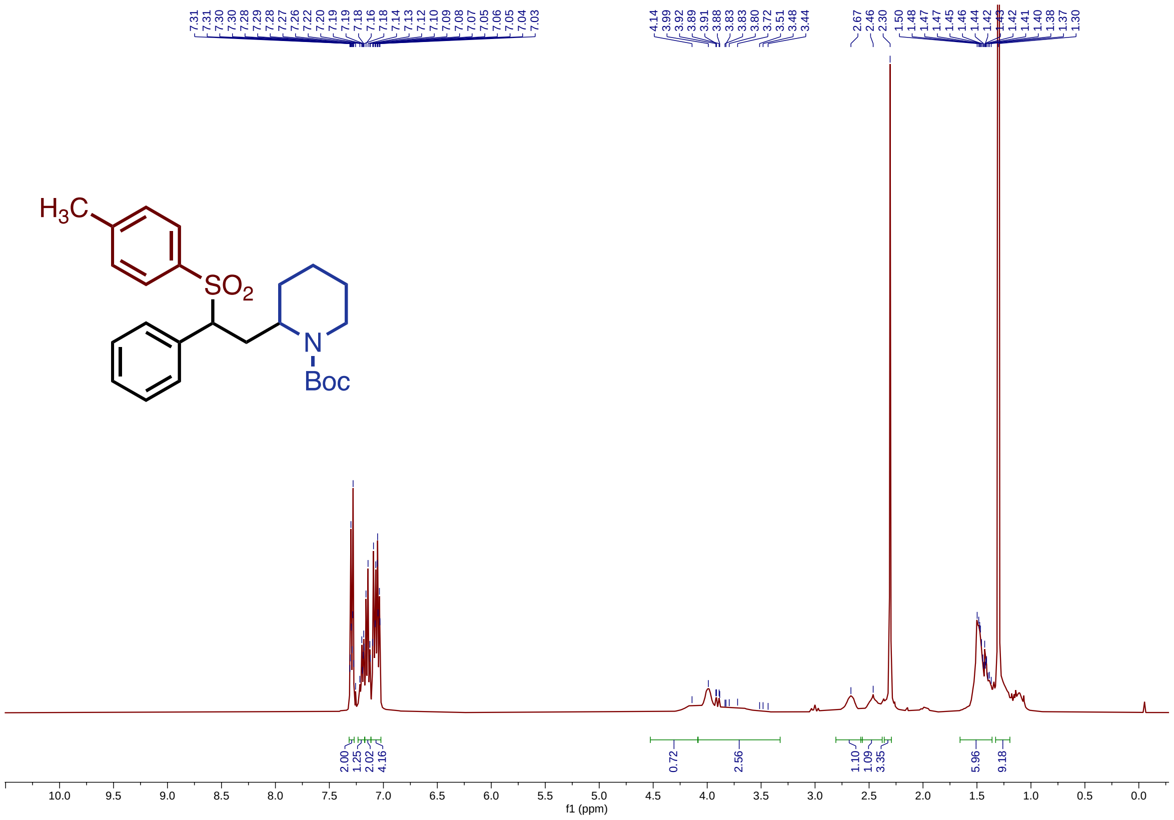

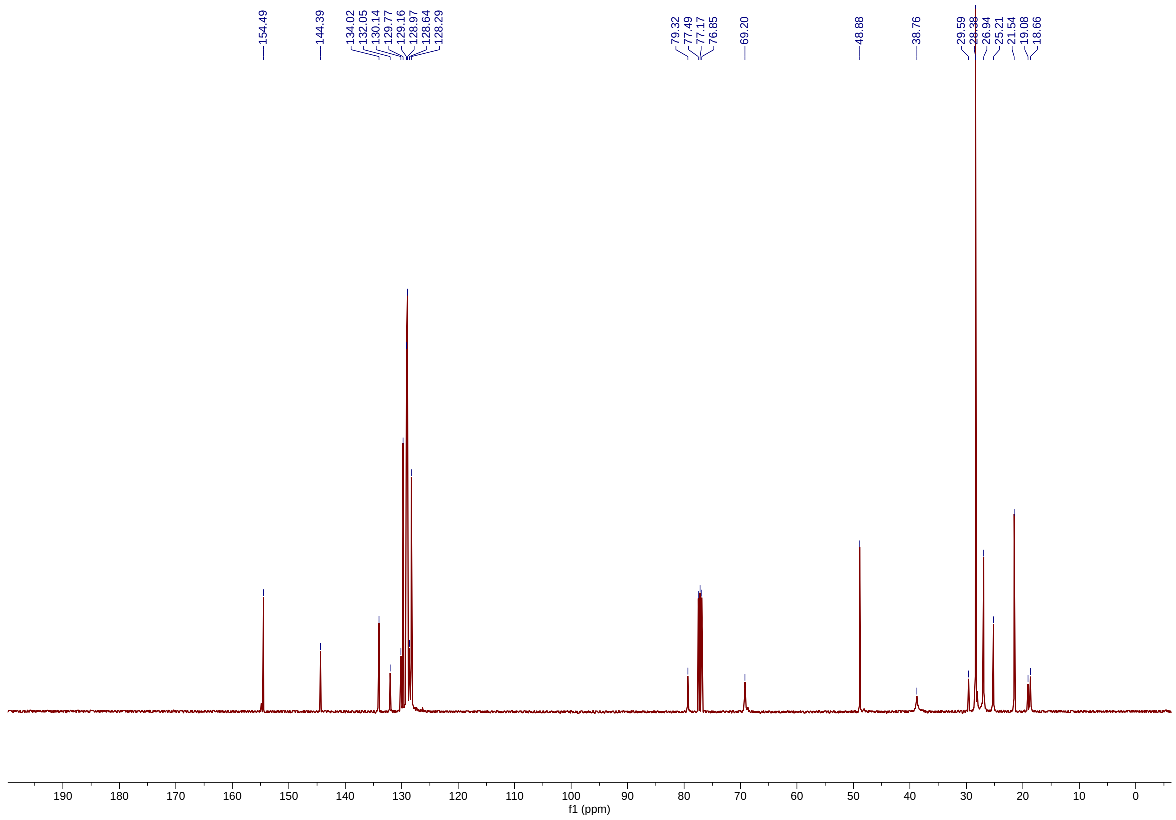

^{1}H (400 MHz) and ^{13}C (101 MHz) spectra of compound 5d in $CDCl_3$

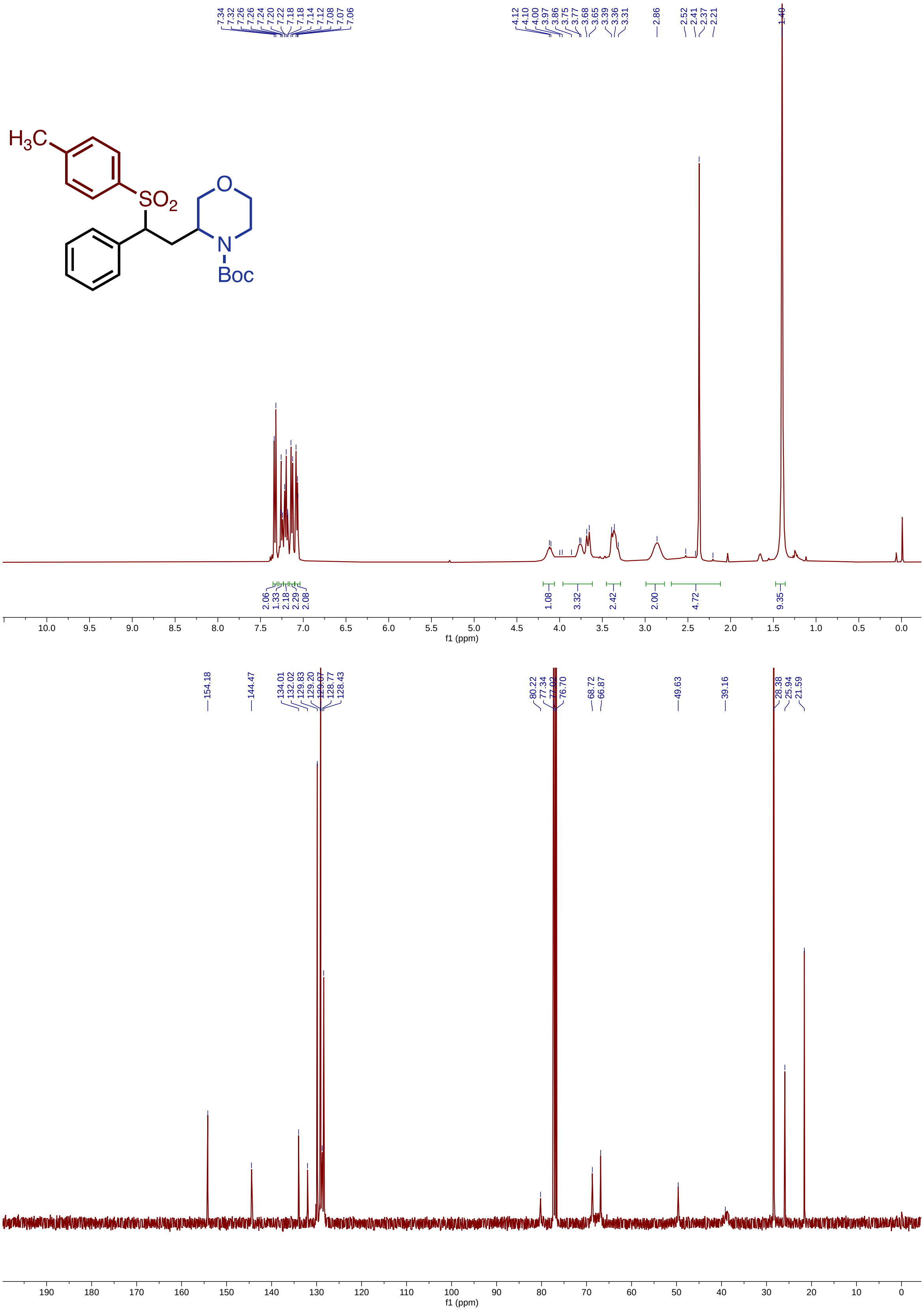

^{1}H (400 MHz) and ^{13}C (101 MHz) spectra of compound 5e in $CDCl_3$

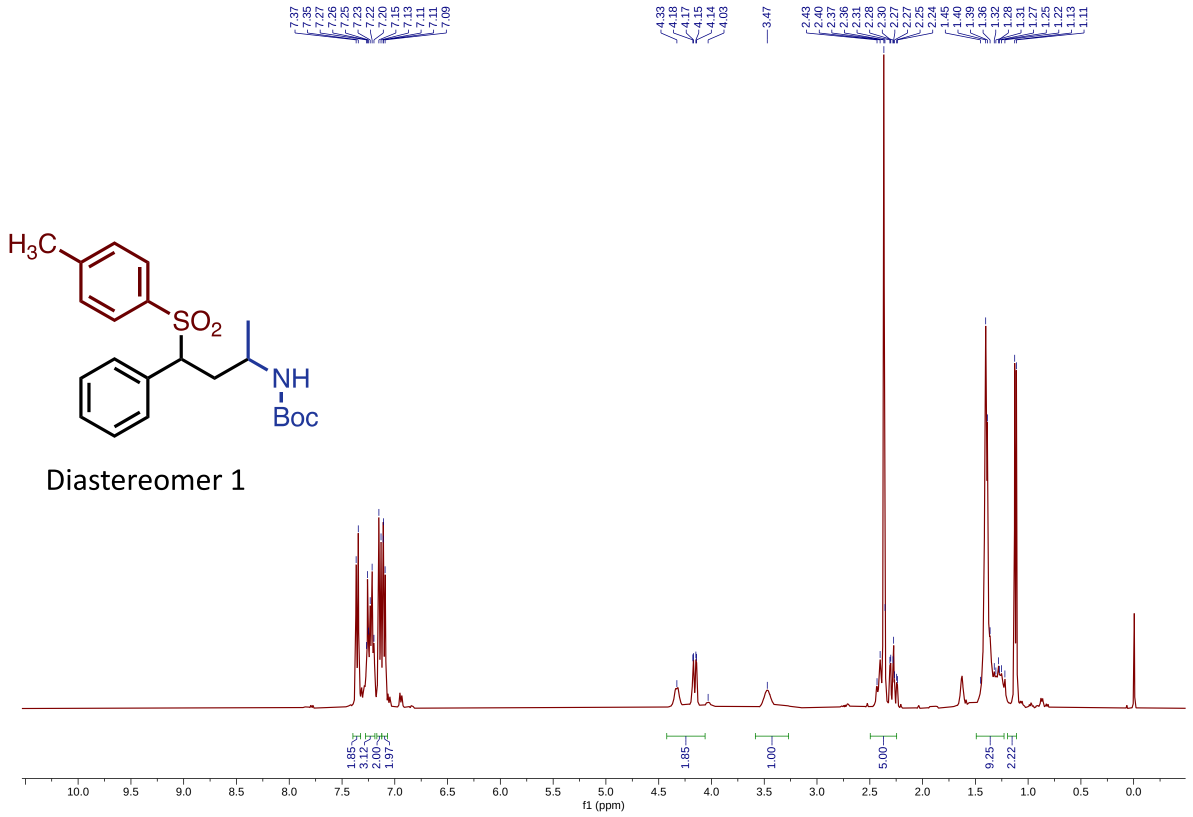

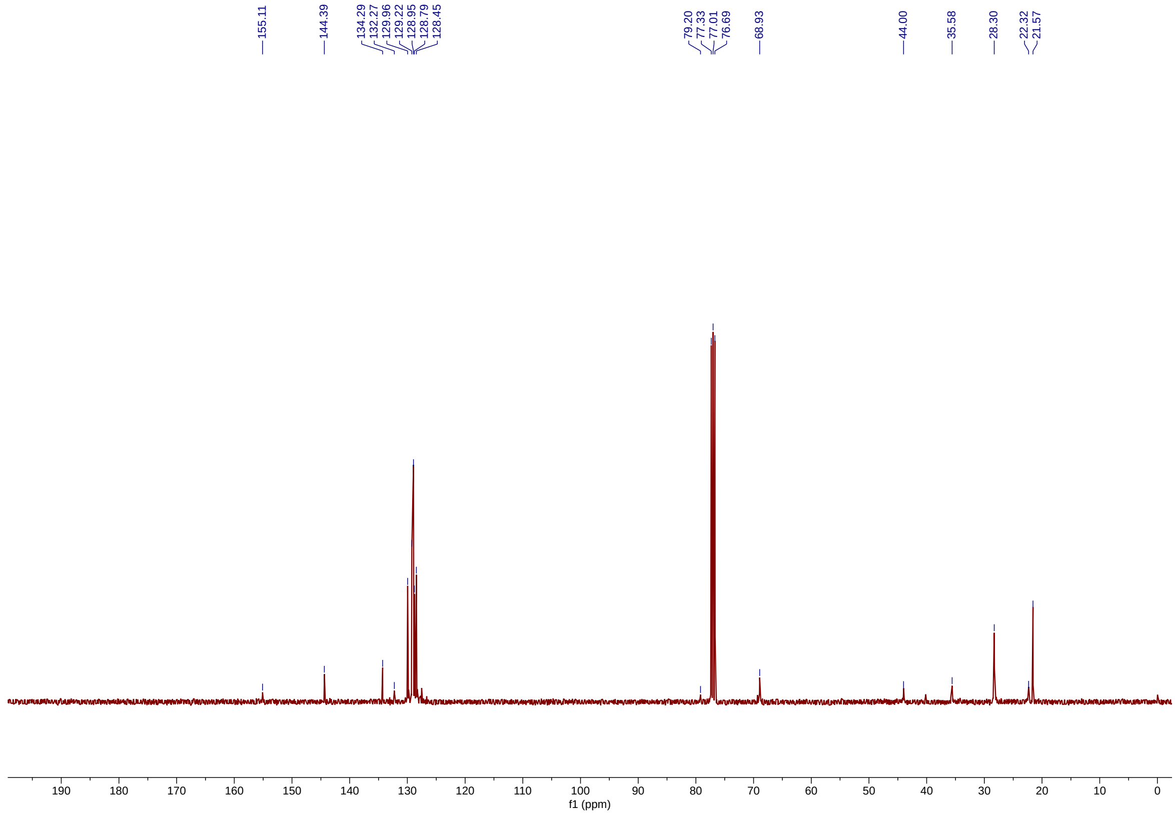

^{1}H (400 MHz) and ^{13}C (101 MHz) spectra of compound 5f in $CDCl_3$

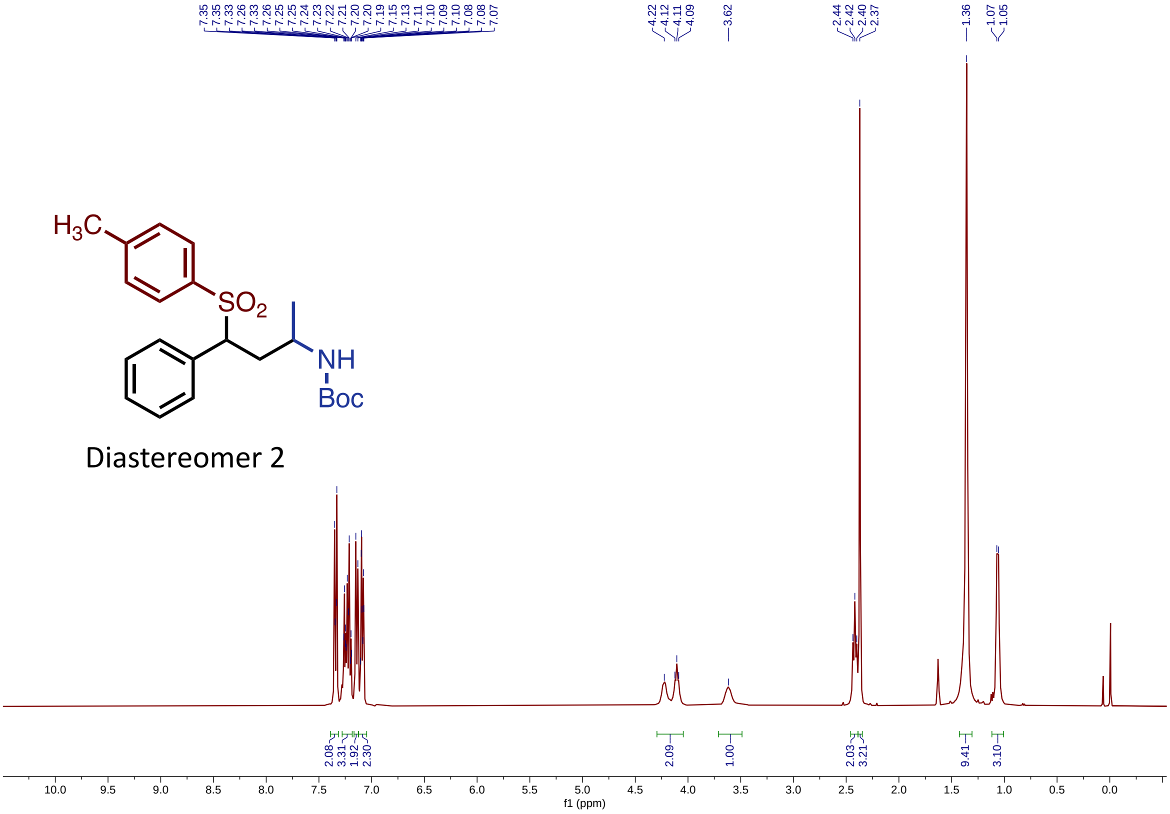

Diastereomer 2

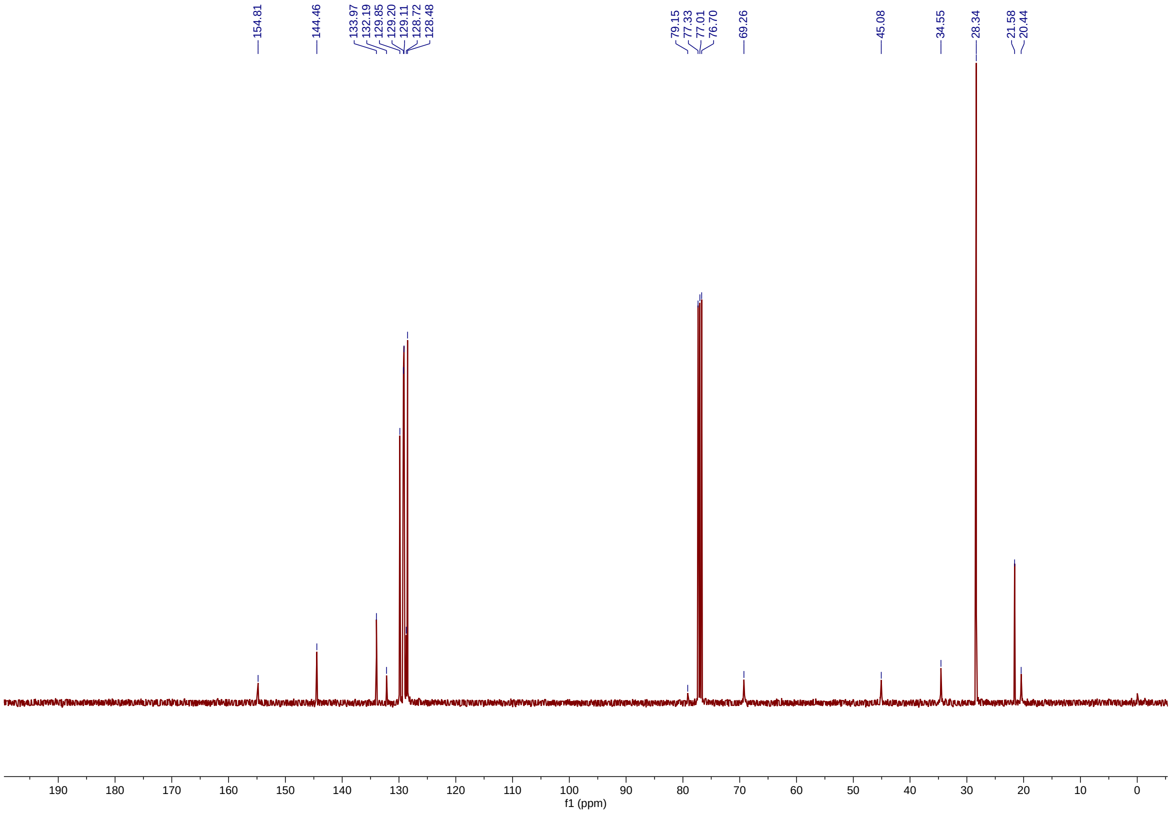

^{1}H (400 MHz) and ^{13}C (101 MHz) spectra of compound 5f in $CDCl_3$

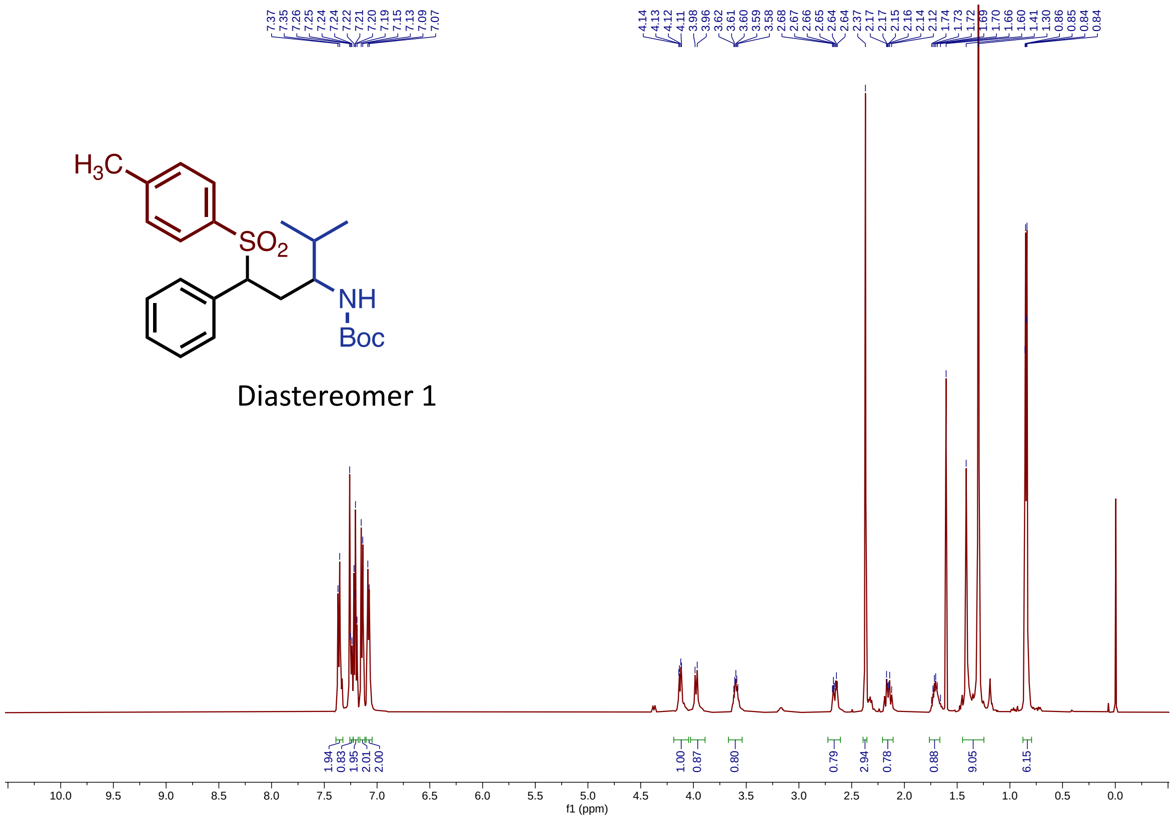

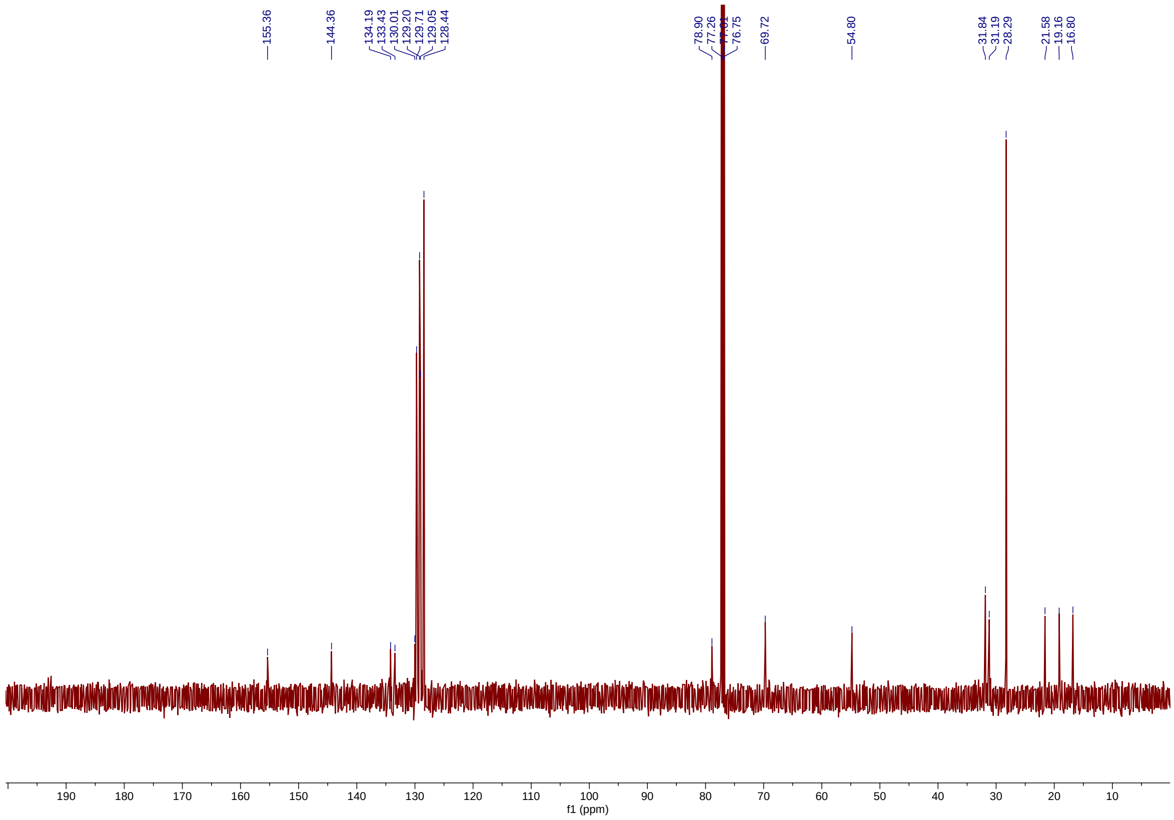

^{1}H (500 MHz) and ^{13}C (126 MHz) spectra of compound 5g in $CDCl_3$

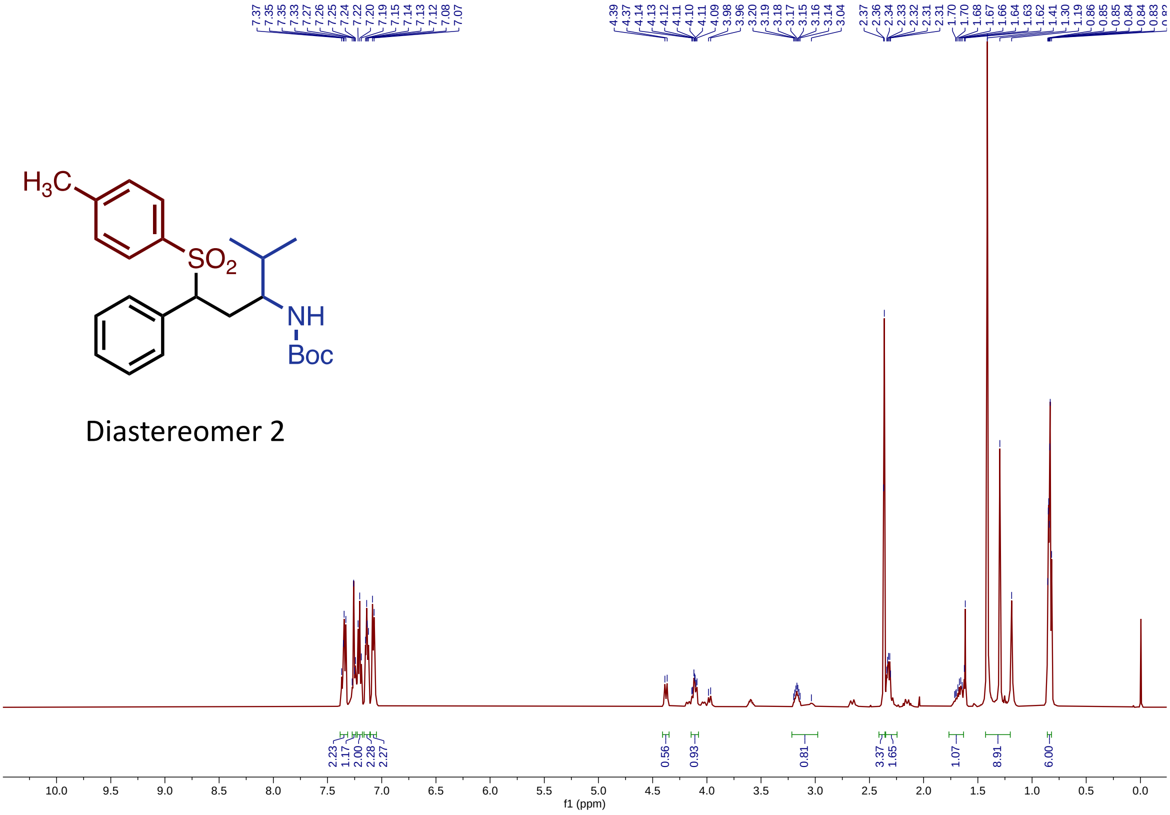

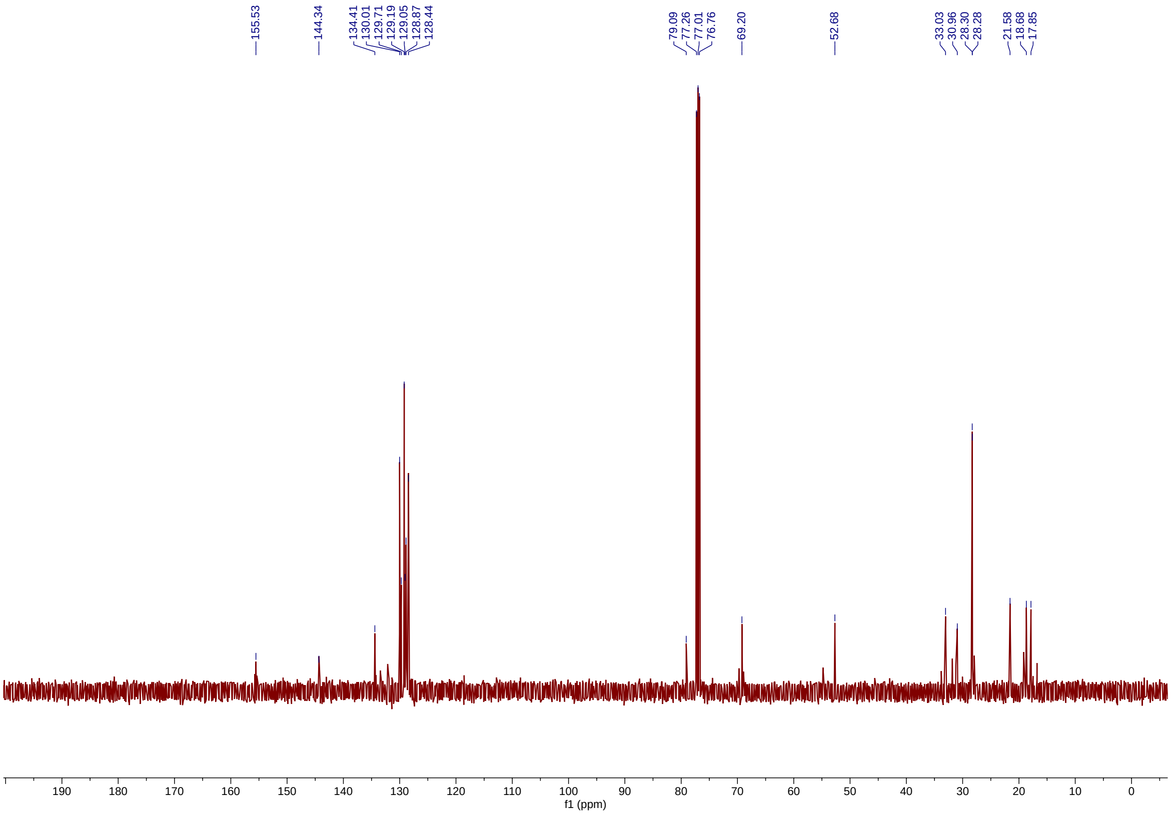

^{1}H (500 MHz) and ^{13}C (126 MHz) spectra of compound 5g in $CDCl_3$

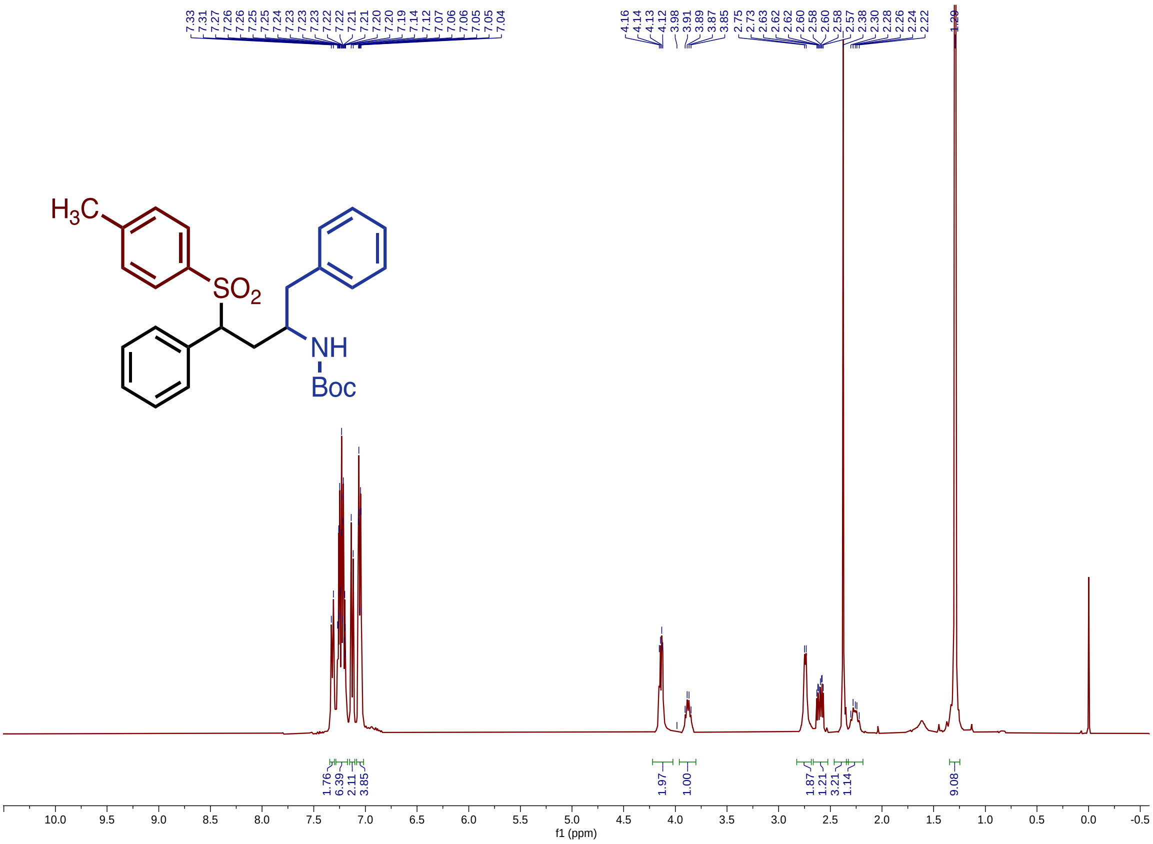

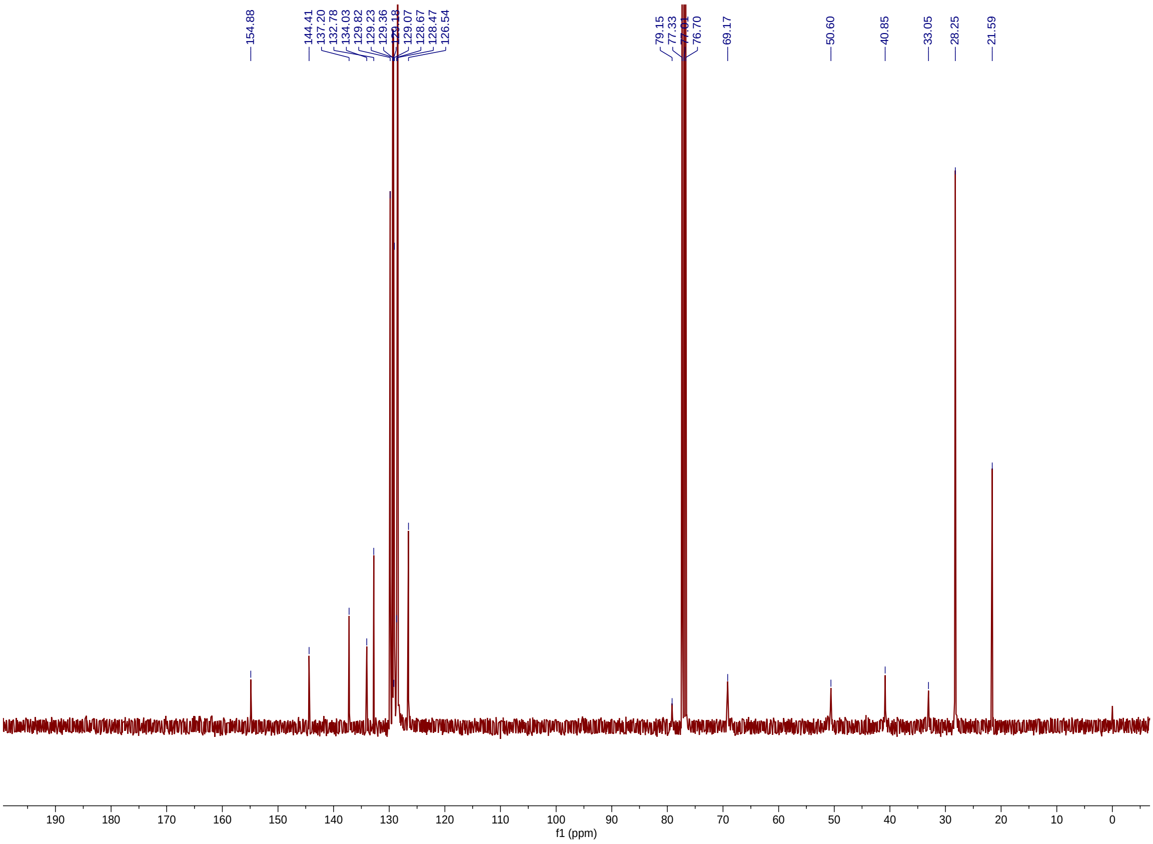

^{1}H (400 MHz) and ^{13}C (101 MHz) spectra of compound 5h in $CDCl_3$

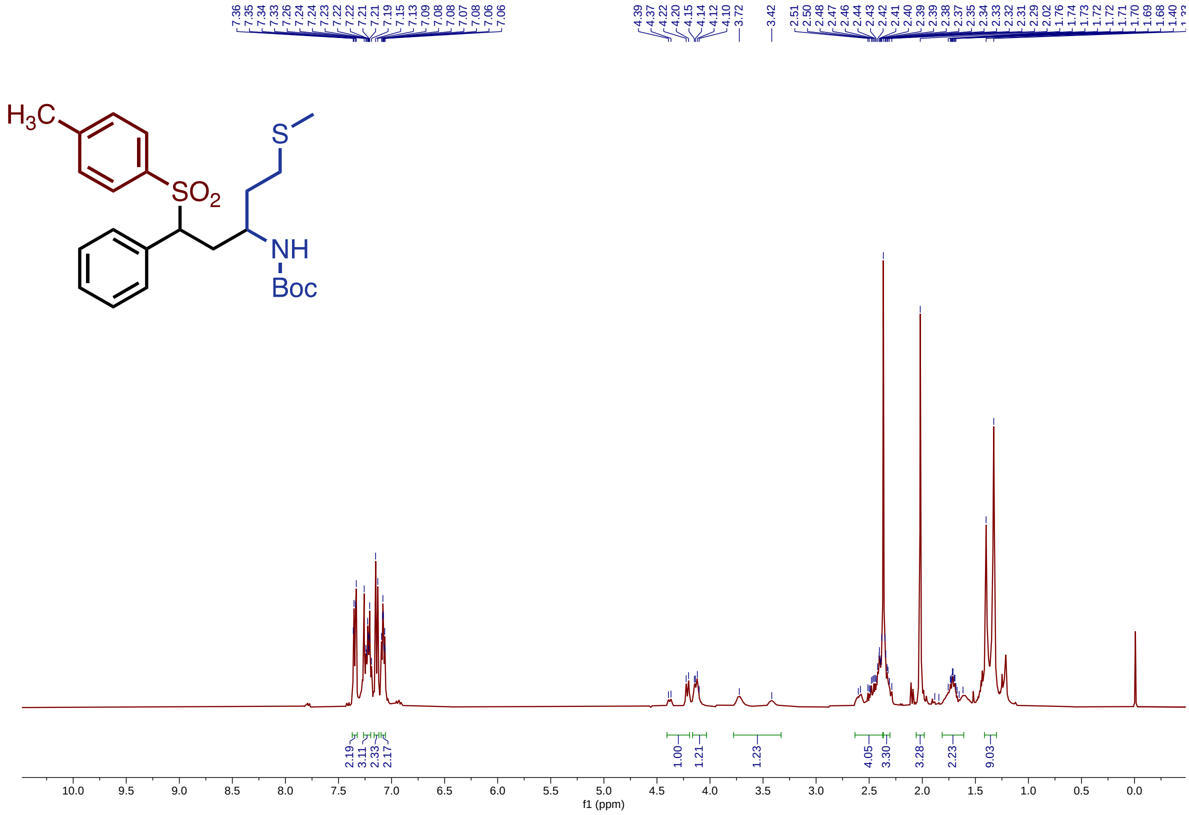

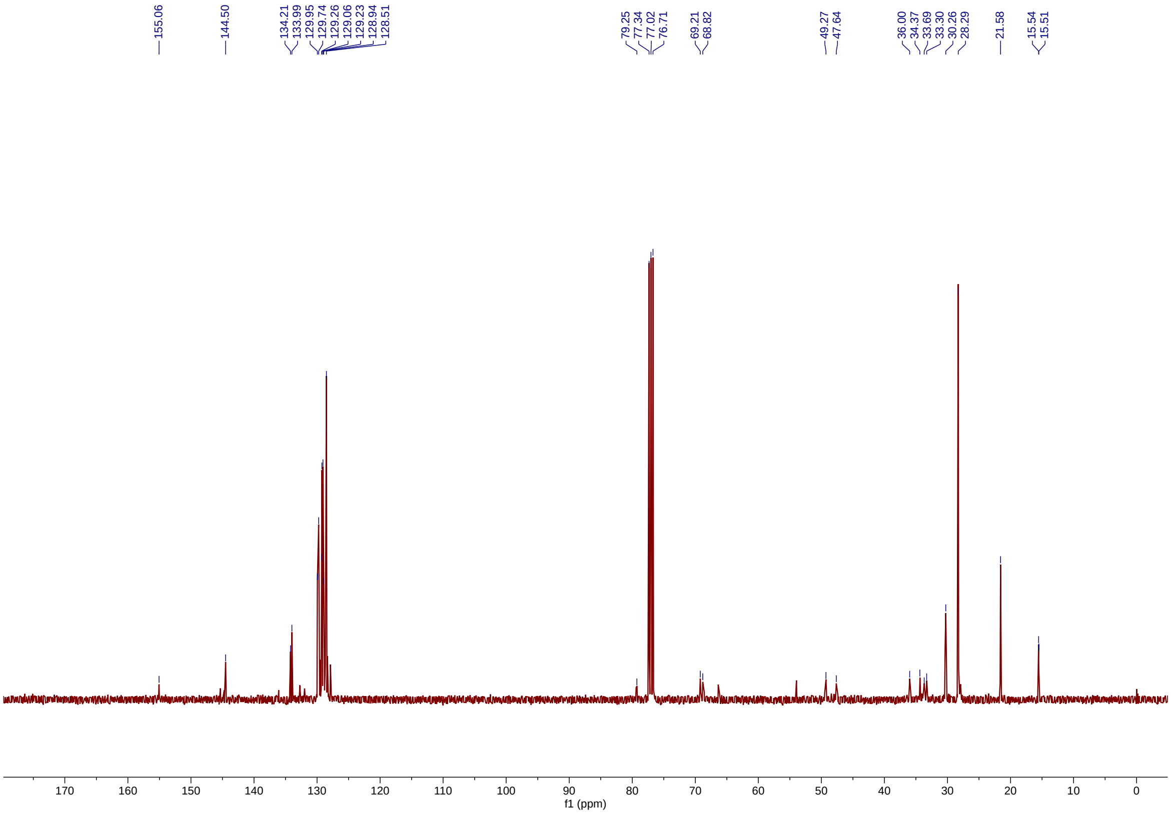

^{1}H (400 MHz) and ^{13}C (101 MHz) spectra of compound 5i in $CDCl_3$

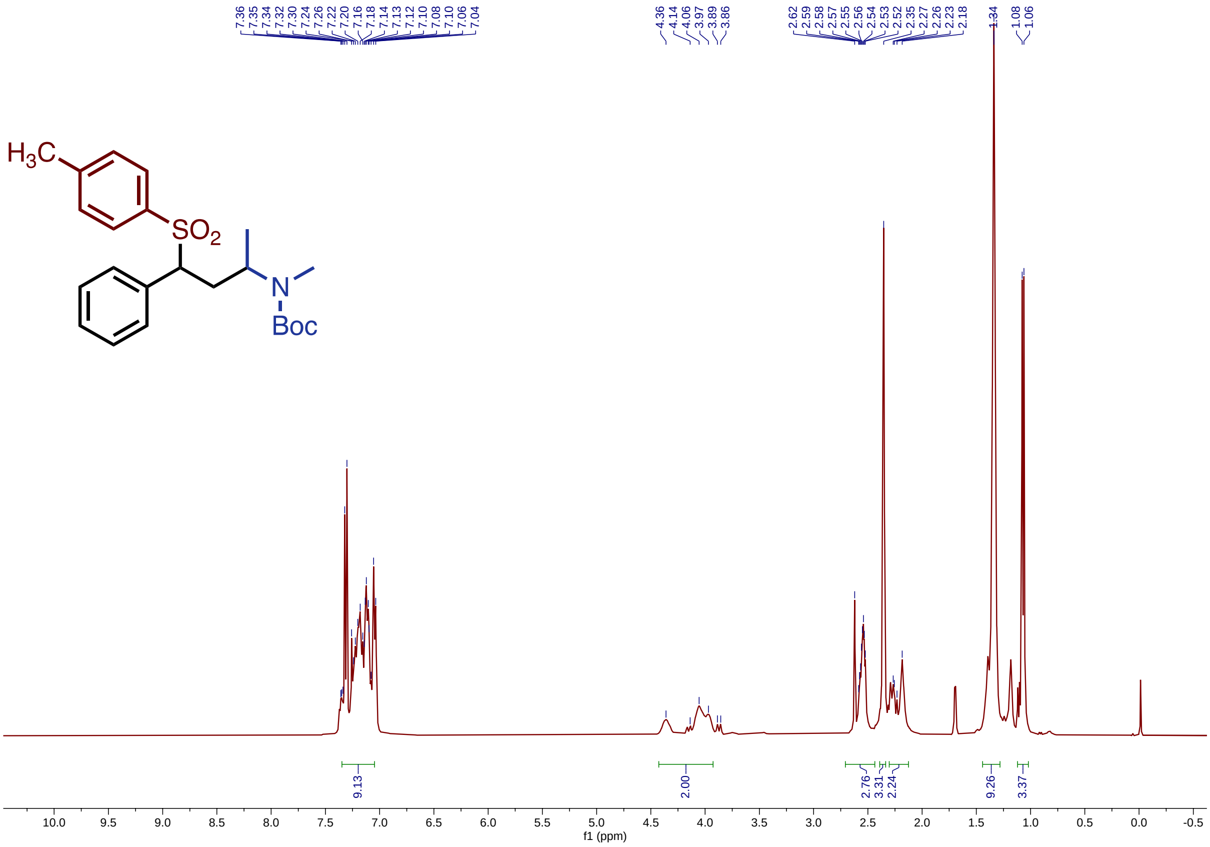

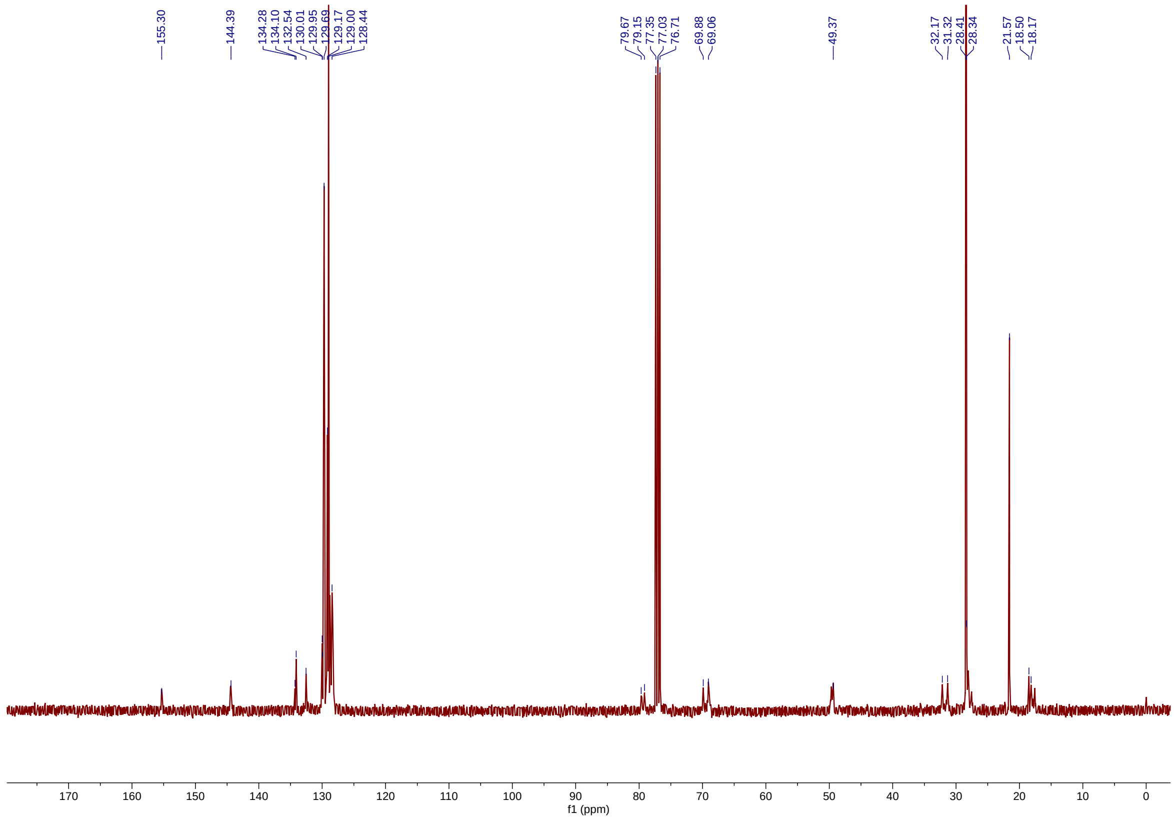

^{1}H (400 MHz) and ^{13}C (101 MHz) spectra of compound 5j in $CDCl_3$

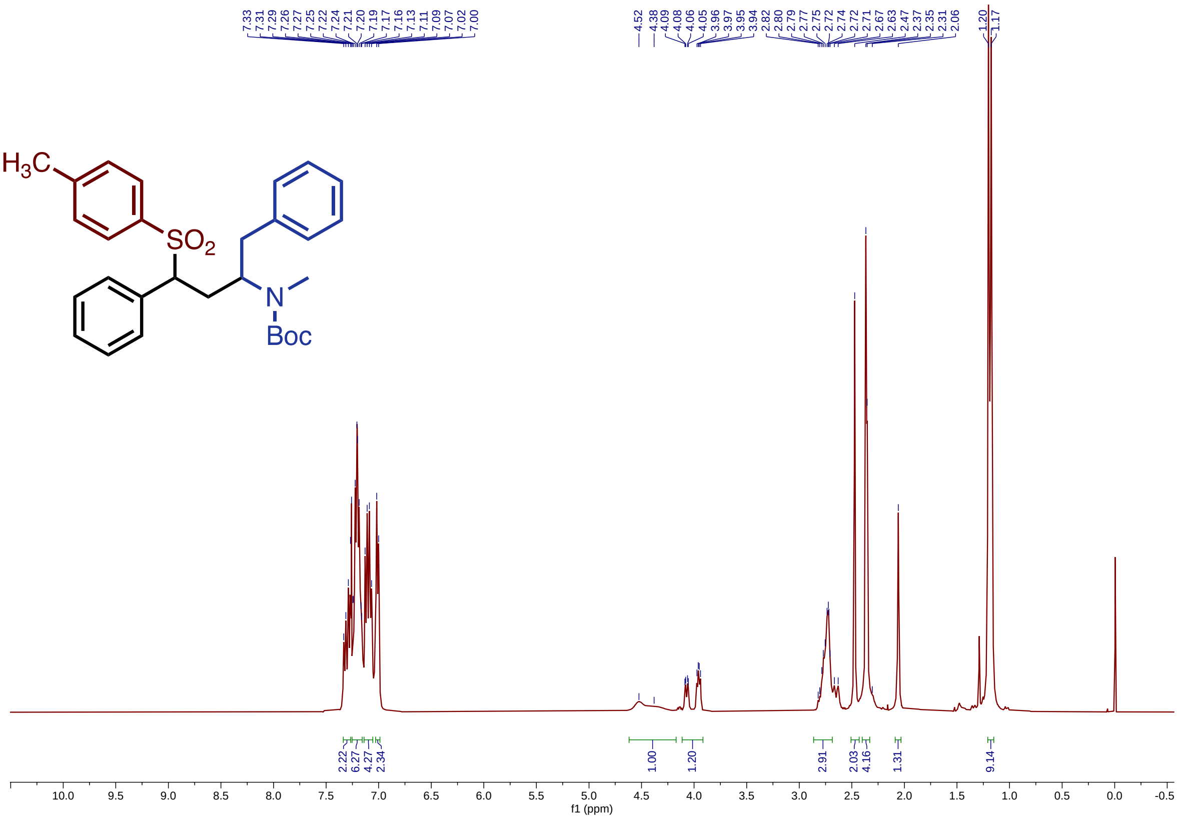

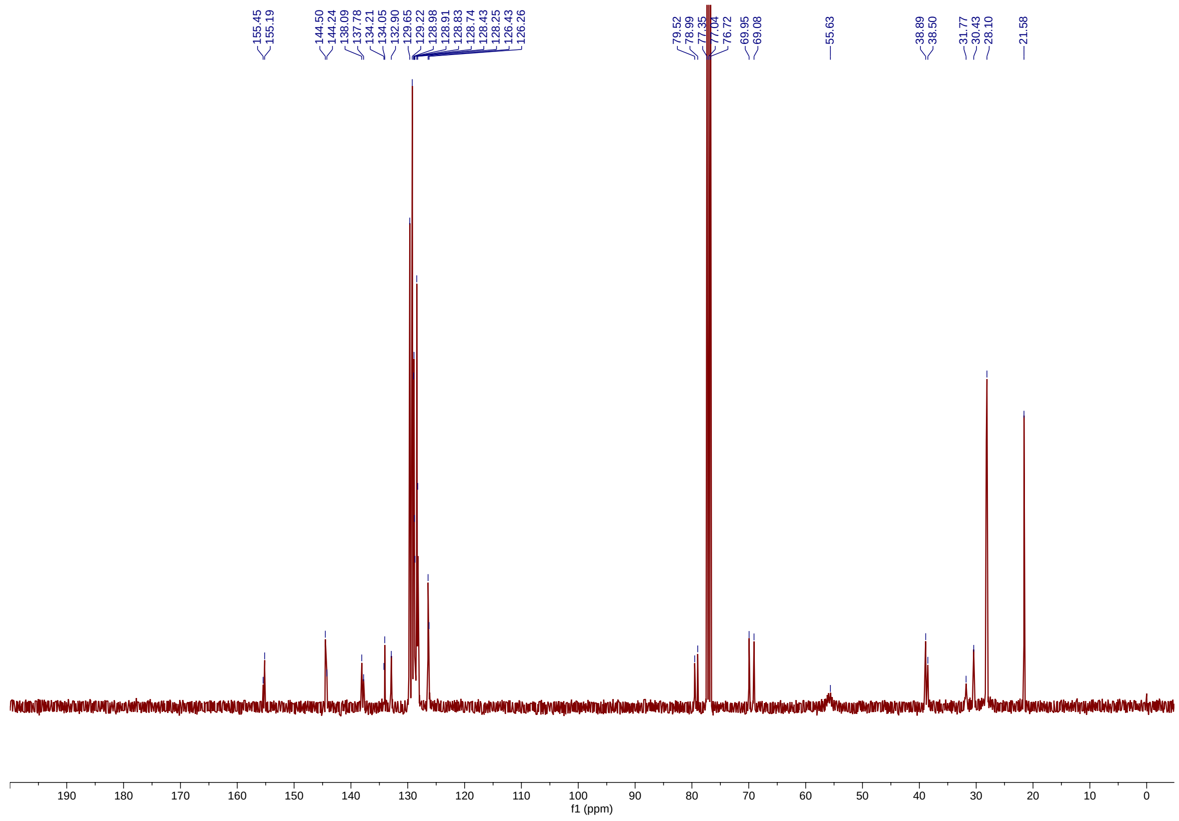

^{1}H (400 MHz) and ^{13}C (101 MHz) spectra of compound 5k in $CDCl_3$

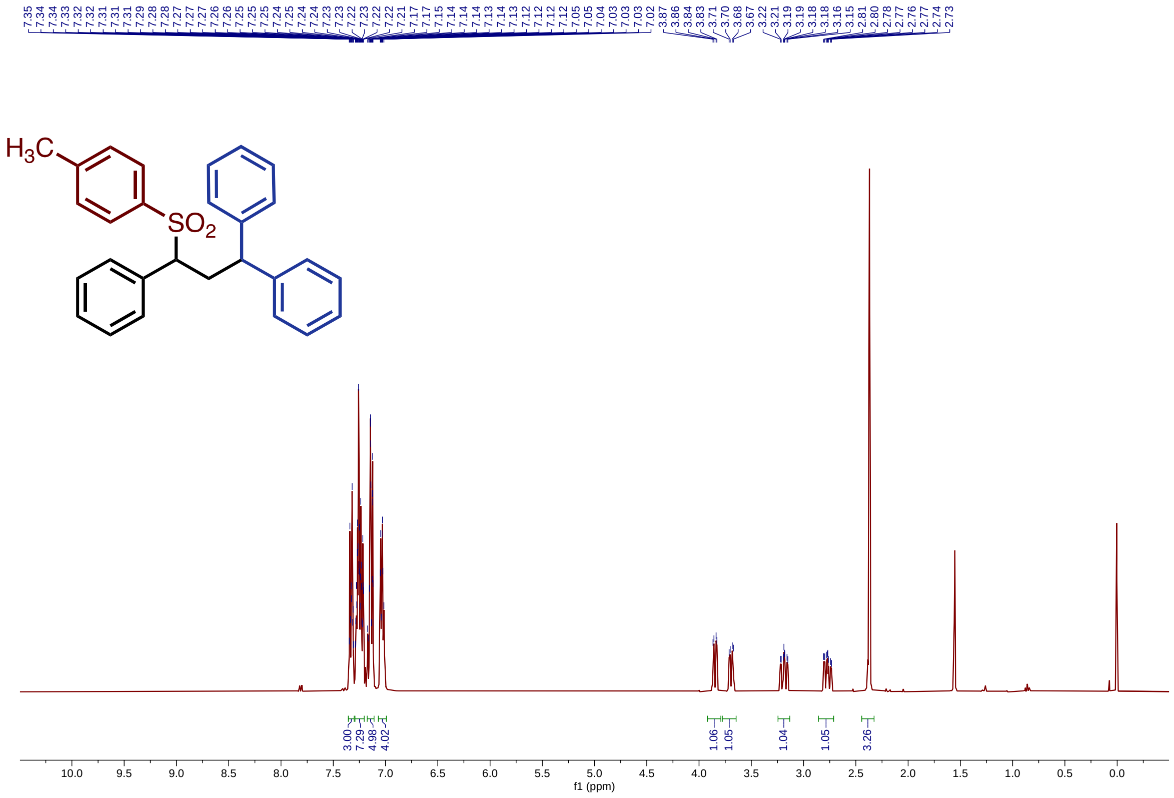

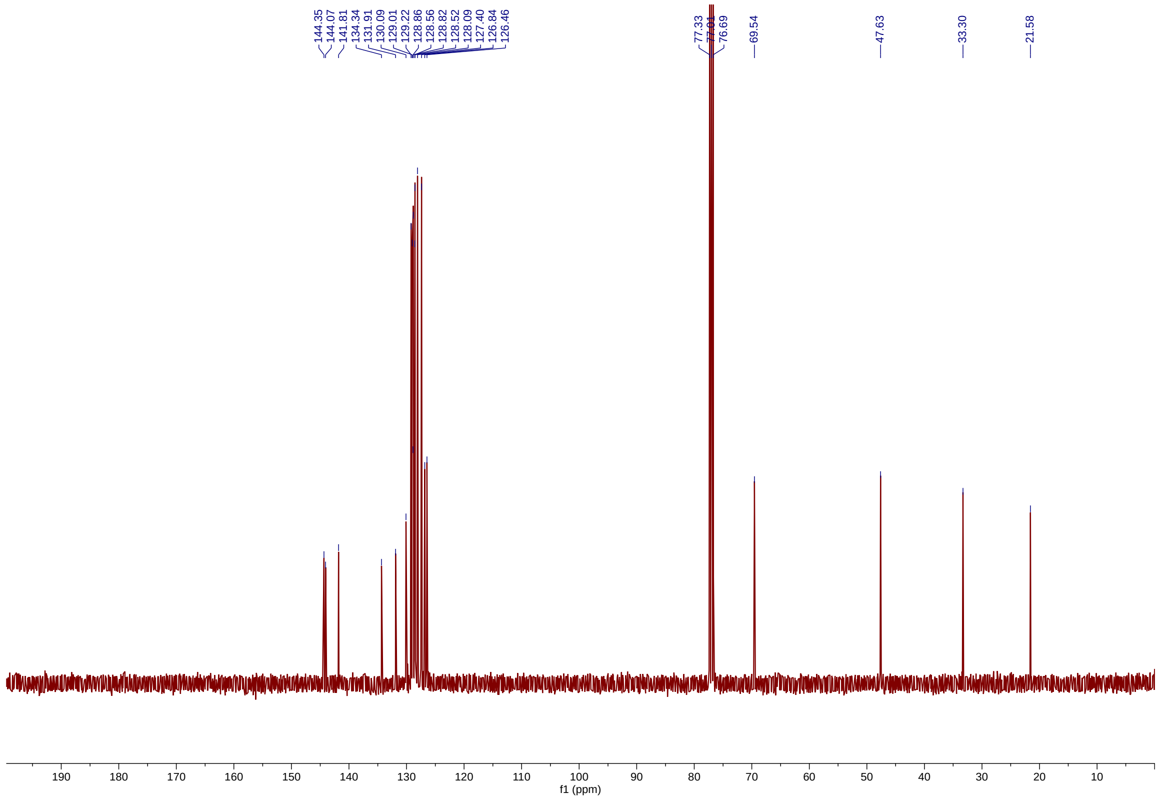

^1H (400 MHz) and ^{13}C (101 MHz) spectra of compound 5l in $CDCl_3$

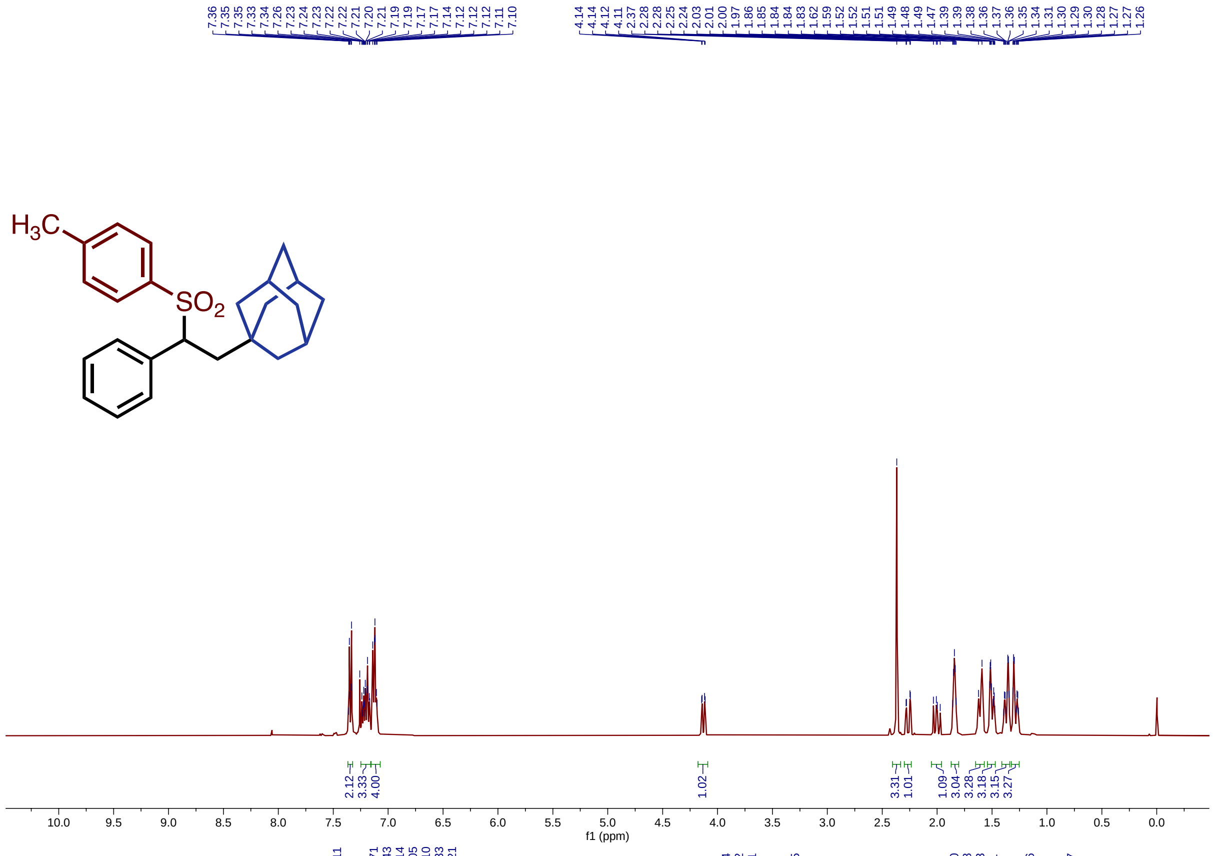

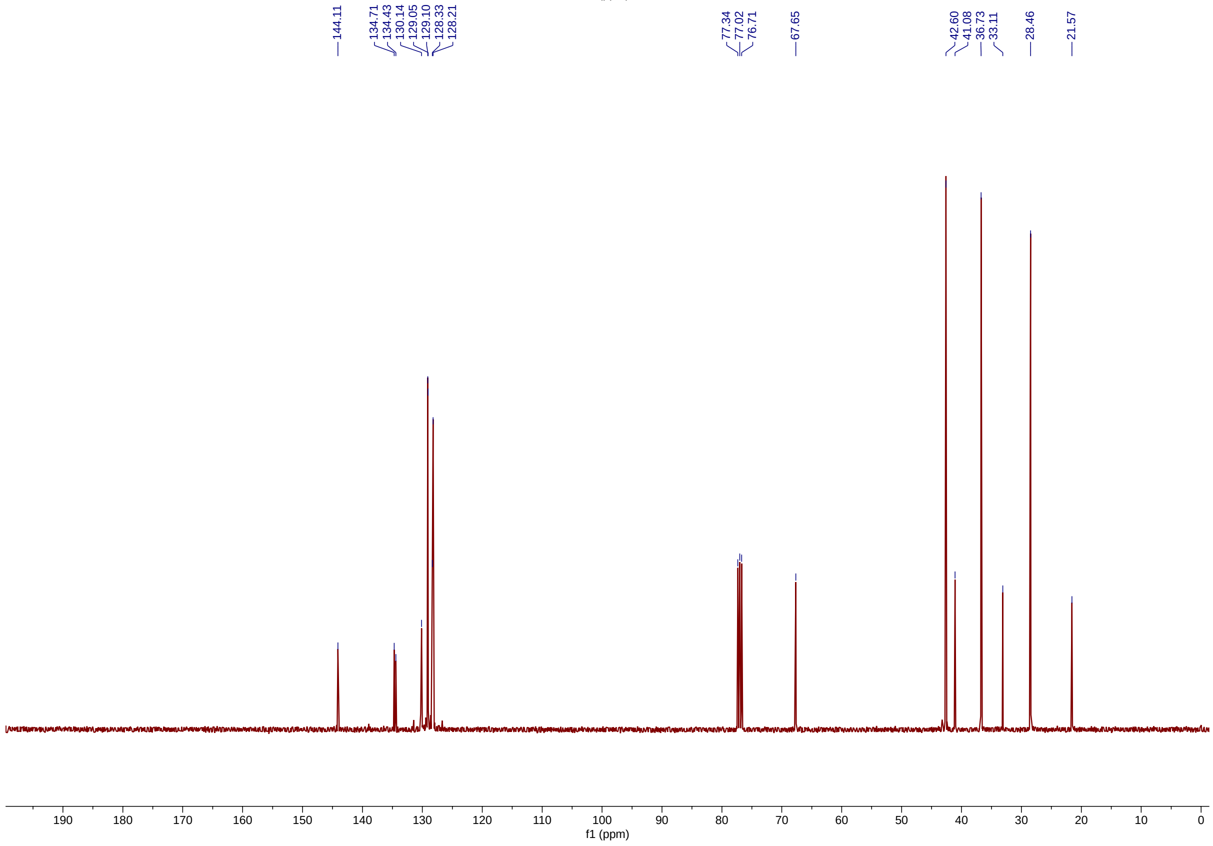

^{1}H (400 MHz) and ^{13}C (101 MHz) spectra of compound 5m in $CDCl_3$

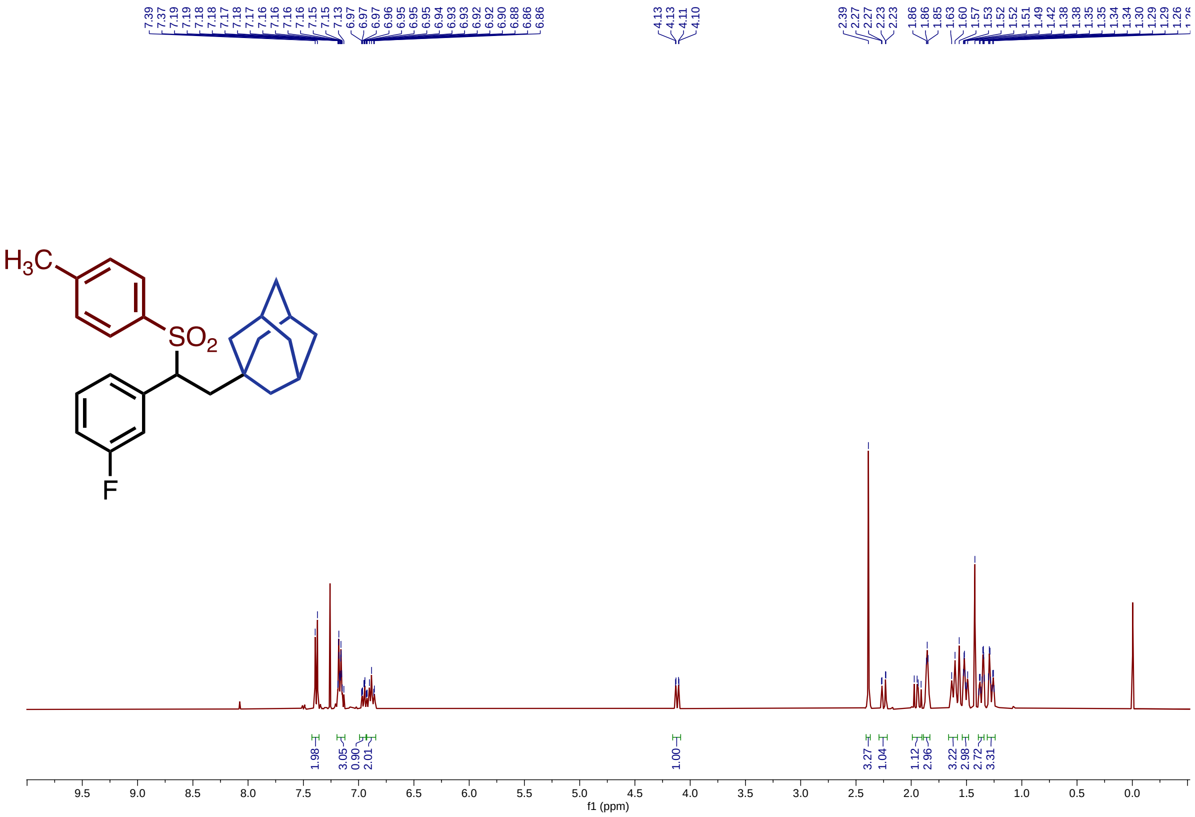

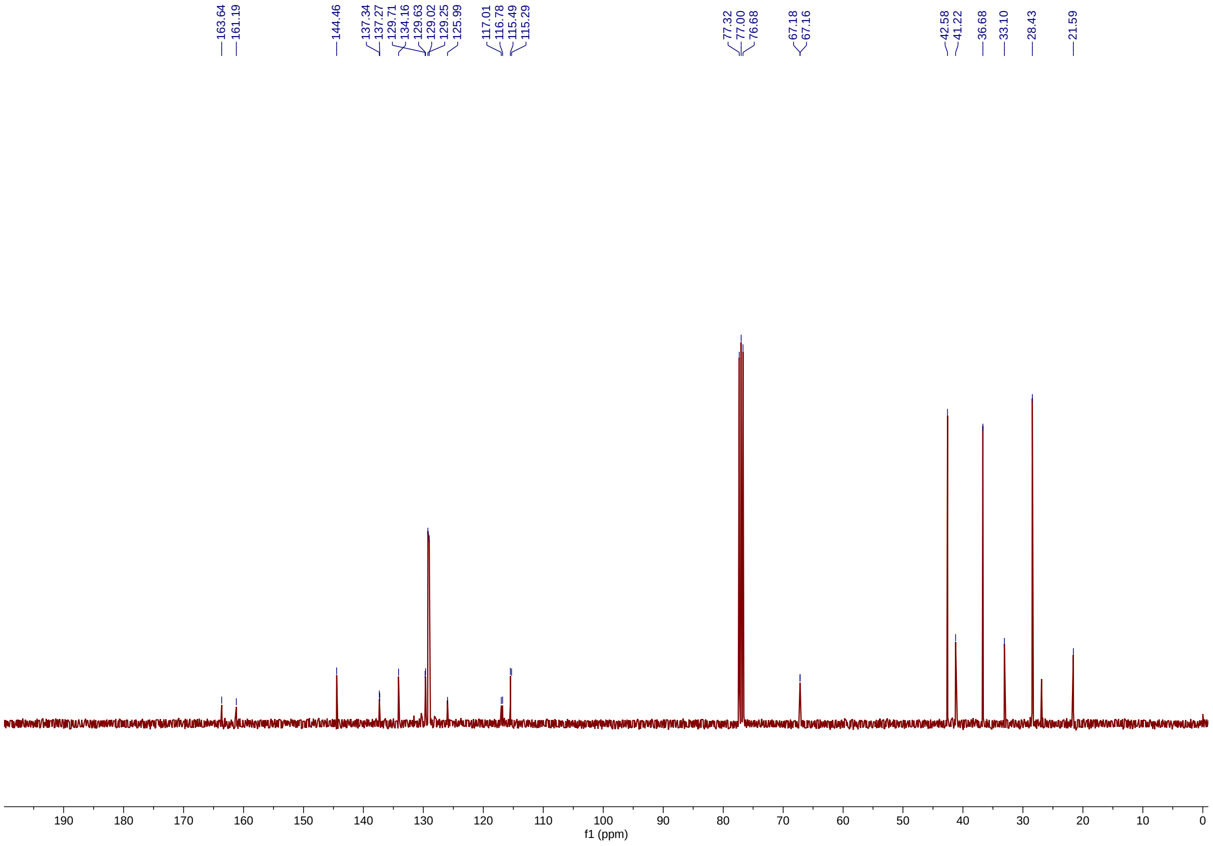

^{1}H (400 MHz) and ^{13}C (101 MHz) spectra of compound 5n in $CDCl_3$

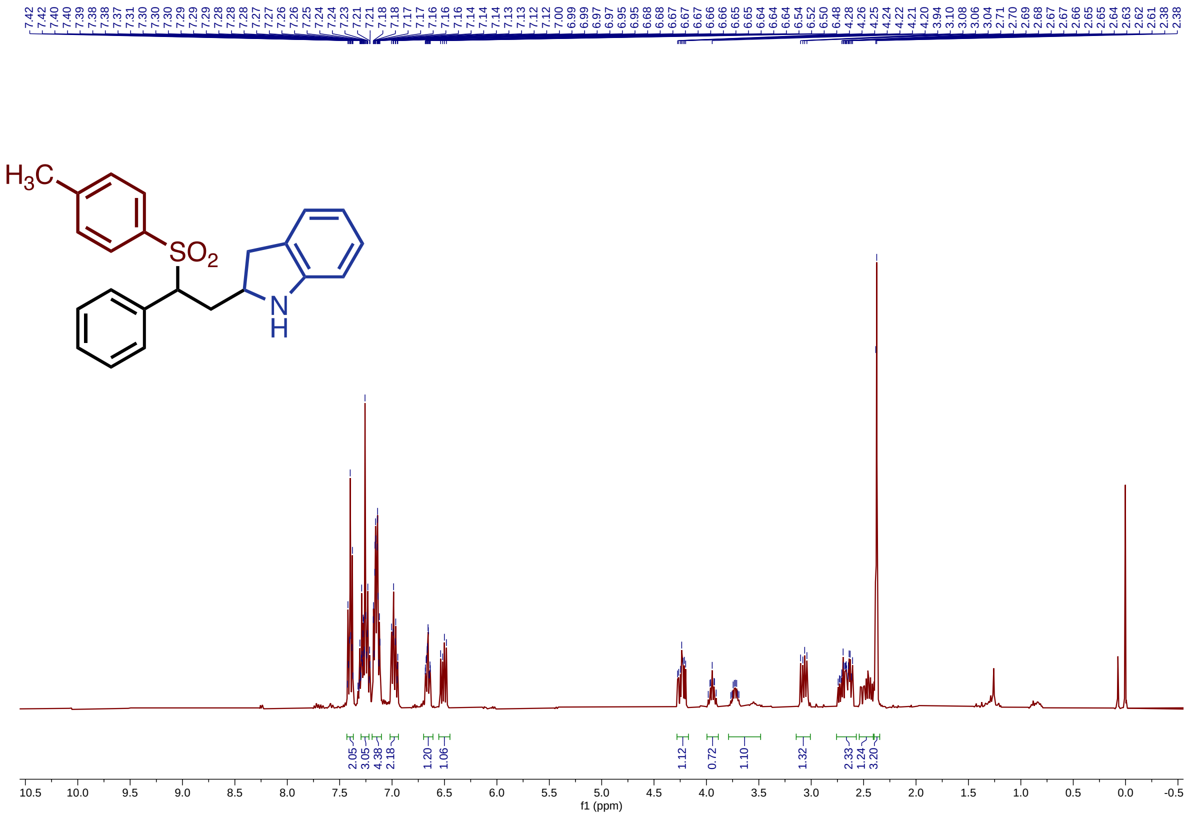

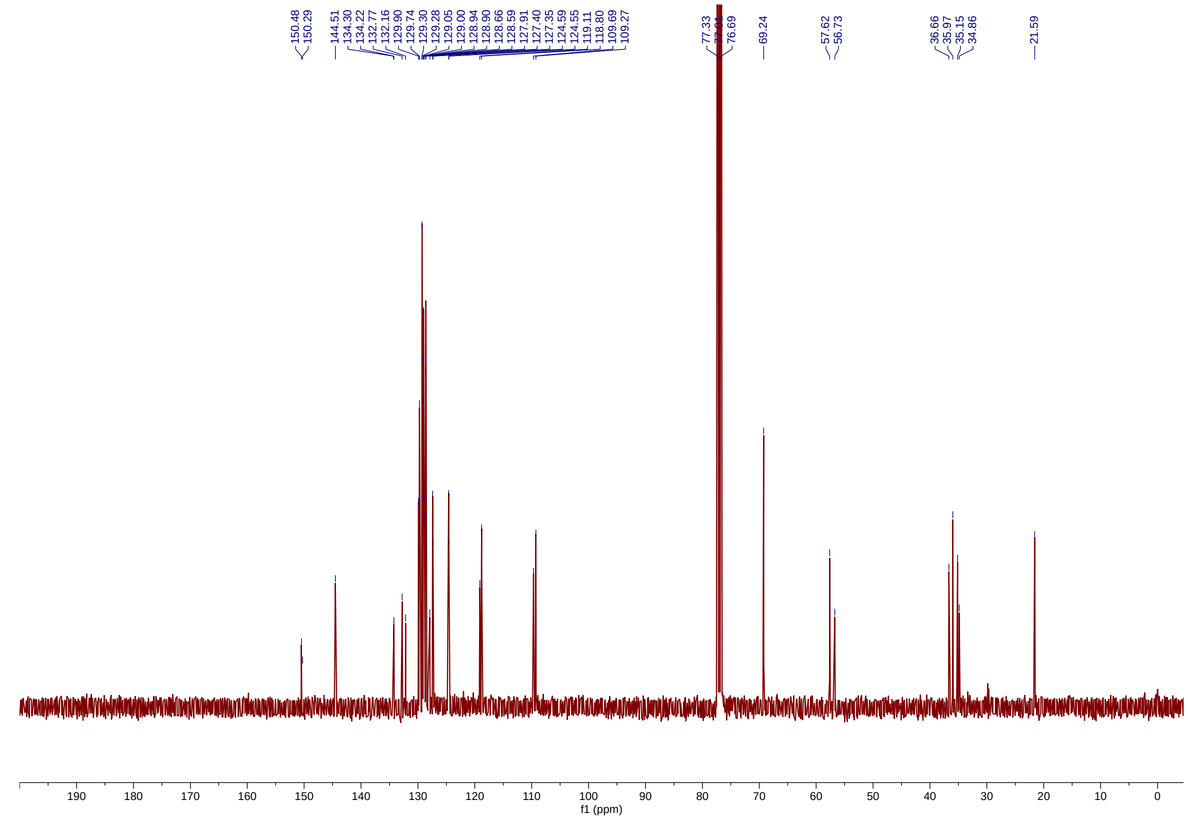

^{1}H (400 MHz) and ^{13}C (101 MHz) spectra of compound 5o in $CDCl_3$

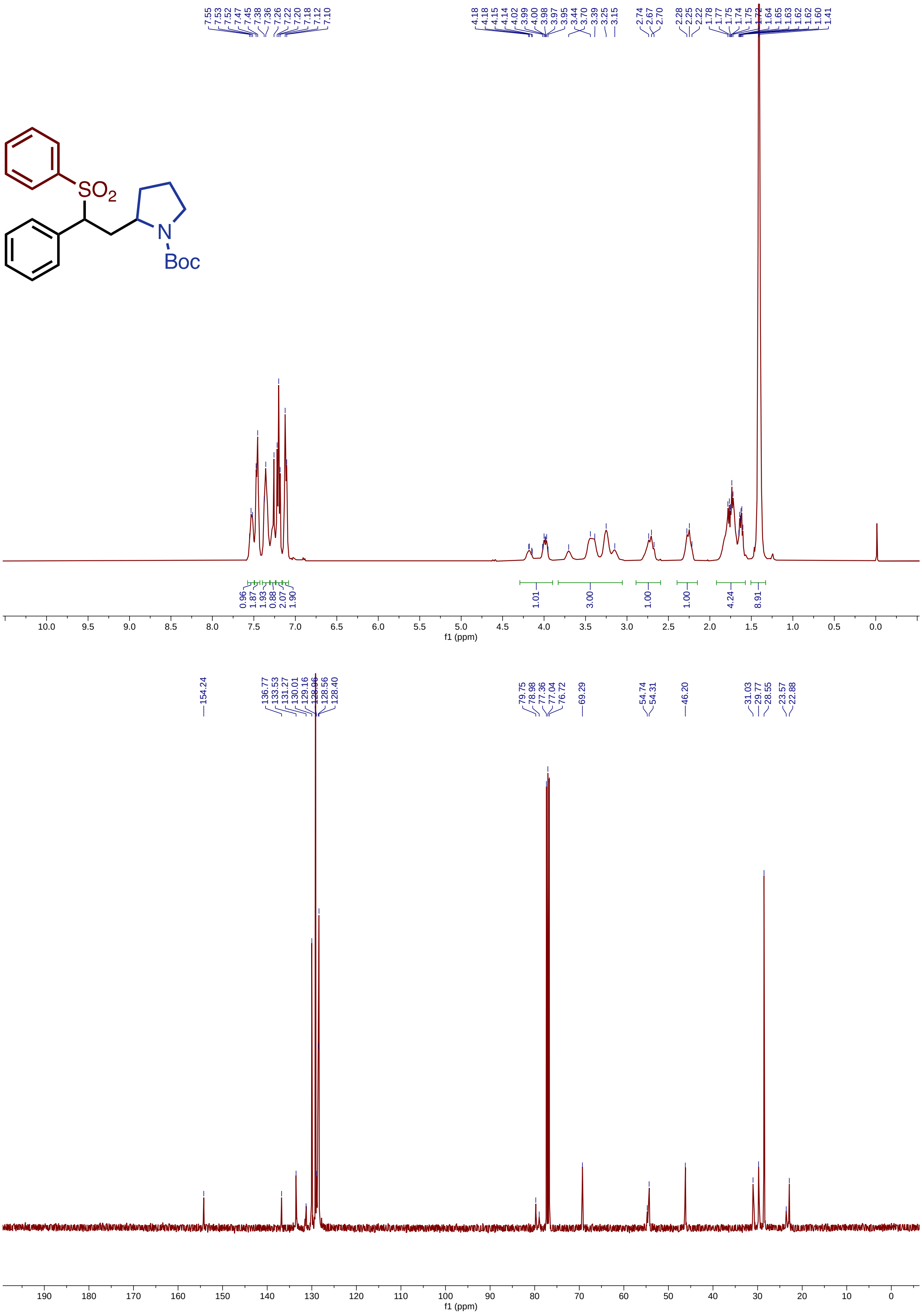

^{1}H (400 MHz) and ^{13}C (101 MHz) spectra of compound 6a in CDCl3

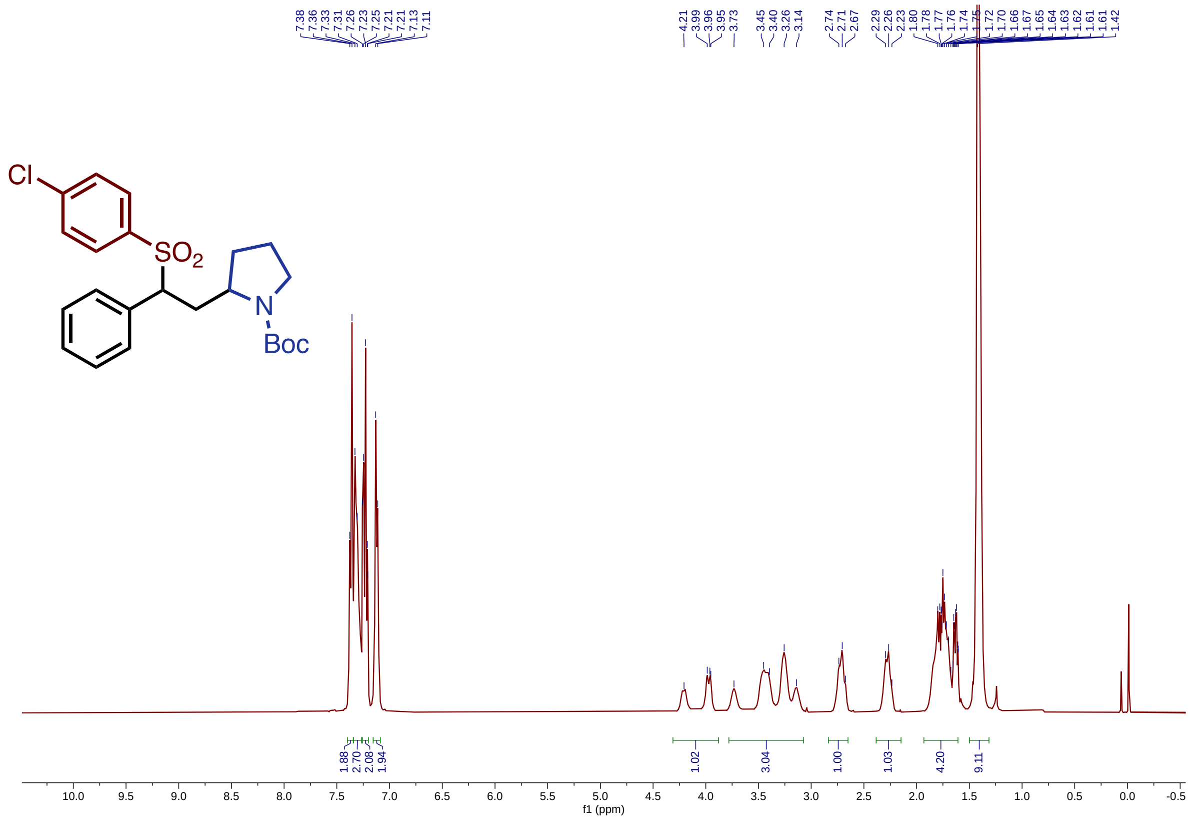

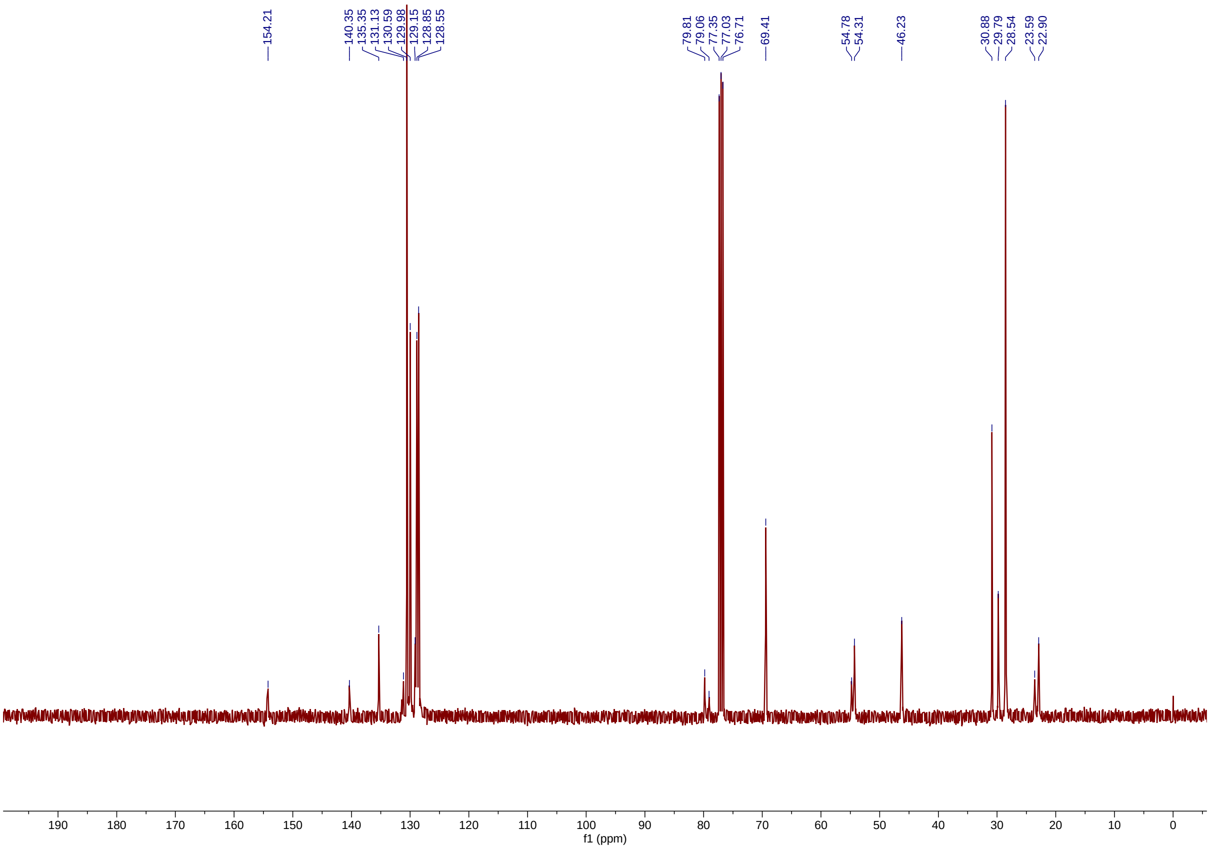

^{1}H (400 MHz) and ^{13}C (101 MHz) spectra of compound 6b in $CDCl_3$

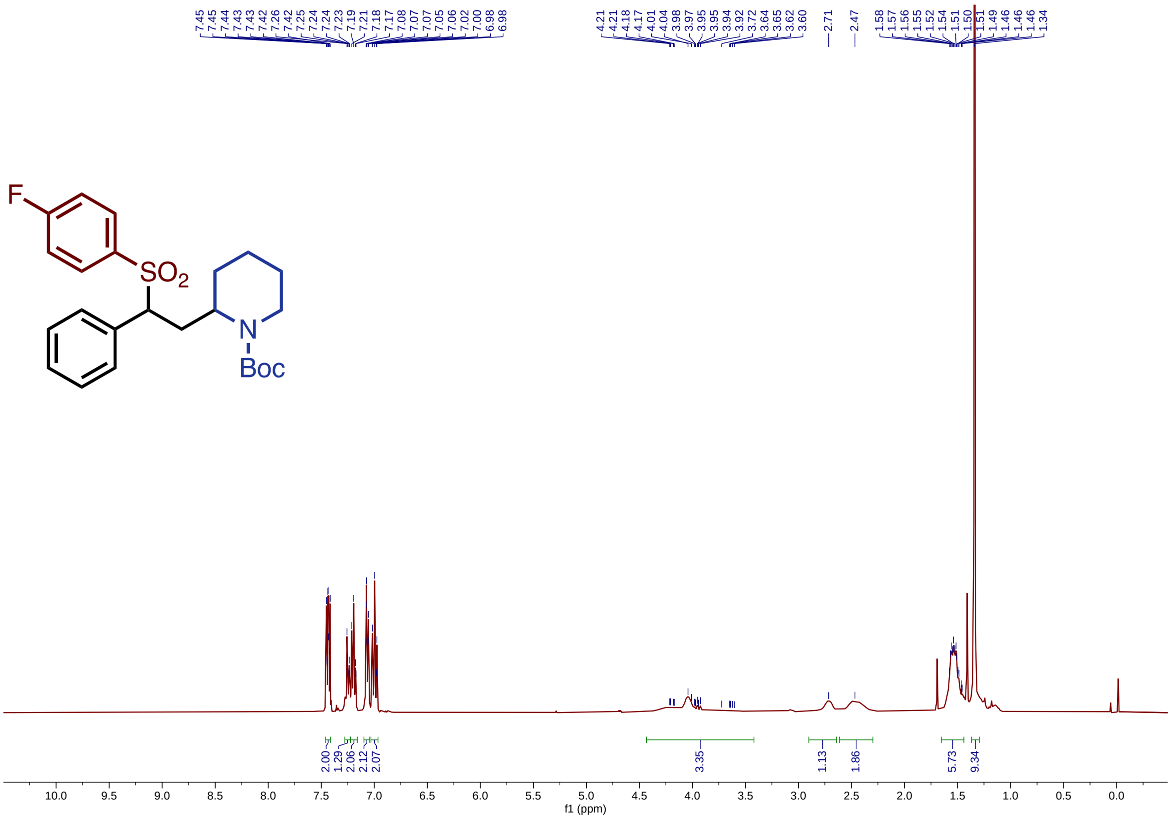

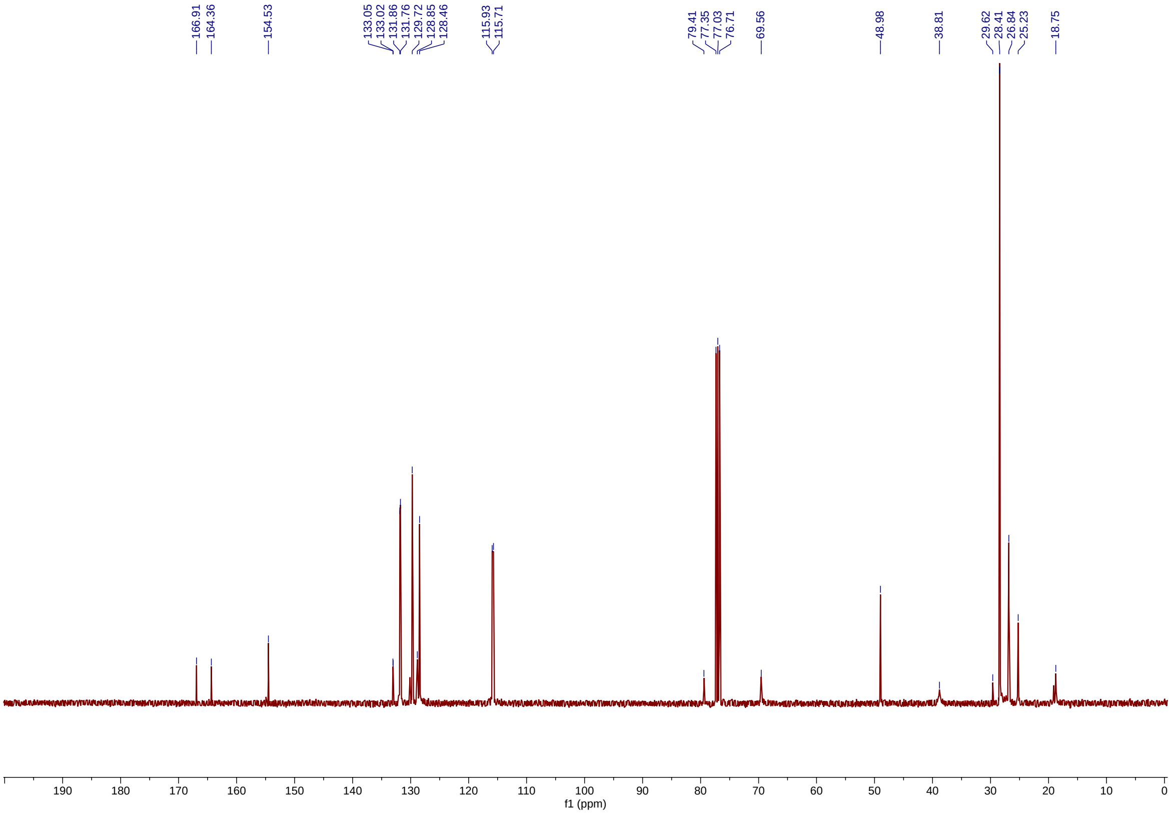

^{1}H (400 MHz) and ^{13}C (101 MHz) spectra of compound 6c in $CDCl_3$

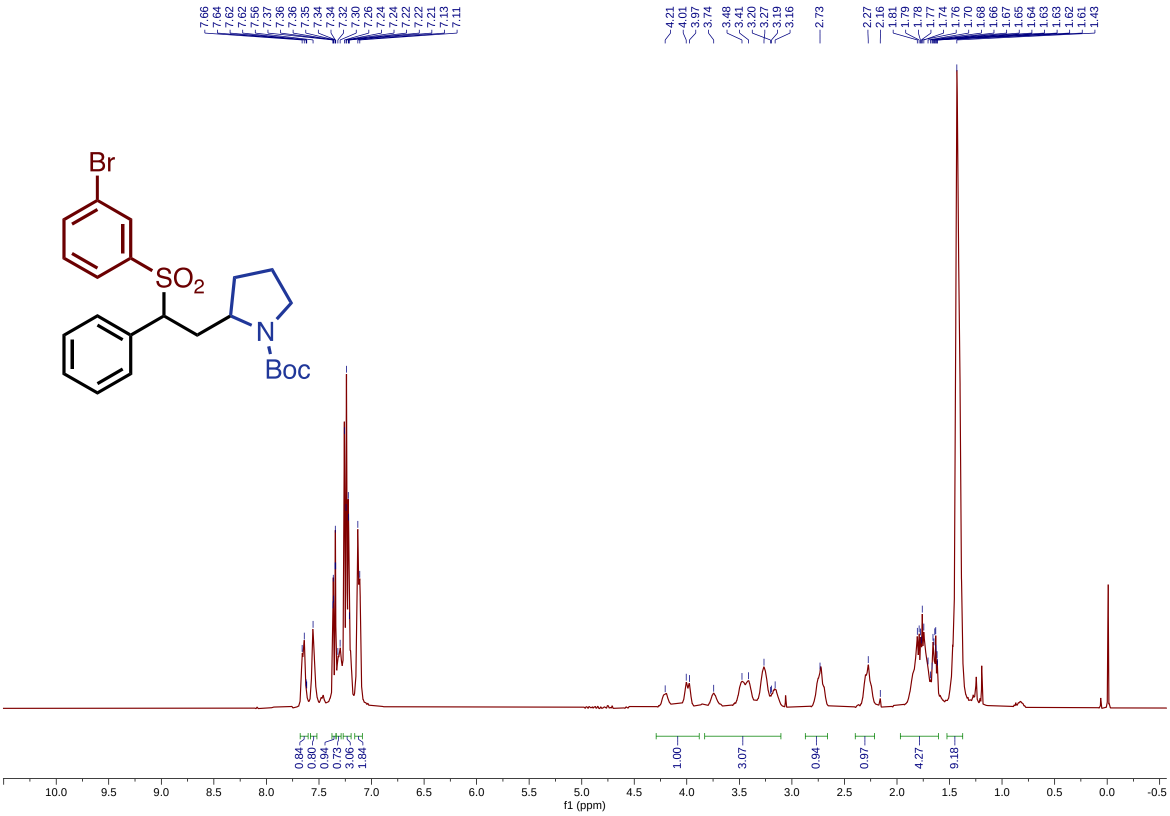

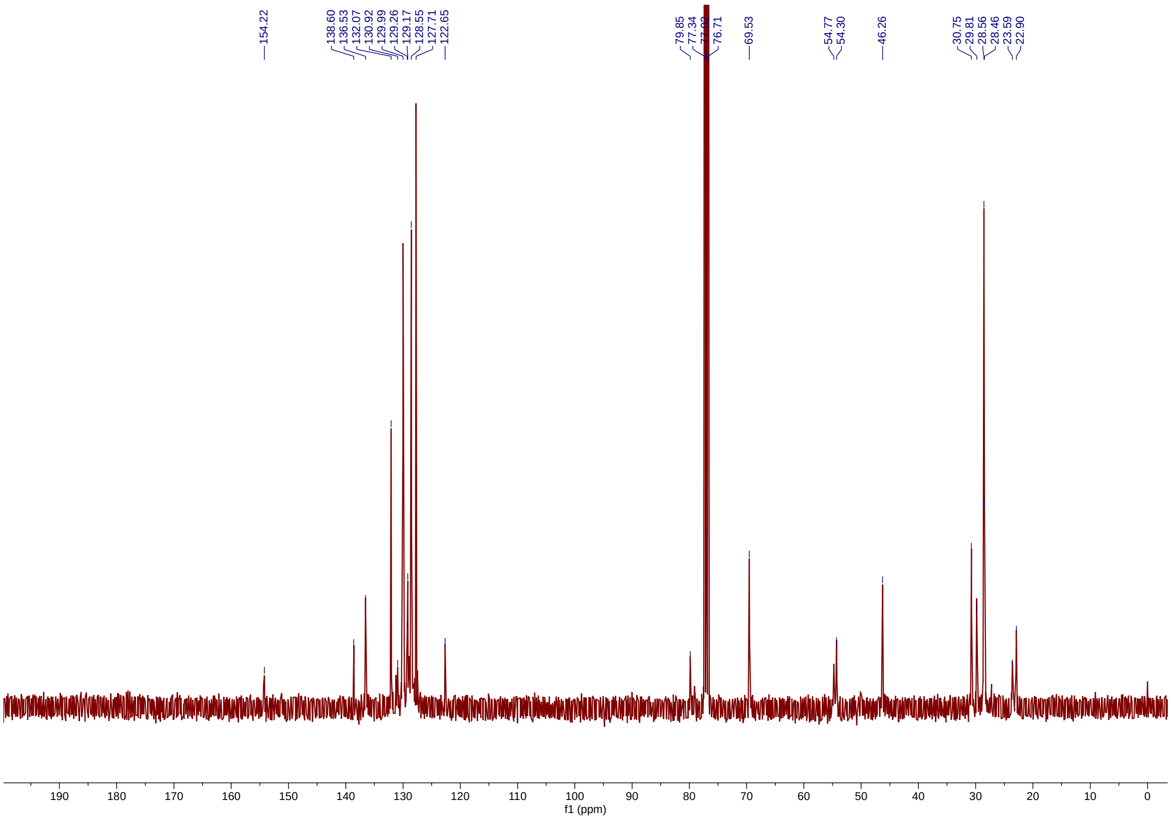

^{1}H (400 MHz) and ^{13}C (101 MHz) spectra of compound 6d in $CDCl_3$

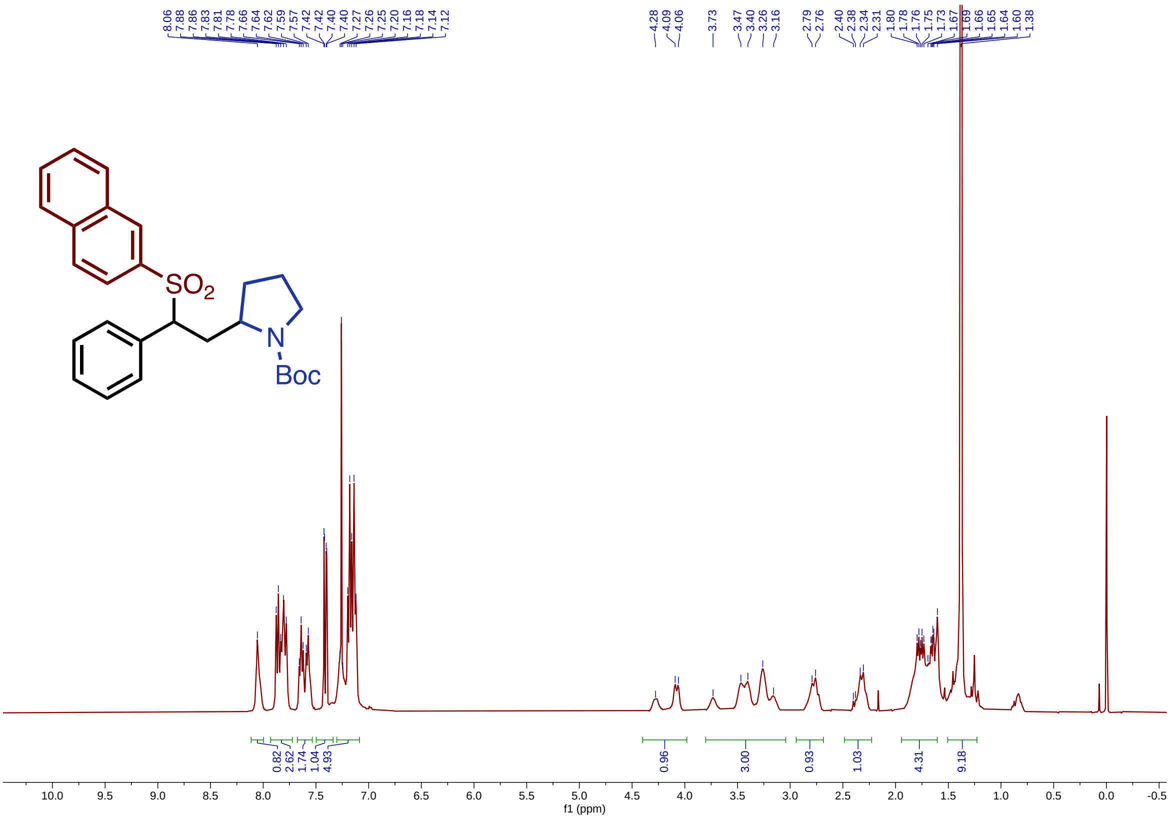

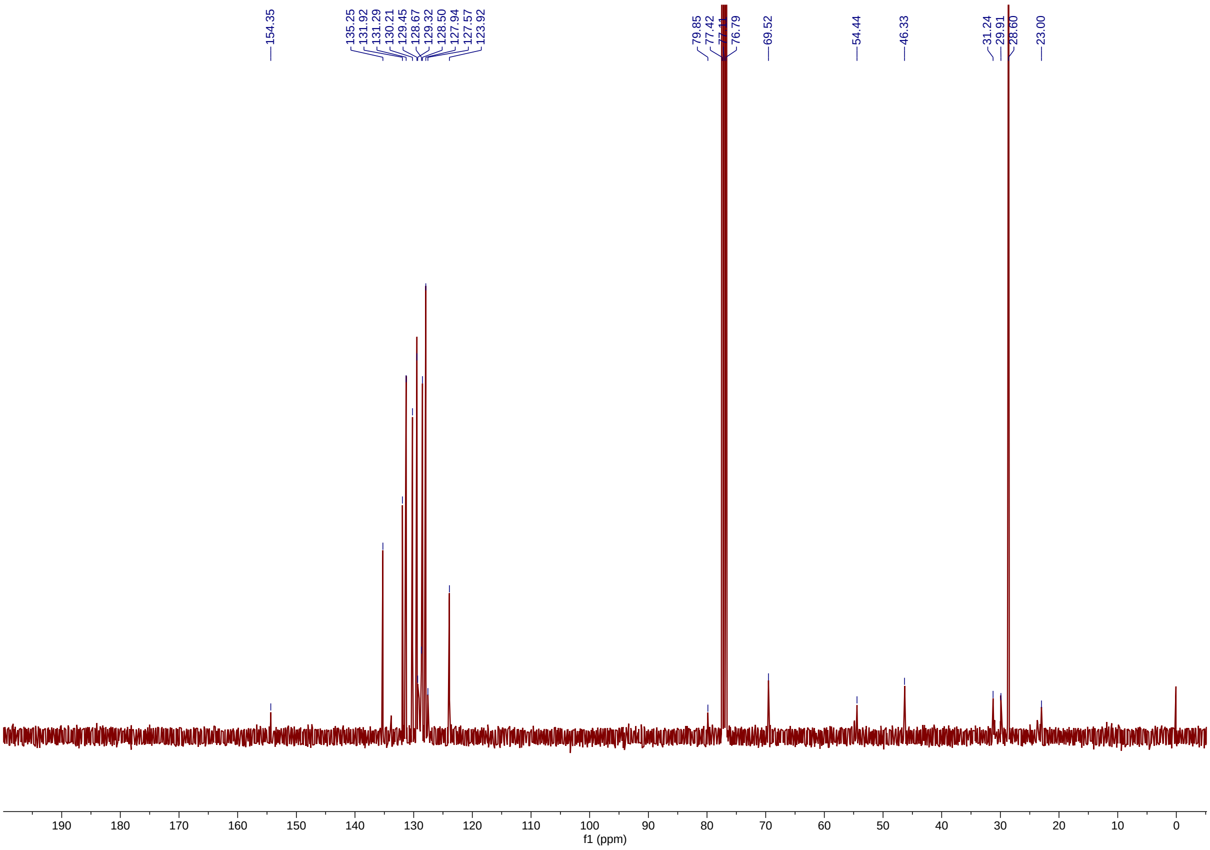

^{1}H (400 MHz) and ^{13}C (101 MHz) spectra of compound 6e in $CDCl_3$

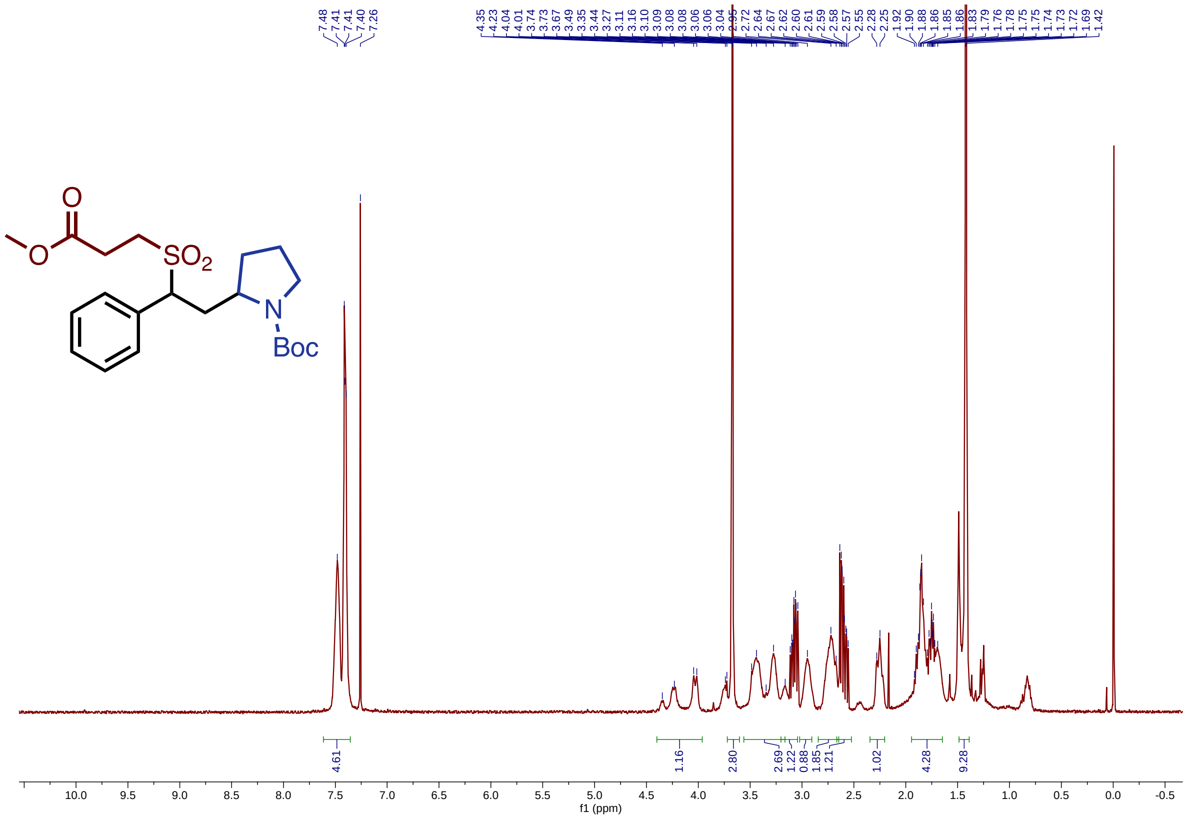

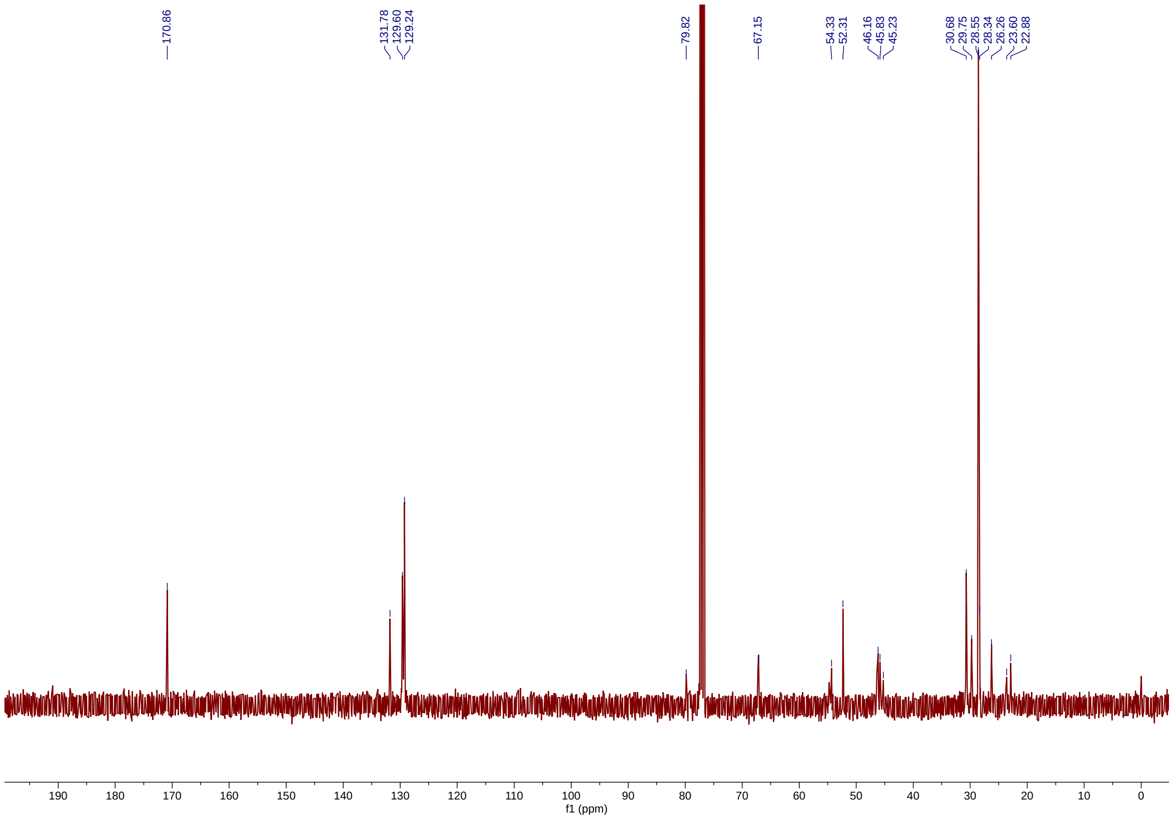

^{1}H (400 MHz) and ^{13}C (101 MHz) spectra of compound 6g in $CDCl_3$

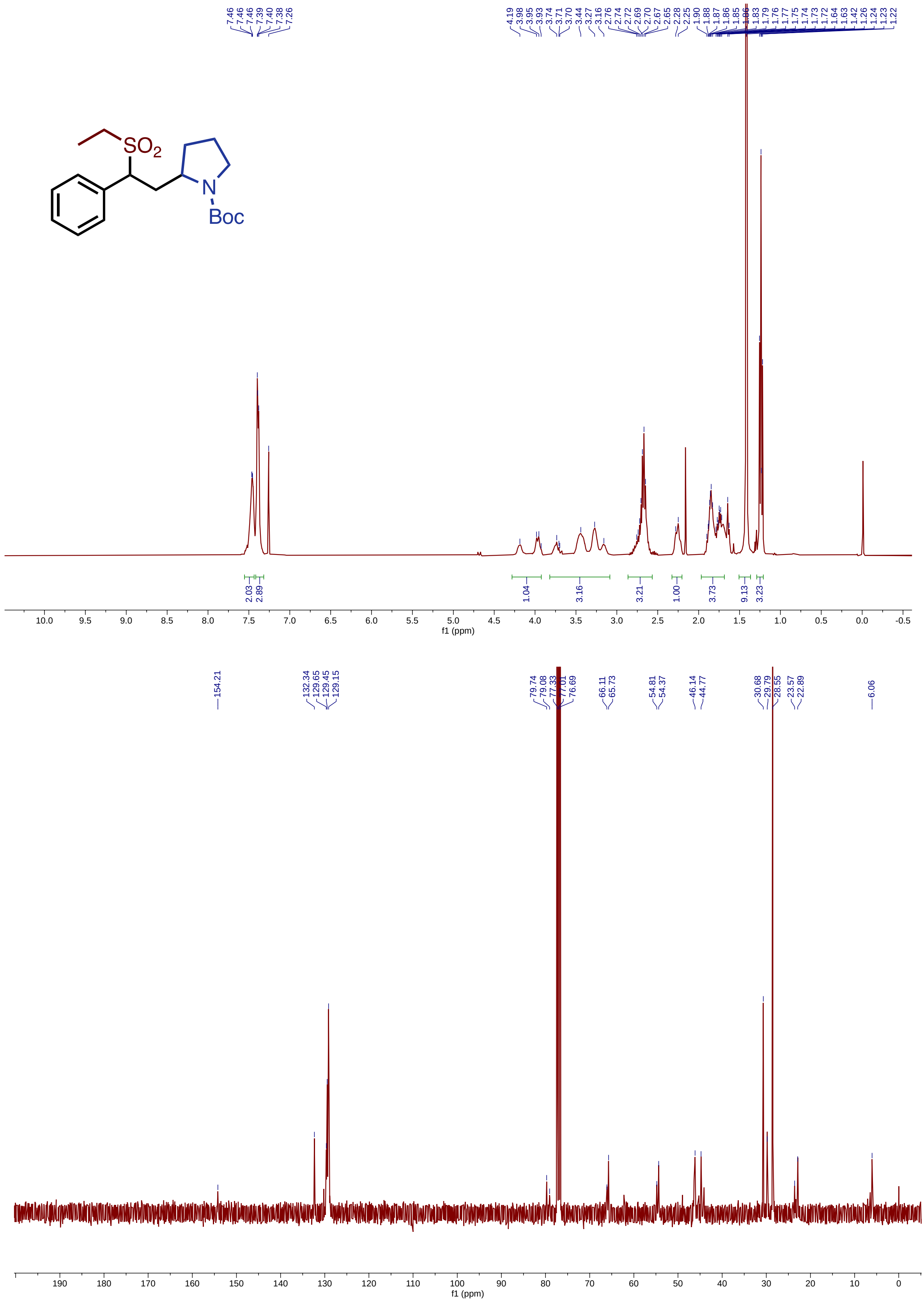

^{1}H (400 MHz) and ^{13}C (101 MHz) spectra of compound 6h in $CDCl_3$

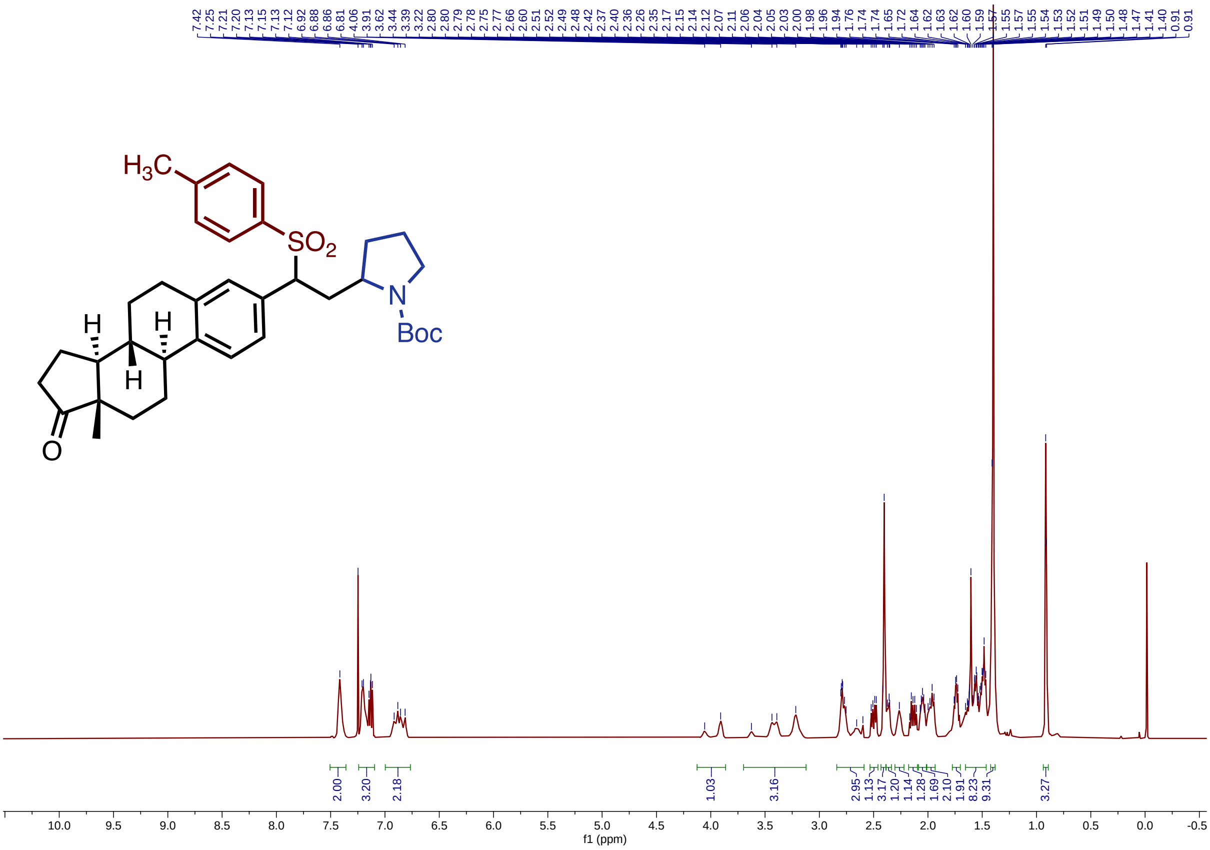

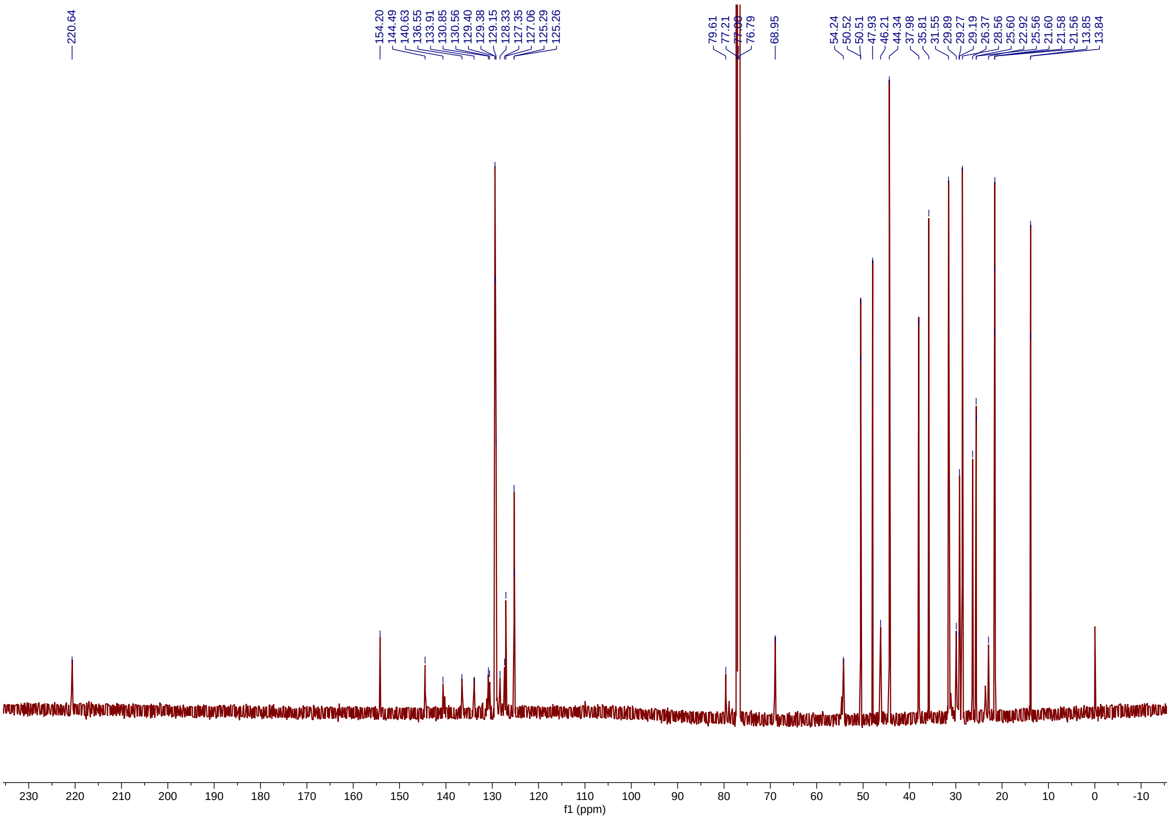

^{1}H (600 MHz) and ^{13}C (151 MHz) spectra of compound 7a in $CDCl_3$

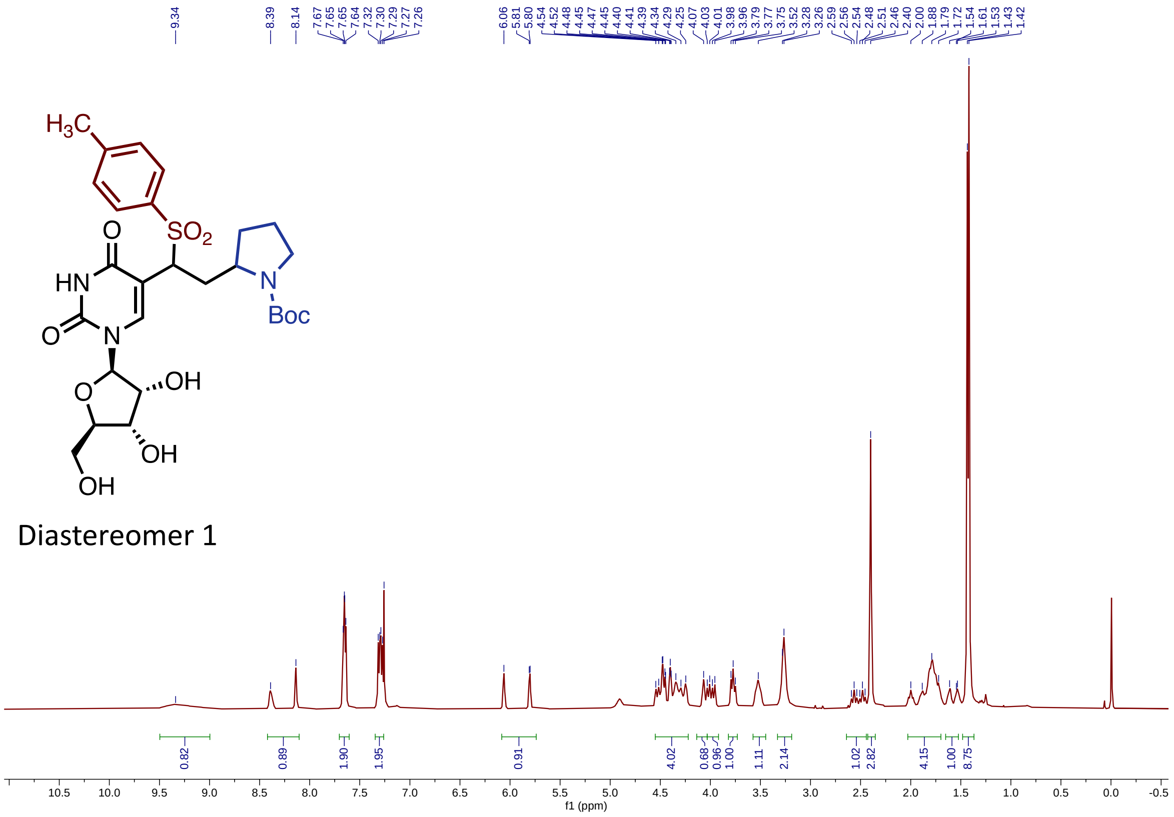

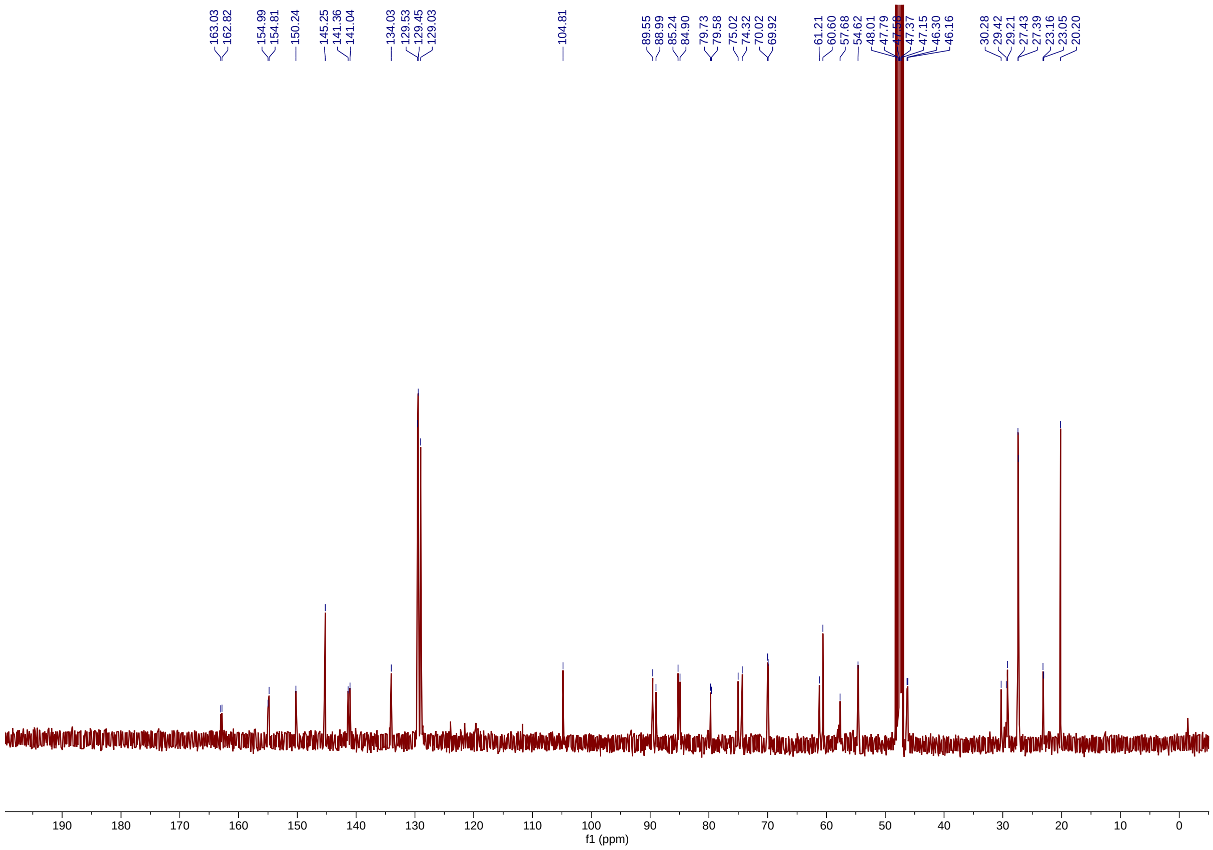

^{1}H (400 MHz) and ^{13}C (101 MHz) spectra of compound 7b in $CDCl_3$ and MeOD

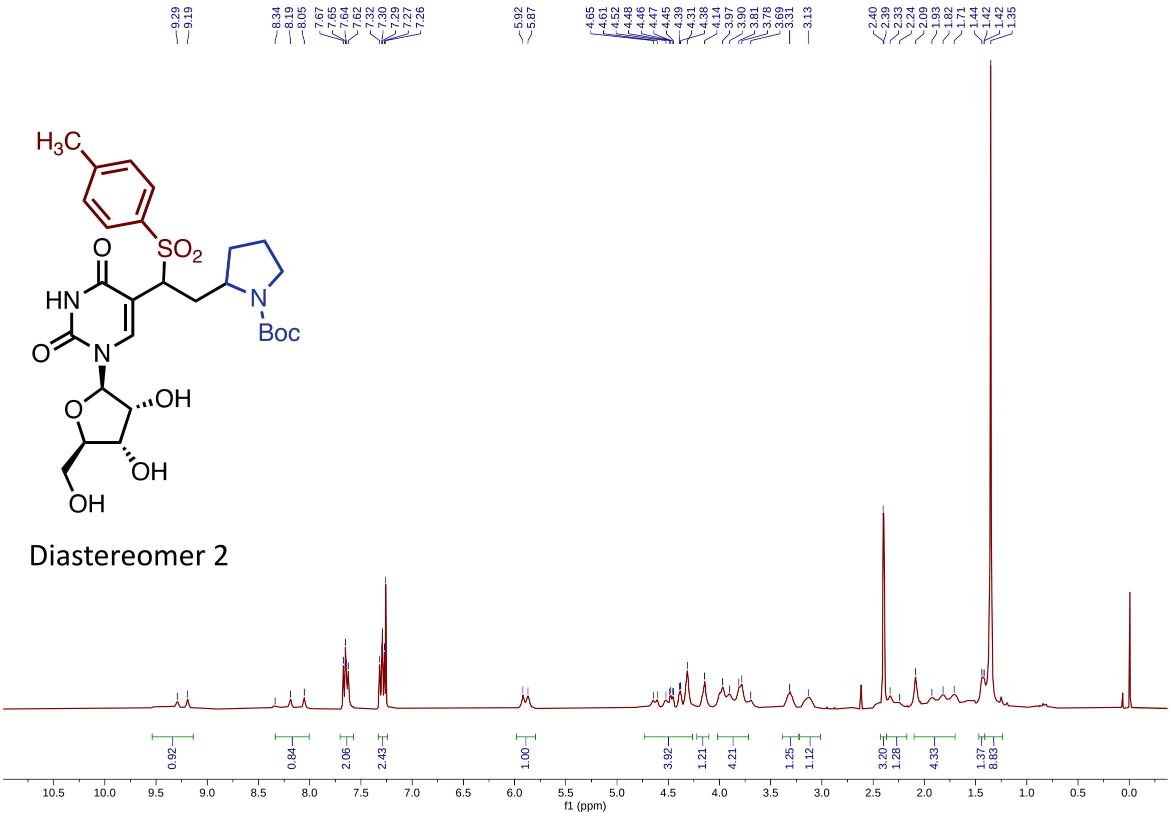

Diastereomer 2

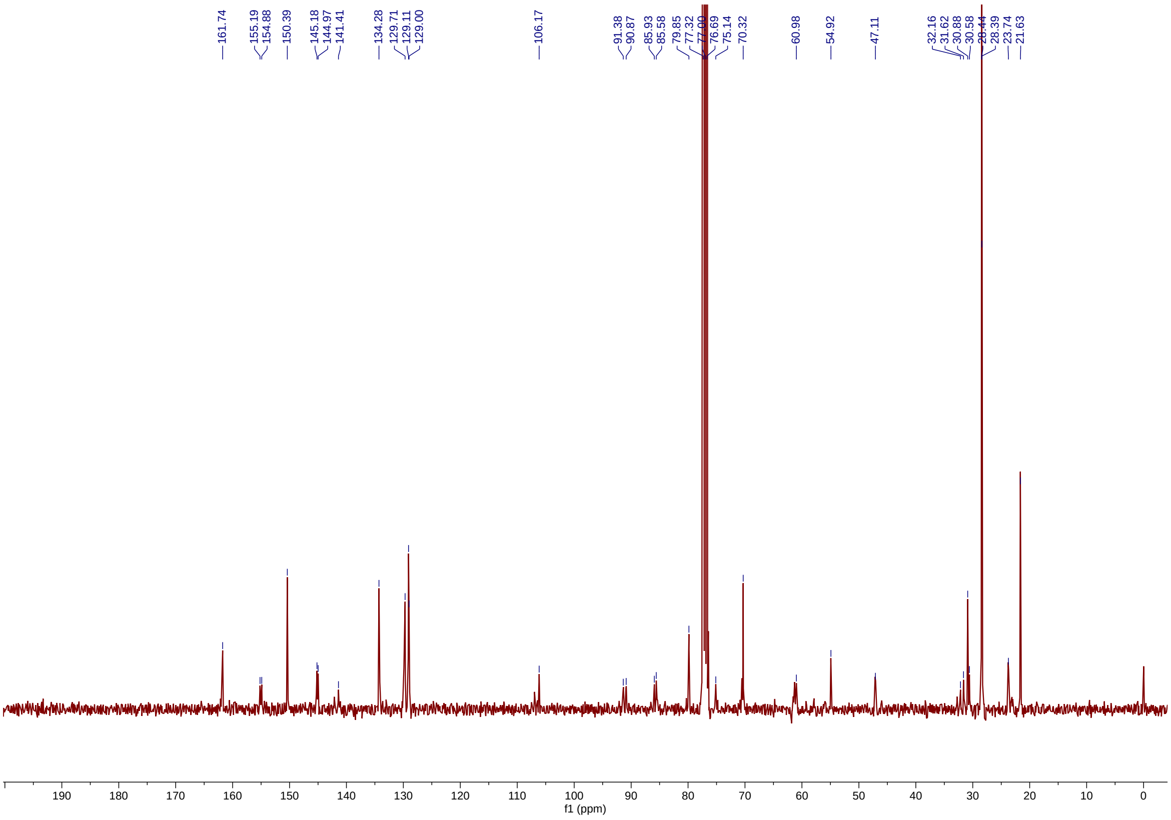

^{1}H (400 MHz) and ^{13}C (101 MHz) spectra of compound 7b in $CDCl_3$

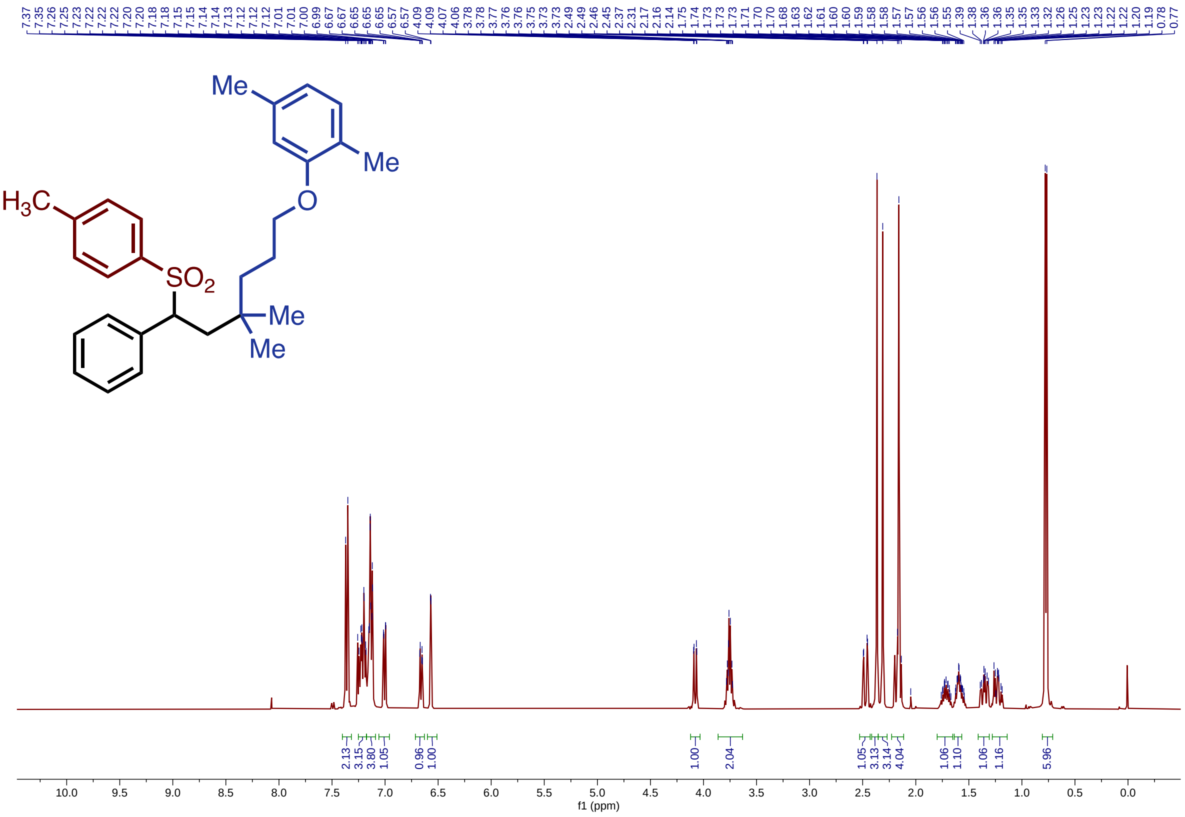

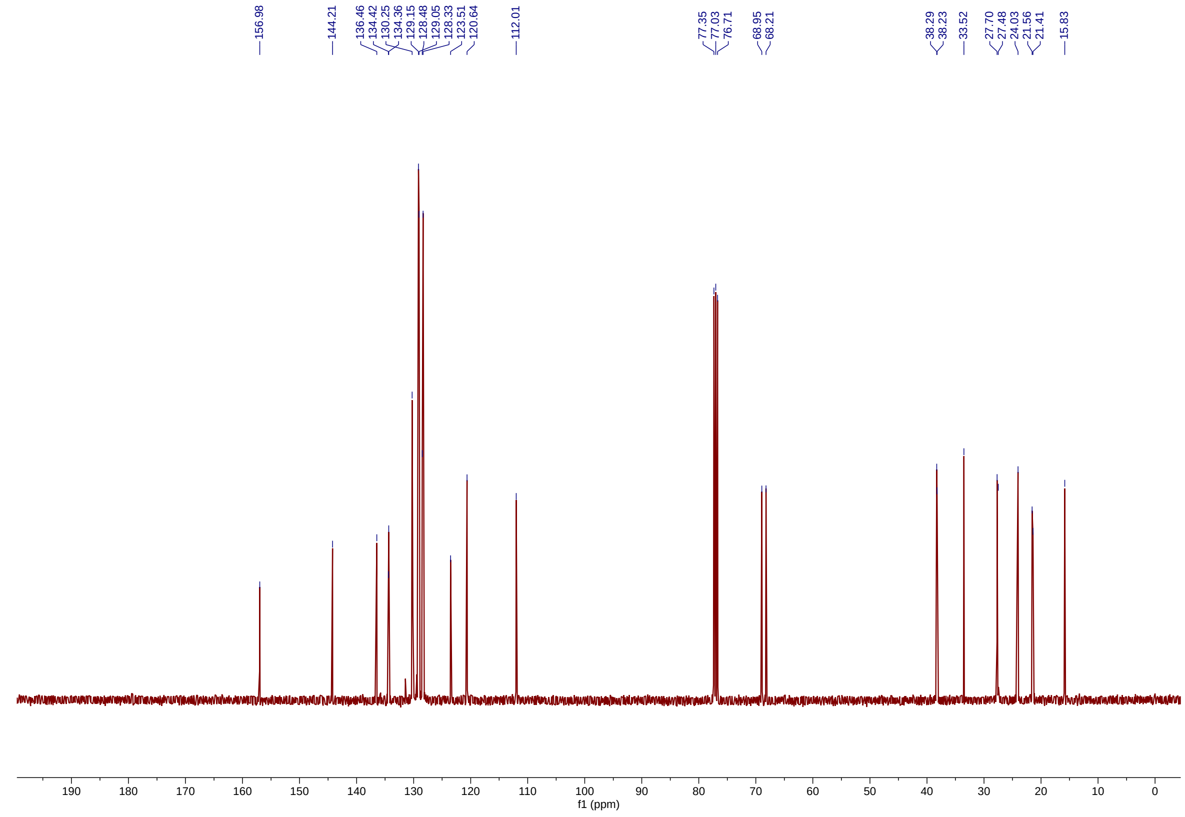

^{1}H (400 MHz) and ^{13}C (101 MHz) spectra of compound 7c in $CDCl_3$

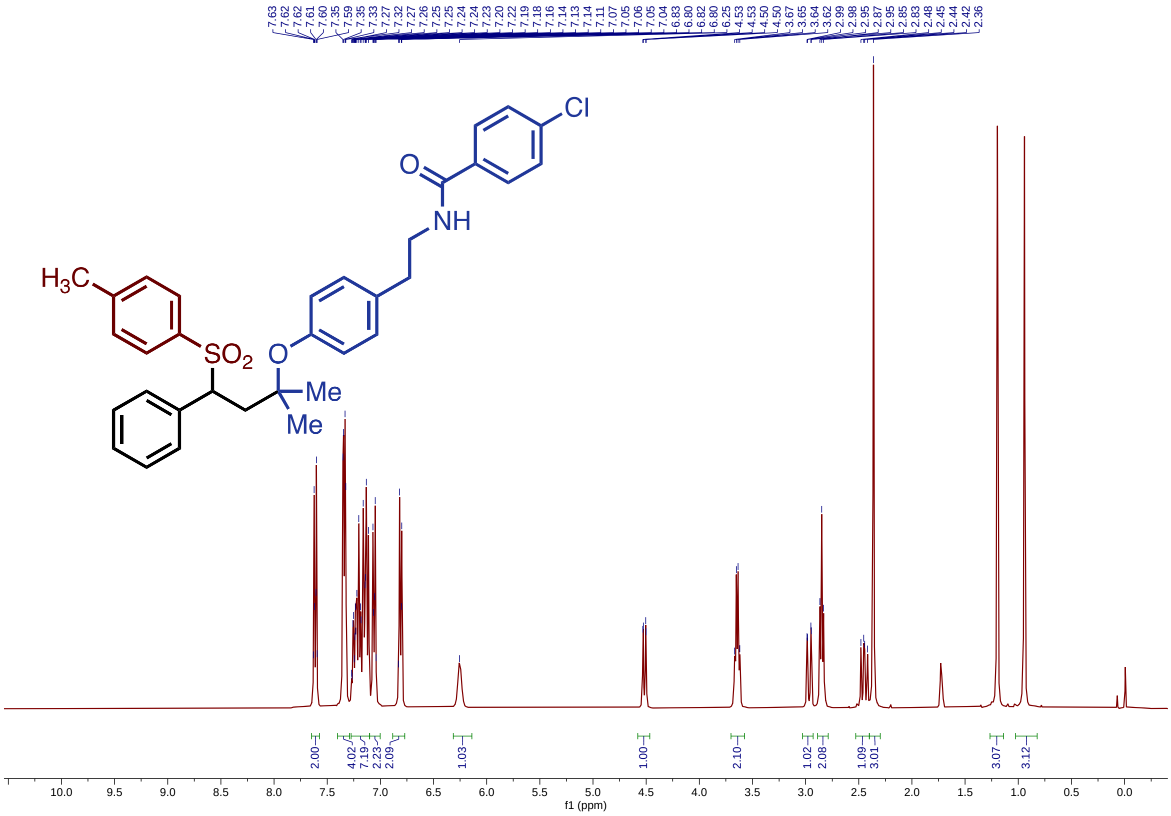

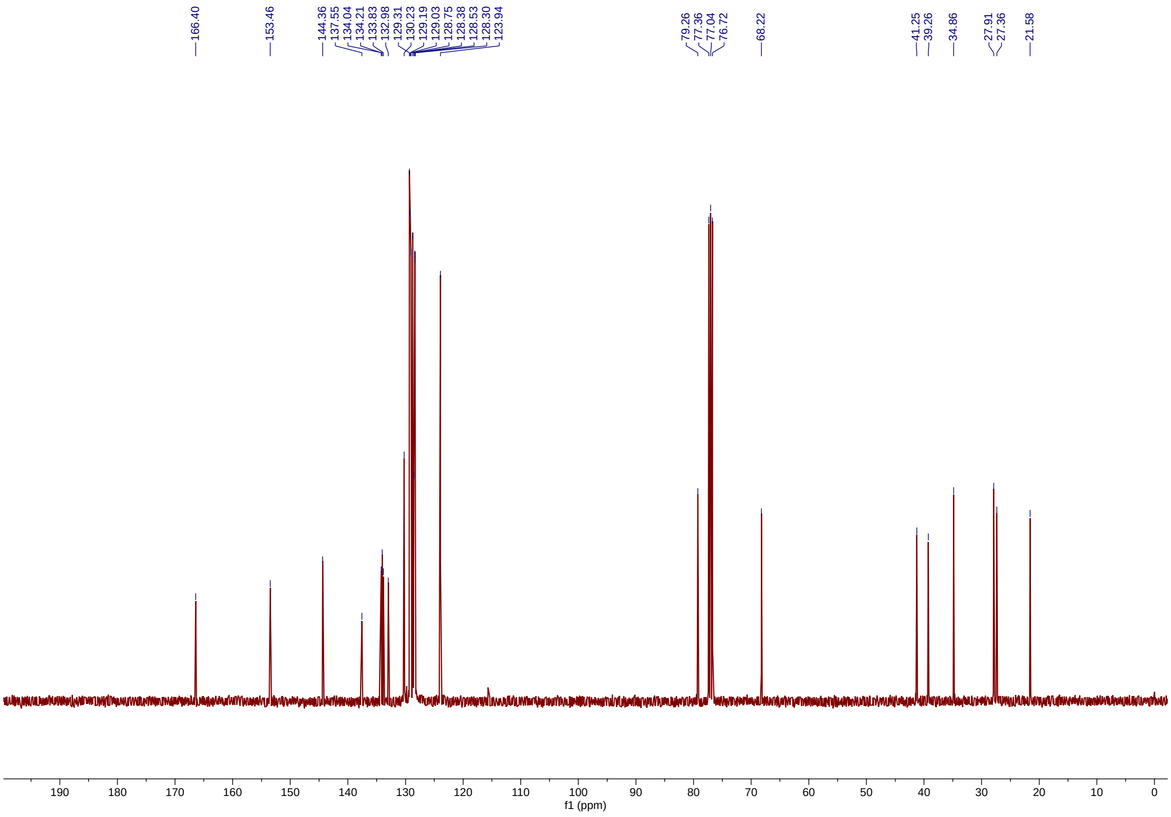

^{1}H (400 MHz) and ^{13}C (101 MHz) spectra of compound 7d in $CDCl_3$

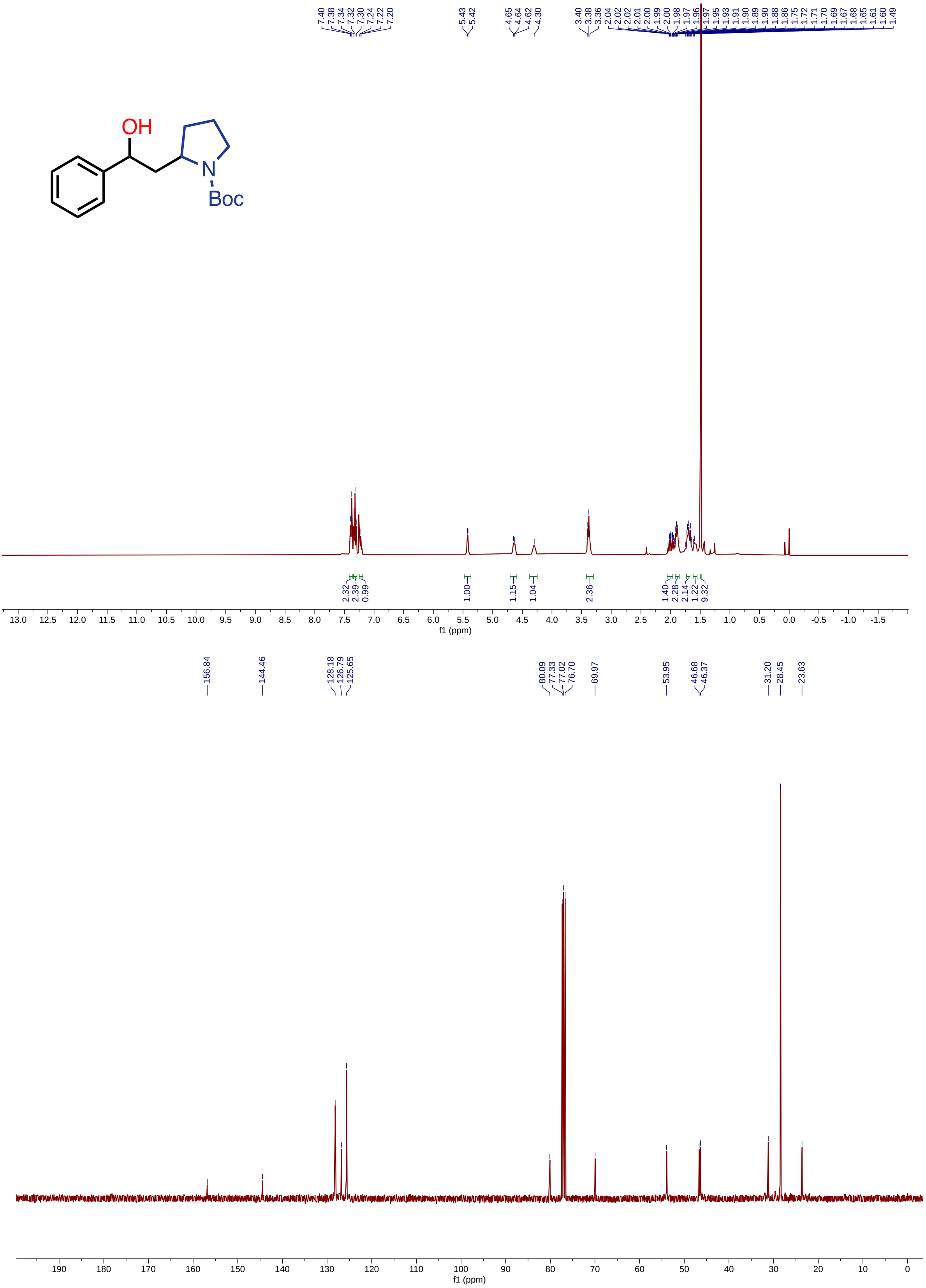

^{1}H (400 MHz) and ^{13}C (101 MHz) spectra of compound 8a in $CDCl_3$

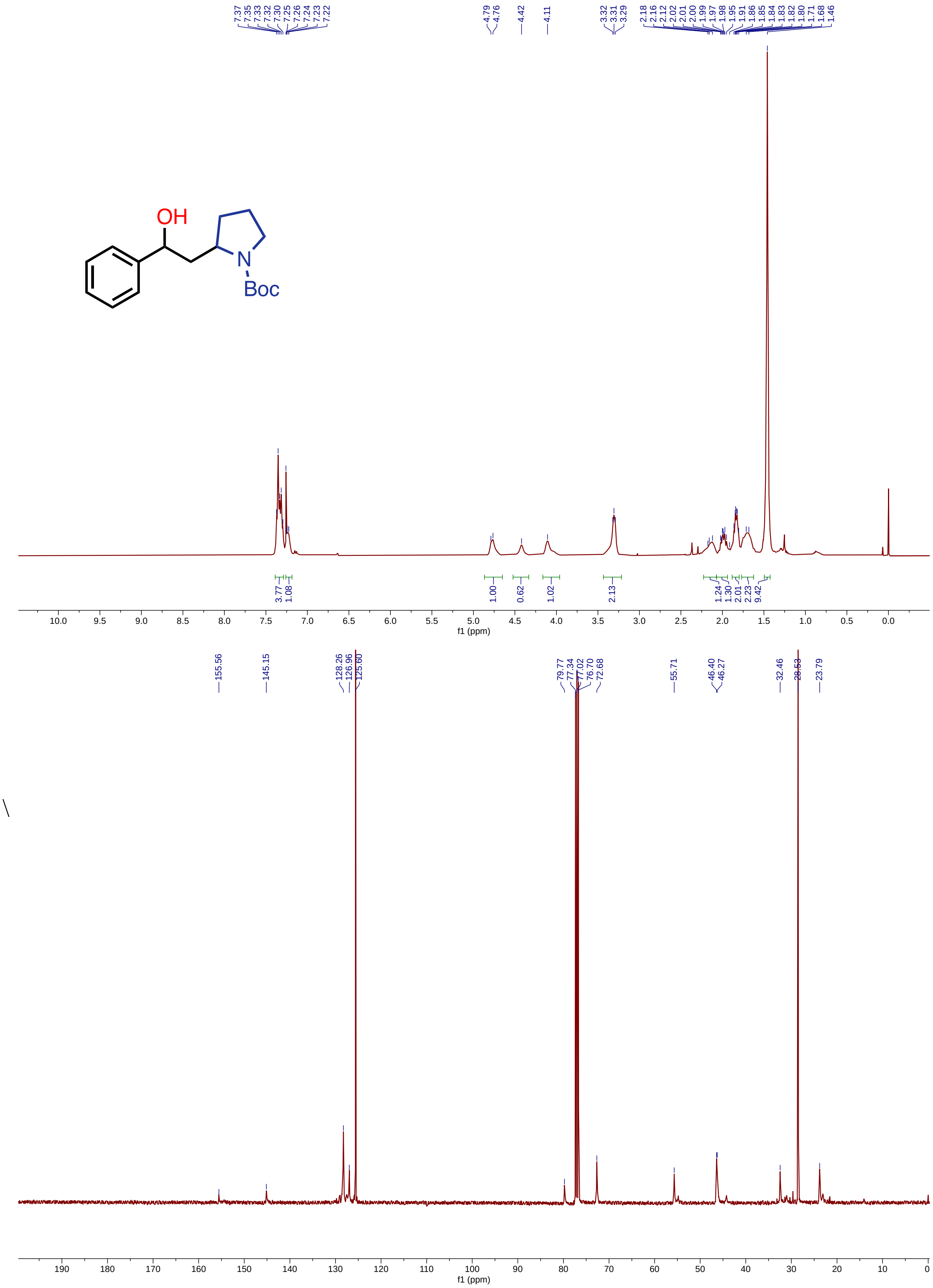

^{1}H (400 MHz) and ^{13}C (101 MHz) spectra of compound 8b in $CDCl_3$

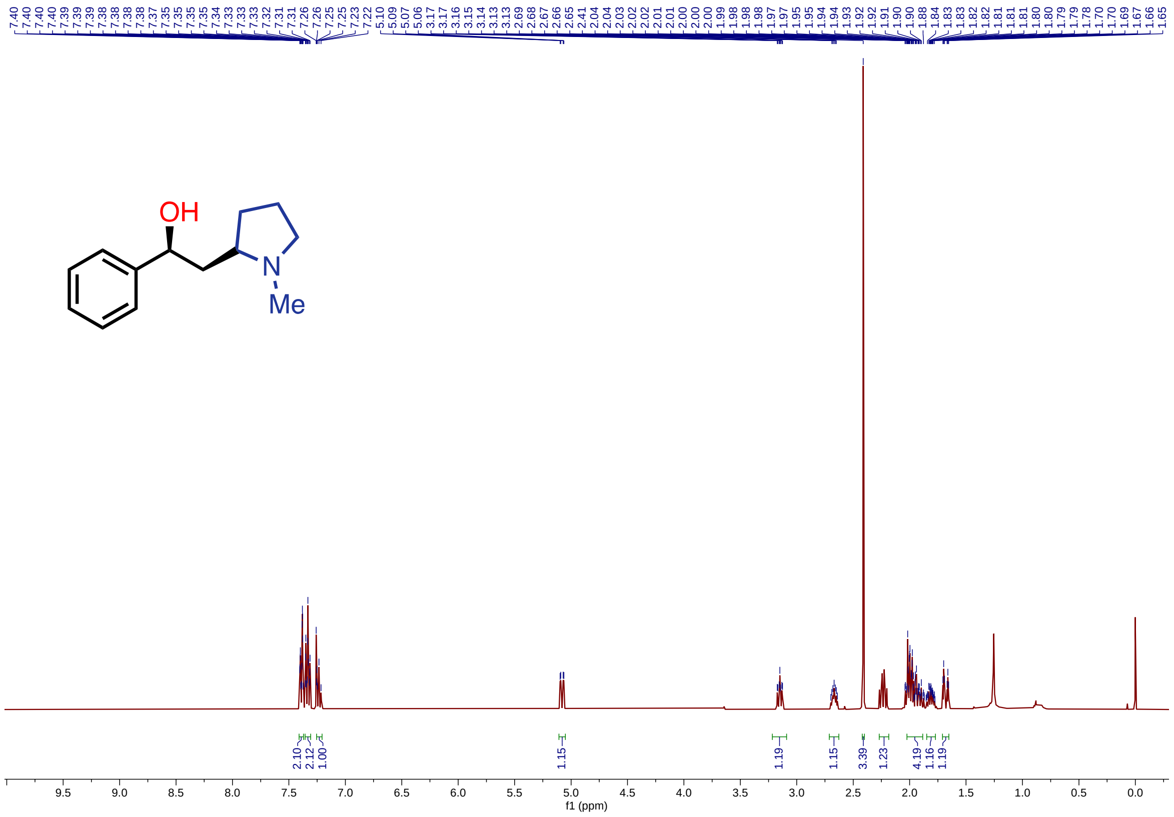

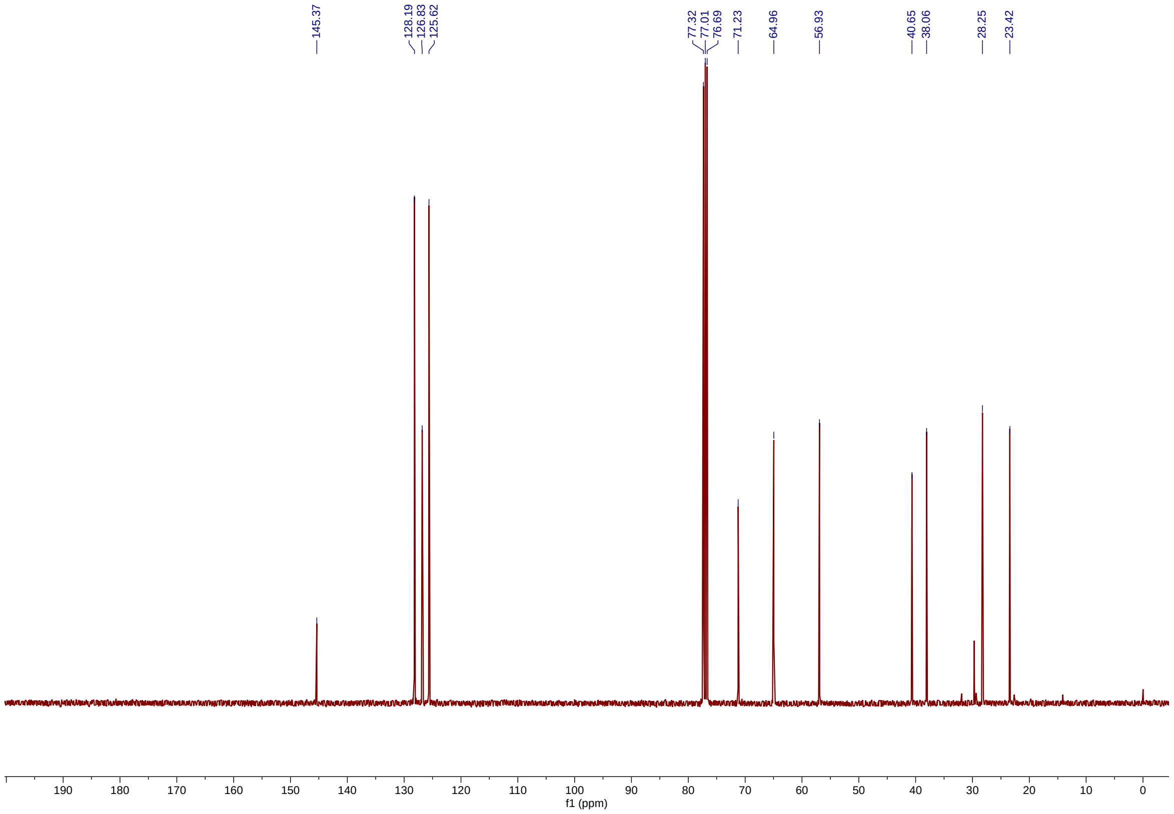

^{1}H (400 MHz) and ^{13}C (101 MHz) spectra of (±)-Pyrrolallosedamine in $CDCl_3$

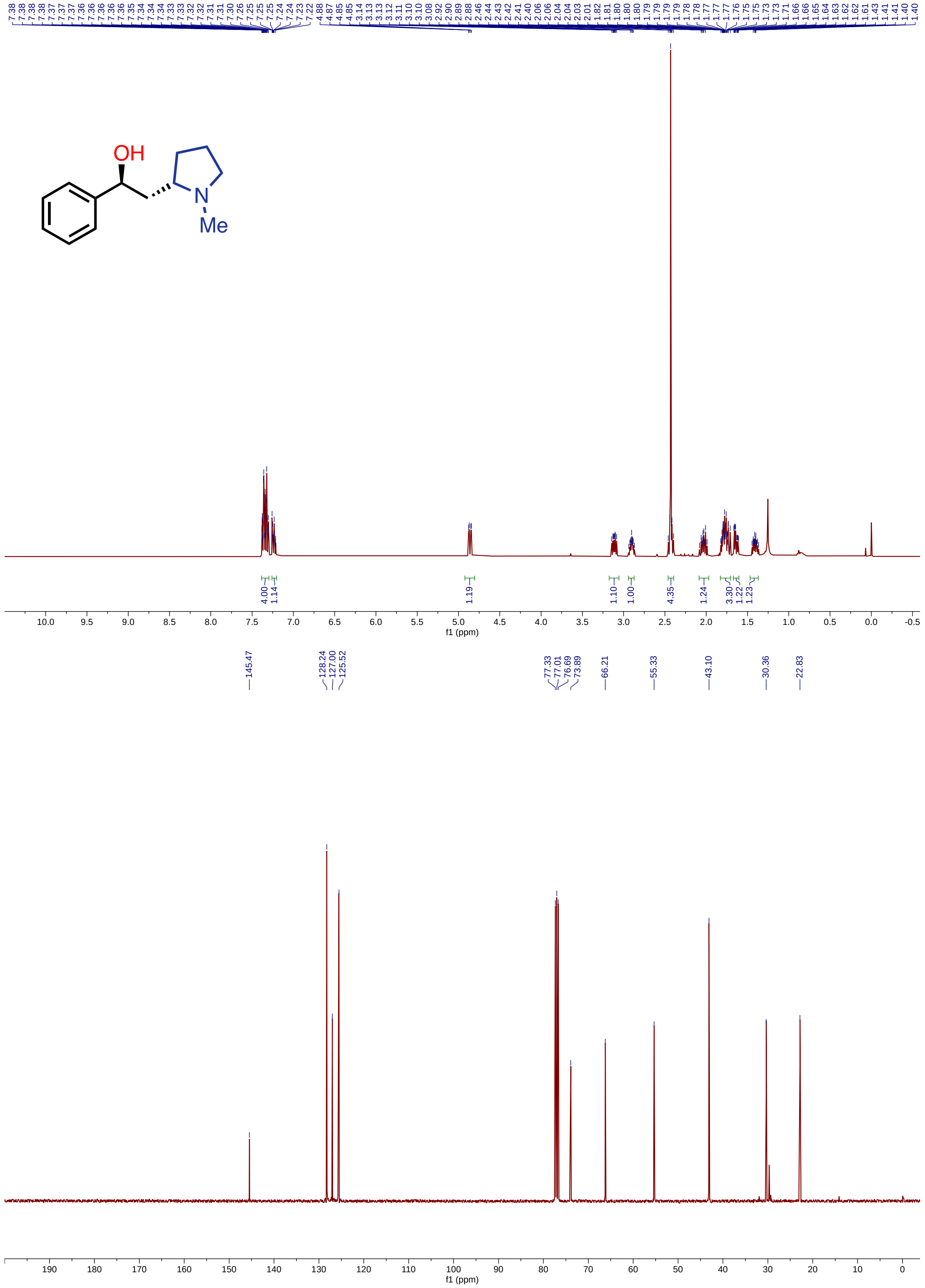

^{1}H (400 MHz) and ^{13}C (101 MHz) spectra of (±)-Pyrrolsedamine in $CDCl_3$

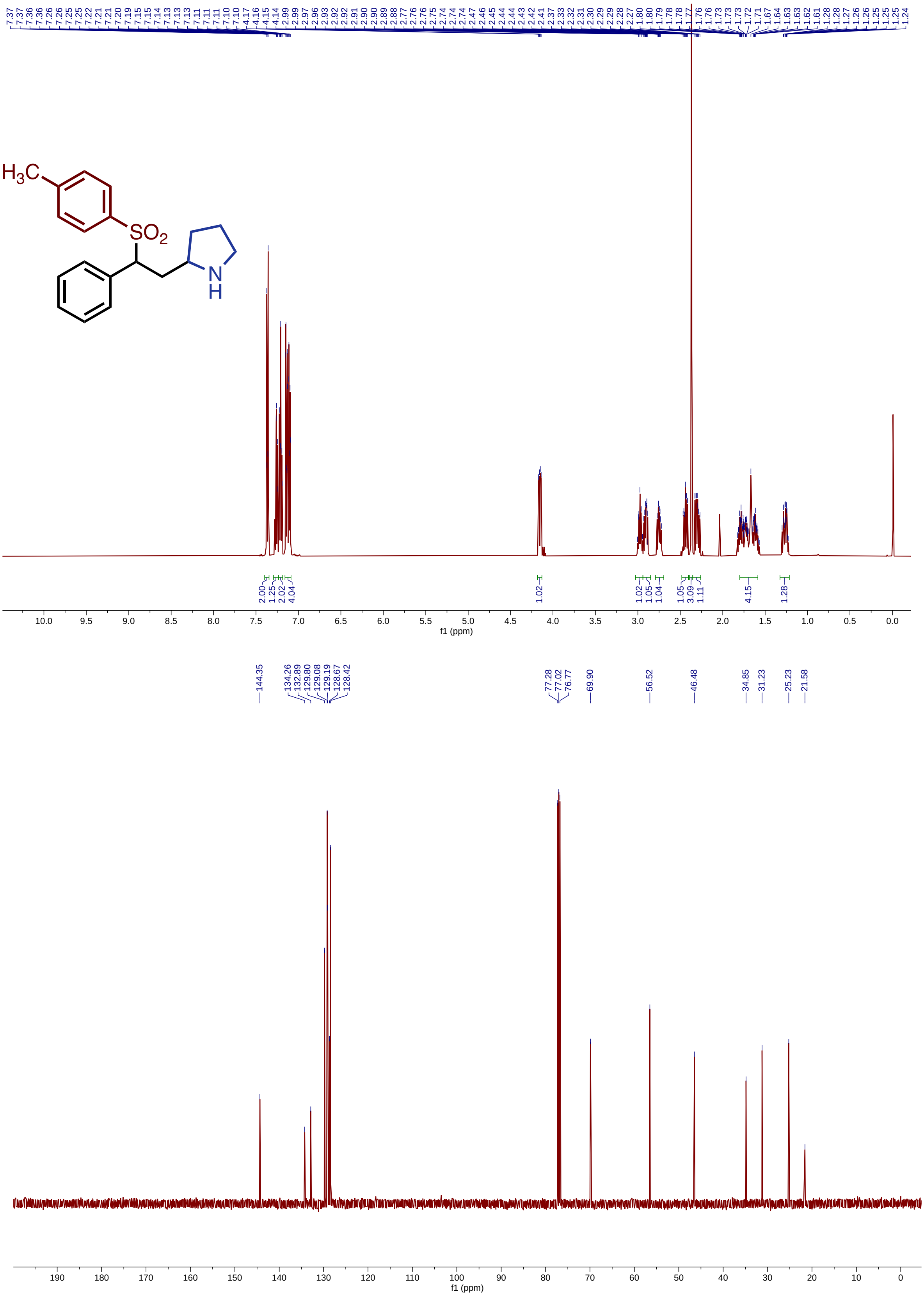

^{1}H (500 MHz) and ^{13}C (126 MHz) spectra of compound 9 in $CDCl_3$

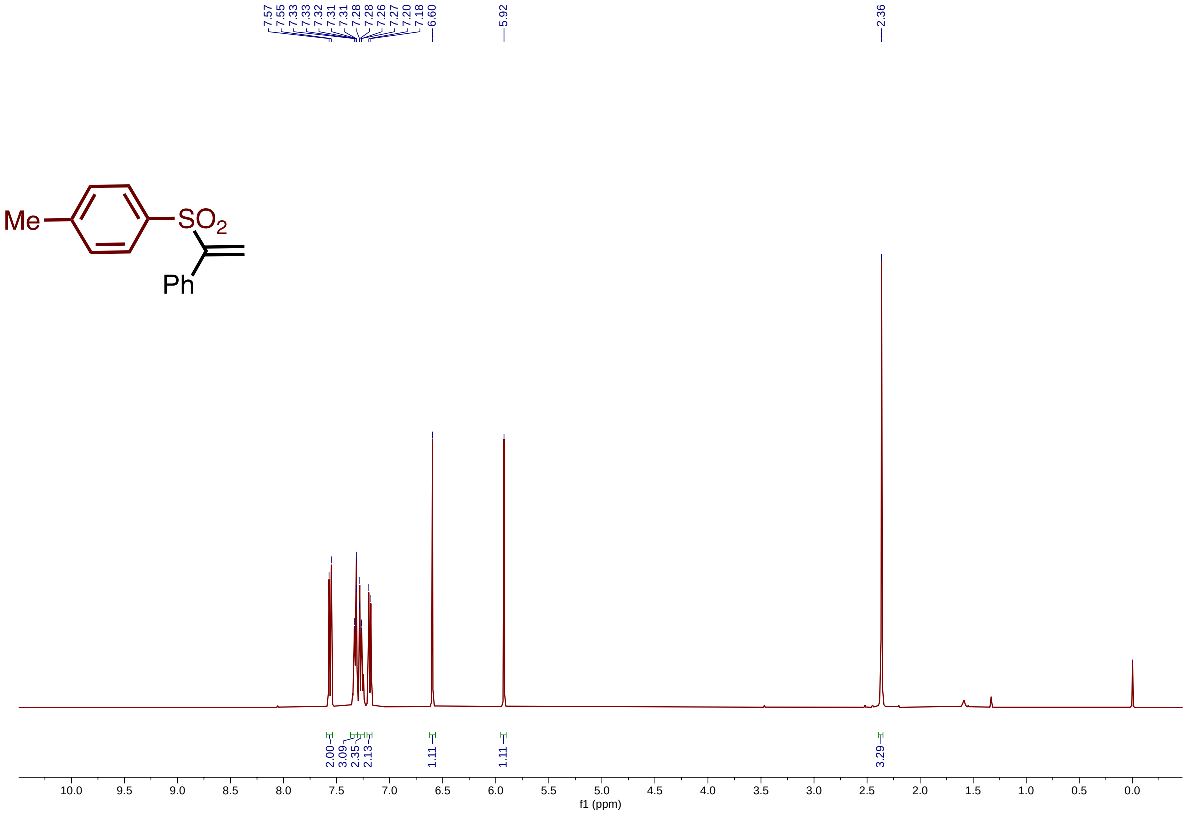

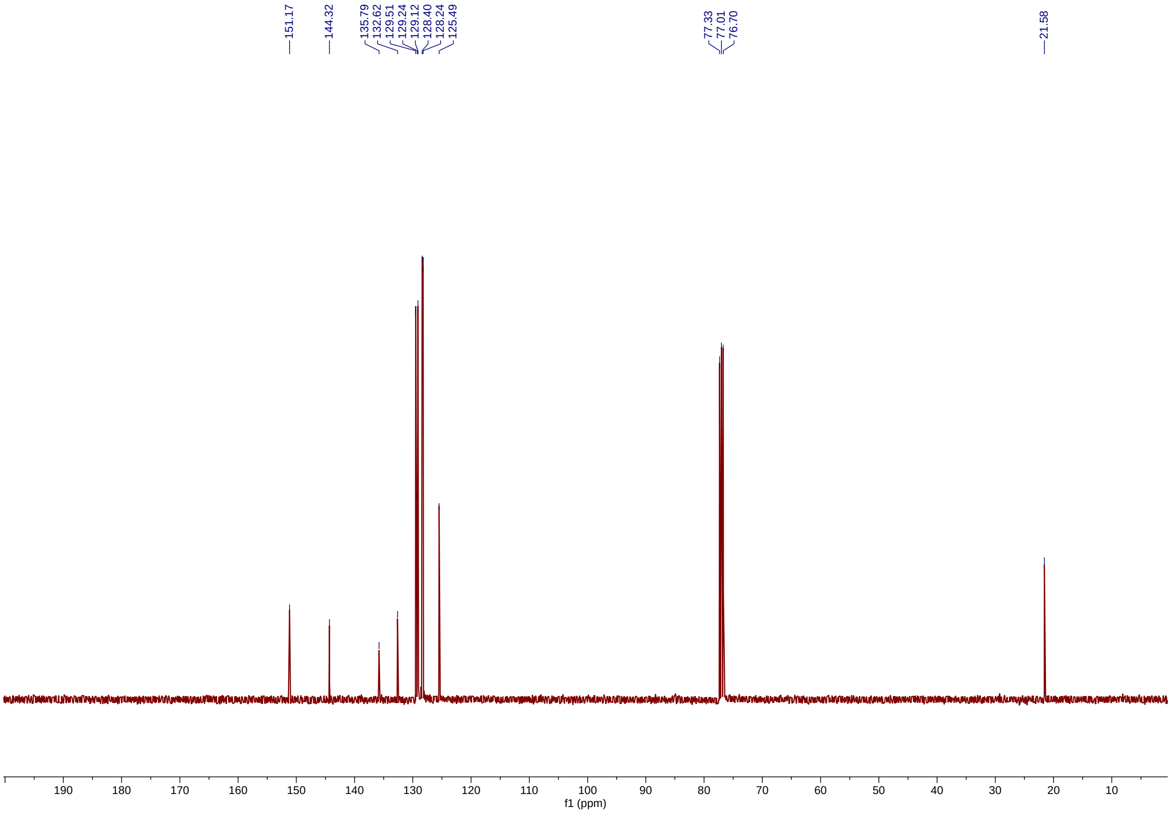

^{1}H (400 MHz) and ^{13}C (101 MHz) spectra of compound 10 in $CDCl_3$

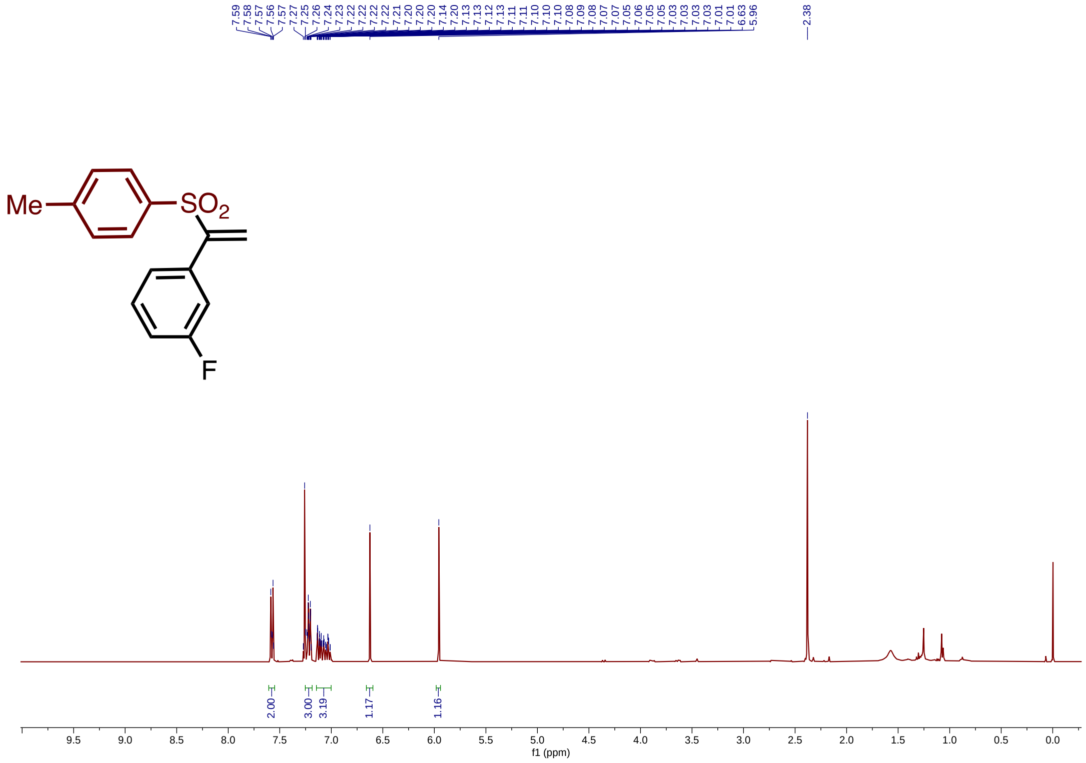

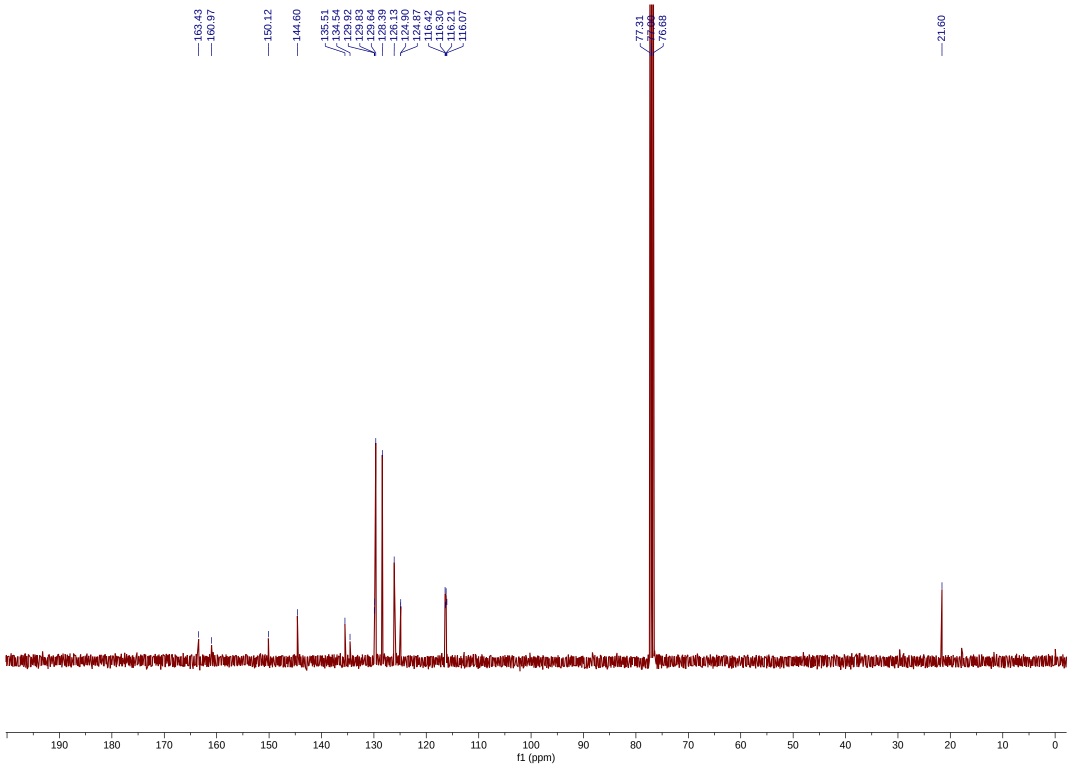

^{1}H (400 MHz) and ^{13}C (101 MHz) spectra of compound 10f in CDCl$_3$
